# The genome of the water strider *Gerris buenoi* reveals expansions of gene repertoires associated with adaptations to life on the water

**DOI:** 10.1186/s12864-018-5163-2

**Published:** 2018-11-21

**Authors:** David Armisén, Rajendhran Rajakumar, Markus Friedrich, Joshua B. Benoit, Hugh M. Robertson, Kristen A. Panfilio, Seung-Joon Ahn, Monica F. Poelchau, Hsu Chao, Huyen Dinh, Harsha Vardhan Doddapaneni, Shannon Dugan, Richard A. Gibbs, Daniel S. T. Hughes, Yi Han, Sandra L. Lee, Shwetha C. Murali, Donna M. Muzny, Jiaxin Qu, Kim C. Worley, Monica Munoz-Torres, Ehab Abouheif, François Bonneton, Travis Chen, Li-Mei Chiang, Christopher P. Childers, Andrew G. Cridge, Antonin J. J. Crumière, Amelie Decaras, Elise M. Didion, Elizabeth J. Duncan, Elena N. Elpidina, Marie-Julie Favé, Cédric Finet, Chris G. C. Jacobs, Alys M. Cheatle Jarvela, Emily C. Jennings, Jeffery W. Jones, Maryna P. Lesoway, Mackenzie R. Lovegrove, Alexander Martynov, Brenda Oppert, Angelica Lillico-Ouachour, Arjuna Rajakumar, Peter Nagui Refki, Andrew J. Rosendale, Maria Emilia Santos, William Toubiana, Maurijn van der Zee, Iris M. Vargas Jentzsch, Aidamalia Vargas Lowman, Severine Viala, Stephen Richards, Abderrahman Khila

**Affiliations:** 1 Institut de Génomique Fonctionnelle de Lyon, Université de Lyon, Université Claude Bernard Lyon 1, CNRS UMR 5242, Ecole Normale Supérieure de Lyon 46, allée d’Italie, 69364 Lyon Cedex 07, France; 2 Department of Molecular Genetics & Microbiology and UF Genetics Institute, University of Florida, 2033 Mowry Road, Gainesville, FL 32610-3610 USA; 3 Department of Biological Sciences, Wayne State University, Detroit, MI 48202 USA; 4 Department of Biological Sciences, McMicken College of Arts and Sciences, University of Cincinnati, 318 College Drive, Cincinnati, OH 45221-0006 USA; 5 Department of Entomology, University of Illinois at Urbana-Champaign, Urbana, IL 61801 USA; 6 Institute for Zoology: Developmental Biology, University of Cologne, Zülpicher Str. 47b, 50674 Cologne, Germany; 7 School of Life Sciences, University of Warwick, Gibbet Hill Campus, Coventry, CV4 7AL UK; 8 USDA-ARS Horticultural Crops Research Unit, 3420 NW Orchard Avenue, Corvallis, OR 97330 USA; 9 Department of Crop and Soil Science, Oregon State University, 3050 SW Campus Way, Corvallis, OR 97331 USA; 10 USDA Agricultural Research Service, National Agricultural Library, Beltsville, MD 20705 USA; 11 Human Genome Sequencing Center, Department of Human and Molecular Genetics, Baylor College of Medicine, One Baylor Plaza, Houston, TX 77030 USA; 12 Howard Hughes Medical Institute, University of Washington, Seattle, WA 98195 USA; 13 Lawrence Berkeley National Laboratory, Berkeley, CA 94720 USA; 14 Department of Biology, McGill University, 1205 Avenue Docteur Penfield Avenue, Montréal, Québec H3A 1B1 Canada; 15 Laboratory for Evolution and Development, Department of Biochemistry, University of Otago, P.O. Box 56, Dunedin, New Zealand; 16 School of Biology, Faculty of Biological Sciences, University of Leeds, Leeds, LS2 9JT UK; 17 A.N. Belozersky Institute of Physico-Chemical Biology, Moscow State University, Moscow, 119991 Russia; 18 Institute of Biology, Leiden University, Sylviusweg 72, 2333 BE Leiden, Netherlands; 19 Max Planck Institute for Chemical Ecology, Hans-Knöll Strasse 8, 07745 Jena, Germany; 20 Department of Entomology, University of Maryland, College Park, MD 20742 USA; 21 Smithsonian Tropical Research Institute, Apartado Postal 0843-03092, Balboa Ancon, Panama City, Panama; 22 Center of Life Sciences, Skolkovo Institute of Science and Technology, Skolkovo, 143025 Russia; 23 USDA ARS Center for Grain and Animal Health Research, 1515 College Ave., Manhattan, KS-66502 USA; 24 Department of Evolutionary Genetics, Max-Planck-Institut für Evolutionsbiologie, August-Thienemann-Straße 2, 24306 Plön, Germany

**Keywords:** Water striders, Genome sequence, Water surface locomotion, Evolution, Adaptation

## Abstract

**Background:**

Having conquered water surfaces worldwide, the semi-aquatic bugs occupy ponds, streams, lakes, mangroves, and even open oceans. The diversity of this group has inspired a range of scientific studies from ecology and evolution to developmental genetics and hydrodynamics of fluid locomotion. However, the lack of a representative water strider genome hinders our ability to more thoroughly investigate the molecular mechanisms underlying the processes of adaptation and diversification within this group.

**Results:**

Here we report the sequencing and manual annotation of the *Gerris buenoi* (*G. buenoi*) genome; the first water strider genome to be sequenced thus far. The size of the *G. buenoi* genome is approximately 1,000 Mb, and this sequencing effort has recovered 20,949 predicted protein-coding genes. Manual annotation uncovered a number of local (tandem and proximal) gene duplications and expansions of gene families known for their importance in a variety of processes associated with morphological and physiological adaptations to a water surface lifestyle. These expansions may affect key processes associated with growth, vision, desiccation resistance, detoxification, olfaction and epigenetic regulation. Strikingly, the *G. buenoi* genome contains three insulin receptors, suggesting key changes in the rewiring and function of the insulin pathway. Other genomic changes affecting with opsin genes may be associated with wavelength sensitivity shifts in opsins, which is likely to be key in facilitating specific adaptations in vision for diverse water habitats.

**Conclusions:**

Our findings suggest that local gene duplications might have played an important role during the evolution of water striders. Along with these findings, the sequencing of the *G. buenoi* genome now provides us the opportunity to pursue exciting research opportunities to further understand the genomic underpinnings of traits associated with the extreme body plan and life history of water striders.

**Electronic supplementary material:**

The online version of this article (10.1186/s12864-018-5163-2) contains supplementary material, which is available to authorized users.

## Background

The semi-aquatic bugs (Gerromorpha) are a monophyletic group of predatory heteropteran insects characterized by their ability to live at the water-air interface [1–4]. Over 200 million years ago, the ancestor of the Gerromorpha transitioned from terrestrial habitats to the water surface, leading to a radiation that has generated over 2,000 species classified into eight families [1]. Phylogenetic reconstructions suggest that the ancestral habitat of the Gerromorpha was either humid and terrestrial or marginally aquatic [1, 5, 6]. Water striders subsequently became true water surface dwellers and colonized a diverse array of niches, including streams, lakes, ponds, marshes, and the open ocean [1, 7, 8]. The invasion of these new habitats provided access to resources previously underutilized by insects and made the Gerromorpha the dominant group of insects at water surfaces [1]. This novel specialized life style makes the Gerromorpha an exquisite model system to study how new ecological opportunities can drive adaptation and species diversification [2, 9–11].

**Fig. 1:**
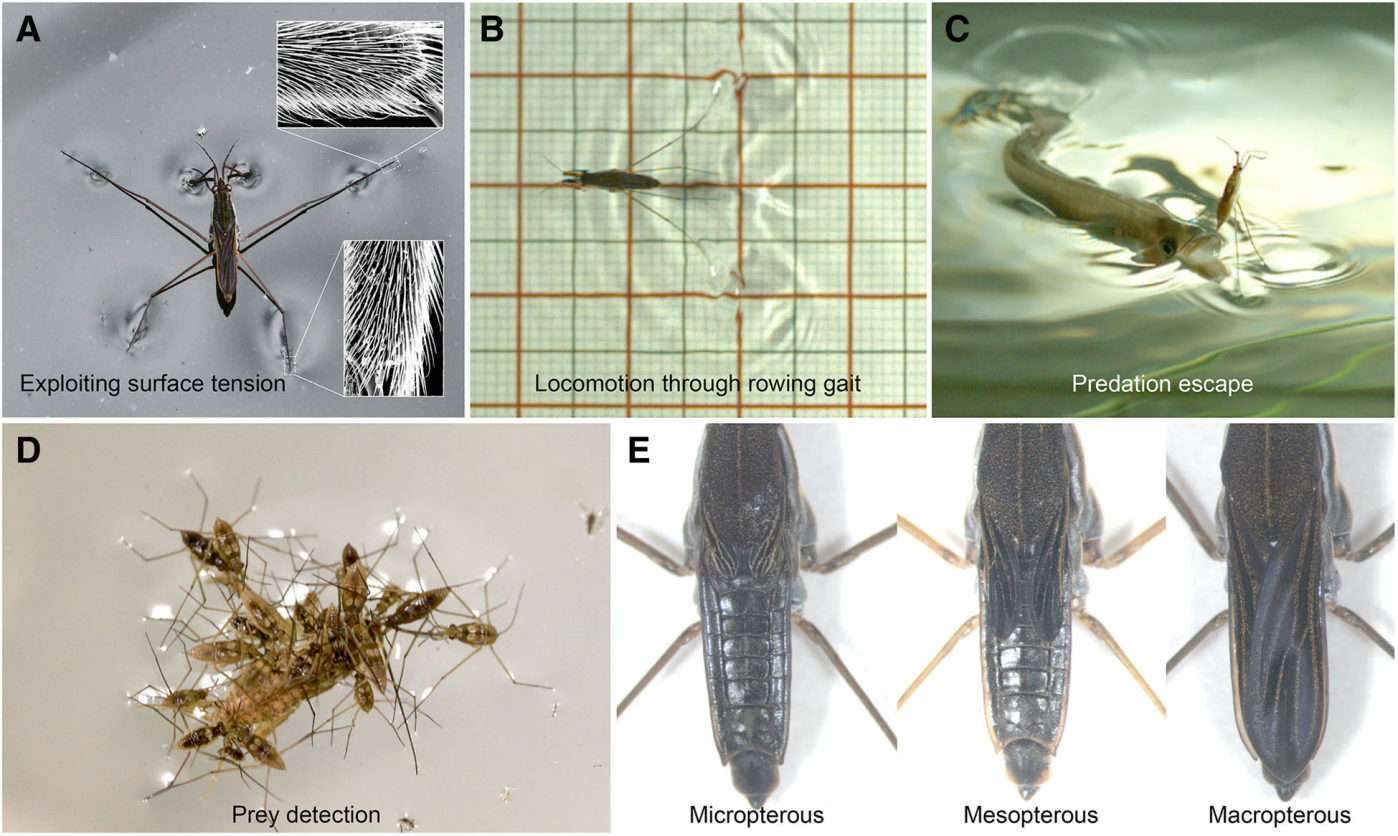
Aspects of the biology of water striders. **a** Adult *Gerris sp* on water and zoom in on the bristles allowing this adaptation using Scanning Electron Microscopy (insets). **b** *G. buenoi* rowing on the water surface, illustrating the adaptive locomotion mode. **c** Water strider jumping using its long legs to escape the strike of a surface hunting fish. **d** Hoarding behavior in water striders consisting of multiples individuals feeding on a cricket trapped by surface tension. **e** Wing polymorphism in *G. buenoi*, here illustrated by three distinct morphs with regard to wing size

This shift in habitat exposed the Gerromorpha to new selective pressures compared to their terrestrial ancestors. The Gerromorpha face two primary challenges unique among insects: how to remain afloat and how to generate efficient thrust on the fluid substrate for locomotion [2, 3, 12]. Due to their specific arrangement and density, the bristles covering the legs of water striders are adapted to keep them afloat by acting as non-wetting structures, which exploit water surface tension by trapping air between the leg and water (Fig. 1a) [2, 3, 12, 13]. Furthermore, locomotion is made possible through evolutionary changes in the morphology and biomechanical adaptions associated with patterns of leg movement (Fig. 1b) [2, 3, 12, 13]. Two distinct modes of locomotion are employed by distinct species: an ancestral mode using a tripod gait with alternating leg movements, and a derived mode using a rowing gait through a simultaneous sculling motion of the pair of middle legs (Fig. 1b) [2, 12]. The rowing mode is characteristic of the Gerridae and some Veliidae and is associated with a derived body plan where the middle legs are the longest (Fig. 1a–b) [2, 12]. The evolutionary trajectory of this group is also thought to have been shaped by the novel predator-prey interactions (Fig. 1c and d) associated with their water surface life history. Following the invasion of water surfaces, other adaptations have emerged, including: (1) the adaption of their visual system to the surface-underwater environment; (2) the evolution of wing polymorphisms associated with dispersal strategies and habitat quality (Fig. 1e) [14], and changes in cuticle composition that optimized water exchange and homeostasis associated with living on water.

While we are starting to uncover the developmental genetic and evolutionary processes underlying the adaptation of water striders to the requirements of water surface locomotion, predator-prey, and sexual interactions [2, 15–19], studies of these mechanisms at the genomic level are hampered by the lack of a representative genome. Here we report the genome of the water strider *G. buenoi*, the first sequenced member of the infraorder Gerromorpha. *G. buenoi* is part of the family Gerridae, and has been previously used as a model to study sexual selection and developmental genetics [15, 20–22]. Moreover, *G. buenoi* can easily breed in laboratory conditions and is closely related to several other *Gerris* species used as models for the study of water-walking hydrodynamics, salinity tolerance, and sexual conflict. With a particular focus on manual annotation and analyses of processes involved in phenotypic adaptations to life on water, our analysis of the *G. buenoi* genome suggests that the genomic basis of water surface invasion might be, at least in part, underpinned by clustered gene family expansions and tandem gene duplications.

## Results and discussion

### General features of the *G. buenoi* genome

**Fig. 2:**
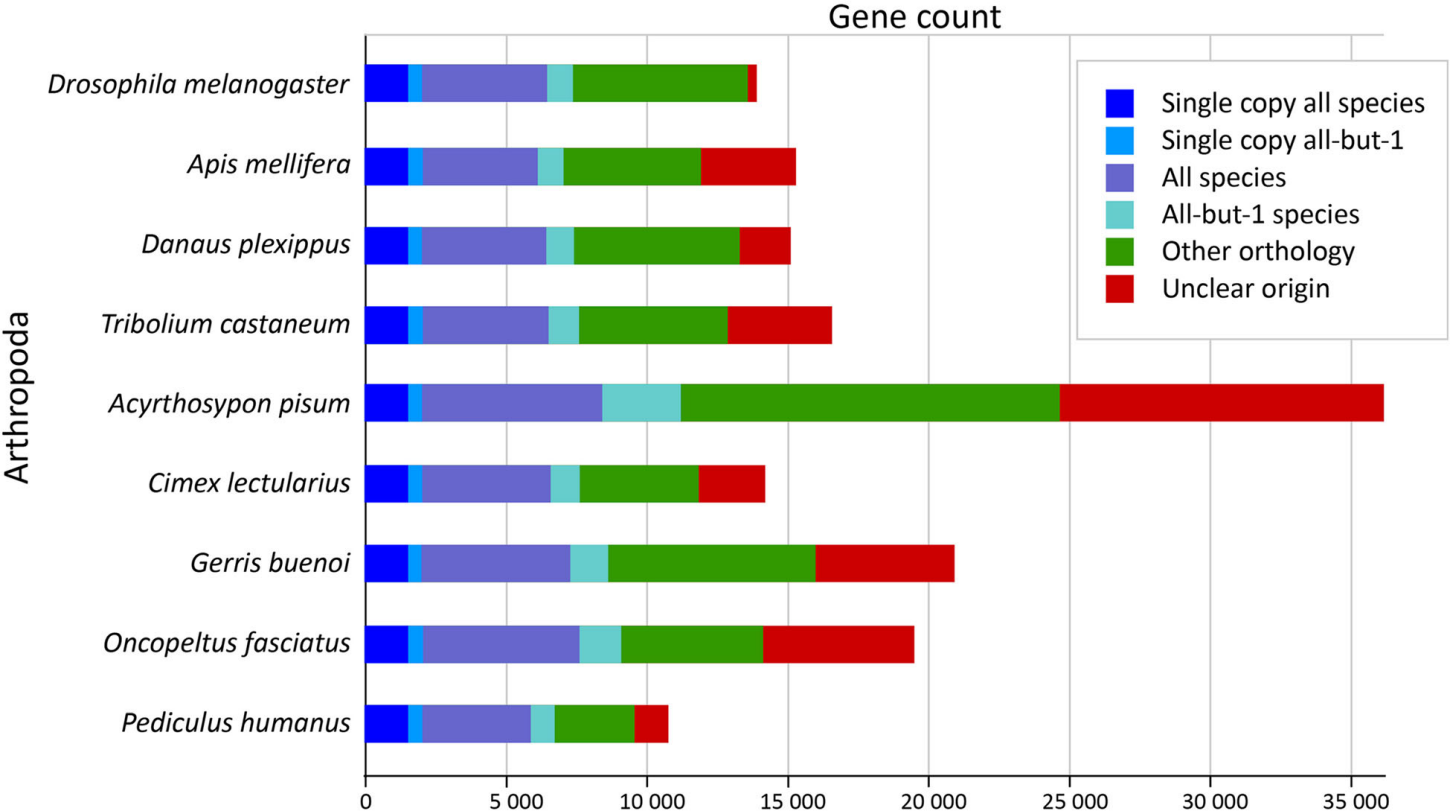
Orthology comparison between *Gerris buenoi* and other arthropod species. Genome proteins were clustered with proteins of other 12 arthropod species based on OrthoDB orthology

The draft assembly of *G. buenoi* genome comprises 1,000,194,699 bp (GC content: 32.46%) in 20,268 scaffolds and 304,909 contigs (N50 length is 344,118 and 3812 bp, respectively). The assembly recovers ~ 87% of the genome size estimated at ~ 1.15 GB based on k-mer analysis. The *G.buenoi* genome is organized into 18 autosomal chromosomes with a XX/X0 sex determination system [23]. The MAKER automatic annotation pipeline predicted 20,949 protein-coding genes, which is greater than the 16,398 isogroups previously annotated in the transcriptome of the closely related species *Limnoporus dissortis* (PRJNA289202) [18, 24], as well as the 14,220 genes in the bed bug *Cimex lectularius* genome [25] and the 19,616 genes in the genome of the milkweed bug *Oncopeltus fasciatus* [26]. The final *G. buenoi* official gene set (OGS) 1.0 includes 1,277 manually annotated genes, including 1,378 mRNAs and 15 pseudogenes, representing development, growth, immunity, cuticle formation as well as olfaction and detoxification pathways genes, amongst others (see Additional file 1). Using OrthoDB [27, 28], we found that ~ 75% of *G. buenoi* genes have at least one orthologue in other arthropod species (Fig. 2). We then used benchmarking sets of universal single-copy orthologs (BUSCOs) [29, 30] to assess the completeness of the assembly. A total of 85.4% of BUSCOs were found complete and 12.3% were fragmented.

In addition to BUSCOs, we used Hox and Iroquois Complex (Iro-C) gene clusters as indicators of draft genome quality and as an opportunity to assess synteny among species. The Hox cluster is conserved across the Bilateria [31], and the Iro-C is found throughout the Insecta [25, 32]. In *G. buenoi*, we were able to find and annotate gene models for all ten Hox genes (Additional file 1: Table S3). While linkage of the highly conserved central class genes *Sex combs reduced*, *fushi tarazu*, and *Antennapedia* occurred in the expected order and with the expected transcriptional orientation, the linked models of *proboscipedia* and *zerknüllt* (*zen*) occur in opposite transcriptional orientations (head-to-head, rather than both 3′ to 5′). Inversion of the divergent *zen* locus is not new in the Insecta [33], but was not observed in the hemipteran *C. lectularius*, in which the complete Hox cluster was fully assembled [25]. Future genomic data will help to determine whether such a microinversion within the Hox cluster is conserved within the hemipteran family Gerridae. Assembly limitations are also evident in our Hox cluster analysis. For example, the complete gene model for *labial* is present but split across scaffolds, while only partial gene models could be created for *Ultrabithorax* and *Abdominal-B*. Furthermore, while there are clear single-copy orthologues of members of the small Iroquois complex, *iroquois* and *mirror*, they are not linked in the current assembly (Additional file 1: Table S3). However, both genes are located near the ends of their scaffolds, and direct concatenation of the scaffolds (5′-Scaffold451–3′, 3′-Scaffold2206-5′) would correctly reconstruct this cluster: (1) with both genes in the 5′-to-3′ transcriptional orientation along the (+) DNA strand, (2) with no predicted intervening genes within the cluster, and (3) with a total cluster size of 308 Kb, which is fairly comparable with that of other recently sequenced hemipterans in which the Iro-C cluster linkage was recovered (391 Kb in *C. lectularius* [25] and 403 Kb in *O. fasciatus* [26]). Lastly, building on the automated BUSCO assessment for presence of expected genes, we examined genes associated with autophagy processes, which are highly conserved among insects, and all required genes are present within the genome (Additional file 2). Therefore, along with the Hox and Iroquois Complex (Iro-C) gene cluster analyses, the presence of a complete set of required autophagy genes suggest good gene representation and supports further analysis.

### Adaptation to water surface locomotion

One of the most important morphological adaptations that enabled water striders to conquer water surfaces is the change in shape, density, and arrangement of the bristles that span the contact surface between their legs and the fluid substrate. These bristles, by trapping air, act as non-wetting structures, forming a cushion between the legs and the water surface (Fig. 1a) [2, 3, 12, 13]. QTL studies in flies uncovered dozens of candidate genes and regions linked to variation in bristle density and morphology [34]. In the *G. buenoi* genome we were able to annotate 90 out of 120 genes known to be involved in bristle development [34, 35] (Additional file 1: Table S4). Among these, we found a single duplication, the gene *Beadex* (*Bx*). A similar duplication found in *C. lectularius* and *H. halys* suggest that the *Bx* duplication may have predated the separation of these lineages and the radiation of Gerromorpha, although a broader phylogenetic sampling is needed to strengthen this conclusion. In *Drosophila*, *Bx* is involved in neural development by controlling the activation of *achaete*-*scute* complex genes [36] and mutants of *Bx* have extra sensory organs [36]. Based on this, it is reasonable to speculate that duplication of *Beadex* might have been exploited by water striders and subsequently linked to changes in bristle pattern and density. This possibility opens up new research avenues to further understand the adaptation of water striders to living on the water surface.

### A new duplication in the Insulin Receptor gene family in the Gerromorpha

**Fig. 3:**
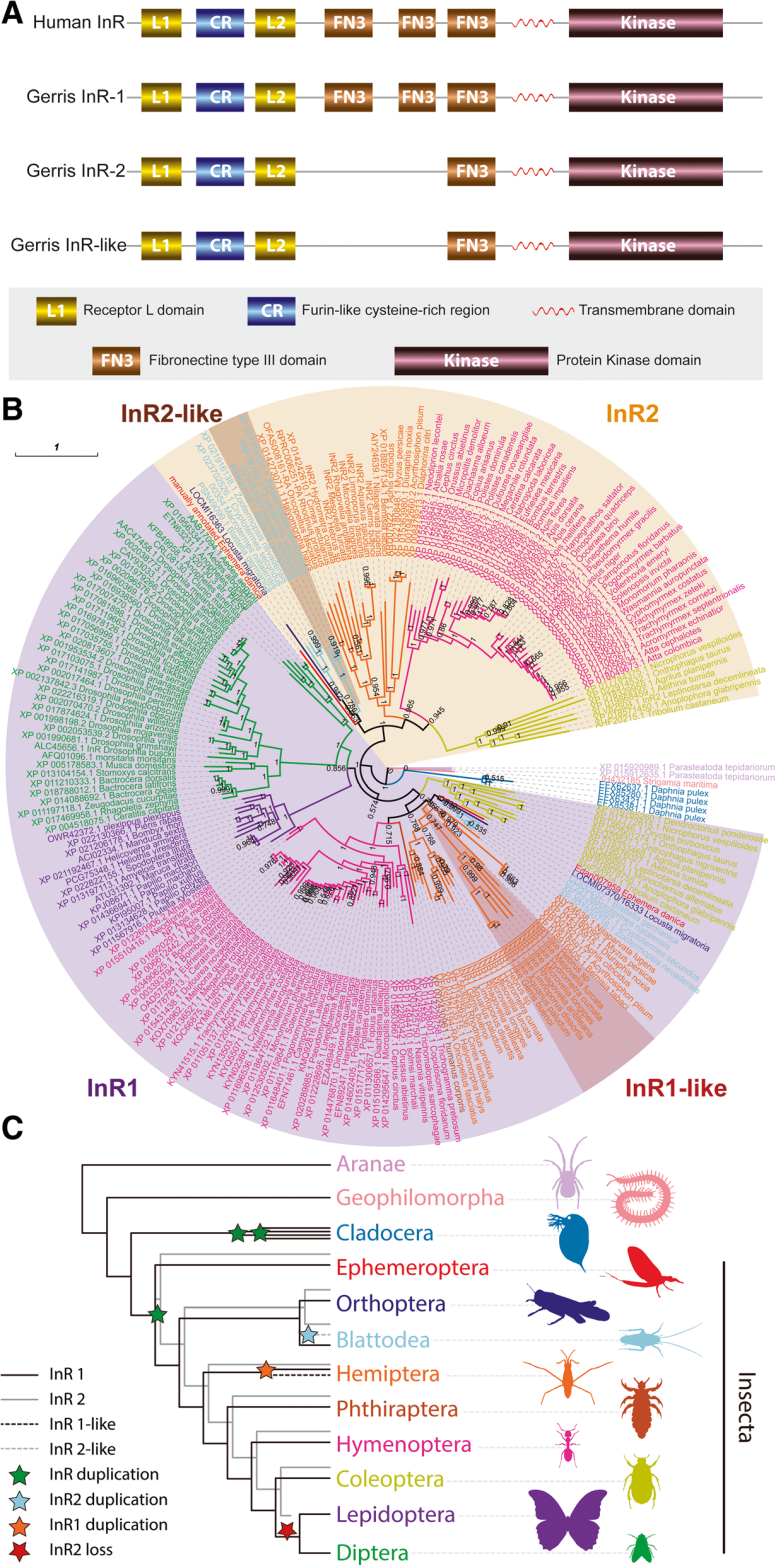
Characterization of the three copies of the Insulin Receptor in *Gerris buenoi*. **a** Protein domain comparison between the three InRs of *G. buenoi* and the Human InR. **b** InR phylogenetic relationship amongst Insecta. Branch support numbers at branches. A non-circular version included in Additional file 1: Figure S1. **c** Simplified Arthropoda phylogeny based on [115] depicting InR duplications and loss events

The insulin signaling pathway coordinates hormonal and nutritional signals in animals [37–39]. This facilitates the complex regulation of several fundamental molecular and cellular processes including transcription, translation, cell stress, autophagy, and physiological states, such as aging and starvation [39–42]. The action of insulin signaling is mediated through the Insulin Receptor (InR), a transmembrane receptor of the tyrosine kinase class [43]. While vertebrates possess one copy of the InR [44], arthropods generally possess either one or two copies, although the highly duplicated *Daphnia pulex* genome [45] contains four copies [46]. Interestingly, the *G. buenoi* genome contains three distinct InR copies. Further sequence examination using in-house transcriptome databases of multiple Gerromorpha species confirmed that this additional copy is common to all of them, indicating that it was present in the common ancestor of the group (Fig. 3). In addition, cloning of the three InR sequences using PCR indicates that these sequences originate from three distinct coding genes that are actively transcribed in this group of insects. Comparative protein sequence analysis revealed that the three InR copies possess all the characteristic domains found in InR in both vertebrates and invertebrates (Fig. 3a). Together, these results validate the presence of three InR copies in Gerromorpha, an exceptional situation amongst Arthropoda.

While this manuscript as under evaluation, an independent study reported the presence of a third InR gene in Blattodea [47]. To determine: (1) the origin of the three InR copies in the *G. buenoi* genome; and (2) whether the third copy in Gerromorpha and Blattodea share a common ancestor, we performed a phylogenetic reconstruction that included the sequences of eight Gerromorpha (three InR copies), four Blattodea (three InR copies), *Daphnia* (four copies) and an additional sample of 126 Arthropoda, all of which possess either one or two InR copies (see Additional files 3 and 4). The four InR duplicates of *Daphnia* were all lineage-specific and together formed a sister group to those found in insects. Within insects, this analysis clustered two InR copies into distinct InR1 and InR2 clusters (Fig. 3b). Furthermore, gerromorphan InR1 and InR2 copies clustered with bed bug and milkweed bug InR1 and InR2, respectively, while the Gerromorpha-restricted copy clustered alone (Fig. 3b; Additional file 1: Figure S1). These data suggest that the new InR copy, which we designated InR1-like, most likely originated from the InR1 gene in the common ancestor of the Gerromorpha. In contrast, the third InR copy in Blattodea clustered with InR2, suggesting an independent origin of novel InR copies in Gerromorpha, which we therefore would suggest be designated InR2-like. A closer examination of the organization of the genomic locus of the InR1-like gene in *G. buenoi* revealed that this copy is intronless. This observation, together with the phylogenetic reconstruction, suggests that InR1-like is a retrocopy of InR1 that may have originated through RNA-based duplication [48]. In addition, our analysis suggests two independent losses of InR2. InR2 is lost among the parasitoid wasps yet retained in other wasps, and InR2 is also lost in the common ancestor of Diptera and Lepidoptera. Taken together, our current phylogenetic reconstruction demonstrates that: (1) InR was duplicated at the base of insects, generating InR1 and InR2; (2) InR1 was subsequently duplicated within the Gerromorpha, while InR2 was duplicated at the common ancestor of Blattodea; (3) InR2 was independently lost in the common ancestor of Lepidoptera and Diptera as well as among the parasitoid wasps, while other wasps have retained it.

In insects, the insulin signaling pathway has been implicated in the developmental regulation of complex nutrient-dependent growth phenotypes such as beetle horns and wing polyphenisms in plant hoppers, as well as morphological caste differentiation in social termites and bees [49–52]. In the particular case of wing polymorphism in *G. buenoi* [1, 14, 52], our analysis found no DNA methylation signature, as previously found in wing polyphenic ants and aphids [53–57], but rather an increased number of histone clusters and a unique duplication of the histone methyltransferase *grappa* (see Additional file 1: Supplementary Data). Taken together, it will be of interest to test the functional significance of the new InR copy in relation to wing polyphenism, as well as more generally how it may be potentially involved in appendage plasticity, either independent of, or alongside, epigenetic processes. Moreover, a comparative functional approach between the novel InR genes in Gerromorpha and Blattodea will shed light on the role independent insulin receptor duplications have played in functional convergence and/or diversification.

### A lineage-specific expansion and possible sensitivity shifts of long wavelength sensitive opsins

**Fig. 4:**
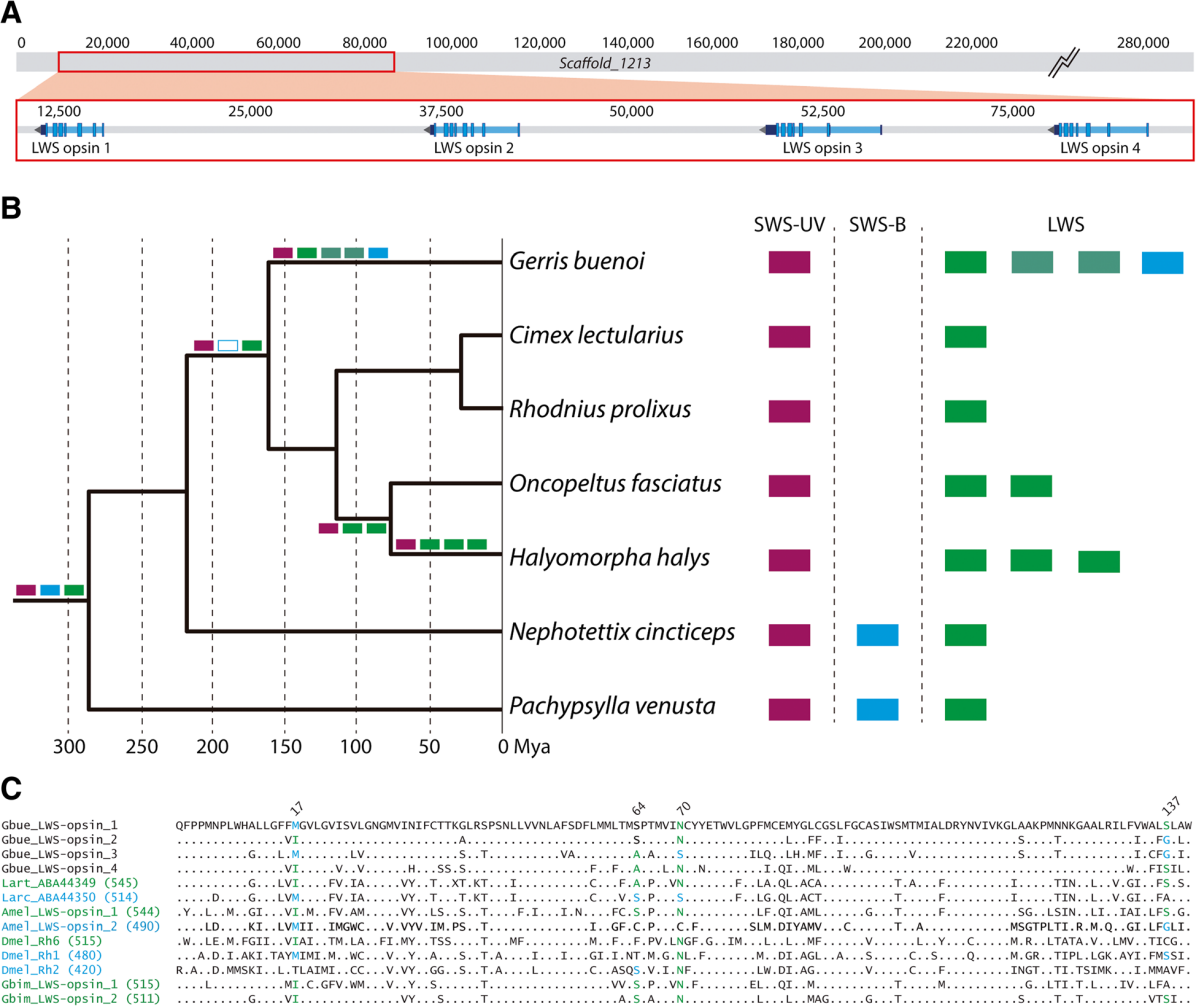
Genomic locus and global analysis of the *Gerris buenoi* opsin gene repertoire. **a** Structure of the scaffold containing the four *G. buenoi* long wavelength (LWS) opsins. **b** Retinal opsin repertoires of key hemipteran species and reconstructed opsin subfamily loss and expansion events along the hemipteran phylogeny. **c** Comparison of amino acid residues at the four tuning sites identified in the LWS opsins of Lepidoptera [68, 69]. Site numbers based on [68]. Numbers in parentheses are experimentally determined sensitivity maxima. Species abbreviations: Amel = *Apis mellifera*, Dmel = *Drosophila melanogaster*, Gbue = *Gerris buenoi*, Gbim = *Gryllus bimaculatus*, Larc = *Limenitis archippus*, Lart = *Limenitis arthemis astyanax*

Visual ecology at the air-water interface and the exceptionally specialized visual system of water striders has drawn considerable interest [58, 59]. Consisting of over 900 ommatidia, the prominent compound eyes of water striders are involved in prey localization, mating partner pursuit, predator evasion and dispersal by flight [60–62]. Realization of the first three tasks is associated with dorsal-ventral differences in the photoreceptor organization of the eye [63, 64], and polarized light-sensitivity [65] (see Additional file 1: Supplementary Data). Each water strider ommatidium contains six outer and two inner Recent work has produced evidence of at least two types of ommatidia, with outer photoreceptors that are sensitive to either green (~ 530 nm) or blue (~ 470–490 nm) wavelengths [66], but the wavelength specificity of the two inner photoreceptors cells is still unknown. At the molecular level, the wavelength specificity of photoreceptor subtypes is mostly determined by the expression of paralogous opsins (light sensitive G-protein coupled receptor proteins), which differ in their wavelength absorption maxima. Interestingly, our genomic analysis of opsin diversity in *G. buenoi* uncovered 8 opsin homologs. Among these, we uncovered three arthropod non-retinal opsins (c-opsin, Arthropsin and Rh7 opsin) (see Additional file 1: Supplementary Data) in addition to five retinal opsins (Fig. 4a; Additional file 1: Figure S2). One of these five retinal opsins was identified as a member of the UV-sensitive opsin subfamily and the other four were identified as tandem, clustered members of the long wavelength sensitive (LWS) opsin subfamily (Fig. 4b).

Surprisingly, both genomic and transcriptomic searches in *G. buenoi* and other water strider species failed to detect sequence evidence of homologs of the otherwise deeply conserved blue-sensitive opsin subfamily (Fig. 4b; Additional file 1: Table S5) [67]. Although the apparent lack of blue opsin in *G. buenoi* was unexpected given the presence of blue sensitive photoreceptors [66], it was consistent with the lack of blue opsin sequence evidence in the available genomes and transcriptomes of other heteropteran species including *Halyomorpha halys*, *Oncopeltus fasciatus*, *Cimex lectularius*, and *Rhodnius prolixus*. Blue opsin, however, is present in other hemipteran clades, including Cicadomorpha (*Nephotettix cincticeps*) and Sternorrhyncha (*Pachypsylla venusta*) (Fig. 4b). Based on the currently available sample of hemipteran species, these data suggest that the blue-sensitive opsin subfamily was lost early in the last common ancestor of the Heteroptera (Fig. 4b and Additional file 1: Table S5). This raises the question of which compensatory events explain the presence of blue sensitive photoreceptors in water striders.

Studies in butterflies and beetles produced evidence of blue sensitivity shifts in both UV- and LWS-opsin homologs following gene duplication [68–70]. In butterflies, molecular evolutionary studies have implicated amino acid residue differences at four protein sequence sites in sensitivity shifts from green to blue: Ile17Met, Ala64Ser, Asn70Ser, and Ser137Ala [68, 69] (Fig. 4c; Additional file 1: Figure S2 and Supplementary Data). Based on sequence information from physiologically characterized LWS opsins in other insect orders and the degree of amino acid residue conservation at these sites in a sample of 114 LWS opsin homologs from 54 species representing 12 insect orders (Additional file 1: Supplementary Data and Additional file 5), we could identify *G. buenoi* LWS opsin 3 as a high confidence candidate for a blue-shifted paralog, followed by *G. buenoi* LWS opsin 1 and 2. Moreover, the *G. buenoi* LWS opsin 4 paralog matches all of the butterfly green-sensitive amino acid residue states, thus favoring this paralog as green-sensitive (Fig. 4). These conclusions are further backed by the fact that water striders lack ocelli, which implies that all four paralogs are expressed in photoreceptors of the compound eye. Overall, it is most likely that the differential expression of the highly diverged *G. buenoi* LWS opsin paralogs accounts for the presence of both blue- and green-sensitive peripheral photoreceptors in water striders. Moreover, given that the outer blue photoreceptors have been specifically implicated in the detection of contrast differences in water striders [66], it is tempting to speculate that the deployment of blue-shifted LWS opsins is a convergent characteristic of a fast-tracking visual system, similar to visual systems in dipteran species that also feature open rhabdomeres, neural superposition, and polarized light-sensitivity.

### Expansion of cuticle gene repertoires

Desiccation resistance is essential to the colonization of terrestrial habitats by arthropods [71]. However, contrary to most insects, the Gerromorpha spend their entire life cycle in contact with water and exhibit poor desiccation resistance [1]. Cuticle proteins and aquaporins are essential for desiccation resistance through regulation of water loss and rehydration [72–75]. Unexpectedly in the *G. buenoi* genome, most members of cuticular and aquaporin protein families are present in similar numbers compared to other hemipterans (Additional file 1: Table S6 and Figure S3; Additional files 6 and 7). We identified 155 putative cuticle proteins belonging to five cuticular families: CPR (identified by Rebers and Riddiford Consensus region), CPAP1 and CPAP3 (Cuticular Proteins of Low-Complexity with Alanine residues), CPF (identified by a conserved region of about 44 amino acids), and TWDL (Tweedle) [76, 77] (Additional file 1: Table S6). Interestingly, almost half of them are arranged in clusters, indicative of local duplication events (Additional file 1: Table S7). Moreover, while most insect species, including other hemipterans, have only three TWDL genes, we found that the TWDL family in *G. buenoi* has been expanded to ten genes (Additional file 1: Figure S4). This expansion of the TWDL family is similar to that observed in some Diptera that possess *Drosophila*-specific and mosquito-specific TWDL expansions [77, 78]. Mutations in the *Drosophila TwdlD* are known to alter body shape [78]. Given the high diversification in body sizes and shapes in association with various aquatic habitats in the Gerromorpha in general [1, 2] and the Gerridae in particular [79, 80], it is possible that the expansion of the TWDL gene family is linked to this diversification. Therefore, a functional analysis of TWDL genes and comparative analysis with other hemipterans will provide important insights into the evolutionary origins and functional significance of TWDL expansion in *G. buenoi*.

### Prey detection in water surface environments

Unlike many closely related species that feed on plant sap or animal blood, *G. buenoi* feeds on various arthropods trapped by surface tension (Fig. 1d), thus making their diet highly variable. Chemoreceptors play a crucial role for prey detection and selection, in addition to vibrational and visual signals. We annotated the three families of chemoreceptors that mediate most of the sensitivity and specificity of chemoperception in insects: odorant receptors (ORs; Additional file 1: Figure S5A and Additional file 8), gustatory receptors (GRs; Additional file 1: Figure S5B and Additional file 8) and ionotropic receptors (IRs; Additional file 1: Figure S5C and Additional file 8) (e.g. [81, 82]). Interestingly, we found an increase in the number of chemosensory genes in *G. buenoi* (Additional file 1: Table S8). First, the OR family is expanded, with a total of 155 OR proteins. This expansion is the result of lineage-specific “blooms” of particular gene subfamilies, including expansions of the 4, 8, 9, 13, 13, 16, 18, and 44 subfamilies (Additional file 1: Figure S5A and Supplementary Data). Second, the GR family is also fairly large (Additional file 1: Figure S5B), but the expansions here are primarily the result of extensive alternative splicing, such that 60 genes encode 135 GR proteins (Additional file 1: Table S8). These GRs include six genes encoding proteins related to the carbon dioxide receptors of flies, three related to sugar receptors, and one related to the fructose receptor (Additional file 1: Figure S5B). The remaining GRs include several highly divergent proteins, as well as four blooms, the largest of which comprises 80 proteins (Additional file 1: Figure S5B and Supplementary Data). By analogy with *D. melanogaster*, most of these proteins are likely to be “bitter” receptors, although some might be involved in perception of cuticular hydrocarbons and other molecules. Finally, the IR family is expanded to 45 proteins. In contrast with the OR/GR families, where the only orthologs across four heteropterans (*Rhodnius prolixus*, *Cimex lectularius*, *Oncopeltus fasciatus* and *Gerris buenoi*) and *Drosophila* are the single OrCo and fructose receptors, the IR family has single orthologs in each species. This is not restricted to only the highly conserved co-receptors (IR8a, 25a, and 76b) but also includes receptors implicated in sensing amino acids, temperature, and humidity (Ir21a, 40a, 68a, and 93a). As is common in other insects the amine-sensing IR41a lineage is expanded to four genes, while the acid-sensing IR75 lineage is highly expanded to 24 genes, and like the other heteropterans there are nine more highly divergent IRs (Additional file 1: Figure S5C and Supplementary Data).

We hypothesize that the high number of ORs may be linked to prey detection mediated by odor molecules at the air-water interface, although functional analysis will be needed to test this. As *G. buenoi* are faced with prey that have fallen on the water surface, and therefore individuals exhibit more of a scavenger strategy as compared to a hunter strategy, this expansion of ORs may enhance their ability to evaluate palatability. As toxic molecules are often perceived as bitter, the GR expansion might provide a complex bitter taste system to detect and even discriminate between molecules of different toxicities [83]. Finally, expansion of the IR family could be linked with prey detection as well as pheromone detection of water-soluble hydrophilic acids and amines, many of which are common chemosensory signals for aquatic species [84, 85].

### Detoxification pathways

Water striders can be exposed to various toxic compounds found in the water, including those generated by pesticides, insecticides, and from other human activities as well as those found in their prey. Insect cytochrome P450 (CYP) proteins play a role in metabolic detoxification of xenobiotics including insecticides [86, 87]. They are also known to be responsible for the synthesis and degradation of endogenous molecules, such as ecdysteroids [88] and juvenile hormone [89]. The insect CYPs, one of the oldest and largest gene families in insects, underwent a high degree of diversification after multiple instances of gene duplication, which may have enhanced a species’ adaptive range [90]. In addition to CYP proteins we have also surveyed the presence of UDP-glycosyltransferase (UGT) genes in *G. buenoi*. UGTs are important for xenobiotic detoxification and the regulation of endobiotics in insects [91]. UGTs catalyse the conjugation of a range of small hydrophobic compounds to produce water-soluble glycosides that can be easily excreted in a number of insects [92, 93].

**Fig. 5:**
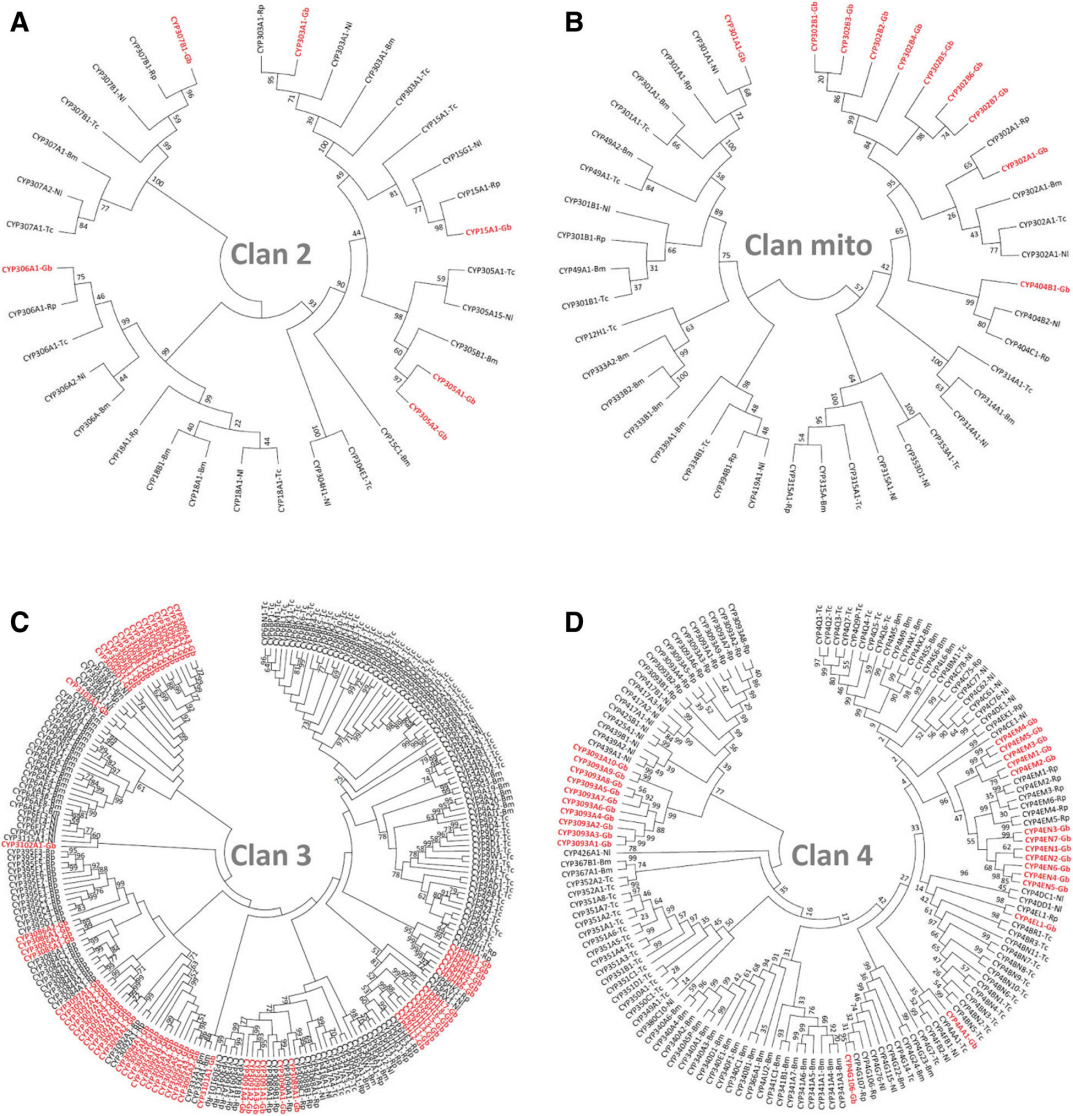
Phylogenetic analysis of four different Clans of the cytochrome P450s of *Gerris buenoi* with other insect species. **a** Clan 2, **b** Clan mitochondria, **c** Clan 3, and **d** Clan 4. The *G. buenoi* sequences are indicated in red and bold

We annotated and analyzed a total of 103 CYP genes (Additional file 1: Table S9, Additional files 3 and 9) and 28 putative UGT genes, including several partial sequences due to genomic gaps (Additional file 1: Table S10). Ten more CYP fragments were found, but they were not included in this analysis due to their short lengths (<250 aa). This is the largest number of CYP genes among the hemipteran and other species’ genomes in which CYPomes were annotated: *O. fasciatus* (58 CYPs), *R. prolixus* (88 CYPs) and *N. lugens* (68 CYPs) [26, 94, 95], *D. melanogaster* (85), *A. mellifera* (45), and *B. mori* (86) (Additional file 1: Table S9). Indeed, the *G. buenoi* CYP protein family size is only exceeded by that of *T. castaneum* (131 proteins). CYP genes fall into one of the four distinct subfamilies: Clan 2 (6 genes), Clan mito (62 genes), Clan 3 (25 genes) and Clan 4 (10 genes) (Fig. 5; see Additional file 1: Supplementary Data). Similarly, the number of UGT genes is also higher than that of *O. fasciatus* (1) [26], *C. lectularius* (7) [25], *D. melanogaster* (11), *A. mellifera* (6) and *B. mori* (14) [96], and identical to *T. castaneum* (28) [96].

Interestingly, both CYP and UGT gene family expansions seem to be closely linked with tandem duplication events. In the particular case of *G. buenoi* CYPs, the Clan 2 and Clan mito have undergone relatively little gene expansion (Fig. 5a and b). However, an exceptional gene expansion is observed in the mitochondrial Clan of the *G. buenoi* CYPs, where seven CYP302Bs form a lineage-specific cluster (Fig. 5b). The Clan 3 and Clan 4 are highly expanded in insects such as *T. castaneum*, *B. mori*, *R. prolixus*, and *N. lugens*, as well as in *G. buenoi*, of which 45% (28/62 CYP genes) might have been generated by tandem gene duplications (Fig. 5c and d). On the other hand, ten UGT genes are clustered on Scaffold1549, suggesting gene duplication events may have produced this large gene cluster (Additional file 1: Figure S6). In addition, multiple UGT genes are linked within Scaffold1323, Scaffold3228, and Scaffold2126. A consensus Maximum-likelihood tree (Additional file 1: Figure S7) based on the conserved C-terminal half of the deduced amino acid sequences from *G. buenoi* UGTs supports the conclusion that genes clustered within the genome derive from recent tandem duplications.

Overall, our phylogenetic analysis revealed the conservation of CYPs and UGTs across insects, and the possibility for expansions via lineage-specific gene duplication. We hypothesize that this expansion may have been important in order to diversify the xenobiotic detoxification range and the regulation of endobiotics during the terrestrial-to-water surface transition.

## Conclusions

The sequencing of the *G. buenoi* genome provides a unique opportunity to understand the molecular mechanisms underlying initial adaptations to water surface life and the subsequent diversification that followed. In particular, gene duplication is known to drive the evolution of adaptations and evolutionary innovations in a variety of lineages including water striders [80, 97–99]. The *G. buenoi* genome revealed a number of clustered duplications in genes that can be linked to processes associated with the specialized life style of water striders. Some are shared with closely related Hemiptera, for example, the duplicated factor *Beadex* is an activator of the *Achaete*/*Scute* complex known to play an important role in bristle development. Other genes and gene family duplications are particularly rare, such as that found with the insulin receptors, which are known in other insects to be involved in a range of rocesses including wing development, growth, as well as a number of life history traits including reproduction [49, 52, 100]. The functional significance of the duplication of the histone methyltransferase *grappa* and histone cluster duplications remains unknown, yet opens up new avenues for investigation into the relationship between epigenetics and phenotypic plasticity. Expansions in the cuticle protein families involved in desiccation resistance or genes repertoires involved in xenobiotic detoxification and endobiotic regulation pathways may have played an important role during water surface specialization [78, 101]. Furthermore, the expansion of the opsin gene family and possible light sensitivity shifts are also likely associated with particularities of polarized light detection within the aquatic environment in which *G. buenoi* specializes. The impact of these duplications on the ability of water striders to function efficiently in water surface habitats remains to be experimentally tested. *G. buenoi*, which is now emerging as a tractable experimental model, offers a range of experimental tools to test these hypotheses. More generally, the *G. buenoi* genome provides a good opportunity to further understand the molecular and developmental genetic basis underlying adaptive radiations and diversification upon the conquest of new ecological habitats.

## Methods

### Animal collection and rearing

Adult *G. buenoi* individuals were collected from a pond in Toronto, Ontario, Canada. *G. buenoi* were kept in aquaria at 25 °C with a 14-h light/10-h dark cycle and fed on live crickets. Pieces of floating Styrofoam were regularly supplied to female water striders to lay eggs. The colony was inbred following a sib-sib mating protocol for six generations prior to DNA/RNA extraction.

### DNA and total RNA extraction

Genomic DNA was isolated from adults using Qiagen Genome Tip 20 (Qiagen Inc., Valencia CA). The 180 and 500 bp paired-end libraries as well as the 3 kb mate-pair library were made from eight adult males. The 8 kb mate-pair library was made from eight adult females. Total RNA was isolated from 39 embryos, three first instar nymphs, one second instar nymph, one third instar nymph, one fourth instar nymph, one fifth instar nymph, one adult male and one adult female. RNA was extracted using a Trizol protocol (Invitrogen).

### Genome sequencing and assembly

Genomic DNA was sequenced using HiSeq2500 Illumina technology. 180 and 500 bp paired-end and 3 and 10 kb mate-pair libraries were constructed and 100 bp reads were sequenced. Estimated coverage was 28.6×, 7.3×, 21×, 17×, 72.9× respectively for each library. Sequenced reads were assembled in draft assembly using ALLPATHS-LG [102] and automatically annotated using custom MAKER2 annotation pipeline [103]. (More details can be found in Additional file 1: Supplementary Data). Expected genome size was calculated counting from Kmer based methods and using Jellyfish 2.2.3 and perl scripts from [104].

### Community curation of the *G. buenoi* genome

International groups within the i5k initiative have collaborated on manual curation of *G. buenoi* automatic annotation. These curators selected genes or gene families based on their own research interests and manually curated MAKER-predicted gene set GBUE_v0.5.3 at the i5k Workspace@NAL [105] resulting in the non-redundant Official Gene Set OGSv1.0 [106].

### Assessing genome assembly and annotation completeness with BUSCOs

Genome assembly completeness was assessed using BUSCO [29]. The Arthropoda gene set of 2675 single copy genes was used to test *G. buenoi* predicted genes.

### Orthology analyses

OrthoDB8 (http://orthodb.org/) was used to find orthologues of *G. buenoi* (OGS 1.0) on 76 arthropod species. Proteins on each species were categorised using custom Perl scripts according to the number of hits on other eight arthropod species: *Drosophila melanogaster*, *Danaus plexippus*, *Tribolium castaneum*, *Apis mellifera*, *Acyrthosiphon pisum*, *Cimex lectularius*, *Pediculus humanus* and *Daphnia pulex*.

### Insulin receptors phylogeny

Sequences were retrieved from ‘nr’ database by sequence similarity using BLASTp with search restricted to Insecta (taxid:50557). Each *G. buenoi* InR sequence was individually blasted and best 250 hits were recovered. A total of 304 unique id sequences were retrieved. Additionally, we recovered the genes annotated by Kremer et al. [47] as well as *Caenorhabditis elegans* insulin receptor homolog AAC47715.1 as outgroup. We performed a preliminary analysis aligning the sequences with Clustal Omega [107–109] and building a simple phylogeny using MrBayes [110] (one chain, 100,000 generations). Based on that preliminary phylogeny, we selected a single isoform for each InR gene (Additional file 4). Final InR phylogeny tree was estimated aligning the sequences with MAFFT [111] using E-INS-i iterative method and using MrBayes (four chains, for 1,000,000 generations). Final phylogeny include InR sequences from (copy number in parenthesis):

*Acromyrmex echinatior* (2), *Acyrthosiphon pisum* (2), *Aedes aegypti* (1), *Aedes albopictus* (1), *Aethina tumida* (2), *Agrilus planipennis* (2), *Amyelois transitella* (1), *Anopheles darlingi* (1), *Anopheles gambiae* (1), *Anopheles sinensis* (1), *Anoplophora glabripennis* (2), *Aphis citricidus* (2), *Apis cerana* (2), *Apis dorsata* (2), *Apis florea* (1), *Apis mellifera* (2), *Aquarius paludum* (3), *Athalia rosae* (2), *Atta cephalotes* (2), *Atta colombica* (2), *Bactrocera dorsalis* (1), *Bactrocera latifrons* (1), *Bactrocera oleae* (1), *Bemisia tabaci* (2), *Blattella germanica* (3), *Bombus impatiens* (2), *Bombus terrestris* (2), *Bombyx mori* (1), *Caenorhabditis elegans* (1), *Camponotus floridanus* (2), *Cephus cinctus* (2), *Ceratina calcarata* (2), *Ceratitis capitata* (1), *Ceratosolen solmsi marchali* (1), *Cimex lectularius* (2), *Clunio marinus* (1), *Copidosoma floridanum* (1), *Cryptotermes secundus* (3), *Cyphomyrmex costatus* (2), *Danaus plexippus* (1), *Daphnia pulex* (4), *Dendroctonus ponderosae* (1), *Diachasma alloeum* (2), *Diaphorina citri* (1), *Dinoponera quadriceps* (2), *Diuraphis noxia* (2), *Drosophila ananassae* (1), *Drosophila arizonae* (1), *Drosophila biarmipes* (1), *Drosophila bipectinata* (1), *Drosophila busckii* (1), *Drosophila elegans* (1), *Drosophila erecta* (1), *Drosophila eugracilis* (1), *Drosophila ficusphila* (1), *Drosophila grimshawi* (1), *Drosophila kikkawai* (1), *Drosophila melanogaster* (1), *Drosophila miranda* (1), *Drosophila mojavensis* (1), *Drosophila obscura* (1), *Drosophila persimilis* (1), *Drosophila pseudoobscura* (1), *Drosophila rhopaloa* (1), *Drosophila sechellia* (1), *Drosophila serrata* (1), *Drosophila simulans* (1), *Drosophila suzukii* (1), *Drosophila takahashii* (1), *Drosophila virilis* (1), *Drosophila willistoni* (1), *Drosophila yakuba* (1), *Dufourea novaeangliae* (2), *Ephemera danica* (2), *Eufriesea mexicana* (2), *Fopius arisanus* (2), *Gerris buenoi* (3), *Glossina morsitans morsitans* (1), *Habropoda laboriosa* (2), *Halyomorpha halys* (2), *Harpegnathos saltator* (2), *Hebrus sp* (3), *Helicoverpa armigera* (1), *Heliothis virescens* (1), *Hydrometra cumata* (3), *Lasius niger* (2), *Leptinotarsa decemlineata* (2), *Limnoporus dissortis* (3), *Linepithema humile* (2), *Locusta migratoria* (2), *Macrotermes natalensis* (3), *Manduca sexta* (1), *Maruca vitrata* (1), *Megachile rotundata* (2), *Melipona quadrifasciata* (1), *Mesovelia furcata* (3), *Microplitis demolitor* (2), *Microvelia longipes* (3), *Monochamus alternatus* (1), *Monomorium pharaonis* (2), *Musca domestica* (1), *Myzus persicae* (2), *Nasonia vitripennis* (1), *Neodiprion lecontei* (2), *Nicrophorus vespilloides* (2), *Nilaparvata lugens* (2), *Oncopeltus fasciatus* (2), *Onthophagus nigriventris* (1), *Onthophagus taurus* (2), *Ooceraea biroi* (2), *Orussus abietinus* (2), *Oryctes borbonicus* (1), *Papilio machaon* (2), *Papilio polytes* (1), *Papilio xuthus* (1), *Parasteatoda tepidariorum* (2), *Pediculus humanus corporis* (1), *Pieris rapae* (1), *Plutella xylostella* (1), *Pogonomyrmex barbatus* (2), *Polistes canadensis* (2), *Polistes dominula* (2), *Pseudomyrmex gracilis* (2), *Rhagoletis zephyria* (1), *Rhagovelia antilleana* (3), *Rhodnius prolixus* (2), *Solenopsis invicta* (2), *Spodoptera litura* (1), *Stomoxys calcitrans* (1), *Strigamia maritima* (1), *Trachymyrmex cornetzi* (2), *Trachymyrmex septentrionalis* (2), *Trachymyrmex zeteki* (2), *Tribolium castaneum* (2), *Trichogramma pretiosum* (1), *Trichomalopsis sarcophagae* (1), *Vollenhovia emeryi* (2), *Wasmannia auropunctata* (2), *Zeugodacus cucurbitae* (1), and *Zootermopsis nevadensis* (3).

### Cytochrome P450 proteins phylogeny

CYPs phylogenetic analysis was performed using Maximum-Likelihood method and the trees were generated by MEGA 6. The phylogenetic trees were generated by MEGA 6 with Maximum-Likelihood method using the amino acid sequences from *Gerris buenoi* (Gb), *Rhodnius prolixus* (Rp), *Nilaparvata lugens* (Nl), *Bombyx mori* (Bm) *and Tribolium castaneum* (Tc). All nodes have significant bootstrap support based on 1000 replicates.

## Additional files

Additional file 1: Supplementary Online Information. Additional files 10 and 11. (ZIP 18100 kb)

Additional file 2: List of annotated autophagy genes in *Gerris buenoi*. (XLSX 12 kb)

Additional file 3: Accession number and gene name of 225 arthropod genes used to reconstruct Insulin receptors phylogeny (highlighted). (XLSX 91 kb)

Additional file 4:  Nucleotide sequences of Insulin receptors genes annotated in Gerromorpha (*Gerris buenoi*, *Aquarius paludum*, *Limnoporus dissortis*, *Rhagovelia antilleana*, *Microvelia longipes*, *Mesovelia furcata*, *Hydrometra cumata* and *Hebrus sp*). (DOCX 40kb)

Additional file 5: SNPs summary at position 17, 64, 70 and 137 of 114 LWS opsin homologs from 54 species representing 12 insect orders. (XLSX 26 kb)

Additional file 6: List of annotated aquaporin genes in *Gerris buenoi* including gene names and closest identity. (XLSX 58 kb)

Additional file 7: List of annotated aquaporin genes in *Gerris buenoi* including gene names and closest identity. (XLSX 15 kb)

Additional file 8: Protein sequences of annotated chemoreceptors in *Gerris buenoi* : 155 Or, 135 Gr and 45 IR genes. (DOCX 83 kb)

Additional file 9: List of cytochrome P450 (CYP) genes annotated from the *Gerris buenoi* genome. (XLSX 14 kb)

Additional file 10: *Gerris buenoi* serosins nucleotide and protein sequences. (DOCX 12 kb)

Additional file 11: List of antioxidant enzyme genes identified in the *Gerris buenoi* genome. (XLSX 13 kb)
