# Supplementary material for: The genome of the water strider Gerris buenoi reveals expansions of gene repertoires associated with adaptations to life on the water
Source: BMC Genomics. 2018 Nov 21;19:832. doi: 10.1186/s12864-018-5163-2 (PMC6249893; doi:10.1186/s12864-018-5163-2)
Supplement: Supplementary file 1 — Supplementary Online Information. Additional files 10 and 11. (ZIP 18100 kb) [file 12864_2018_5163_MOESM1_ESM.zip › Armisen_et_al_SOM_R4.docx]

**The genome of the water strider *Gerris buenoi* reveals expansions of gene repertoires associated with adaptations to life on the water**

[**Supplementary Data** 3](#_Toc525573979)

[Immune genes 3](#_Toc525573980)

[Early Developmental Genes 4](#_Toc525573981)

[Nuclear receptors and bHLH-PAS proteins 5](#_Toc525573982)

[Insulin/TOR signalling pathways 6](#_Toc525573983)

[Wnt Signaling Pathway 7](#_Toc525573984)

[Cysteine peptidases from the papain C1 family 10](#_Toc525573985)

[Visual genes 11](#_Toc525573986)

[Chemoreceptor gene families 16](#_Toc525573987)

[Detoxification pathways 20](#_Toc525573988)

[Wing development and polyphenism 24](#_Toc525573989)

[DNA methylatransferases 26](#_Toc525573990)

[Histone genes and histone modification machinery 27](#_Toc525573991)

[Antioxidant Proteins 29](#_Toc525573992)

[**Supplementary Methods** 30](#_Toc525573993)

[Genome sequencing and assembly 30](#_Toc525573994)

[Automated Gene Annotation Using a Maker 2.0 Pipeline Tuned for Arthropods 32](#_Toc525573995)

[Community annotation and Official Gene Set generation 33](#_Toc525573996)

[Bristle genes 34](#_Toc525573997)

[Cuticular proteins 34](#_Toc525573998)

[Prey detection and selection on water environments 34](#_Toc525573999)

[Wing polyphenism 35](#_Toc525574000)

[Wnt Signaling Pathway 36](#_Toc525574001)

[Early Developmental Genes 37](#_Toc525574002)

[Antioxidant genes 38](#_Toc525574003)

[**Supplementary Figure legends** 40](#_Toc525574004)

[Figure S1 40](#_Toc525574005)

[Figure S2 40](#_Toc525574006)

[Figure S3 40](#_Toc525574007)

[Figure S4 41](#_Toc525574008)

[Figure **S**5 41](#_Toc525574009)

[Figure S6 42](#_Toc525574010)

[Figure S7 42](#_Toc525574011)

[Figure S8 42](#_Toc525574012)

[Figure S9 42](#_Toc525574013)

[Figure S10 43](#_Toc525574014)

[Figure S11 43](#_Toc525574015)

[Table S1 44](#_Toc525574016)

[Table S2 44](#_Toc525574017)

[Table S3 47](#_Toc525574018)

[Table S4 51](#_Toc525574019)

[Table S5 52](#_Toc525574020)

[Table S6 53](#_Toc525574021)

[Table S7 54](#_Toc525574022)

[Table S8 55](#_Toc525574023)

[Table S10 57](#_Toc525574024)

[Table S11 60](#_Toc525574025)

[Table S12 62](#_Toc525574026)

[Table S13 65](#_Toc525574027)

[Table S14 67](#_Toc525574028)

[Table S15 74](#_Toc525574029)

[Table S16 75](#_Toc525574030)

[Table S17 76](#_Toc525574031)

[Table S18 78](#_Toc525574032)

[**References** 79](#_Toc525574033)

# **Supplementary Data**

### Immune genes

While mammals have both innate and adaptive immune response, only innate immune response has been described in arthropods [1]. In particular, the Toll and IMD (Immunodeficiency) pathways are the two major regulators of the immune response known in arthropods [2-4] which act by regulating the expression of other effector molecules such as antimicrobial peptides (AMPs).

In the *Gerris buenoi* genome we could annotate more than 60 immune genes, including orthologs of all components of the Toll signalling pathway, which is activated mainly by Gram-positive bacteria and fungi [5, 6]. However, whereas the Toll1-4 receptors were only represented by a single ortholog called Toll1, six Toll9 paralogs were found which raises important questions about a possible adaptation to gram-positive bacteria present in the water. On the other hand, IMD pathway responds mainly to Gram-negative bacteria infection [5, 6] but many of its genes, including IMD, dFADD, Dredd, and Relish could not be found in the first sequenced hemipteran, *Acyrthosiphon pisum* [7, 8]. Further sequencing of other hemipterans extended this absence to the kissing bug *Rodnius prolixus* and the bed bug *Cimex lectularius*, as well as the pest species *Diaphorina citri*, *Pachypsylla venusta* and *Halyomorpha halys*. However, among the 60 immune genes annotated in the genome of *Gerris buenoi*, we could identify a homolog of IMD, a unique feature amongst sequenced Hemiptera species only shared with recently sequenced true bug *Oncopeltus fasciatus* (Figure S8) [9]. However, like in *Oncopeltus fasciatus*, the important IMD pathway components dFADD and Kenny seem to be missing in *Gerris buenoi*. Further research is required to elucidate how the IMD pathway functions in water striders and why IMD has been conserved in *Gerris* while it has been lost in other hemipterans.

Despite the lack of shared components between Toll and IMD, both pathways can regulate immune response through regulation of antimicrobial peptides (AMPs). Antimicrobial peptide (AMPs) families prevent the invasion of potential pathogens playing a fundamental role on innate immunity [10]. However, AMP families differ greatly among groups of insects [11] and only two defensin-like, one lysozyme and 6 of the Hemiptera-specific Serosins (unpublished) could be identified (Additional file 10 : File S9). We failed to identify any attacins, hemiptericins or thaumatins in the *Gerris buenoi* genome. These results suggest that following Gerromorpha invasion of water environment they have been faced with a myriad of new potential pathogens, which may have accelerated Gerromorpha's AMPs divergence.

Finally, we could annotate an ortholog of the innate immune response gene gamma-interferon-inducible thiol reductase (*gilt*) in *Gerris buenoi* genome. Despite only innate immune response has been classically described in arthropods, recent studies on *Drosophila melanogaster* have shown that *gilt* ortholog gene has a role on adaptive immune response in flies [1]. However, the exact mechanism of *gilt* function in immune response remains unknown. Moreover, in water striders including *Gerris buenoi*, although no immune role of *gilt* has been tested yet, knockdown analyses using RNA interference have shown an important new role in leg growth and adaptation [12]. These findings raise interesting questions about the functional divergence of arthropod immune system.

### Early Developmental Genes

One of the main reasons for choosing to sequence the *Gerris buenoi* genome was due to its emerging status as a developmental model system [13]. Therefore, it was of particular interest to analyze its developmental gene content. In total 24 genes that are known, in other insects, to be involved in developmental processes were manually annotated (Table S11). These include both genes encoding transcription factors and members of signaling pathways. These genes are identified and named as distinct development genes by the nomenclature from *Drosophila melanogaster* (Table S12). *Gerris buenoi* has evidence of a canonical insect developmental pathway and can be expected to contain all components required to establish a normal anterior/posterior axis pattern. Compared to the later acting genes, the early developmental genes identified in *Gerris buenoi* show greater divergence from those found in *Drosophila melanogaster* and *Tribolium castaneum* (Table S13), consistent with observations between *Drosophila* species [14]. Developmental genes previously identified in *Limnoporus dissortis* (e.g. *decapentaplegic*) were also identified in the *Gerris buenoi* genome [15] confirming the presence of canonical insect developmental toolkit in this species. No duplication in the early development genes was observed. Early patterning genes appear conserved form what is known in other insects. As expected, there is no *bicoid* orthologue. Other genes known in *Drosophila* but not found in other insects, such as swallow are also not found in *Gerris*, such is the case of *caudal*. However, we suspect due to the identification of tailless that the absence of caudal from the genome is due to incomplete coverage of the sequencing effort, rather than an actual absence of the gene in the genome. We identified gene models for the terminal patterning genes *torso*, and *torso-like* in *Gerris buenoi*. Although models homologous to PTTH were identified they were not well supported. However, is it more than likely that *Gerris buenoi* possess a PTTH orthologue given that PTTH orthologues are found in other hemipterans. As with other Hemiptera, we could not find a model for *trunk*.

### Nuclear receptors and bHLH-PAS proteins

We have annotated the genome of *Gerris buenoi* for all the genes of two families of ligand-dependent transcription factors: nuclear receptors and bHLH-PAS proteins. These regulators share many characteristics, such as response to small lipophilic ligands that can act either as signalling molecules or as xenobiotics and heterodimerisation factors with other members of their family. Numerous cross-talk interactions are known between nuclear receptors and bHLH-PAS proteins.

All but one of the 21 nuclear receptor genes expected for an insect were found in the genome of *Gerris buenoi*. The missing gene E78 is also absent in *Pediculus humanus* [16] but is present in the genome of *Acyrthosiphon pisum* [17, 18]. We found 3 NR0 genes (knirps-related, eagle), as in *Pediculus humanus* and *Apis mellifera* [19]. Based on the work of [20], we could also identify all the isoforms of ECR and NR2E6 genes.

The genome of *Gerris buenoi* contains at least 10 genes of the bHLH-PAS family. The gene *tango* (*tgo*) was not found, whereas it is present in the genome of the *Acyrthosiphon pisum* [21]. This absence is surprising, since *tgo* is the homolog of ARNT, which is the heterodimeric partner of several members of this family in mammals. Since the gene called «*germ cell-expressed*» (*gce*) in *Drosophila* is known to be a diptera specific duplication of *Methoprene-tolerant* (*Met*), its absence in the genome of *Gerris buenoi* was expected. The gene *single-minded* (*sim*) is duplicated, as in *Tribolium castaneum* [22].

In conclusion, we found a strong conservation of the number and identity of nuclear receptors and bHLH-PAS proteins with other insects.

### Insulin/TOR signalling pathways

The Insulin and TOR pathways function together as an integrated metabolic signalling pathway that is known to coordinate hormonal and nutritional signals in developing animals [23-25]. This facilitates the complex regulation of several fundamental molecular and cellular processes including transcription [26, 27], translation, cell stress, autophagy, and physiological states, including aging, starvation, hormonal regulation, as well as both organism-wide and tissue-specific growth [25-30]. In insects, these pathways have been implicated in the developmental regulation of complex nutrient-dependent phenotypes ranging from beetle horns to the social castes of termites and bees [31-33]. For example, in beetles, the insulin receptor is known to be a critical regulator of appendage growth and it has been proposed that downstream transcription factors of the pathway (Foxo), can mediate organ-specific sizing and growth [34, 35]. Taken together, the interplay between these two pathways may play an integral role in the growth and sizing of the different legs, and perhaps, even sexually dimorphic sized appendages found across the morphologically diverse array of water strider species. For this reason, we searched for and annotated various key players of this pathway. We found that *Gerris buenoi* possesses all components of this pathway including the *forkhead box protein O* (*foxo*), *insulin receptor 1* (*InR1*), *insulin receptor 2*(*InR2*), the *insulin receptor substrate Chico*, the negative insulin pathway regulator *Phosphatase and Tensine homologue* (*Pten*), *Rheb/Ras homolog enriched in brain* (*Rheb*), the *S6 kinase* (*S6k*), *Target of Rapamycin* (*Tor*), the binding protein of the translation initiation factor eI4E (*4E-BP/Thor*), *Tuberous sclerosis complex 1* and *2* (*Tsc1 & Tsc2/gigas*), the phosphoinositide-3-OH-kinase-dependent serine/threonine protein kinase *Akt1/Pkb*, the amino acid transporter *Slimfast* (*slif*) and two Phosphoinositide 3-kinases (*Pi3K92E* & *Pi3K21B*). In addition to this, *Gerris buenoi* appears to have an additional, third, insulin receptor of unknown function and no known ortholog in insects. Therefore, the water strider *Gerris buenoi* possesses the entire Insulin/TOR toolkit, which would be a potential target for future research into nutrient-dependent differential body-plan growth and evolution in water striders.

### Wnt Signaling Pathway

The Wnt pathway is a signal transduction pathway with fundamental regulatory roles in embryonic development in all metazoans. The emergence of several gene families of both Wnt ligands and Frizzled receptors allowed the evolution of complex combinatorial interactions with multiple layers of regulation [36]. Wnt signalling affects cell migration and segment polarity as well as segment patterning in most arthropods [37]. Surveying and comparing the gene repertoire of conserved gene families within and between taxonomic groups is the first step towards understanding their function during development and evolution.

Here we curated gene models for the main components of the Wnt signalling pathway and confirmed their orthology by phylogenetic analysis. We found 6 Wnt ligand subfamilies, three Frizzled transmembrane receptor subfamilies, the co-receptor *arrow*, and the downstream components *armadillo*/*beta*-*catenin*, *dishevelled*, *arrow*, *axin*, and *shaggy*/*GSK*-3. All of these genes were present in single copy in the assembly.

The *Gerris* Wnt ligand repertoire is comparable to other hemipterans and holometabolous insect species that have been analysed in detail. This supports observations of a reduction in the ligand repertoire in insects compared to an inferred ancestral complement of 17 subfamilies, with most extant Metazoan retaining ligands from 11-12 subfamilies. Nevertheless, assessments of gene absence need to be done with caution when dealing with draft assemblies from second generation sequencing, which is the case for most recently published genomes.

A total of 18 models for the main Wnt signalling genes were curated in the *Gerris buenoi* assembly (Table S14). The gene models generated by the MAKER pipeline were a very good start for the curation process in most cases, where most of the time only the 5' end of the models had to be edited by changing the translation start or adding upstream exons. The exceptions to this were the *dishevelled* isoforms where, despite very strong RNA-seq support for the complete model, only a small 5-exon model (for a gene with 16 exons in this species) for the middle part of the gene was present in the automated set. Despite curation, the models of three genes are incomplete. Similarly, *WntA* was missing the first exon in an upstream gap, and the *armadillo* model was missing the N-terminal region due to a gap directly upstream of the model. The third gene, *GSK-3 beta*, was split across two scaffolds despite strong RNA-seq support, with part 2 of this model filling the complete scaffold 10229 and yet still missing fragments at both ends.

All models were isolated on individual scaffolds, with the exception of *axin* and *arrow*. Interestingly, this linkage is not found in *Drosophila melanogaster, Tribolium castaneum,* or other i5k pilot project hemipteroid species surveyed to date. On the other hand, the absence of the ancient synteny of *wingless*-*Wnt6-Wnt10* [38], which was wholly or partially confirmed in other i5k pilot hemipteroid species, is likely due to limitations in the current draft assembly. Regarding gene copy number, it is worth noting that *armadillo*, which encodes an intracellular transducer in the Wnt pathway, is represented by a single ortholog in the current assembly. As many insects, including other heteropterans, have two copies of *armadillo* (*Drosophila, Tribolium, Cimex, Oncopeltus*), it is surprising that there is no evidence for a second gene in *Gerris*.

We identified 6 *Wnt* gene subfamilies in the *Gerris* assembly, all with single copy genes: *wingless/Wnt1, Wnt5, Wnt*7, *Wnt*8, *Wnt10* and *WntA*. This is identical to the ligand subfamily representation in *Oncopeltus fasciatus*, with the slight difference that there has been a duplication in *Oncopeltus Wnt8* [9]. There were also only six *Wnt* gene subfamilies found in the pea aphid (*Acyrthosiphon* *pisum*), although for a slightly different constellation of subfamilies: *wingless/Wnt1, Wnt5, Wnt* 7, *Wnt11, Wnt16* and *WntA* [18]*.* Together with earlier observations [38], this report supports the idea that members of the Hemiptera have the fewest *Wnt* gene families reported in insects, with some of these losses perhaps having occurred relatively recently and independently in this clade.

Three models were curated for the *frizzled* (*fz*) transmembrane receptor families: *frizzled*, *frizzled*-*2*, and *frizzled*-*3*. These correspond to three of the four ancient *fz* families expected to have been present in the common ancestor of arthropods: *fz*, *fz2*, *fz3*, *fz4* [39]*.* The loss of *fz4* was also observed in *Oncopeltus fasciatus* [9] and *Acyrthosiphon* *pisum* [18]*.*

### Cysteine peptidases from the papain C1 family

Cysteine peptidases from the papain C1 family (MEROPS classification [40]) are important lysosomal cathepsins, and participate as regulators and signaling molecules in a large number of biological processes [41]. In addition, cysteine cathepsins in a limited number of insect groups are important digestive enzymes evolved from lysosomal ancestors [42, 43]. In Cucujiformia beetles, digestive cysteine cathepsins are an evolutionary response to a seed diet rich in serine peptidase inhibitors [42, 44]. In the case of true bugs, it is proposed that their sap-sucking ancestors lost digestive serine peptidases in adapting to plant sap, and the adaptation of cysteine cathepsins for digestive functions is a consequence of a return to a protein diet [45]. A detailed study of cysteine cathepsins in the beetles *Tenebrio molitor* and *Tribolium castaneum* (Coleoptera: Tenebrionidae) revealed expansions of genes encoding cysteine digestive cathepsins [46, 47]. Cysteine cathepsins in *T. castaneum* larvae are important components of adaptive responses in overcoming the effect of dietary protease inhibitors [48].

There are few publications of cysteine peptidases in Heteroptera. Most of the early publications suggested that cysteine peptidases are the major digestive peptidases in several families of this insect order (see [42, 43]), such as *Reduviidae*, where digestive cathepsins L and B were identified in two *Triatoma* species [49, 50]. Sequencing the *Rhodnius prolixus* gut transcriptome revealed 11 cysteine peptidases expressed in the gut [51]. We are unaware of any publications on digestive peptidases of the bugs from the family Gerridae, and the specific biology of this semi-aquatic insect can impact the set of digestive enzymes.

In *Gerris buenoi,* we found 28 genes and gene fragments that encode cysteine cathepsins of the C1 family. These enzymes primarily belong to the cathepsin L-like subfamily [52], while the cathepsin B-like subfamily was represented by only three potentially active enzymes and one putatively catalytically inactive TINAL-like protein [53]. Members of the cathepsin L-like family included two types of peptidase genes: (i) those encoding conserved cathepsins, which include orthologs of mammalian cathepsin L and cathepsin F, and orthologs of cathepsin I and cathepsin Ll (26-29kD-proteinase) that are found in most insects (manuscript in preparation); (ii) 13 species-specific cathepsin L-like genes that do not have orthologs in other insects and are unique to *Gerris buenoi*, The cathepsin B-like family contained an ortholog of mammalian cathepsin B and two species-specific cathepsin B-like peptidase genes.

Conserved cathepsins of *Gerris buenoi* have a unique profile: there are eight cathepsin Ll genes, while in most species only one copy of the gene is found. Functional analysis of cathepsin Ll is premature, but previous studies suggested that those peptidases (26-29kD-proteinases) could play a role in immune defense system degrading foreign proteins [54] or participate in metamorphosis [47]. Species-specific cysteine peptidases include 15 different genes, 11 of which form two phylogenetic clades presumably derived from an original cathepsin L through the course of evolution, and localized as sequential clusters of 2 to 4 genes. Considering all Heteroptera species described thus far have digestive cysteine peptidases [49-51], we propose that they also may play a digestive role in *Gerris buenoi*. This hypothesis is supported by the fact that similar species-specific clades of cysteine peptidases in the more thoroughly studied coleopterans *Tribolium castaneum* [46, 47], *Tenebrio molitor* [46] and *Leptinotarsa decemlineata* [55] are linked to digestion of food.

### Visual genes

Water striders have drawn exceptional interest by visual scientists due to their exceptional visual ecology and correspondingly specialized organization of the visual system. The prominent, over 900 ommatidia counting compound eyes of water striders are involved in prey localization, mating partner pursuit, and predator evasion [56-58]. Although water striders utilize vision for dispersal by flight, water strider vision is considered specifically adapted to maximally sensitive 2-dimensional perception, i.e. the horizontal horizon of their water surface environment. Main evidence for this is the lateral acute zone, which facilitates neural superposition vision [59, 60]. Similar to higher Diptera like Drosophila, each ommatidial input is optically insulated from neighboring ommatidia through apposition optics. The sensitivity of target neurons in the lamina, however, is heightened at the level or neural organization of photoreceptor axons in target locations of the optic neuropils defined as neural superposition [56]. A likely functional morphological corollary of this is the open organization of the rhabdom in water strider ommatidia: Most of the individual photoresponsive membrane compartments (rhabdomeres) of each of the 8 photoreceptors per ommatidium are physically separated from each other [61]. This trait is shared derived trait for Heteroptera in contrast to Auchenorrhyncha and Coleorrhyncha [62], which feature a closed rhabdom where all rhabdomeres are in contact with each other along the proximodistal axis of the ommatidium. Each water strider ommatidium contains 6 outer and 2 inner photoreceptors. Recent work has produced evidence of at least 2 types of ommatidia with either green (~530nm) or blue (~470-490nm) sensitive outer photoreceptors [63], but the wavelength specificity of the two inner photoreceptors cells is still unknown.

Further notable for water strider vision is the dimorphism of ventral and dorsal ommatidia at the level of inner photoreceptor organization [62]. In the both dorsal and lateral ommatidia, both of the two inner photoreceptors contribute rhabdomeres in a highly organized orientation related to the rhabdomeres of the outer photoreceptors. In ventral ommatidia, by contrast, only the inner photoreceptor R8 forms a rhabdomere while the inner photoreceptor R7 does not. Interestingly, the specific orientation of the ventral R8 rhabdomeres is variable across Gerromorpha species. The tandem position of the R7 and R8 rhabdomeres in dorsal ommatidia has been proposed to be shared derived for Gerromorpha [62].

Typical for aquatic insects [64], *Gerris* is also polarized light-sensitive [65]. Schneider and Langer [61] describe how the cellular structure of photoreceptors relates to different polarized light sensitivities in the dorsal and ventral eyes. Studying the spectral sensitivity of *Gerris* photoreceptors to polarized light [63] concluded that the peripheral photoreceptors are either green or blue sensitive while the inner photoreceptors sensitivity remains unknown. On the other hand, Bartsch [66] recorded 37 photoreceptor cells, only 7 of which were blue sensitive while the rest were green sensitive. This study further revealed the existence of green and blue sensitive polarized light detecting subsystems in the lateral-equatorial and lateral-dorsal region of the eye. The green-sensitive subsystem has been proposed to mediate object detection while the function of the blue sensitive system has remained enigmatic.

Our genomic analysis of *G. buenoi* uncovered 8 opsin homologs (5 retinal and 3 non-retinal). The five retinal opsins (Figure 4A and Figure S2) were sorted into one member of the UV-sensitive opsin subfamily and 4 tightly tandem clustered members of the long wavelength sensitive (LWS) opsin subfamily (Figure 4A). The three extra-retinal opsins detected in the *Gerris* genome include: the deeply conserved yet functionally still poorly understood Rh7 opsin subfamily [67, 68], Arthropsin [69-71], and c-opsin (Figure S2 and Table S5). Only partial sequences Arthropsin and c-opsin were detectable in the *Gerris buenoi* genome assembly. However, complete transcript sequences were found in the transcriptome of the closely related water strider species *Limnoporus* *dissortis* (Figure S2).

Surprisingly, both genomic and transcriptome search in *G. buenoi* and other water strider species failed to detect sequence evidence of homologs of the otherwise deeply conserved blue-sensitive opsin subfamily (Figure 4B; Table S5) [72].

Although the apparent lack of blue opsin in *G. buenoi* was unexpected given the presence of blue sensitive photoreceptors [63], it was consistent with the lack of blue opsin sequence evidence in available genomes and transcriptomes of other heteropteran species including *Halyomorpha halys*, *Oncopeltus fasciatus*, *Cimex lectularius*, *Rhodnius prolixus*. Blue opsin, however, is present in other hemipteran clades, including Cicadomorpha (*Nephotettix cincticeps*) and Sternorrhyncha (*Pachypsylla venusta*) (Figure 4B). Taken together, these data lead to the conclusion that the blue-sensitive opsin subfamily was lost early in the last common ancestor of the Heteroptera (Figure 4B and Table S5). This raised the question of which compensatory events explain the presence of blue sensitive photoreceptors in water striders.

Studies in butterflies and beetles produced evidence of blue sensitivity shifts in both UV- and LWS-opsin homologs following gene duplication [73-75]. Given that the UV SWS-opsin family is generally conserved throughout insects even in crepuscular species like kissing bugs and bed bugs (Figure S2), and that evidence of UV-sensitive photoreceptors has been reported for backswimmers [76], it seems reasonable to hypothesize that one or more of the newly expanded *G. buenoi* LWS opsin genes represent blue-shifted paralogs. In further support of this hypothesis, the 4 *G. buenoi* LWS opsin paralogs have accumulated substantial sequence divergence amounting to pairwise 40 to 80 amino acid differences despite their tight genomic linkage, raising the possibility of wavelength-sensitivity change through adaptive tuning substitutions. In butterflies, molecular evolutionary studies have implicated amino acid residue differences at four protein sequence sites in sensitivity shifts from green to blue: Ile17Met, Ala64Ser, Asn70Ser, and Ser137Ala [73, 74] (Figure 4C, Figure S2 and Supplementary Data). We took two approaches to probe for the generality of the correlation of these protein sequence site states with wavelength specificity. First, we consulted sequence site information from physiologically characterized LWS opsins in other insect orders. This included the green-sensitive honeybee LWS opsin 1 (λ_max_ 544nm) and its blue-shifted homolog LWS opsin 2 (λ_max_ 490nm) [77], the green-sensitive Drosophila LWS opsin Rh6 (λ_max_ 515nm) and its blue-shifted paralogs Rh1 (λ_max_ 480nm) and Rh2 (λ_max_ 420nm), and the two green-sensitive LWS opsin paralogs of the cricket *Gryllus bimaculatus* (λ_max_ 515nm and λ_max_ 511nm) [62] (Figure 4B). In parallel, we assessed the degree of amino acid residue conservation at these sites in a sample of 114 LWS opsin homologs from 54 species representing 12 insect orders (Additional file 5 : File S4). Based on these criteria, sites 64 and 137 emerged as only ambiguous indicators of green vs blue sensitivity due high overall amino acid state variation in the 114 sampled opsin sequences and inconsistent representation of blue and green sensitivity states in the physiologically characterized opsin sequences of Drosophila, cricket, and the honeybee. Tuning sites 17 and 70, however, appear to be high confidence indicators of green vs blue-shifted LWS homologs. At site 17, the green-sensitive isoleucine state is found in the green-sensitive LWS-opsins of Drosophila, honeybee, and the cricket, while the blue-sensitive methionine state is shared with the blue-shifted opsin homologs of both Drosophila (Rh1) and the honeybee (LWS2) (Figure 4B). Further, the likely ancestral green-sensitive isoleucine state is present in over 70% of the surveyed 114 insect LWS opsins. Equally significant, the blue-sensitive methionine is the second most frequent state due to its conservation in dipteran orthologs of the blue-shifted Drosophila Rh1 (5) or hymenopteran orthologs of honeybee LWS opsin 2 (10). Thus, based on amino acid site 17, *G. buenoi* LWS opsin 2 and 4 represent green-sensitive paralogs while *G. buenoi* LWS opsin 1 and 3 represent likely blue-shifted LWS opsins.

Although less resolved, a similar picture emerges for site 70 where *G. buenoi* LWS opsin 3 stands out as a rare example of sharing a serine residue with blue-shifted butterfly LWS opsins. The putatively green-sensitive asparagine state, by contrast, is much more conserved, accounting for over 90% of the 114 insect LWS opsins surveyed, including even both blue-shifted Drosophila LWS opsins. Intriguingly, a cysteine is found at this site in the blue-shifted honeybee LWS opsin 2 homolog, which resembles serine as sulfur/selenium-containing amino acid residue (Figure 4B). The extreme rarity of the blue-sensitivity associated serine state at position 70 thus further supports *G. buenoi* LWS opsin 3 as blue-shifted together with the blue-shift indicative methionine at position 17.

Taken together, the comparative evidence identifies Gbue LWS opsin 3 as a candidate of blue-shifted paralog with the highest confidence followed by Gbue LWS opsin 1 and 2. This conclusion is further backed by the fact that water striders lack ocelli, which implies that all four paralogs are most likely expressed in photoreceptors of the compound eye. Overall, it thus seems most likely that the differential expression of the highly sequence-diverged Gbue LWS opsin paralogs accounts for the presence of both blue- and green-sensitive photoreceptors in water striders. Moreover, given that the outer blue photoreceptors have been specifically implicated in the detection of contrast differences in water striders [63], it is tempting to speculate that the deployment of blue-shifted LWS opsins represents another parallel to the fast-tracking visual system of higher Diptera. While these predictions await physiological verification in water striders, the genomic exploration of *Gerris buenoi* vision identifies water striders and Heteroptera as a whole as an exceptionally relevant group in the molecular study of adaptive visual system evolution for comparison to Lepidoptera, Hymenoptera, and the higher Diptera (Brachycera).

### Chemoreceptor gene families

The three chemoreceptor families addressed herein are the seven-transmembrane-domain Odorant and Gustatory Receptors that together comprise the insect chemoreceptor superfamily, and the unrelated three-transmembrane-domain Ionotropic Receptors [78, 79]. All three families have recently been fully documented from three other heteropterans with genome sequence used as comparators here, the kissing bug *Rhodnius prolixus* [80], the bedbug *Cimex lectularius* [81], and the milkweed bug *Oncopeltus fasciatus* [9]. More distant comparisons with other hemipteroid insects like the pea aphid *Acyrthosiphon pisum* [82] and the human body louse *Pediculus humanus* [16] are not included here as these chemoreceptors are mostly highly divergent from these four species, and comparisons including all five above species are available in Panfilio et al. [9].

The Odorant Receptors (ORs) is a large family, which, at least in several endopterygotes, have been shown to mediate most of insect olfaction (e.g. [79]). The OR family evolved within basal insects [83, 84] and consists of the single highly conserved Odorant receptor Co-receptor protein and a set of “specific” ORs, each of which is co-expressed with OrCo, generally one specific OR per olfactory sensory neuron type. The OR family in *Gerris* consists of at least 153 genes, two of which are modelled as being alternatively spliced in a fashion found in many other insects, with two long first exons encoding most of the protein that are alternatively spliced into several short-shared exons encoding the C-terminus. Thirteen of these OR genes are pseudogenic in the genome assembly, so the total of seemingly intact ORs in this compilation is 146, however many are partial models and many gene fragments remain. Phylogenetic analysis along with the other three heteropterans reveals the usual high conservation of the single OrCo proteins (Figure S5A). There are three possible simple orthologs of “specific” ORs across these four heteropterans, indicated with an asterisk in Figure S5A, and two more with simple duplications in one or more species (two asterisks). Otherwise the relationships consist either of highly divergent genes, or large expansions or “blooms” of ORs within a particular heteropteran lineage. In the case of *Gerris* these include expansions of 4 (Or64-67), 8 (Or145-152), 9 (Or90-97a/b), 13 (Or72-84), 13 (Or98-110), 16 (Or111-125), 18 (Or44-61), and 44 proteins (Or1-43). Comparable expansions were previously described in *Rhodnius* and *Oncopeltus* and are clear in this analysis as well (Figure S5A). In contrast, *Cimex* has almost no lineage-specific expansions, with OR clades consisting of only 1, 2, or 3 genes.

The Gustatory Receptors (GRs) is also a large family and consist of subfamilies and lineages that predate even the origins of the OR family [78, 84-86]. The most prominent of these are the sugar, carbon dioxide, and fructose receptor subfamilies (Figure S5B). The sugar receptors, represented here by Gr1/2 from *Apis mellifera*, were lost from the obligate blood feeders *Cimex* and *Rhodnius*, but are present as three genes each in *Oncopeltus* and this more general predator (Gr7-9). The carbon dioxide receptor subfamily, represented here by the Gr21a/62a dimer in *D. melanogaster* and Gr1-3 in *Tribolium castaneum*, was lost from most Hymenoptera as well as *Rhodnius*, but multiple related GRs are present in *Cimex*, *Oncopeltus*, and *Gerris* (Gr1-6). It remains to be shown whether these more distant relatives of the carbon dioxide receptors of endopterygotes are involved in perception of this molecule in heteropterans. The fructose receptor implicated also in brain nutrient sensing [87] has a single representative in each heteropteran, although the *Gerris* gene is represented only by a fragment in the current genome assembly (Gr10). This is the only GR lineage that is a simple ortholog across these four heteropterans. The remaining GRs present a pattern similar to that of most of the ORs, that is, a few highly divergent lineages, and several highly expanded lineages. In these GRs, however, these expansions mostly involve large alternatively-spliced loci, comparable to those found in many other insects from *D. melanogaster* [86] to *Calopteryx splendens* [83]. These loci consist of several long first exons encoding most of the receptor (transmembrane domains 1-6) that are modelled as being alternatively spliced into three short shared exons encoding the intracellular loop 3 and TM7. The three largest of these loci, Gr35, 48, and 32 encode 11, 11, and 13 different and sometimes quite divergent receptors, respectively (Figure S5B). The largest of these GR expansions consists of 80 proteins encoded by 27 genes (Gr22-48), while three smaller expansions of 10, 12, and 14 proteins also involve alternatively-spliced loci (Gr45-47, 55-60, and 15-19, respectively). This pattern of expansion of the “bitter” GRs in alternatively-spliced loci is shared with *Oncopeltus* where it has resulted in an even larger repertoire of “bitter” GRs, but barely at all in *Rhodnius* and *Cimex* both of which have comparatively small “bitter” GR subfamilies, presumably reflecting the different chemical ecologies of these four heteropterans.

The Ionotropic Receptors (IRs) is a variant family of the large and ancient superfamily of ionotropic glutamate receptors [78, 88]. The family contains two highly conserved co-receptors that are very similar to the ionotropic glutamate receptors in sequence and structure, Ir8a and 25a (Figure S5C), as well as another widely expressed gene that might also encode a co-receptor, Ir76b, specifically involved in perception of amino acids [89, 90]. These heteropterans have four more single-copy IRs (21a, 40a, 68a, and 93a), most of which are implicated in perception of a variety of stimuli from temperature to humidity [91, 92]. All of these are present as single-copy clear orthologs of the named *Drosophila* genes, and indeed most are older gene lineages than heteropterans [83]. An unusual exception is that there is a divergent duplicate of Ir8a (Ir8a2L) immediately upstream of and in tandem with Ir8a. This gene is missing the first 1/3 of the equivalent length of Ir8a, and there is no RNAseq support for it, unlike Ir8a and 25a, so it might not be functional. As is commonly the case in other insects, there is a small expansion to four genes of the lineage related to the Ir41a/76a/92a lineage in *D. melanogaster*, which for consistency with other genomes are named in an Ir41 series (Ir41d is not shown in Figure S5C because it is a partial model that does not align well). In *Drosophila* Ir41a and 92a have been implicated in detection of amines [93, 94]. A far larger expansion of 24 genes is related to the Ir75a-d/64a/84a lineage in *D. melanogaster*, and again this lineage is also expanded in many other insects, although seldom to this extent. Ir75a/b, 64a, and 84a in *Drosophila* flies have been shown to be involved in perception of several acids [95-99]. Like the other heteropterans and many other insects, there are several highly divergent IRs, falling into two groups with no simple relationships to *D. melanogaster* IRs. These were therefore named in a series from Ir101 to avoid confusion with *D. melanogaster* Ir genes, whose names only go to Ir100a because like the Or and Gr genes they were named for their cytological location in the polytene chromosomes. Ir101-105 are weakly related to a large expansion of so-called “divergent” IRs in *Drosophila*, including the Ir20a clade that function as gustatory receptors [100, 101]. Ir106-109 form a small clade related only to some other divergent heteropteran IRs, and are perhaps also involved in gustation. Thus, while not nearly as large as the OR and GR families, these IRs probably contribute some well-conserved functions shared with their orthologs with *Drosophila*, as well as perception of amines and diverse acids, and contribute to gustation. The only lineage-specific expansion compared with the other heteropterans is the IR75 clade implicated in perception of various acids, but it is unclear how this relates to the chemical ecology of water striders.

### Detoxification pathways

Cytochrome P450

Insect cytochrome P450 (CYP) proteins play a role in metabolic detoxification of xenobiotics including insecticides [102, 103]. They are also known to be responsible for the synthesis and degradation of endogenous molecules, such as ecdysteroids [104] and juvenile hormone [105]. The insect CYPs comprise of one of the oldest and largest gene families in insect, of which great diversity has been resulted from consecutive gene duplications and the subsequent diversification to extend the organism’s adaptive range [106].

A total of 103 CYP genes (Table S9 and Additional file 2 : File S1) were annotated and analyzed in the *G. buenoi* genome. Ten more CYP fragments were found, but they were not included in this analysis due to their short lengths (<250 aa). This is the largest number of CYP genes among the hemipteran species of which CYPomes were genome-widely annotated: *Rhodnius prolixus* (88 CYPs) and *Nilaparvata lugens* (68 CYPs) [107, 108]. It is also higher than that of the fruitfly, the honeybee, and the silkworm (Table S9). They fall into one of the four distinct groups of CYP gene family, named the Clan 2, Clan mito, Clan 3 and Clan 4, where 6 genes 62 genes 25 genes, and 10 genes are present, respectively.

Among the *G. buenoi* CYPs, the Clan 2 show high level of one to one orthology with other insects. The Gerris Clan 2 (6 genes) contains one gene of each CYP15, CYP303, CYP306, and CYP307, and two genes of CYP305 (Figure 5A). The duplicated CYP305s (CYP305A1 and CYP305A2) seems to be unusual compared to other insects, where a single CYP305A1 gene is present. In fact, these two genes are found in one scaffold (Scaffold443) of the *G. buenoi* genome in tandem suggesting a recent duplication. On the other hand, no orthologues of CYP18 and CYP304 were detected neither in transcriptome nor in genome sequences of *G. buenoi*. The mitochondrial Clan is also known to be highly conserved. Although the *G. buenoi* mitochondrial Clan (10 genes) does show such a one-to-one orthology only for CYP301A1, CYP302A1 and CYP404B1, an expanded cluster of CYP302Bs (7 genes) comprises a unique lineage in *G. buenoi* (Figure 5B). The other orthologues found in the other hemipteran mitochondrial Clan, such as CYP301B1, CYP314A1, CYP315A1, CYP353D1, CYP419A1, and CYP394B1 were not detected in *G. buenoi*. The Clan 3 is the largest clan showing the highest degree of gene expansion in insect CYP gene family. In the *G. buenoi* Clan 3 (62 genes), many genes might have undergone lineage-specific gene duplications, resulting in seven gene clusters (Figure 5C). In particular, CYP3096 is composed of 14 genes (including -A, -B, -C, and -D subfamilies), CYP3095A is of 10 genes, CYP6HL is of 8 genes, CYP3097A is of 7 genes, CYP6HK of 6 genes, CYP3091A is of 4 genes, CYP3092A is of 3 genes, CYP3085A is of 2 genes, CYP3086A is of 2 genes. There are four single-gene families, which are CYP3089A, CYP3090A, CYP3101A, CYP3102A, and CYP3103A. Interestingly, CYP9-like genes most likely found in *T. castaneum* and *B. mori* were not detected in the *G. buenoi* genome (Figure 5C). The *G. buenoi* Clan 4 contains 25 genes mostly belonging to CYP4 subfamilies and to the new family CYP3093. The *G. buenoi* CYP3093 forms a 10 duplicated gene cluster, suggesting a large gene expansion as shown in the *R. prolixus* CYP3093s (Figure 5D). There are two more gene clusters, CYP4EN (7 genes) and CYP4EM (5 genes), which seem to be homologous to the *R. prolixus* CYP4EMs. Finally, we found six intronless CYP genes, CYP306A1, CYP301A1, CYP6HL7, CYP4EM3, CYP4EM4, and CYP4EM5, which consist of a single exon in their genomic position. They might have been derived from an initially retrotransposed gene, because, for example, the orthologues of CYP306A1 in other insects have introns.

Overall, genome-wide analysis was performed to assemble and annotate the *G. buenoi* CYP gene family resulting in 103 genes. Phylogenetic analysis revealed not only their conserved orthology in insect, but also their lineage-specific gene expansions, suggesting the CYPs might have provided the water strider to adapt to the challenge in its unique environment. As the *G. buenoi* CYPs have not been highlighted so far, there will be many other interesting aspects to be explored in this multifunctional enzyme family.

UDP-glycosyltransferases

UDP-glycosyltransferases (UGTs) catalyze the conjugation of a range of diverse small hydrophobic compounds with sugars to produce water-soluble glycosides, playing an important role in the detoxification of xenobiotics and in the regulation of endobiotics. Insect UGT enzyme activity has been investigated in several species including the housefly *Musca domestica* [109], the fruitfly *Drosophila melanogaster* [110], the tobacco hornworm *Manduca sexta* [111], the silkworm *Bombyx mori* [112], and other insects [113], revealing that they play an important role in the detoxification and sequestration of a variety of plant allelochemicals and insecticides [114-118]. Enzyme activities of the insect UGTs are detected mostly in the fat body, midgut and other tissues [113], but also expressed in the antenna of *D. melanogaster* [119, 120] and *Spodoptera littoralis* [121]. In addition, many endogenous compounds, like ecdysteroid hormones [122] and cuticle tanning precursors [123, 124] are glycosylated by UGT enzymes. Furthermore, dietary flavonoids have been shown to be sequestered as glucose conjugates to impart color to the wings in a lycaenid butterfly [125] or in *B. mori* to be glycosylated to produce a green color in the cocoon with UV-shielding properties [115]. A UGT enzyme was recently shown to catalyze the final step in synthesis of cyanogenic glucosides by the Burnet moth *Zygaena filipendulae* [126]. These findings suggest multiple roles of the insect UGT enzymes in detoxification, olfaction, endobiotic modulation, and sequestration. Although a comprehensive genomic analysis of diverse insect UGTs was previously reported [127], hemipteran UGTs used in the analysis back then were only from an aphid species, *Acyrthosiphon pisum*. Together with other hemipteran genomes recently sequenced, the water strider (*Gerris buenoi*) genome could shed lights on the molecular evolution of this multigene family particularly in Hemiptera as well as generally in insects.

*Gerris buenoi* genome contains 28 putative UGT genes including several partial sequences due to genomic gaps (Table S10). There are fewer UGT genes in the water strider than in the pea aphid, *Acyrthosiphon pisum* (58 UGTs), but higher than in the bed bug, *Cimex lectularius* (7 UGTs). This is similar number found in the mosquito, *Anopheles gambiae* (26 UGTs). One interesting genomic feature of *G. buenoi* UGT repertoire is that such a large number of genes have been multiplied by tandem-gene duplication. In Scaffold1549, ten UGT genes are arrayed in a row, suggesting gene duplication events might produce such a large gene cluster (Figure S6). In addition, multiple genes lie in Scaffold1323, Scaffold3228, and Scaffold2126 with 4, 3, and 2 UGT genes, respectively. A consensus Maximum-likelihood tree (Figure S7) constructed with conserved C-terminal half of the deduced amino acid sequences from *G. buenoi* UGTs supports the clustered genes placed in the same genomic location are produced by gene duplication, suggesting such a tandem diversification of genes might lead to broaden the enzyme substrate range. Although any of the UGT genes have not been functionally characterized in the water strider yet, genomic analysis could give an insight on further studies on this interesting multigene family.

###

### Wing development and polyphenism

The ability to produce different phenotypes from a single genome in response to environmental cues is called ‘polyphenism’ [128]. Water striders express a seasonal wing polyphenism (Figure 1), where adults are short-winged in the early summer generation when habitats are stable, but are long-winged in the mid-summer generation when habitats become unstable [129, 130]. It is thought that this wing polyphenism reflects an adaptive tradeoff between wing length and reproduction, where in unstable habitats populations invest in long wings and produce fewer offspring, but in stable habitats populations produce short wings and invest in more offspring [129, 130]. The environmental cues that may affect wing morphology include photoperiod, temperature, resource availability, and population density [130-132].

Wing polyphenism and adaptive tradeoffs between flight and reproduction are ecologically important and phylogenetically widespread among insects. In wing polyphenic ants and aphids, for example, previous studies used bioinformatics approaches to infer that the genes involved in the development of wings and the ovaries have a different DNA methylation signature relative to the rest of the genome [133-137]. This suggests that these genes are regulated by epigenetic mechanisms [133-137]. Therefore, in the water strider *Gerris buenoi*, we predicted that genes involved in wing patterning and reproduction will also have a different DNA methylation signature relative to the rest of the genomes. Furthermore, previous studies have shown that juvenile hormone (JH) and insulin signaling pathways are associated with regulation of reproduction and wing polyphenism in insects [128, 138-140]. We therefore analyzed epigenetic signatures in genes involved in both of these pathways relative to the rest of the genome. Finally, we compared genes from *Gerris buenoi* to orthologues in *Rhodnius proxilus* because this closely related species serves as a phylogenetically controlled outgroup, which has not evolved wing polyphenism.

We discovered that the mean CpG_O/E_ values for *Gerris buenoi* genes in the network related to wing polyphenism, juvenile hormone, insulin signalling and reproduction are not significantly different from the mean of the resampled distribution of CpG_O/E_ of all *Gerris buenoi* genes (Figure S9 and Table S15). The mean CpG_O/E_ of the *R. proxilus* orthologues related to wing polyphenism, juvenile hormone regulation, insulin signalling and reproduction is also not significantly different from the mean of the resampled distribution of CpG_O/E_ of all *Rhodnius proxilus* (Figure S9). These results indicate that genes in the network related to wing polyphenism, juvenile hormone, insulin signalling and reproduction do not have a distinct methylation signature relative to the rest of genes in *Gerris buenoi* and *Rhodnius proxilus* genomes.

The sequencing of three ant genomes, each of which possess a dramatic wing and reproductive polyphenism, showed significant methylation signature of genes known to be involved in wing and reproductive development relative to the rest of the genes in the ant genomes [133-135]. We therefore expected that genes involved in wing and reproductive development in the wing polyphenic water strider *Gerris buenoi* would possess a similar methylation signature as in the ants. To our surprise, the results of our analysis reveal that methylation signatures in genes involved in wing and reproductive development are not significant relative to the rest of the genome. This is also the case for the closely-related and non-wing polyphenic insect *Rhodnius proxilus*. These findings suggest that more classical mechanisms for achieving differential gene expression underlying polyphenism, such as endocrine-based mechanisms like hormone secretion and neuropeptide release, are involved in regulating the expression of genes underlying wing polyphenism as well as the trade-off between wing development and reproduction in water striders [141]. Altogether, these results open up exciting future research possibilities for understanding how wing polyphenism is regulated in water striders, and why they appear to differ from other polyphenic insects.

### DNA methylatransferases

DNA methylation is an epigenetic mechanism known to be involved in the regulation of alternative splicing and gene expression in insects [142-144]. In honeybees, it has been demonstrated that the DNA methyltransferase, DNMT3, is critical in sizing, morphology and reproductive organ development associated with caste determination as well as alternative splicing regulation [143-145]. Furthermore, differential DNA methylation is associated with flexible behavioral castes (nurses and foragers) in bees [146]. Therefore, this epigenetic mechanism is considered to be a potentially key regulator of morphological development and behavioral differentiation in insects. Paradoxically, many insects have lost key elements of the DNA methylation toolkit, including DNMT1 and DNMT3, as is the case for *Drosophila melanogaster* [147]. In order to see if this pathway may be worth further investigation for the study of morphological development in water striders, we searched for several core elements that regulate this molecular process. Although we found that the water strider genome does possess *DNMT1*, which is essential for the maintenance of DNA methylation, and *DNMT2*, the protein of which functions to methylate tRNAs, the *Gerris buenoi* genome does not contain an ortholog of *DNMT3*, which is essential for de novo DNA methylation. It is hard to predict the significance of *Gerris buenoi* lacking *DNMT3* because the presence versus absence of this gene is quite erratic across insects [148]. Although it may be associated with the capacity for elaborate environmentally-dependent developing processes, including those that are polyphenic as it is found in a range of invertebrates including the pea aphid [149], Daphnia [150], termites [151] and various hymenoptera including bees and ants that are highly plastic [133, 152, 153]. Still, there are other highly conserved epigenetic processes, such as histone modifications, which are conserved in *Gerris buenoi*, and may serve as alternative mechanisms for the regulation of developmental plasticity.

### Histone genes and histone modification machinery

Chromatin remodelling, via post-translational modifications of histones, is a key regulator of gene expression. These epigenetic processes have been associated with environmental responsiveness and phenotypic plasticity [154]. One of the most striking cases of plasticity in the Gerridae is associated with wing development [155]. Most species of this family exhibit winged and wingless morphs known as apterous and macropterous morphs [155, 156]. Wing development is influenced by both genetic and environmental factors such as habitat stability, day/night cycle and latitude [129, 130, 157]. Other cases of phenotypic plasticity include leg length, pigmentation, and a set of secondary sexual traits in both males and females [158]. While our understanding of the ecology of these cases of phenotypic plasticity is increasingly richer, the lack of a water strider genome has hindered studies of the genetic and developmental factors associated with them. We therefore analysed the *Gerris* *buenoi* genome content in search for components of the epigenetic machinery.

In the *Gerris buenoi* genome we could identify 49 histone proteins encoding loci, a moderately large number of genes similar to that found in *Cimex lectularius* and *Daphnia pulex*, but substantially smaller than that detected in the *Aedes aegypti* or *Drosophila* genomes (Table S16). We identified genes encoding the five major classes of histone proteins (H2A, H2B, H3, H4 and the linker histone H1) as well as copies of genes encoding the variant histones H2AV and H3.3. In *Drosophila* the histone genes are present in the genome in large numbers of quintet clusters, each cluster having one gene from each of the five classes of histones. A similar organization was found in the *Gerris buenoi* genome where two canonical quintet clusters were identified. Both of them consists of one copy of each of the four classes of core histone proteins (H2A, H2B, H3 and H4) and a single copy of the linker histone (H1) (Figure S10). Additional clusters were identified, including one modified cluster containing two copies of the linker histone (H1) and two copies of the H2B core histone, but no copy of the core histone H3, as well as five truncated clusters made of three or four genes including H3 core histone gene and combinations of the other histone genes (Figure S10). The number of these clusters is higher compared to the genomes of the milkweed bug *Oncopeltus fasciatus* and the bed bug *Cimex lectularius,* which contain one and two clusters respectively [9, 81]. The functional significance of these clusters remains unknown, thus opening new avenues in the study of the relationship between epigenetics and phenotypic plasticity [159].

Histone proteins can be post-translationally modified to dynamically influence the structure of the chromatin. We found in the *Gerris* *buenoi* genome genes responsible for all classes of histone modifications: histone acetyltransferases, deacetylases, methylases and demethylases. Interestingly, we found a duplication of the histone acetyltransferases *males absent on the first* (*mof*) and *chameau* (*chm*/HAT1). *Mof* functions in dosage compensation and genome stability in *Drosophila* [160, 161]. Duplications of *mof* and *chm* have previously been reported for *Acyrthosiphon pisum* and were thought to be unique [162] although *mof* duplication was also recently detected in *Oncopeltus fasciatus* [9] and *Cimex lectularius* [81]. Phylogenetic analysis indicates the duplications that have occurred in these species are independent of the duplication that occurred in *Acyrthosiphon pisum* and likely occurred early in the heteropteran lineage ~250 million years ago (Figure S11). Unusually, we also identified a duplication of the *Gerris buenoi* histone deacetylase *Sirt1* (*sir2*) and *Sirt5*; and the histone methyltransferase *grappa*. Sirt1 is a nuclear and cytoplasmic deacetylase that has a role in histone modifications [163] and has been associated with enhanced stress response and life-span extension in numerous species [162, 164, 165]. Grappa, histone methyltransferase, modifies the lysine (K)79 residue of histone H3 and has been implicated in the stress response in *Drosophila* providing protection against oxidative and caloric stress [166]. Interestingly, duplications of *Grappa* have not been detected in any other hemipteran species.

In conclusion, the high number of histone clusters found as well as the duplication of some post-translational modifications of histones genes open up exciting future research possibilities for understanding their role in environmental responsiveness and phenotypic plasticity in *Gerris buenoi*.

### Antioxidant Proteins

Reactive oxygen species (ROS), including superoxide radicals (O_2_^-^), hydroxyl radicals (OH^-^), and hydroperoxides (H_2_O_2_, and ROOH), are generated by aerobic metabolism but may also be encountered in an organism diet or environment [167-169]. Moderate levels of ROS drive a variety of processes including cellular signaling, transcriptional regulation, as well many other physiological processes. However, inability to regulate ROS concentrations can result in the accumulation of ROS-induced damaged lipids, proteins, and nucleic acids [167-169]. Animals have evolved a complex system of antioxidant enzymes and molecules, facilitating the modulation of ROS levels [168, 170-172]. The enzymatic antioxidant system is comprised of a diverse suite of proteins that can be divided into clades based on their modes of action. Catalase (CAT), superoxide dismutase (SOD), and a variety of peroxidases make up the core of the antioxidant response. Thioredoxins and methionine sulphoxide reductases form a secondary system for managing ROS [170, 171].

Thirty putative proteins in seven families related to antioxidant capacity were identified within the *G. buenoi* genome. The thirty antioxidant response proteins showed high homology to related proteins in other published genomes including *Acyrthosiphon pisum, Apis mellifera, Bombyx mori, Cimex lectularis, Drosophila melanogaster, Pediculus humanus, and Tribolium castaneum* (see Supplementary Methods). In most comparisons, homologs in *C. lectularis* genome showed the highest degree of similarity (Table S17). Representatives of all major antioxidant enzyme clades were identified in the *G. buenoi* genome assembly including a *Catalase*-like gene, four heme-binding peroxidases, multiple glutathione-s-transferases, peroxidase, multiple peroxiredoxins, and superoxide dismutases (Additional file 11 : File S10). This representation suggests that the *G. buenoi* genome contains a complete suite of antioxidant enzymes. There is no apparent expansion or reduction in the gene families that were surveyed in this analysis, however further investigation through additional annotation and experimental validation may reveal otherwise.

##

## **Supplementary Methods**

### Genome sequencing and assembly

*Gerris buenoi* is one of thirty arthropod species sequenced as a part of a pilot project for the i5K arthropod genomes project at the Baylor College of Medicine Human Genome Sequencing Center. For all of these species, an enhanced Illumina-ALLPATHS-LG sequencing and assembly strategy enabled multiple species to be approached in parallel at reduced costs. For most species, including *Gerris buenoi*, we sequenced four libraries of nominal insert sizes 180bp, 500bp, 3kb and 8kb. The amount of sequence generated from each of these libraries is noted in Table S18 with NCBI SRA accessions. The 180bp, 500bp and 3kb mate pair libraries were made from a single male individual, and the 8kb mate pair library from female genomic DNA.

To prepare the 180bp and 500bp libraries, we used a gel-cut paired end library protocol. Briefly, 1 µg of the DNA was sheared using a Covaris S-2 system (Covaris, Inc. Woburn, MA) using the 180-bp or 500-bp program. Sheared DNA fragments were purified with Agencourt AMPure XP beads, end-repaired, dA-tailed, and ligated to Illumina universal adapters. After adapter ligation, DNA fragments were further size selected by agarose gel and PCR amplified for 6 to 8 cycles using Illumina P1 and Index primer pair and Phusion® High-Fidelity PCR Master Mix (New England Biolabs). The final library was purified using Agencourt AMPure XP beads and quality assessed by Agilent Bioanalyzer 2100 (DNA 7500 kit) determining library quantity and fragment size distribution before sequencing.

The long mate pair libraries with 3kb or 8kb insert sizes were constructed according to the manufacturer’s protocol (Mate Pair Library v2 Sample Preparation Guide art # 15001464 Rev. A PILOT RELEASE). Briefly, 5 µg (for 2 and 3-kb gap size library) or 10 µg (8-10 kb gap size library) of genomic DNA was sheared to desired size fragments by Hydroshear (Digilab, Marlborough, MA), then end repaired and biotinylated. Fragment sizes between 3-3.7 kb (3kb) or 8-10 kb (8kb) were purified from 1% low melting agarose gel and then circularized by blunt-end ligation. These size selected circular DNA fragments were then sheared to 400-bp (Covaris S-2), purified using Dynabeads M-280 Streptavidin Magnetic Beads, end-repaired, dA-tailed, and ligated to Illumina PE sequencing adapters. DNA fragments with adapter molecules on both ends were amplified for 12 to 15 cycles with Illumina P1 and Index primers. Amplified DNA fragments were purified with Agencourt AMPure XP beads. Quantification and size distribution of the final library was determined before sequencing as described above.

Sequencing was performed on Illumina HiSeq2000s generating 100bp paired end reads. Reads were assembled using ALLPATHS-LG (v35218) [173] on a large memory computer with 1Tbyte of RAM and further scaffolded and gap-filled using in-house tools Atlas-Link (v.1.0) and Atlas gap-fill (v.2.2) [174]. This yielded an assembly of 1 000.16 Mb (653 Mb without gaps within scaffolds) with a contig N50 of 3.8 kb and scaffold N50 of 344kb which has been deposited in the NCBI: GenBank assembly accession GCA_001010745.1

### Automated Gene Annotation Using a Maker 2.0 Pipeline Tuned for Arthropods

Of 30 attempted i5K pilot species, 28 i5K pilot genome assemblies including *G. buenoi* were subjected to automatic gene annotation using a Maker 2.0 annotation pipeline tuned specifically for arthropods. The pipeline is designed to be systematic providing a single consistent procedure for the species in the pilot study, scalable to handle 100’s of genome assemblies, evidence guided using both protein and RNA-seq evidence to guide gen models, and targeted to utilize extant information on arthropod gene sets. The core of the pipeline was a Maker 2 [175] instance, modified slightly to enable efficient running on our computational resources. The genome assembly was first subjected to de-novo repeat prediction and CEGMA analysis to generate gene models for initial training of the ab-initio gene predictors. Three rounds of training of the Augustus [176] and SNAP [177] gene predictors within Maker were used to bootstrap to a high quality training set. Input protein data included 1 million peptides from a non-redundant reduction (90% identity) of Uniprot Ecdysozoa (1.25 million peptides) supplemented with proteomes from eighteen additional species (*Strigamia maritima, Tetranychus urticae, Caenorhabditis elegans, Loa loa, Trichoplax adhaerens, Amphimedon queenslandica, Strongylocentrotus purpuratus, Nematostella vectensis, Branchiostoma ﬂoridae, Ciona intestinalis, Ciona savignyi, Homo sapiens, Mus musculus, Capitella teleta, Helobdella robusta, Crassostrea gigas, Lottia gigantea, Schistosoma mansoni*) leading to a final ‘nr’ peptide evidence set of 1.03 million peptides. RNA-seq transcription data derived from mixed sex embryo’s and nymphs (Table S18) was used judiciously to identify exon-intron boundaries but with a heuristic script to identify and split erroneously joined gene models. We used CEGMA models for QC purposes: for *Gerris buenoi*, of 1 977 CEGMA single copy ortholog gene models, 1 783 were found in the assembly and 1 895 in the final predicted gene set – a reasonable result given the small contig sizes of the assembly. We assume the gene predictors could pull together exons from different contigs with greater success than the sequence comparison used to identify CEGMA genes in the assembly, generating the larger number of control gene models found in the gene set than the underlying assembly. Finally, the pipeline uses a nine-way homology prediction with human, *Drosophila* and *Caenorhabditis elegans*, and InterPro Scan5 to allocate gene names. The automated gene sets are available from the National Agricultural Library [178] where a web-browser of the genome, annotations, and supporting annotation data is accessible.

### Community annotation and Official Gene Set generation

The National Agricultural Library’s i5k Workspace@NAL [179] implemented the Apollo manual annotation software [180] to facilitate community annotation of the Gerris buenoi genome [178]. Volunteer annotators received training in manual annotation via webinar, and were asked to follow a set of annotation guidelines [181]. Once completed, the manual annotations were checked for quality and merged with the automated MAKER2 annotations Gbue_0.5.3 using the NAL’s GFF3toolkit pipeline [182]. Locally unique IDs were generated using in-house scripts, resulting in the non-redundant Official Gene Set OGSv1.0 [183]. Community annotators contributed or modified 1 277 genes, comprising 1 378 mRNAs and 15 pseudogenes in the combined OGSv1.0.

### Bristle genes

Bristle development genes were annotated by performing tblastn searches on the *Gerris buenoi* scaffolds with the corresponding Drosophila gene protein sequences available in FlyBase (release 6)[184]. To confirm orthology, *Gerris buenoi* models were blasted into NCBI 'nr' database. Homology, intron/exon boundary assessments, and protein sequence completeness were identified by manual inspection using RNA-seq alignments available and protein alignments generated with Clustal Omega [185].

### Cuticular proteins

Sequence motifs that are characteristic of several families of cuticle proteins [186] were used to search the genome of *Gerris buenoi* for putative cuticle proteins. 155 genes were identified, analyzed with CutProtFam-Pred, a cuticular protein family prediction tool described in Ioannidou et al. [187], and assigned to one of 5 families (CPR, CPAP1, CPAP3, CPF, and TWDL) (Additional file 9 : File S8).

### Prey detection and selection on water environments

The approach for manual annotation is similar to that used to characterize these three gene families in many other insects, including *Acyrthosiphon* *pisum* [82], *Pediculus* *humanus* [16], *Rhodnius prolixus* [80], *Cimex lectularius* [81] and *Oncopeltus fasciatus* [9]. Briefly, exhaustive and iterative tblastn searches of the genome assembly with the proteins from these other heteropterans were used to find genes, which were modelled as best possible in the WebApollo browser at the i5k site. This effort was sometimes assisted by RNA-seq reads that cross introns in the available whole-body RNA-seq set, however most of these genes were not represented in that dataset. In addition, like *Oncopeltus fasciatus* this genome assembly is rather fragmented, so many of the models are incomplete, while some were joined across scaffolds and a few were improved with raw reads. Several additional gene fragments too short to include in this compilation remain for the OR and GR families and might represent additional intact genes, while some of the partial models might actually be pseudogenes. Many of these proteins are extremely divergent, and because almost none of them were modelled by the genome-wide automated annotation (models that might have facilitated searches for distant relatives using BLASTP), TBLASTN searches to find distant relatives used E values of 1 000. The last two exons of the OR and GR families typically encode the most conserved regions of these proteins and are flanked by phase 0 introns, so their encoded protein sequences were used in TBLASTN searches with LQ before and VS afterwards, representing consensus splice acceptor and donor sites, to assist in finding divergent relatives. Multiple alignments of each family along with representatives from other species and maximum likelihood phylogenetic analyses of the proteins were conducted, and the tree figures prepared, as in Panfilio et al. [9]. All of the proteins are included at Additional file 8 : File S7, and the gene models and transcribed mRNAs for most of them are available from the i5k Workspace at the National Agriculture Library ([178]).

### Wing polyphenism

First, we limited our analysis to genes whose complete coding sequences had been identified and annotated in the following four categories: genes involved in wing polyphenism, juvenile hormone regulation, the insulin signalling pathway, and reproduction. We then used the bioinformatics-based metric described by Elango *et al.* [137] called CpG_O/E_ as a proxy for mutations induced by methylation of CpG islands in the germ line over evolutionary time. This CpG_O/E_ metric uses a historical (evolutionary) measure of the level of DNA methylation by estimating the amount of CpG dinucleotide depletion normalized for GC content for each gene of interest. The CpG_O/E_ metric, or CpG dinucleotide depletion normalized for GC, is a proxy for DNA methylation in the coding sequence of these genes. We define the CpG_O/E_ for each gene as follows:

$$C_{P}G_{O/E}=\frac{P_{CpG}}{P_{c}P_{G}}$$

where CpG_O/E_ is an estimation of the DNA methylation levels, P_CpG_ is the frequency of CG dinucleotides, P_C_ is the frequency of cytosine nucleotides, and P_G_ is the frequency of guanine nucleotides [188, 189]. After cytosine is methylated, it is more amenable to deamination [189]. Over time, this leads to the reduction of CpG dinucleotides from methylated CpG regions [189]. Using a custom Perl script, we evaluated the CpG_O/E_ in the coding sequences of all predicted genes in the *Gerris buenoi* genome and the CpG_O/E_ in the coding sequences of our genes of interest (Table S15).

Second, we compared the mean CpG_O/E_ content for our genes of interest to the mean CpG_O/E_ for all the genes in the genome by executing a Monte-Carlo randomization procedure as described previously [133-137]. Briefly, we randomly selected 50 CpG_O/E_ values from the genome to produce a random distribution, calculated the mean, and repeated this process 10000 times. All mean CpG_O/E_ values were plotted and this distribution was compared to the mean CpG_O/E_ values for each of our candidate gene sets. Gene sets were determined to be significantly different from the randomly generated mean CpG_O/E_ if they fell within the bottom or top 5% of values. These analyses were repeated for *Rhodnius proxilus* orthologues of the *Gerris buenoi* genes in our gene sets.

### Wnt Signaling Pathway

Protein sequences for *Wnt* ligands as well as receptors and downstream components (*armadillo*/*beta*-*catenin*, *dishevelled*, *frizzled*, *arrow*, *axin*, *shaggy*/ *GSK*-3) from *Drosophila* *melanogaster*, *Tribolium* *castaneum*, *Acyrthosiphon* *pisum* and *Oncopeltus fasciatus,* were retrieved from NCBI, and used to perform standalone tblastn searches on the *Gerris buenoi* scaffolds with a maximum e-value of 1e^-10^. Hits from all species together were ordered by scaffold and start position, and for each group of overlapping or closely adjacent hits from multiple orthologous queries, the putative gene name was identified by blasting back the hit sequence against GenBank, with a taxonomic restriction to Arthropoda accessions. The query sequences with the best hits (lowest e-values) for each gene were then used to identify the model to be curated, by doing a tblastn search into the *Gerris* scaffolds from the Blast instance at the National Agricultural Library [190]. The Blast results were visualized in the Web Apollo instance for *Gerris buenoi* [191], where the corresponding automated annotation models were edited. To confirm orthology, we then Blasted the edited *Gerris buenoi* models back into GenBank. Homology, intron/exon boundary assessments, and protein sequence completeness were identified by manual inspection and correction of protein alignments generated with Clustal Omega [185].

The numbering (subfamily identification) for *Wnt and fz* orthologs was assigned based on the corresponding vertebrate homolog (the naming of *Drosophila* orthologs was changed accordingly), based on phylogenetic analyses done at [192].

Possible gene loci duplications were identified by performing tblastn searches on the scaffolds using the protein sequences of completed *Gerris* annotation models as queries, and then re-blasting the resulting hit sequences into GenBank for Arthropoda hits.

### Early Developmental Genes

The choice of early developmental genes (Gap, Pair Rule, and Segment Polarity Genes) to annotate was informed by GO term annotations in *Drosophila melanogaster* (long-germ) and *Tribolium castaneum* (short-germ). Protein sequences for developmental genes for *D. melanogaster* and *T. castaneum* were obtained from FlyBase [184] and BeetleBase [193] respectively. Contig sequences were searched for homology to the selected protein sequences using tbastn. Gene models (Gbue v0.5.3-models) that aligned with the regions of highest homology identified by tbastn search were selected for further analysis. If no official gene model was present in the region of homology identified by tblastn a de novo model was generated using models generated by the Augustus-masked or snap-masked programme. RNAseq mapped reads were compared with the gene models to determine the transcribed regions. The transcribed regions were used to determine protein sequences of the gene. Protein sequences were utilised in a reciprocal blast (blastx NCBI) to confirm the homology of the orthologs. Gene models were manually edited to produce gene models that resolved conflicts between RNAseq, blastx and homology data.

### Antioxidant genes

Antioxidant proteins of *Drosophila melanogaster* were utilized to initially identify potential antioxidant genes within the *Gerris buenoi* genome. The *Drosophila melanogaster* genes were obtained from FlyBase by generating a query that searched for proteins with Gene Ontology terms that were related to response to antioxidant activity and responses. These nucleotide sequences were translated to peptides and were searched against the peptide models of *G. buenoi*. The highest BLAST hit (blastp) was extracted and searched against arthropod entries of the NCBI non-redundant database to confirm the identity of the model (blastp). The confirmed model was then BLAST searched (blastp) against the peptide sequences of *Acyrthosiphon pisum, Apis mellifera, Bombyx mori, Cimex lectularis, Drosophila melanogaster, Pediculus humanus, and Tribolium castaneum* to extract homologs. The extracted *G. buenoi* model was then aligned to the homologs. This information and the RNA-seq data present in the WebApollo were used to manually annotate the model. The corrected model was then once more searched against the arthropod entries of the NCBI non-redundant database (blastp) to ensure that the model was correctly identified.

## **Figure legends**

**Figure S1:** Phylogram of InR evolution as depicted in Figure 3B (circulized). Color codes for species order: Aranae (light purple), Geophilomorpha (light pink), Cladocera (blue), Ephemeroptera (red), Orthoptera (dark blue), Blattodea (light blue), Hemiptera (orange), Phthiraptera (brown), Hymenoptera (pink), Coleoptera (olive yellow), Lepidoptera (purple) and Diptera (green).

**Figure S2**: Phylogenetic analysis and representative sequences from all major insect opsin subfamilies. Protein sequences were aligned with T-Coffee [194] and ambiguous multiple alignment alignment segments were removed applying the “gappyout” setting of TrimAl (v. 1.3) [195]. A neighbor joining tree was estimated in MEGA version 6.0 [196] using gamma-corrected Jones-Taylor-Thornton distances [197] and testing branch support with 1 000 bootstrap samples (numbers at branches). Species abbreviations: Amel = *Apis mellifera*, Apisum = *Acyrthosiphon pisum*, Asie = *Anotogaster sieboldii*, Btab = *Bemisia tabaci*, Cariz = *Clastoptera arizonana*, Clec = *Cimex lectularius*, Carid= *Cuerna arida*, Dcit=*Diaphorina* citris, Evit = *Empoasca vitis*, Gbue = *Gerris buenoi*, Gatr = *Graphocephala atropunctata*, Hhal = *Halyomorpha_halys*, Hlit = *Homalodisca liturata*, Ldis = *Limnoporus dissortis*, Ncin = *Nephotettix cincticeps*, Phum = *Pediculus humanus*, Rpro = *Rhodnius prolixus*, Tcas = *Tribolium castaneum*.

**Figure S3:** Comparison of predicted aquaporins from *Gerris* and *Cimex* using Neighbor-joining tree produced using MEGA6 using Dayhoff model and pairwise matching; branch values indicate support following 1500 bootstraps; values below 50% are omitted. It includes the seven putative aquaporin (AQP) genes identified from the water strider that includes the typical *Drosophila* integral protein (Drip), AQP2, AQP4 (Two genes), AQP5, AQP6 and Big brain (Bib) genes. In addition to these seven, we identified one other predicted partial sequence with matches to AQP sequences from other insects. Overall the number of aquaporins falls within the range of most insects (6-8) and *Gerris* has members of each group previously identified for insects [81].

**Figure S4:** Phylogenetic tree demonstrating relationships of TWDL genes from *Gerris buenoi*, *Drosophila melanogaster*, *Tribolium castaneum*, *Apis mellifera*, *Pediculus humanus* *corporis*, *Acyrthosiphon pisum*, *Bombyx mori*, *Cimex lectularius*, and *Oncopeltus fasciatus*. *G. buenoi* showed a greater number of TWDL genes than other insects, with the notable exception of dipterans such as D. melanogaster. The tree was constructed using the neighbor-joining method in MEGA6 with Poisson correction and bootstrap replicates (10 000 replicates).

**Figure S5:** Phylogenetic analysis of the Chemoreceptor families. **(A)** **Olfactory Receptor family.** The tree was rooted with the highly conserved and basal OrCo proteins. A single asterisk indicates possible simple orthologous relationships and two asterices indicate slightly more complicated relationships involving independent duplications in one or more species. Protein names and the branches leading to them are colored in blue for *Gerris*, brown for *Rhodnius*, red for *Cimex*, and orange for *Oncopeltus*. A suffix of P after the protein number indicates a pseudogene, while alternatively-spliced ORs are indicated by lower case letters after the protein number. Support for nodes is the aLRT value from PhyML v3.0. **(B)** **Gustatory Receptor family.** The tree was rooted with the conserved sugar and carbon dioxide receptor subfamilies. These two subfamilies and the fructose receptor subfamily are highlighted by colored background wedges. **(C)** **Ionotropic Receptor family.** The tree was rooted with the conserved co-receptor Ir8a and 25a lineages, which closely resemble the ionotropic glutamate receptors from which these variant Ionotropic Receptors evolved. The entire *D. melanogaster* IR repertoire was included for comparison. Lower case suffixes do not indicate alternative-splicing, but rather either orthology with particular *Drosophila* IRs, or the Ir41 and 75 series of genes.

**Figure S6:** Genomic orientation of UGT genes in a *Gerris* *buenoi* genomic scaffold. Ten UGT genes are arrayed in a row in Scaffold1549, probably multiplied by gene duplication events.

**Figure S7:** A consensus Maximum-likelihood tree of C-terminal half of the deduced amino acid sequences of *Gerris buenoi* UGTs. The phylogeny was inferred by the method based on the JTT matrix-based model. Bootstrap value was 1 000.

**Figure S8:** Simplified cladogram of Hemiptera based on [198] depicting IMD presence (green) and absence (red).

**Figure S9:** Density plot of frequency (y-axis) versus mean CpG_O/E_ (x-axis) for (A) *Gerris buenoi* (n = 20 949; overall mean = 0.70; mean of wing genes = 0.74; mean of juvenile hormone genes = 0.68; mean of insulin signalling genes = 0.66; mean of reproduction genes = 0.70; p > 0.05)and (B*) Rhodnius prolixus* (n = 15 081; overall mean = 0.71; mean of wing genes = 0.72; mean of juvenile hormone genes = 0.69; mean of insulin signalling genes = 0.76; mean of reproduction genes = 0.71; p > 0.05). The observed mean for genes in the networks underlying wing polyphenism (black line), reproduction (yellow line), juvenile hormone (green line), and insulin signalling (orange line) plotted relative to the distribution of CpG_O/E_ values for all genes in the genome (random resampling of mean CpG_O/E­­­­_ from 50 genes in the genome).

**Figure S10:** Genomic organisation of the histone loci gene clusters annotated in the *Gerris buenoi* genome. Clusters were defined as more than one histone encoding gene present on a genomic scaffold. No clusters were found that were interrupted by non-histone gene encoding loci. Clusters were visualized using genometools v1.5.5 and coloured according to orthology group (Histone H1 (red), Histone H2A (dark blue), Histone H2B (light blue), Histone H3 (green), Histone H4 (yellow).

**Figure S11:** Phylogeny of histone acetyltransferases in Heteropteran lineage. Results show a duplication of *males absent on the first* (*mof*) and *chameau* (*chm*/HAT1) in *Gerris buenoi* similar to previous results in *Oncopeltus fasciatus* [9] and *Cimex lectularius* [81] but also a unique duplication of *Gerris buenoi* histone deacetylase *Sirt1* (*sir2*) and *Sirt5*; and the histone methyltransferase *grappa*.

**Tables:**

| 910 | CompleteBUSCOs |
| --- | --- |
| 14 | - of which duplicated |
| 131 | Fragmented BUSCOs |
| 25 | Missing BUSCOs |
| 1,066 | Total BUSCO groups searched |

Table S1 : Summarized benchmarks in BUSCO notation

|  | **Complete** | **- of which duplicated** | **Fragmented** | **Missing** |
| --- | --- | --- | --- | --- |
| *Drosophila melanogaster* | 98 | 6,4 | 0.6 | 0.3 |
| *Danaus plexipus* | 83 | 8.6 | 11 | 4.3 |
| *Apis mellifera* | 93 | 2.9 | 5.1 | 0.9 |
| *Pediculus humanus* | 92 | 3.9 | 6.1 | 1.6 |
| *Daphnia pulex* | 83 | 3.9 | 11 | 5.1 |
| *Tribolium castaneum* | 95 | 5.8 | 3.9 | 0.8 |
| *Acyrthosiphon pisum* | 72 | 6.1 | 15 | 12 |
| *Cimex lectularius* | 78 | 9.7 | 1.4 | 7.4 |
| *Gerris buenoi* | 85.4 | 1.3 | 12.3 | 2.3 |

Table S2 : BUSCO Genome assessment based on percentage of BUSCO genes identified (<ftp://cegg.unige.ch/OrthoDB7/BUSCO/README.txt>). Species results other than *Gerris buenoi* extracted from supplementary data in [81].

| **Gene** | **Scaffold: start..end** | **Locus length (nt)** | **Protein length (aa)** | **Number of CDS exons** |
| --- | --- | --- | --- | --- |
| *labial -part 1 of 2* | Scaffold2148:49081..49463 | 383  (partial) | 208  (concat-enated) | 2  (concat-enated) |
| *labial -part 2 of 2* | Scaffold688:18951..20594 | 1 644  (partial) |  |  |
| *proboscipedia* | Scaffold917:82996..178853  - strand | 95 858 | 498 | 3 |
| *zerknüllt* | Scaffold917:254614..264809  + strand | 10 196 | 360 | 3 |
| *Deformed* | Scaffold927:71079..127936 | 56 858 | 339 | 2 |
| *Sex combs reduced* | Scaffold111:113209..227662  - strand | 114 454 | 279 | 2 |
| *fushi tarazu* | Scaffold111:292364..296153  - strand | 3 790 | 298 | 2 |
| *Antennapedia* | Scaffold111:608939..620195  - strand | 11 257 | 284 | 2 |
| *Ultrabithorax** | Scaffold280:506616..507456 | 841  (partial) | 178  (partial) | 1  (partial) |
| *abdominal-A* | Scaffold259:352274..461324 | 109 051 | 320 | 3 |
| *Abdominal-B** | Scaffold464:255292..399249 | 143 958 | 254  (partial) | 2  (partial) |
| *iroquois* | Scaffold451:304431-432356 | 127 926 | 426 | 6 |
| *mirror* | Scaffold2206:85783-151112 | 65 330 | 362 | 5 |

Table S3 : Positional information for the annotated homeobox genes. Incomplete gene models are marked with an asterisk (*). Colored shading highlights gene linkage, and coding strand is also indicated for these gene models.

| Gene name | Gene abbreviation | *Gerris buenoi* | *Oncopeltus fasciatus* | *Cimex lectularius* |
| --- | --- | --- | --- | --- |
| *abrupt* | *Ab* | Yes | Yes | Yes |
| *Achaete-scute complex* | *Ac* | Yes | No | No |
| *Actin 5C* | *Act5C* | Yes | Yes | Yes |
| *amphiphysin* | *Amph* | Yes | Yes | Yes |
| *aralar1* | *aralar1* | Yes | Yes | Yes |
| *arrow* | *arr* | Yes | Yes | Yes |
| *Asense* | *ase* | No | Yes | No |
| *astray* | *aay* | Yes | Yes | Yes |
| *bantam* | *ban* | No | No | No |
| *beadex* | *Bx* | Yes | Yes | Yes |
| *bendless* | *ben* | Yes | Yes | Yes |
| *bifocal* | *bif* | Yes | No | No |
| *bonus* | *bon* | Yes | No | Yes |
| *buttonless* | *btn* | No | No | No |
| *calreticulin* | *Crc* | Yes | Yes | Yes |
| *capricious* | *caps* | Yes | Yes | Yes |
| *caupolican* | *caup* | No | Yes | Yes |
| *center divider* | *cdi* | Yes | Yes | Yes |
| *cornetto* | *corn* | No | Yes | No |
| *corto* | *corto* | No | No | No |
| *couch potato* | *cpo* | Yes | Yes | Yes |
| *crooked legs* | *crol* | No | Yes | Yes |
| *dacapo* | *dap* | No | Yes | No |
| *dalmatian* | *dmt* | No | No | No |
| *Darkener of apricot* | *Doa* | No | Yes | Yes |
| *daughterless* | *da* | Yes | Yes | No |
| *deadpan* | *dpn* | Yes | Yes | Yes |
| *Delta* | *Dl* | No | Yes | Yes |
| *diminutive* | *dm* | No | No | No |
| *division abnormally delayed* | *dally* | No | Yes | Yes |
| *dorsotonals (homothorax)* | *hth* | No | Yes | Yes |
| *E(spl) region transcript m7* | *E(spl)m7-HLH* | Yes | Yes | Yes |
| *E2F transcription factor* | *E2f* | Yes | Yes | Yes |
| *Eb1* | *Eb1* | Yes | Yes | Yes |
| *effete* | *eff* | Yes | No | No |
| *egghead* | *egh* | Yes | Yes | Yes |
| *enabled* | *ena* | Yes | Yes | Yes |
| *Enhancer-of-split* | *E(spl)m8-HLH* | No | Yes | Yes |
| *EP2237 (cabut)* | *cbt* | Yes | No | Yes |
| *escargot* | *esg* | Yes | No | Yes |
| *extra macrochaetae* | *emc* | Yes | Yes | Yes |
| *flightless* | *fliI* | Yes | Yes | Yes |
| *frizzled* | *fz* | Yes | Yes | Yes |
| *frizzled 2* | *fz2* | Yes | Yes | Yes |
| *ftz transcription factor 1* | *ftz-f1* | No | Yes | Yes |
| *gliolectin* | *glec* | No | No | No |
| *gliotactin* | *Gli* | No | No | Yes |
| *Glutathione S transferase 2* | *GstS1* | Yes | Yes | Yes |
| *grapes* | *grp* | Yes | Yes | Yes |
| *groucho* | *gro* | Yes | Yes | Yes |
| *Hairless* | *H* | Yes | Yes | Yes |
| *hairy* | *h* | Yes | Yes | Yes |
| *headcase* | *hdc* | Yes | Yes | Yes |
| *hephaestus* | *heph* | Yes | Yes | Yes |
| *Hormone receptor-like in 39* | *Hr39* | No | Yes | Yes |
| *IGF-II mRNA-binding protein* | *Imp* | Yes | Yes | Yes |
| *kekkon-1* | *kek1* | Yes | Yes | Yes |
| *kuzbanian* | *kuz* | Yes | Yes | Yes |
| *Laminin A* | *LanA* | Yes | Yes | Yes |
| *lethal (1) G0007* | *l(1)G0007* | Yes | Yes | Yes |
| *liquid facets* | *lqf* | Yes | Yes | Yes |
| *lola like* | *lolal* | Yes | Yes | Yes |
| *longitudinals lacking* | *lola* | Yes | Yes | Yes |
| *melted* | *melt* | Yes | Yes | Yes |
| *mushroom body defect* | *mud* | No | No | No |
| *nebbish* | *neb* | Yes | Yes | No |
| *nejire* | *nej* | Yes | Yes | Yes |
| *neuralized* | *neur* | Yes | Yes | Yes |
| *notch* | *N* | Yes | Yes | Yes |
| *nuclear fallout* | *nuf* | No | No | No |
| *pavarotti* | *pav* | Yes | Yes | Yes |
| *pebble* | *pbl* | Yes | Yes | Yes |
| *pipsqueak* | *psq* | Yes | Yes | Yes |
| *pointed* | *pnt* | Yes | Yes | Yes |
| *Poly(ADP-ribose) glycohydrolase* | *Parg* | Yes | Yes | Yes |
| *polychaetoid* | *pyd* | Yes | Yes | Yes |
| *prospero* | *pros* | Yes | Yes | Yes |
| *Protein kinase 61C* | *Pdk1* | Yes | Yes | Yes |
| *Protein tyrosine phosphatase 10D* | *Ptp10D* | Yes | Yes | Yes |
| *pumilio* | *pum* | Yes | Yes | Yes |
| *pxb* | *pxb* | No | No | No |
| *quemao* | *qm* | Yes | Yes | Yes |
| *Ras oncogene at 85D* | *Ras85D* | No | Yes | Yes |
| *Ras-like protein A* | *Rala* | Yes | No | No |
| *raspberry* | *ras* | Yes | Yes | Yes |
| *Rhomboid* | *rho* | Yes | Yes | Yes |
| *Ribosomal protein S5* | *RpS5a* | Yes | Yes | Yes |
| *roundabout* | *robo* | Yes | Yes | Yes |
| *rutabaga* | *rut* | No | Yes | Yes |
| *sanpodo* | *spdo* | Yes | Yes | Yes |
| *scabrous* | *sca* | Yes | Yes | Yes |
| *scalloped* | *sd* | Yes | Yes | Yes |
| *scratch* | *scrt* | Yes | Yes | No |
| *scribbled* | *scrib* | Yes | Yes | Yes |
| *scribbler* | *sbb* | Yes | Yes | Yes |
| *scute* | *sc* | No | Yes | Yes |
| *seven up* | *svp* | Yes | Yes | Yes |
| *shaggy* | *sgg* | Yes | Yes | Yes |
| *singed* | *sn* | Yes | Yes | Yes |
| *smooth* | *sm* | Yes | Yes | Yes |
| *Sp1* | *Sp1* | No | Yes | No |
| *SP71 (Trynity)* | *Tyn* | Yes | Yes | Yes |
| *spitz* | *spi* | No | No | No |
| *split ends* | *spen* | Yes | Yes | Yes |
| *string* | *stg* | Yes | Yes | Yes |
| *sugarless* | *sgl* | Yes | Yes | Yes |
| *taranis* | *tara* | Yes | Yes | Yes |
| *Tcp-1eta* | *Tcp-1eta* | Yes | Yes | Yes |
| *Tollo* | *Tollo* | Yes | Yes | Yes |
| *tout-velu* | *ttv* | No | Yes | Yes |
| *tramtrack* | *ttk* | Yes | Yes | Yes |
| *Trehalose receptor 1 (Trapped in endoderm 1)* | *Tre1* | No | Yes | No |
| *tribbles* | *trbl* | Yes | Yes | Yes |
| *tweety* | *tty* | Yes | Yes | Yes |
| *Twin of m4* | *Tom* | No | No | No |
| *u-turn (ventral veins lacking)* | *wl* | Yes | Yes | Yes |
| *Ubiquitin activating enzyme 1* | *Uba1* | Yes | Yes | Yes |
| *Ubiquitin conjugating enzyme 2* | *UbcD2* | Yes | Yes | No |
| *Vacuolar H+ ATPase 16kD subunit* | *Vha16-1* | Yes | Yes | Yes |
| *β-amyloid protein precursor-like* | *Appl* | Yes | Yes | Yes |

Table S4 : Annotation of genes involved in bristle number and neural development based on *Drosophila melanogaster* quantitative analyses [199].

| **Species** | **Order** | **Suborder** | **LWS** | **SWS-B** | **SWS-UV** | **Rh7** | **Arthropsin** | **c-Opsin** |
| --- | --- | --- | --- | --- | --- | --- | --- | --- |
| *Gerris buenoi* | Hemiptera | Heteroptera | 4 | - | 1 | 1 | 1 | 1 |
| *Cimex lectularius* | Hemiptera | Heteroptera | 1 | - | 1 | 1 | - | 1 |
| *Rhodnius prolixus* | Hemiptera | Heteroptera | 1 | - | 1 | 1 | - | 1 |
| *Acyrthosiphon pisum* | Hemiptera | Sternorrhyncha | 1 | - | 2 | 4 | 1 | 1 |
| *Megoura viciae* | Hemiptera | Sternorrhyncha | 1 | - | 1 | na | na | na |
| *Nephotettix cincticeps* | Hemiptera | Auchenorryhncha | 1 | 1 | 1 | na | na | na |

Table S5 : Opsin conservation in Hemiptera. [80, 81, 200-202]

| **Species** | **CPR_RR-1** | **CPR_RR-2** | **CPR_Uncl** | **CPAP1** | **CPAP3** | **CPF** | **TWDL** | **Total** |
| --- | --- | --- | --- | --- | --- | --- | --- | --- |
| *Drosophila melanogaster* | 61 | 42 | 34 | 29 | 10 | 5 | 29 | 210 |
| *Glossina* *morsitans* | 33 | 27 | 17 | 11 | 6 | 1 | 9 | 104 |
| *Culex quinquefasciatus* | 49 | 97 | 30 | 10 | 8 | 5 | 9 | 208 |
| *Aedes aegypti* | 66 | 150 | 28 | 14 | 9 | 3 | 6 | 276 |
| *Anopheles gambiae* | 43 | 103 | 21 | 13 | 10 | 4 | 12 | 206 |
| *Bombyx mori* | 47 | 78 | 19 | 13 | 6 | 1 | 4 | 168 |
| *Danaus plexippus* | 47 | 57 | 18 | 16 | 10 | 1 | 5 | 154 |
| *Apis mellifera* | 13 | 15 | 10 | 15 | 7 | 4 | 2 | 66 |
| *Nasonia vitripennis* | 19 | 32 | 18 | 16 | 6 | 5 | 2 | 98 |
| *Pediculus humanus* | 9 | 15 | 17 | 12 | 6 | 0 | 2 | 61 |
| *Daphnia pulex* | 101 | 36 | 152 | 20 | 12 | 0 | 0 | 321 |
| *Tetranychus urticae* | 0 | 7 | 31 | 14 | 5 | 0 | 0 | 57 |
| *Tribolium castaneum* | 34 | 55 | 21 | 13 | 7 | 5 | 3 | 138 |
| *Acyrthosiphon pisum* | 9 | 84 | 20 | 10 | 8 | 2 | 3 | 136 |
| *Cimex lectularius* | 18 | 70 | 32 | 15 | 6 | 5 | 3 | 149 |
| *Gerris buenoi* | 22 | 74 | 30 | 10 | 6 | 3 | 10 | 155 |

Table S6 : Detection and classification of putative structural cuticular proteins. Information from other species than *Gerris* *buenoi* adapted from Ioannidou, et al. [187] and Benoit, et al. [81].

|  | **Scaffold #** | **# Genes** | **Family** | **Length (Kbp)** | **Density (Kbp/gene)** |
| --- | --- | --- | --- | --- | --- |
| 1 | 431 | 14 | CPR RR-1/CPR Uncl | 398 | 28.4 |
| 2 | 32 | 13 | CPR RR-2 | 183 | 14.1 |
| 3 | 41 | 9 | CPR RR-2 | 92 | 10.2 |
| 4 | 349 | 8 | CPR RR-2 | 224 | 27.9 |
| 5 | 996 | 6 | CPR RR-2 | 73 | 12.2 |
| 6 | 683 | 4 | CPAP3 | 250 | 62.5 |
| 7 | 2496 | 4 | CPR RR-2/CPR Uncl | 92 | 23.0 |
| 8 | 46 | 3 | CPF | 49 | 16.2 |
| 9 | 80 | 3 | TWDL | 62 | 20.6 |
| 10 | 132 | 3 | CPR Uncl | 249 | 83.1 |
| 11 | 706 | 3 | CPR Uncl | 66 | 21.9 |

Table S7 : Clusters of genes coding cuticle proteins in the genome of *Gerris* *buenoi*

|  | **Ionotropic** | **Gustatory** | **Odorant** |
| --- | --- | --- | --- |
| *Gerris buenoi* | 45/45 | 60/135 | 153/155 |
| *Oncopeltus fasciatus* | 37/37 | 115/169 | 120/121 |
| *Rhodnius prolixus* | 33/33 | 28/30 | 116/116 |
| *Cimex lectularius* | 30/30 | 24/36 | 48/49 |
| *Drosophila melanogaster* | 65/65 | 60/68 | 60/62 |

Table S8 : Numbers of genes and encoded proteins in three chemoreceptor families in heteropterans with genome sequences, and *Drosophila melanogaster* for comparison.

| Order | Hemiptera | | | Diptera | Hymenoptera | Coleoptera | Lepidoptera |
| --- | --- | --- | --- | --- | --- | --- | --- |
| Species | *Gerris buenoi* | *Rhodnius prolixus* | *Nilaparvata lugens* | *Drosophila melanogaster* | *Apis mellifera* | *Tribolium castaneum* | *Bombyx mori* |
| Clan 2 | 6 | 5 | 10 | 6 | 8 | 8 | 10 |
| Clan 3 | 62 | 50 | 19 | 36 | 28 | 70 | 36 |
| Clan 4 | 25 | 27 | 27 | 32 | 4 | 44 | 32 |
| Clan mito | 10 | 6 | 12 | 11 | 6 | 9 | 8 |
| Total P450 | 103 | 88 | 68 | 85 | 46 | 131 | 86 |

Table S9: Numbers of cytochrome P450 genes annotated in some selected insect genomes and their distribution across P450 clans. Data are taken from [107, 108, 203-205], and from a CYP450 database [206].

| **Gene name** | **OGS name** | **Genomic scaffold** | **Length (aa)** | **Remark** |
| --- | --- | --- | --- | --- |
| UGT-01 | GBUE014547-RA | Scaffold1506 | 530 | complete |
| UGT-02 | GBUE015333-RA | Scaffold1907 | 515 | complete |
| UGT-03 | GBUE018966-RA | Scaffold3228 | 533 | complete |
| UGT-04 | GBUE018967-RA | Scaffold3228 | 515 | complete |
| UGT-05 | GBUE018968-RA | Scaffold3228 | 543 | complete |
| UGT-06 | GBUE014164-RA | Scaffold2126 | 524 | complete |
| UGT-07 | GBUE014165-RA | Scaffold2126 | 527 | complete |
| UGT-08 | GBUE013499-RA-1 | Scaffold1323 | 529 | complete |
| UGT-09 | GBUE013499-RA-2 | Scaffold1323 | 512 | complete |
| UGT-10 | GBUE013499-RA-3 | Scaffold1323 | 527 | complete |
| UGT-11 | GBUE013500-RA | Scaffold1323 | 218 | partial |
| UGT-12p* | GBUE019125-RA | Scaffold3054 | 470 | partial |
| UGT-13 | GBUE010586-RA | Scaffold838 | 524 | complete |
| UGT-14 | GBUE012986-RA | Scaffold1320 | 697 | complete |
| UGT-15 | GBUE013062-RA | Scaffold1042 | 437 | partial |
| UGT-16 | GBUE020555-RA | Scaffold5464 | 326 | partial |
| UGT-17p | GBUE020560-RA | Scaffold6284 | 243 | partial |
| UGT-18 | GBUE012772-RA | Scaffold1549 | 422 | partial |
| UGT-19 | GBUE012773-RA | Scaffold1549 | 347 | partial |
| UGT-20 | GBUE012774-RA | Scaffold1549 | 434 | partial |
| UGT-21 | GBUE012775-RA | Scaffold1549 | 201 | partial |
| UGT-22 | GBUE012776-RA | Scaffold1549 | 378 | partial |
| UGT-23 | GBUE012777-RA | Scaffold1549 | 522 | complete |
| UGT-24 | GBUE012778-RA | Scaffold1549 | 540 | complete |
| UGT-25 | GBUE012779-RA | Scaffold1549 | 519 | complete |
| UGT-26 | GBUE012780-RA | Scaffold1549 | 534 | complete |
| UGT-27 | GBUE012781-RA | Scaffold1549 | 529 | complete |
| UGT-28 | no OGS name | Scaffold4983 | 235 | partial |

Table S10 : List of UDP-glycosyltransferase genes in *Gerris buenoi* genome. (*refers to pseudogene.)

| **Gene type** | ***Gene name*** | **Location [Accession#]** | **Protein Length** | **Domains** |
| --- | --- | --- | --- | --- |
| ***Gap*** | *orthodenticle* | Scaffold177:468498-481641 + strand  GbueTmpM005873-RA | 542 | zinc finger C2H2 |
|  | *buttonhead* | Scaffold1076:201924-220125 + strand  GbueTmpM009254-RA | 437 | zinc finger M2C2 |
|  | *collier* | Scaffold128:650434 - 661155 + strand  GbueTmpM003852-RA  GbueTmpM003853-RA | 236 | IPT Superfamily |
|  | *cap-n-collar* | Scaffold1737:125742 - 180215 + strand  GbueTmpA013482-RA | 414 | bZIP Superfamily |
|  | *crocodile* | Scaffold417:94048 -94890 – strand  GbueTmpA005876-RA | 280 | Forkhead Superfamily |
|  | *Krüppel* | Scaffold66:273659 - 274706 + strand  GbueTmpA001375-RA | 246 | zinc finger M2C2 |
|  | *huckebein* | Scaffold1050:35145-36625 + strand  GbueTmpA011673-RA | 153 | zinc finger C2H2 |
|  | *empty spiracles* | Scaffol640:42899 - 108829 + strand  GbueTmpA010166-RA  GbueTmpA010167-RA  GbueTmpA010168-RA | 237 | Homeobox Superfamily |
|  | *giant* | Scaffold1313:205754 - 259477 – strand  GbueTmpM012482-RA | 290 | bZIP Superfamily |
|  | *gomdanji* | Scaffold177:546605-551844 + strand  GbueTmpM005874-RA | 101 | Δ meth_res Superfamily |
| ***Segment***  ***polarity*** | *shifted* | Scaffold4383:9065-11975 + strand | 268 | WIF Superfamily |
|  | *roadkill* | Scaffold7:1346380-1347546 + strand | 388 | MATH superfamily  BTB Domain |
|  | *perli-like* | Scaffold542:114112-120587 – strand  GbueTmpM009219-RA | 214 | Perli Domain |
|  | *microtubule star* | Scaffold83:875664-876641 + strand | 325 | MPP Superfamily |
|  | *flapwing* | Scaffold362:255357-260899 - strand | 240 | MPP Superfamily |
|  | *cullin1* | Scaffold15:198529-200865 - strand | 778 | Cullin Superfamily |
|  | *dispatched* | Scaffold2487:39972-52586 - strand | 434 | ND |
|  | *costa* | Scaffold666:175038-178490 + strand | 1150 | Kinesin Domain |
|  | *paxillin* | Scaffold927:228836-245479 - strand | 300 | LIM Superfamily |
| ***Terminal patterning*** | *Torso* | Scaffold626:104429-114052 + strand  GbueTmpA010687-RA | 412 | PKc_like superfamily  FN3 superfamily |
|  | *Torso-like* | Scaffold7:1642089-1661652 + strand | 356 | MACPF  Superfamily |
| ***General*** | *decapentaplegic* | Scaffold488:247243-262588 – strand  GbueTmpA009289-RA | 323 | TGF-Beta Domain |
|  | *cubitus interruptus* | Scaffold2762:18072-42310 – strand  GbueTmpA017830-RA | 960 | zinc finger-H |
|  | *lipophorin-like* | Scaffold940:71827-78984 + strand  GbueTmp8010317-RA | 1202 | DUF1943 Superfamily |

Table S11 : Current Early Developmental Genes identified in the *Gerris buenoi* genome. The table lists Gap Genes and Segment Polarity Genes models, model location and accession number, protein length, and protein domain identified in the model.

| ***Gerris buenoi early patterning genes*** | |
| --- | --- |
| ***Gap Genes*** | |
| *caudal* | ? |
| *hunchback* | Yes |
| *orthodenticle* | Yes |
| *buttonhead* | Yes |
| *collier* | Yes |
| *cap-n-collar* | Yes |
| *crocodile* | Yes |
| *Krüppel* | Yes |
| *huckebein* | Yes |
| *sloppy-paired* | Yes |
| *empty spiracles* | Yes |
| *giant* | Yes |
| *knirps* | Yes |
| *tailless* | Yes |
| *gomdanji* | Yes |
| ***Pair Rule Genes*** | |
| *even-skipped* | Yes |
| *paired* | Yes |
| *odd-skipped* | Yes |
| *paired* | Yes |
| *runt* | Yes |
| *hairy* | Yes |
| *Tenascin major* | Yes |
| *sister-of-odd-and-bowl* | Yes |
| ***Segment Polarity Genes*** | |
| *engrailed* | Yes |
| *invected* | Yes |
| *shifted* | Yes |
| *roadkill* | Yes |
| *peril-like* | Yes |
| *patched* | Yes |
| *nejire* | Yes |
| *microtubule star* | Yes |
| *flapwing* | Yes |
| *cullin1* | Yes |
| *dispatched* | Yes |
| *costa* | Yes |
| *paxillin* | Yes |
| ***Terminal Patterning Genes*** | |
| *torso* | Yes |
| *PTTH* | ? |
| *torso-like* | Yes |
| *trunk* | No |

Table S12 : Presence/absence of *Drosophila melanogaster* early patterning genes in the genomes of *Gerris buenoi.*

| **Gene type** | **Gene name** | ***Drosophila melanogaster*** | | ***Tribolium castaneum*** | |
| --- | --- | --- | --- | --- | --- |
|  |  | **QC (ID)** | **Bit Score** | **QC (ID)** | **Bit Score** |
| ***Gap*** | *orthodenticle* | 24% (98%) | 94 | 42% (71%) | 97 |
|  | *buttonhead* | 26% (61%) | 145 | 29% (70%) | 187 |
|  | *collier* | 43% (70%) | 94 | 47% (62%) | 90 |
|  | *cap-n-collar* | 19% (43%) | 47 | 56% (34%) | 116 |
|  | *crocodile* | 81% (52%) | 234 | 39% (65%) | 166 |
|  | *Krüppel* | 70% (71%) | 242 | 77% (59%) | 240 |
|  | *huckebein* | 71% (68%) | 174 | ND | ND |
|  | *empty spiracles* | 86% (71%) | 179 | 90% (60%) | 281 |
|  | *giant* | 33% (62%) | 77 | 40% (48%) | 117 |
|  | *gomdanji* | 64% (34%) | 45 | ND | ND |
| ***Segment***  ***polarity*** | *shifted* | 95% (55%) | 286 | 92% (68%) | 350 |
|  | *roadkill* | 96% (56%) | 424 | 96% (57%) | 437 |
|  | *peril-like* | 77% (56%) | 194 | 96% (61%) | 262 |
|  | *microtubule star* | 84% (50%) | 295 | ND | ND |
|  | *flapwing* | 87% (42%) | 184 | ND | ND |
|  | *cullin1* | 99% (61%) | 956 | 100% (79%) | 1274 |
|  | *dispatched* | 98% (25%) | 181 | 96% (31%) | 181 |
|  | *costa* | 72% (36%) | 227 | 70% (27%) | 159 |
|  | *paxillin* | 89% (65%) | 367 | 81% (66%) | 330 |
| ***Terminal patterning*** | *Torso* | 93% (53%) | 140 | 92% (31%) | 194 |
|  | *Torso-like* | 90% (46%) | 321 | 89% (49%) | 335 |
| ***General*** | *decapentaplegic* | 55% (34%) | 173 | 58% (39%) | 295 |
|  | *cubitus interruptus* | 94% (46%) | 301 | 98% (42%) | 280 |
|  | *lipophorin-like* | 77% (23%) | 215 | 95% (33%) | 587 |

Table S13 : Represents Query Coverage (Identity) and E-value of the annotated gene models pairwise aligned to orthologues in other species. Pairwise alignment was performed using NCBI blast. ND – Not Determined.

| **Gene** | **Scaffold: start..end** | **Locus length (nt)** | **Protein length (aa)** | **Number of CDS exons** |
| --- | --- | --- | --- | --- |
| *axin* | Scaffold136:602832..659508 | 56 677 | 1496 | 16 |
| *armadillo** | Scaffold2236:76533..96972 | 20 440  (partial) | 716  (partial) | 11 |
| *arrow* | Scaffold136:139587..222403 | 82 817 | 1490 | 24 |
| *dishevelled -RA* | Scaffold441:78333..107479 | 29 147 | 602 | 15 |
| *dishevelled -RB* | Scaffold441:78333..124793 | 46 461 | 597 | 14 |
| *frizzled* | Scaffold288:270554..271759 | 1 206 | 401 | 1 |
| *frizzled-2* | Scaffold1053:141773..145781 | 4 009 | 597 | 1 |
| *frizzled-3* | Scaffold304:383672..482292 | 98 621 | 500 | 2 |
| *glycogen synthase kinase-3 beta -RA -part 1 of 2** | Scaffold1391:148463..174822 | 26 360  (partial) | 302  (partial) | 6 |
| *glycogen synthase kinase-3 beta -RB -part 1 of 2** | Scaffold1391:148463..174822 | 26 360  (partial) | 286  (partial) | 6 |
| *glycogen synthase kinase-3 beta -part 2 of 2** | Scaffold10229:2..3044 | 3 043  (partial) | 150  (partial) | 2 |
| *wingless* | Scaffold2771:12925..70979 | 58 055 | 331 | 3 |
| *Wnt7* | Scaffold163:273675..338565 | 64 891 | 456 | 10 |
| *Wnt8* | Scaffold1136:57077..65015 | 7 939 | 302 | 5 |
| *Wnt5* | Scaffold3063:28070..66680 | 38 611 | 321 | 6 |
| *Wnt10* | Scaffold2796:27374..49167 | 21 794 | 273 | 5 |
| *WntA** | Scaffold20: 632685..638039 | 5 355  (partial) | 287  (partial) | 5 |
| *wntless* | Scaffold190:240723..250315 | 9 593 | 538 | 11 |

Table S14 : Positional information for the 18 Wnt signaling genes annotated. Incomplete gene models are marked with an asterisk (*).

| **Gene set** | **Gene name** | ***Gerris buenoi***  **CpG_O/E_ value** | ***Rhodnius proxilus***  **CpG_O/E_ value** |
| --- | --- | --- | --- |
| Insulin signalling | *Chico* | 0.658374618 |  |
| Insulin signalling | *forkhead box protein O* |  |  |
| Insulin signalling | *Foxo* | 1.014152563 | 0.963514594 |
| Insulin signalling | *Insulin receptor 1* | 1.090538511 | 1.133882478 |
| Insulin signalling | *Insulin receptor 1-like* | 0.865210624 |  |
| Insulin signalling | *Insulin receptor 2* | 0.394382326 | 0.781348977 |
| Insulin signalling | *Insulin receptor substrate* |  |  |
| Insulin signalling | *Phosphatase and tensine homologue* | 0.444946289 |  |
| Insulin signalling | *Phosphoinositide 3-kinase Pi3K21B* | 0.730078776 | 0.783423219 |
| Insulin signalling | *Phosphoinositide 3-kinase Pi3K92E* | 0.438681484 | 0.575389176 |
| Insulin signalling | *Protein Kinase B* | 0.395861448 | 0.563182964 |
| Insulin signalling | *Rheb/Ras homolog enriched in brain* | 0.540547798 |  |
| Insulin signalling | *RPS6-p70-protein kinase* | 0.731629717 | 0.704464786 |
| Insulin signalling | *Slimfast* | 0.77679356 | 0.7171875 |
| Insulin signalling | *Target of rapamycin* | 0.648267284 | 0.641665967 |
| Insulin signalling | *Thor* | 0.910084034 | 0.907818533 |
| Insulin signalling | *Tsc1 Tuberous sclerosis complex 1* | 0.383532463 | 0.517120208 |
| Insulin signalling | *Tsc2/gigas/Tuberin* | 0.580956324 | 0.815878378 |
| Juvenile Hormone | *Allostatin C* |  |  |
| Juvenile Hormone | *broad* | 0.905797101 | 0.973075749 |
| Juvenile Hormone | *Chd64* |  |  |
| Juvenile Hormone | *FK506-binding protein 1* | 0.480397835 | 0.498673415 |
| Juvenile Hormone | *FK506-binding protein 14 ortholog* |  |  |
| Juvenile Hormone | *FK506-binding protein FKBP59* | 0.553441364 | 0.759341109 |
| Juvenile Hormone | *Juvenile hormone acid methyltransferase* | 0.853085106 | 0.627682228 |
| Juvenile Hormone | *Juvenile hormone epoxide hydrolase 1* | 0.497504096 | 0.823006391 |
| Juvenile Hormone | *Juvenile hormone esterase* |  |  |
| Juvenile Hormone | *Juvenile hormone esterase duplication* |  |  |
| Juvenile Hormone | *Juvenile hormone-inducible protein 1* | 0.437671182 | 0.579799692 |
| Juvenile Hormone | *Juvenile hormone-inducible protein 26* | 0.90600823 | 0.302261307 |
| Juvenile Hormone | *Kruppel homolog 1* | 0.883615819 | 1.019771301 |
| Juvenile Hormone | *Methoprene-tolerant* | 0.633364098 | 0.59144385 |
| Juvenile Hormone | *taiman* |  |  |
| Reproduction | *Armitage* | 0.900408271 | 0.735040693 |
| Reproduction | *Aubergine (annotated as Piwi-like)* | 0.497755107 |  |
| Reproduction | *Bazooka/PAR-3* |  | 0.68762606 |
| Reproduction | *cappuccino* |  |  |
| Reproduction | *capsuleen* |  |  |
| Reproduction | *Dynein light chain 90F* | 1.006892418 | 0.784722222 |
| Reproduction | *eIF5B* | 0.66963049 | 0.699717583 |
| Reproduction | *Heat shock protein 83/90* | 0.661795474 | 0.78564613 |
| Reproduction | *Heat shock protein 83/90 2* |  | 0.558785904 |
| Reproduction | *Hunchback* | 1.00601711 | 0.902307812 |
| Reproduction | *Laminin A* |  |  |
| Reproduction | *Laminin B2* |  |  |
| Reproduction | *loki/Chk2* |  |  |
| Reproduction | *maelstrom* |  |  |
| Reproduction | *meiotic 41/ATR* | 0.318670549 |  |
| Reproduction | *Merlin* |  |  |
| Reproduction | *Moesin* |  |  |
| Reproduction | *nanos* | 0.392635135 |  |
| Reproduction | *N-ethylmaleimide-sensitive factor 2* |  |  |
| Reproduction | *Par - 6* | 0.493019601 | 0.75739645 |
| Reproduction | *Par-1* |  |  |
| Reproduction | *pebble/ECT2* | 0.346433041 | 0.556323529 |
| Reproduction | *Piwi (annotated as piwi-like)* |  |  |
| Reproduction | *Rab11* | 1.21100186 | 0.891789661 |
| Reproduction | *sevenless* |  |  |
| Reproduction | *Smaug* |  | 0.374331551 |
| Reproduction | *Spindle-D* |  |  |
| Reproduction | *Spindle-E* |  |  |
| Reproduction | *staufen* | 0.335958039 | 0.63898769 |
| Reproduction | *Stellate* |  |  |
| Reproduction | *telomere fusion* |  |  |
| Reproduction | *tudor* | 1.249130153 | 0.799734986 |
| Reproduction | *vasa* | 0.693071093 |  |
| Wing | *Acetylcholine esterase* | 1.056863669 | 0.931578947 |
| Wing | *apterous* |  |  |
| Wing | *argos* |  |  |
| Wing | *armadillo* | 0.404645677 | 0.517999969 |
| Wing | *baboon* | 0.516144578 | 0.667751211 |
| Wing | *basket* | 0.740959251 | 0.7426405 |
| Wing | *bifid* |  |  |
| Wing | *blistered* |  |  |
| Wing | *brinker* | 0.876838162 | 0.574162679 |
| Wing | *Buffy* | 0.602699055 |  |
| Wing | *capricious* | 0.914409241 | 0.819466248 |
| Wing | *clot* | 0.872160934 | 1.03902439 |
| Wing | *cut* | 0.362195409 | 0.754880803 |
| Wing | *Death regulator Nedd2-like caspase* | 1.146718147 |  |
| Wing | *Death related ICE-like caspase* | 0.6890625 | 0.804121212 |
| Wing | *Death-associated inhibitor of apoptosis 1* |  |  |
| Wing | *Decapping protein 1* | 1.189357953 | 0.711444547 |
| Wing | *division abnormally delayed* | 0.535155846 | 0.513011152 |
| Wing | *eiger* | 0.838224085 | 0.839430894 |
| Wing | *engrailed* | 1.247013856 | 0.814175728 |
| Wing | *Epidermal growth factor receptor* | 0.697416093 | 0.659715546 |
| Wing | *fringe* | 1.294816794 | 0.638368984 |
| Wing | *hedgehog* | 0.964415584 | 0.773176471 |
| Wing | *Keren* | 0.754096776 |  |
| Wing | *Mad1* | 0.517751479 |  |
| Wing | *Mad2* | 0.414863782 | 0.499577603 |
| Wing | *Mad3* | 0.440286166 | 0.555261005 |
| Wing | *mastermind* | 0.516834008 | 0.690080382 |
| Wing | *Medea* | 0.497130418 | 0.534404253 |
| Wing | *mind bomb 1* | 0.533591731 | 0.771083019 |
| Wing | *nemo* | 1.09630137 | 0.58400637 |
| Wing | *Nipped-A* |  |  |
| Wing | *patched* | 0.475015567 | 0.732220161 |
| Wing | *punt* | 0.990559836 | 0.428825279 |
| Wing | *punt 2* | 0.451908397 |  |
| Wing | *Ras oncogene at 85D* | 1.040664452 | 0.743847875 |
| Wing | *saxophone* | 0.949921557 |  |
| Wing | *schnurri* | 0.347452969 |  |
| Wing | *Serrate* |  |  |
| Wing | *smoothened* | 0.431910569 |  |
| Wing | *spalt major* | 0.925619236 | 0.699655862 |
| Wing | *Star* | 0.658335154 |  |
| Wing | *Suppressor of Hairless* | 0.542231327 | 0.624452765 |
| Wing | *tartan* | 0.732986444 | 1.144366197 |
| Wing | *thickveins* | 0.586962236 |  |
| Wing | *wingless* | 1.124115983 | 1.017095821 |

Table S15 : List of genes in the networks underlying wing polyphenism, reproduction, juvenile hormone, and insulin signalling included in the analysis and their CpG_O/E_ value for *Gerris* *buenoi* and *Rhodnius* *prolixus*. Genes that were annotated in *Gerris* *buenoi* but excluded from the analysis because they did have a complete codding sequence are also listed but without a CpG_O/E_ value.

|  |  | Core histones | | | |
| --- | --- | --- | --- | --- | --- |
|  | H1 | H2A | H2B | H3 | H4 |
| *Aedes aegypti* | 6 | 19 | 11 | 18 | 15 |
| *Apis mellifera* | 2 | 6 | 5 | 6 | 4 |
| *Acyrthosiphon pisum* | 6 | 5 | 5 | 7 | 5 |
| *Oncopeltus fasciatus* | 1 | 3 | 4 | 3 | 2 |
| *Cimex lectularius* | 4 | 14 | 6 | 13 | 8 |
| ***Gerris buenoi*** | **10** | **11** | **9** | **10** | **9** |
| *Daphnia pulex* | 5 | 10 | 12 | 10 | 6 |
| *Tetranychus urticae* | 1 | 4 | 7 | 6 | 3 |
| *Ixodes scapularis* | 4 | 6 | 4 | 4 | 1 |
| *Strigamia maritima* | 3 | 7 | 15 | 4 | 4 |

Table S16 : Number of loci within the genomes of arthropod species encoding the five classes of histones. Orthologs for *Aedes aegypti*, *Daphnia pulex*, *Tetranychus urticae* and *Ixodes scapularis* were obtained by BLAST analysis. Orthologs for *Apis mellifera* and *Acyrthosiphon pisum* were obtained from published literature [162, 207]. Orthologs for *Oncopeltus fasciatus* (manuscript in preparation) and *Cimex lectularius* [81] were obtained during genome annotation.

| **Species** | **Number of antioxidant genes** |
| --- | --- |
| *Acyrthosiphon pisum* | 6 |
| *Apis mellifera* | 1 |
| *Bombyx mori* | 2 |
| *Cimex lectularis* | 16 |
| *Drosophila melanogaster* | 0 |
| *Pediculus humanus* | 0 |
| *Tribolium castaneum* | 5 |

Table S17 : Number of genes for each species compared to that had highest similarity to *G. Buenoi* antioxidant genes.

| Bio Projects | i5K Pilot NCBI Bio-project | PRJNA163973  https://www.ncbi.nlm.nih.gov/bioproject/163973 |
| --- | --- | --- |
|  | *Gerris buenoi* NCBI Bio-project | PRJNA203045  <https://www.ncbi.nlm.nih.gov/bioproject/203045> |
|  | NCBI Bio-sample | SAMN02800617  https://www.ncbi.nlm.nih.gov/biosample/2800617 |
| Genome Sequence | 180bp insert *male* DNA | 1 Illumina HiSeq 2000 run: 122.1M read pairs, 24.7Gbp |
|  | 500bp insert *male* DNA | 1 Illumina HiSeq 2500 run: 36.4M read pairs, 7.4Gbp |
|  | 3kb insert *male* DNA | 1 Illumina HiSeq 2000 run: 137.4M read pairs, 27.8 Gbp |
|  | 8kb insert *female* DNA | 1 Illumina HiSeq 2000 run: 135.9M read pairs, 27.4 Gbp |
|  | 180bp insert NCBI SRA Accession | SRX493944  <https://www.ncbi.nlm.nih.gov/sra/SRX493944> |
|  | 500bp insert NCBI SRA Accession | SRX493946  <https://www.ncbi.nlm.nih.gov/sra/SRX493946> |
|  | 3kb insert NCBI SRA Accession | SRX493945  https://www.ncbi.nlm.nih.gov/sra/SRX493945 |
|  | 8kb insert NCBI SRA Accession | SRX493943  <https://www.ncbi.nlm.nih.gov/sra/SRX493943> |
| Genome Assembly | Number of contigs | 304,893 |
|  | Contig N50 | 3,812 bp |
|  | Number of scaffolds | 20,259 |
|  | Scaffold N50 | 344,118 bp |
|  | Size of final assembly | 1,000,161,732 bp |
|  | Size of final assembly - without gaps | 653,297,297 bp |
|  | NCBI Genome Assembly Accession | GCA_001010745.1  <https://www.ncbi.nlm.nih.gov/assembly/GCA_001010745.1> |
| RNAseq data | *Gerris buenoi*  Transcriptome Bio-project | PRJNA275657  https://www.ncbi.nlm.nih.gov/bioproject/275657 |
|  | Mixed sex embryos and nymphs RNAseq reads | 32M read pairs, 6.5 Gbp |
|  | Mixed sex embryos and nymphs SRA Accession | SRX896710  https://www.ncbi.nlm.nih.gov/sra/SRX896710 |
| Automated Genome Annotation (Gbue_0.5.3) | Genes (Gbue_0.5.3) | 20 949 |
|  | Average Transcript length | 1 298 |
|  | Average CDS length | 954 bp (318 aa) |
|  | Exons per gene | 4.81 |
|  | Genome Annotation Link | National Agricultural Library  https://i5k.nal.usda.gov/Gerris_buenoi |

Table S18 : Sequencing, assembly, annotation statistics and accession numbers

## **References**

1. Kongton K, McCall K, Phongdara A: **Identification of gamma-interferon-inducible lysosomal thiol reductase (GILT) homologues in the fruit fly Drosophila melanogaster**. *Developmental & Comparative Immunology* 2014, **44**(2):389-396.

2. De Gregorio E, Spellman PT, Tzou P, Rubin GM, Lemaitre B: **The Toll and Imd pathways are the major regulators of the immune response in Drosophila**. *EMBO Journal* 2002, **21**(11):2568-2579.

3. Zou Z, Evans JD, Lu Z, Zhao P, Williams M, Sumathipala N, Hetru C, Hultmark D, Jiang H: **Comparative genomic analysis of the Tribolium immune system**. *Genome biology* 2007, **8**(8):R177.

4. Chipman AD, Ferrier DE, Brena C, Qu J, Hughes DS, Schroder R, Torres-Oliva M, Znassi N, Jiang H, Almeida FC *et al*: **The first myriapod genome sequence reveals conservative arthropod gene content and genome organisation in the centipede Strigamia maritima**. *PLoS biology* 2014, **12**(11):e1002005.

5. Hoffmann JA, Reichhart JM: **Drosophila innate immunity: an evolutionary perspective**. *Nature immunology* 2002, **3**(2):121-126.

6. Tzou P, De Gregorio E, Lemaitre B: **How Drosophila combats microbial infection: a model to study innate immunity and host-pathogen interactions**. *Current opinion in microbiology* 2002, **5**(1):102-110.

7. Consortium TIAG: **Genome sequence of the pea aphid Acyrthosiphon pisum**. *PLoS Biology*

2010, **8**(2):e1000313.

8. Gerardo NM, Altincicek B, Anselme C, Atamian H, Barribeau SM, de Vos M, Duncan EJ, Evans JD, Gabaldon T, Ghanim M *et al*: **Immunity and other defenses in pea aphids, Acyrthosiphon pisum**. *Genome biology* 2010, **11**(2):R21.

9. Panfilio KA, Vargas Jentzsch IM, Benoit JB, Erezyilmaz D, Suzuki Y, Colella S, Robertson HM, Poelchau MF, Waterhouse RM, Ioannidis P *et al*: **Molecular evolutionary trends and feeding ecology diversification in the Hemiptera, anchored by the milkweed bug genome**. *bioRxiv* 2017, 201731:doi: 10.1101/201731.

10. Jenssen H, Hamill P, Hancock RE: **Peptide antimicrobial agents**. *Clinical Microbioly Reviews* 2006, **19**(3):491-511.

11. Vilcinskas A: **Evolutionary plasticity of insect immunity**. *Journal of insect physiology* 2013, **59**(2):123-129.

12. Armisen D, Refki PN, Crumiere AJ, Viala S, Toubiana W, Khila A: **Predator strike shapes antipredator phenotype through new genetic interactions in water striders**. *Nature communications* 2015, **6**:8153.

13. Khila A, Abouheif E, Rowe L: **Evolution of a novel appendage ground plan in water striders is driven by changes in the Hox gene Ultrabithorax**. *PLoS genetics* 2009, **5**(7):e1000583.

14. Kalinka AT, Varga KM, Gerrard DT, Preibisch S, Corcoran DL, Jarrells J, Ohler U, Bergman CM, Tomancak P: **Gene expression divergence recapitulates the developmental hourglass model**. *Nature* 2010, **468**(7325):811-814.

15. Refki PN, Khila A: **Key patterning genes contribute to leg elongation in water striders**. *Evodevo* 2015, **6**:14.

16. Kirkness EF, Haas BJ, Sun W, Braig HR, Perotti MA, Clark JM, Lee SH, Robertson HM, Kennedy RC, Elhaik E *et al*: **Genome sequences of the human body louse and its primary endosymbiont provide insights into the permanent parasitic lifestyle**. *Proceedings of the National Academy of Sciences* 2010, **107**(27):12168-12173.

17. Christiaens O, Iga M, Velarde RA, Rouge P, Smagghe G: **Halloween genes and nuclear receptors in ecdysteroid biosynthesis and signalling in the pea aphid**. *Insect molecular biology* 2010, **19 Suppl 2**:187-200.

18. Shigenobu S, Bickel RD, Brisson JA, Butts T, Chang CC, Christiaens O, Davis GK, Duncan EJ, Ferrier DE, Iga M *et al*: **Comprehensive survey of developmental genes in the pea aphid, Acyrthosiphon pisum: frequent lineage-specific duplications and losses of developmental genes**. *Insect molecular biology* 2010, **19 Suppl 2**:47-62.

19. Naggan Perl T, Schmid BG, Schwirz J, Chipman AD: **The evolution of the knirps family of transcription factors in arthropods**. *Molecular biology and evolution* 2013, **30**(6):1348-1357.

20. Watanabe T, Takeuchi H, Kubo T: **Structural diversity and evolution of the N-terminal isoform-specific region of ecdysone receptor-A and -B1 isoforms in insects**. *BMC Evolultionary Biology*

2010, **10**:40.

21. Dang CW, Wang Y, Chen KP, Yao Q, Zhang DB, Guo M: **The basic helix-loop-helix transcription factor family in the pea aphid, Acyrthosiphon pisum**. *Journal of Insect Science* 2011, **11**:84.

22. Bitra K, Tan A, Dowling A, Palli SR: **Functional characterization of PAS and HES family bHLH transcription factors during the metamorphosis of the red flour beetle, Tribolium castaneum**. *Gene* 2009, **448**(1):74-87.

23. Baker KD, Thummel CS: **Diabetic larvae and obese flies-emerging studies of metabolism in Drosophila**. *Cell Metabolism* 2007, **6**(4):257-266.

24. Edgar BA: **How flies get their size: genetics meets physiology**. *Nature Reviews Cancer* 2006, **7**(12):907-916.

25. Martin DE, Hall MN: **The expanding TOR signaling network**. *Current opinion in cell biology* 2005, **17**(2):158-166.

26. Junger MA, Rintelen F, Stocker H, Wasserman JD, Vegh M, Radimerski T, Greenberg ME, Hafen E: **The Drosophila forkhead transcription factor FOXO mediates the reduction in cell number associated with reduced insulin signaling**. *Journal of Biology* 2003, **2**(3):20.

27. Puig O, Tjian R: **Transcriptional feedback control of insulin receptor by dFOXO/FOXO1**. *Genes & development* 2005, **19**(20):2435-2446.

28. Kapahi P, Zid BM, Harper T, Koslover D, Sapin V, Benzer S: **Regulation of lifespan in Drosophila by modulation of genes in the TOR signaling pathway**. *Current Biology* 2004, **14**(10):885-890.

29. Wang MC, Bohmann D, Jasper H: **JNK extends life span and limits growth by antagonizing cellular and organism-wide responses to insulin signaling**. *Cell* 2005, **121**(1):115-125.

30. Wullschleger S, Loewith R, Hall MN: **TOR signaling in growth and metabolism**. *Cell* 2006, **124**(3):471-484.

31. Emlen DJ, Szafran Q, Corley LS, Dworkin I: **Insulin signaling and limb-patterning: candidate pathways for the origin and evolutionary diversification of beetle 'horns'**. *Heredity (Edinb)* 2006, **97**(3):179-191.

32. Hattori A, Sugime Y, Sasa C, Miyakawa H, Ishikawa Y, Miyazaki S, Okada Y, Cornette R, Lavine LC, Emlen DJ *et al*: **Soldier morphogenesis in the damp-wood termite is regulated by the insulin signaling pathway**. *Journal of Experimental Zoology B Molecular and Developmental Evolution* 2013, **320**(5):295-306.

33. Patel A, Fondrk MK, Kaftanoglu O, Emore C, Hunt G, Frederick K, Amdam GV: **The making of a queen: TOR pathway is a key player in diphenic caste development**. *PloS one* 2007, **2**(6):e509.

34. Emlen DJ, Warren IA, Johns A, Dworkin I, Lavine LC: **A mechanism of extreme growth and reliable signaling in sexually selected ornaments and weapons**. *Science* 2012, **337**(6096):860-864.

35. Snell-Rood EC, Moczek AP: **Insulin signaling as a mechanism underlying developmental plasticity: the role of FOXO in a nutritional polyphenism**. *PloS one* 2012, **7**(4):e34857.

36. Murat S, Hopfen C, McGregor AP: **The function and evolution of Wnt genes in arthropods**. *Arthropod Structure & Development* 2010, **39**(6):446-452.

37. Oberhofer G, Grossmann D, Siemanowski JL, Beissbarth T, Bucher G: **Wnt/beta-catenin signaling integrates patterning and metabolism of the insect growth zone**. *Development* 2014, **141**(24):4740-4750.

38. Janssen R, Le Gouar M, Pechmann M, Poulin F, Bolognesi R, Schwager EE, Hopfen C, Colbourne JK, Budd GE, Brown SJ *et al*: **Conservation, loss, and redeployment of Wnt ligands in protostomes: implications for understanding the evolution of segment formation**. *BMC Evolultionary Biology*

2010, **10**:374.

39. Beermann A, Pruhs R, Lutz R, Schroder R: **A context-dependent combination of Wnt receptors controls axis elongation and leg development in a short germ insect**. *Development* 2011, **138**(13):2793-2805.

40. Rawlings ND, Barrett AJ, Finn R: **Twenty years of the MEROPS database of proteolytic enzymes, their substrates and inhibitors**. *Nucleic acids research* 2016, **44**(D1):D343-350.

41. Turk V, Stoka V, Vasiljeva O, Renko M, Sun T, Turk B, Turk D: **Cysteine cathepsins: from structure, function and regulation to new frontiers**. *Biochimica et biophysica acta* 2012, **1824**(1):68-88.

42. Terra WR, Ferreira C: **Insect digestive enzymes: properties, compartmentalization and function**. *Comparative Biochemistry and Physiology Part B: Comparative Biochemistry* 1994, **109**(1):1-62.

43. Terra WR, Ferreira C: **11 - Biochemistry and Molecular Biology of Digestion A2 - Gilbert, Lawrence I**. In: *Insect Molecular Biology and Biochemistry.* San Diego: Academic Press; 2012: 365-418.

44. Murdock LL, Brookhart G, Dunn PE, Foard DE, Kelley S, Kitch L, Shade RE, Shukle RH, Wolfson JL: **Cysteine digestive proteinases in Coleoptera**. *Comparative Biochemistry and Physiology Part B: Comparative Biochemistry* 1987, **87**(4):783-787.

45. Houseman JG, Downe AER: **Cathepsin D-like activity in the posterior midgut of hemipteran insects**. *Comparative Biochemistry and Physiology Part B: Comparative Biochemistry* 1983, **75**(3):509-512.

46. Martynov AG, Elpidina EN, Perkin L, Oppert B: **Functional analysis of C1 family cysteine peptidases in the larval gut of capital Tenebrio molitor and Tribolium castaneum**. *BMC genomics* 2015, **16**:75.

47. Perkin L, Elpidina EN, Oppert B: **Expression patterns of cysteine peptidase genes across the Tribolium castaneum life cycle provide clues to biological function**. *PeerJ* 2016, **4**:e1581.

48. Oppert B, Elpidina EN, Toutges M, Mazumdar-Leighton S: **Microarray analysis reveals strategies of Tribolium castaneum larvae to compensate for cysteine and serine protease inhibitors**. *Comparative Biochemistry and Physiology - Part D: Genomics and Proteomics* 2010, **5**(4):280-287.

49. Kollien AH, Waniek PJ, Nisbet AJ, Billingsley PF, Schaub GA: **Activity and sequence characterization of two cysteine proteases in the digestive tract of the reduviid bug Triatoma infestans**. *Insect molecular biology* 2004, **13**(6):569-579.

50. Waniek PJ, Pacheco Costa JE, Jansen AM, Costa J, Araujo CA: **Cathepsin L of Triatoma brasiliensis (Reduviidae, Triatominae): sequence characterization, expression pattern and zymography**. *Journal of insect physiology* 2012, **58**(1):178-187.

51. Ribeiro JM, Genta FA, Sorgine MH, Logullo R, Mesquita RD, Paiva-Silva GO, Majerowicz D, Medeiros M, Koerich L, Terra WR *et al*: **An insight into the transcriptome of the digestive tract of the bloodsucking bug, Rhodnius prolixus**. *PLoS Neglected Tropical Diseases* 2014, **8**(1):e2594.

52. Novinec M, Lenarcic B: **Papain-like peptidases: structure, function, and evolution**. *Biomolecular Concepts* 2013, **4**(3):287-308.

53. Sakurai M, Sato Y, Mukai K, Suematsu M, Fukui E, Yoshizawa M, Tanemura K, Hoshino Y, Matsumoto H, Sato E: **Distribution of tubulointerstitial nephritis antigen-like 1 and structural matrix proteins in mouse embryos during preimplantation development in vivo and in vitro**. *Zygote* 2014, **22**(2):259-265.

54. Saito H, Kurata S, Natori S: **Purification and characterization of a hemocyte proteinase of Sarcophaga, possibly participating in elimination of foreign substances**. *European Journal of Biochemistry* 1992, **209**(3):939-944.

55. Gruden K, Popovic T, Cimerman N, Krizaj I, Strukelj B: **Diverse enzymatic specificities of digestive proteases, 'intestains', enable Colorado potato beetle larvae to counteract the potato defence mechanism**. *Journal of Biological Chemistry* 2003, **384**(2):305-310.

56. Meyer HW: **Visuelle Schlüsselreize für die Auslösung der Beutefanghandlung beim Bachwasserläufer Velia caprai (Hemiptera, Heteroptera)**. *Zeitschrift für vergleichende Physiologie* 1971, **72**(3):260-297.

57. Rowe L: **The costs of mating and mate choice in water striders.** *Animal Behaviour* 1994, **48**:1049-1056.

58. Spence JR, Anderson N: **Biology of water striders: interactions between systematics and ecology**. *Annual Review of Entomology* 1994, **39**(1):101-128.

59. Dahmen H: **Eye specialisation in waterstriders: an adaptation to life in a flat world**. *Journal of Comparative Physiology A* 1991, **169**(5):623-632.

60. Wolburg-Buchholz K: **The organization of the lamina ganglionaris of the hemipteran insects, Notonecta glauca, Corixa punctata and Gerris lacustris**. *Cell and Tissue Research* 1979, **197**(1):39-59.

61. Schneider L, Langer H: **Die Struktur des Rhabdoms im „Doppelauge“ des Wasserläufers Gerris lacustris**: Zeitschrift für Zellforschung und Mikroskopische Anatomie; 1969.

62. Fischer C, Mahner M, Wachmann E: **The rhabdom structure in the ommatidia of the Heteroptera (Insecta), and its phylogenetic significance**. *Zoomorphology* 2000, **120**(1):1-13.

63. Frolov R, Weckström M: **Developmental changes in biophysical properties of photoreceptors in the common water strider (Gerris lacustris): better performance at higher cost**. *Journal of neurophysiology* 2014, **112**(4):913-922.

64. Schwind R: **Polarization vision in water insects and insects living on a moist substrate**. *Journal of Comparative Physiology A* 1991, **169**(5):531-540.

65. Bohn H, Täuber U: **Beziehungen zwischen der Wirkung polarisierten Lichtes auf das Elektroretinogramm und der Ultrastruktur des Auges von Gerris lacustris L**. *Zeitschrift für vergleichende Physiologie* 1971, **72**(1):32-53.

66. Bartsch K: **Polarization-sensitive photoreceptors of different spectral types in the compound eye of waterstriders**. *Naturwissenschaften* 1995, **82**(6):292-293.

67. Brody T, Cravchik A: **Drosophila melanogaster G protein-coupled receptors**. *Journal of Cell Biology* 2000, **150**(2):F83-88.

68. Senthilan PR, Helfrich-Forster C: **Rhodopsin 7-The unusual Rhodopsin in Drosophila**. *PeerJ* 2016, **4**:e2427.

69. Colbourne JK, Pfrender ME, Gilbert D, Thomas WK, Tucker A, Oakley TH, Tokishita S, Aerts A, Arnold GJ, Basu MK *et al*: **The ecoresponsive genome of Daphnia pulex**. *Science* 2011, **331**(6017):555-561.

70. Eriksson BJ, Fredman D, Steiner G, Schmid A: **Characterisation and localisation of the opsin protein repertoire in the brain and retinas of a spider and an onychophoran**. *BMC Evolultionary Biology*

2013, **13**:186.

71. Hering L, Mayer G: **Analysis of the opsin repertoire in the tardigrade Hypsibius dujardini provides insights into the evolution of opsin genes in panarthropoda**. *Genome biology and evolution* 2014, **6**(9):2380-2391.

72. Henze MJ, Oakley TH: **The Dynamic Evolutionary History of Pancrustacean Eyes and Opsins**. *Integrative and comparative biology* 2015, **55**(5):830-842.

73. Frentiu FD, Bernard GD, Cuevas CI, Sison-Mangus MP, Prudic KL, Briscoe AD: **Adaptive evolution of color vision as seen through the eyes of butterflies**. *Proceedings of the National Academy of Sciences* 2007, **104 Suppl 1**:8634-8640.

74. Frentiu FD, Bernard GD, Sison-Mangus MP, Brower AV, Briscoe AD: **Gene duplication is an evolutionary mechanism for expanding spectral diversity in the long-wavelength photopigments of butterflies**. *Molecular biology and evolution* 2007, **24**(9):2016-2028.

75. Sharkey CR, Fujimoto MS, Lord NP, Shin S, McKenna DD, Suvorov A, Martin GJ, Bybee SM: **Overcoming the loss of blue sensitivity through opsin duplication in the largest animal group, beetles**. *Reports* 2017, **7**(1):8.

76. Briscoe AD, Chittka L: **The evolution of color vision in insects**. *Annual Review of Entomology* 2001, **46**:471-510.

77. Goldsmith TH, Ruck PR: **The spectral sensitivities of the dorsal ocelli of cockroaches and honeybees; an electrophysiological study**. *Journal of General Physiology* 1958, **41**(6):1171-1185.

78. Benton R: **Multigene Family Evolution: Perspectives from Insect Chemoreceptors**. *Trends in ecology & evolution* 2015, **30**(10):590-600.

79. Joseph RM, Carlson JR: **Drosophila Chemoreceptors: A Molecular Interface Between the Chemical World and the Brain**. *Trends in Genetics* 2015, **31**(12):683-695.

80. Mesquita RD, Vionette-Amaral RJ, Lowenberger C, Rivera-Pomar R, Monteiro FA, Minx P, Spieth J, Carvalho AB, Panzera F, Lawson D *et al*: **Genome of Rhodnius prolixus, an insect vector of Chagas disease, reveals unique adaptations to hematophagy and parasite infection**. *Proceedings of the National Academy of Sciences* 2015, **112**(48):14936-14941.

81. Benoit JB, Adelman ZN, Reinhardt K, Dolan A, Poelchau M, Jennings EC, Szuter EM, Hagan RW, Gujar H, Shukla JN *et al*: **Unique features of a global human ectoparasite identified through sequencing of the bed bug genome**. *Nature communications* 2016, **7**:10165.

82. International Aphid Genomics C: **Genome sequence of the pea aphid Acyrthosiphon pisum**. *PLoS biology* 2010, **8**(2):e1000313.

83. Ioannidis P, Simao FA, Waterhouse RM, Manni M, Seppey M, Robertson HM, Misof B, Niehuis O, Zdobnov EM: **Genomic features of the damselfly Calopteryx splendens representing a sister clade to most insect orders**. *Genome biology and evolution* 2017.

84. Missbach C, Dweck HK, Vogel H, Vilcinskas A, Stensmyr MC, Hansson BS, Grosse-Wilde E: **Evolution of insect olfactory receptors**. *eLife* 2014, **3**:e02115.

85. Robertson HM: **The Insect Chemoreceptor Superfamily Is Ancient in Animals**. *Chemical Senses* 2015, **40**(9):609-614.

86. Robertson HM, Warr CG, Carlson JR: **Molecular evolution of the insect chemoreceptor gene superfamily in Drosophila melanogaster**. *Proceedings of the National Academy of Sciences* 2003, **100 Suppl 2**:14537-14542.

87. Miyamoto T, Slone J, Song X, Amrein H: **A fructose receptor functions as a nutrient sensor in the Drosophila brain**. *Cell* 2012, **151**(5):1113-1125.

88. Rytz R, Croset V, Benton R: **Ionotropic receptors (IRs): chemosensory ionotropic glutamate receptors in Drosophila and beyond**. *Insect biochemistry and molecular biology* 2013, **43**(9):888-897.

89. Croset V, Schleyer M, Arguello JR, Gerber B, Benton R: **A molecular and neuronal basis for amino acid sensing in the Drosophila larva**. *Reports* 2016, **6**:34871.

90. Ganguly A, Pang L, Duong VK, Lee A, Schoniger H, Varady E, Dahanukar A: **A Molecular and Cellular Context-Dependent Role for Ir76b in Detection of Amino Acid Taste**. *Cell Reports* 2017, **18**(3):737-750.

91. Enjin A, Zaharieva EE, Frank DD, Mansourian S, Suh GS, Gallio M, Stensmyr MC: **Humidity Sensing in Drosophila**. *Current Biology* 2016, **26**(10):1352-1358.

92. Knecht ZA, Silbering AF, Ni L, Klein M, Budelli G, Bell R, Abuin L, Ferrer AJ, Samuel AD, Benton R *et al*: **Distinct combinations of variant ionotropic glutamate receptors mediate thermosensation and hygrosensation in Drosophila**. *eLife* 2016, **5**.

93. Hussain A, Zhang M, Ucpunar HK, Svensson T, Quillery E, Gompel N, Ignell R, Grunwald Kadow IC: **Ionotropic Chemosensory Receptors Mediate the Taste and Smell of Polyamines**. *PLoS biology* 2016, **14**(5):e1002454.

94. Min S, Ai M, Shin SA, Suh GS: **Dedicated olfactory neurons mediating attraction behavior to ammonia and amines in Drosophila**. *Proceedings of the National Academy of Sciences* 2013, **110**(14):E1321-1329.

95. Ai M, Min S, Grosjean Y, Leblanc C, Bell R, Benton R, Suh GS: **Acid sensing by the Drosophila olfactory system**. *Nature* 2010, **468**(7324):691-695.

96. Gorter JA, Jagadeesh S, Gahr C, Boonekamp JJ, Levine JD, Billeter JC: **The nutritional and hedonic value of food modulate sexual receptivity in Drosophila melanogaster females**. *Reports* 2016, **6**:19441.

97. Grosjean Y, Rytz R, Farine JP, Abuin L, Cortot J, Jefferis GS, Benton R: **An olfactory receptor for food-derived odours promotes male courtship in Drosophila**. *Nature* 2011, **478**(7368):236-240.

98. Prieto-Godino LL, Rytz R, Bargeton B, Abuin L, Arguello JR, Peraro MD, Benton R: **Olfactory receptor pseudo-pseudogenes**. *Nature* 2016, **539**(7627):93-97.

99. Prieto-Godino LL, Rytz R, Cruchet S, Bargeton B, Abuin L, Silbering AF, Ruta V, Dal Peraro M, Benton R: **Evolution of Acid-Sensing Olfactory Circuits in Drosophilids**. *Neuron* 2017, **93**(3):661-676 e666.

100. Koh TW, He Z, Gorur-Shandilya S, Menuz K, Larter NK, Stewart S, Carlson JR: **The Drosophila IR20a clade of ionotropic receptors are candidate taste and pheromone receptors**. *Neuron* 2014, **83**(4):850-865.

101. Stewart S, Koh TW, Ghosh AC, Carlson JR: **Candidate ionotropic taste receptors in the Drosophila larva**. *Proceedings of the National Academy of Sciences* 2015, **112**(14):4195-4201.

102. Ffrench-Constant RH, Daborn PJ, Le Goff G: **The genetics and genomics of insecticide resistance**. *Trends in Genetics* 2004, **20**(3):163-170.

103. Scott JG: **Cytochromes P450 and insecticide resistance**. *Insect biochemistry and molecular biology* 1999, **29**(9):757-777.

104. Rewitz KF, O'Connor MB, Gilbert LI: **Molecular evolution of the insect Halloween family of cytochrome P450s: phylogeny, gene organization and functional conservation**. *Insect biochemistry and molecular biology* 2007, **37**(8):741-753.

105. Helvig C, Koener JF, Unnithan GC, Feyereisen R: **CYP15A1, the cytochrome P450 that catalyzes epoxidation of methyl farnesoate to juvenile hormone III in cockroach corpora allata**. *Proceedings of the National Academy of Sciences* 2004, **101**(12):4024-4029.

106. Good RT, Gramzow L, Battlay P, Sztal T, Batterham P, Robin C: **The molecular evolution of cytochrome P450 genes within and between drosophila species**. *Genome biology and evolution* 2014, **6**(5):1118-1134.

107. Lao SH, Huang XH, Huang HJ, Liu CW, Zhang CX, Bao YY: **Genomic and transcriptomic insights into the cytochrome P450 monooxygenase gene repertoire in the rice pest brown planthopper, Nilaparvata lugens**. *Genomics* 2015, **106**(5):301-309.

108. Schama R, Pedrini N, Juarez MP, Nelson DR, Torres AQ, Valle D, Mesquita RD: **Rhodnius prolixus supergene families of enzymes potentially associated with insecticide resistance**. *Insect biochemistry and molecular biology* 2016, **69**:91-104.

109. Morello A, Repetto Y: **UDP-glucosyltransferase activity of housefly microsomal fraction**. *Biochemical Journal* 1979, **177**(3):809-812.

110. Real MD, Ferre J, Chapa FJ: **UDP-glucosyltransferase activity toward exogenous substrates in Drosophila melanogaster**. *Analytical biochemistry* 1991, **194**(2):349-352.

111. Ahmad SA, Hopkins TL: **Phenol β-glucosyltransferase and β-glucosidase activities in the tobacco hornworm larva Manduca sexta (L.): Properties and tissue localization**. *Archives of Insect Biochemistry and Physiology* 1992, **21**(3):207-224.

112. Luque T, Okano K, O'Reilly DR: **Characterization of a novel silkworm (Bombyx mori) phenol UDP-glucosyltransferase**. *European Journal of Biochemistry* 2002, **269**(3):819-825.

113. Ahmad SA, Hopkins TL: **Phenol β-glucosyltransferases in six species of insects: properties and tissue localization**. *Comparative Biochemistry and Physiology Part B: Comparative Biochemistry* 1993, **104**(3):515-519.

114. Ahn SJ, Badenes-Perez FR, Heckel DG: **A host-plant specialist, Helicoverpa assulta, is more tolerant to capsaicin from Capsicum annuum than other noctuid species**. *Journal of insect physiology* 2011, **57**(9):1212-1219.

115. Daimon T, Hirayama C, Kanai M, Ruike Y, Meng Y, Kosegawa E, Nakamura M, Tsujimoto G, Katsuma S, Shimada T: **The silkworm Green b locus encodes a quercetin 5-O-glucosyltransferase that produces green cocoons with UV-shielding properties**. *Proceedings of the National Academy of Sciences* 2010, **107**(25):11471-11476.

116. Kojima W, Fujii T, Suwa M, Miyazawa M, Ishikawa Y: **Physiological adaptation of the Asian corn borer Ostrinia furnacalis to chemical defenses of its host plant, maize**. *Journal of insect physiology* 2010, **56**(9):1349-1355.

117. Lee H-S, Hieu T, Ahn Y-J: **Oviposition-stimulating activity of (E)-capsaicin identified in Capsicum annuum fruit and related compounds towards Helicoverpa assulta (Lepidoptera: Noctuidae)**. *Chemoecology* 2006, **16**(3):153-157.

118. Sasai H, Ishida M, Murakami K, Tadokoro N, Ishihara A, Nishida R, Mori N: **Species-specific glucosylation of DIMBOA in larvae of the rice Armyworm**. *Bioscience, biotechnology, and biochemistry* 2009, **73**(6):1333-1338.

119. Wang Q, Hasan G, Pikielny CW: **Preferential expression of biotransformation enzymes in the olfactory organs of Drosophila melanogaster, the antennae**. *Journal of Biological Chemistry* 1999, **274**(15):10309-10315.

120. Younus F, Chertemps T, Pearce SL, Pandey G, Bozzolan F, Coppin CW, Russell RJ, Maibeche-Coisne M, Oakeshott JG: **Identification of candidate odorant degrading gene/enzyme systems in the antennal transcriptome of Drosophila melanogaster**. *Insect biochemistry and molecular biology* 2014, **53**:30-43.

121. Bozzolan F, Siaussat D, Maria A, Durand N, Pottier MA, Chertemps T, Maibeche-Coisne M: **Antennal uridine diphosphate (UDP)-glycosyltransferases in a pest insect: diversity and putative function in odorant and xenobiotics clearance**. *Insect molecular biology* 2014, **23**(5):539-549.

122. Svoboda JA, Weirich GF: **Sterol metabolism in the tobacco hornworm, Manduca sexta--a review**. *Lipids* 1995, **30**(3):263-267.

123. Ahmad SA, Hopkins TL, Kramer KJ: **Tyrosine β-Glucosyltransferase in the tobacco hornworm, Manduca sexta (L.): properties, tissue localization, and developmental profile**. *Insect biochemistry and molecular biology* 1996, **26**(1):49-57.

124. Hopkins TL, Kramer JB: **Insect Cuticle Sclerotization**. *Annual Review of Entomology* 1992, **37**(1):273-302.

125. Wiesen B, Krug E, Fiedler K, Wray V, Proksch P: **Sequestration of host-plant-derived flavonoids by lycaenid butterflyPolyommatus icarus**. *Journal of chemical ecology* 1994, **20**(10):2523-2538.

126. Jensen NB, Zagrobelny M, Hjerno K, Olsen CE, Houghton-Larsen J, Borch J, Moller BL, Bak S: **Convergent evolution in biosynthesis of cyanogenic defence compounds in plants and insects**. *Nature communications* 2011, **2**:273.

127. Ahn SJ, Vogel H, Heckel DG: **Comparative analysis of the UDP-glycosyltransferase multigene family in insects**. *Insect biochemistry and molecular biology* 2012, **42**(2):133-147.

128. Simpson SJ, Sword GA, Lo N: **Polyphenism in insects**. *Current Biology* 2011, **21**(18):R738-749.

129. Roff DA: **The Evolution of Wing Dimorphism in Insects**. *Evolution; international journal of organic evolution* 1986, **40**(5):1009-1020.

130. Harada T, Taneda K: **Seasonal changes in alary dimorphism of a water strider, Gerris paludum insularis (Motschulsky)**. *Journal of insect physiology* 1989, **35**(12):919-924.

131. Vepsäläinen K: **Determination of wing length and diapause in water-striders (gerris fabr., heteroptera)**. *Hereditas* 1974, **77**(2):163-176.

132. Vepsäläinen K: **Wing Dimorphism and Diapause in Gerris: Determination and Adaptive Significance**. In: *Evolution of Insect Migration and Diapause.* Edited by Dingle H: Springer US; 1978: 218-253.

133. Suen G, Teiling C, Li L, Holt C, Abouheif E, Bornberg-Bauer E, Bouffard P, Caldera EJ, Cash E, Cavanaugh A *et al*: **The genome sequence of the leaf-cutter ant Atta cephalotes reveals insights into its obligate symbiotic lifestyle**. *PLoS genetics* 2011, **7**(2):e1002007.

134. Smith CR, Smith CD, Robertson HM, Helmkampf M, Zimin A, Yandell M, Holt C, Hu H, Abouheif E, Benton R *et al*: **Draft genome of the red harvester ant Pogonomyrmex barbatus**. *Proceedings of the National Academy of Sciences* 2011, **108**(14):5667-5672.

135. Smith CD, Zimin A, Holt C, Abouheif E, Benton R, Cash E, Croset V, Currie CR, Elhaik E, Elsik CG *et al*: **Draft genome of the globally widespread and invasive Argentine ant (Linepithema humile)**. *Proceedings of the National Academy of Sciences* 2011, **108**(14):5673-5678.

136. Brisson JA: **Aphid wing dimorphisms: linking environmental and genetic control of trait variation**. *Philosophical transactions of the Royal Society of London Series B, Biological sciences* 2010, **365**(1540):605-616.

137. Elango N, Hunt BG, Goodisman MA, Yi SV: **DNA methylation is widespread and associated with differential gene expression in castes of the honeybee, Apis mellifera**. *Proceedings of the National Academy of Sciences* 2009, **106**(27):11206-11211.

138. Nijhout HF: **Development and evolution of adaptive polyphenisms**. *Evolution & development* 2003, **5**(1):9-18.

139. Nijhout HF: **Insect polyphenisms and adaptation.** *American Zoologist* 2001, **41**(6):1540-1540.

140. Xu HJ, Xue J, Lu B, Zhang XC, Zhuo JC, He SF, Ma XF, Jiang YQ, Fan HW, Xu JY *et al*: **Two insulin receptors determine alternative wing morphs in planthoppers**. *Nature* 2015, **519**(7544):464-467.

141. Nijhout HF: **Control Mechanisms of Polyphenic Development in Insects: In polyphenic development, environmental factors alter some aspects of development in an orderly and predictable way.** *Bioscience* 1999, **49**(3):181-192.

142. Alvarado S, Rajakumar R, Abouheif E, Szyf M: **Epigenetic variation in the Egfr gene generates quantitative variation in a complex trait in ants**. *Nature Communications*

2015, **6**:6513.

143. Foret S, Kucharski R, Pellegrini M, Feng S, Jacobsen SE, Robinson GE, Maleszka R: **DNA methylation dynamics, metabolic fluxes, gene splicing, and alternative phenotypes in honey bees**. *Proceedings of the National Academy of Sciences* 2012, **109**(13):4968-4973.

144. Li-Byarlay H, Li Y, Stroud H, Feng S, Newman TC, Kaneda M, Hou KK, Worley KC, Elsik CG, Wickline SA *et al*: **RNA interference knockdown of DNA methyl-transferase 3 affects gene alternative splicing in the honey bee**. *Proceedings of the National Academy of Sciences* 2013, **110**(31):12750-12755.

145. Kucharski R, Maleszka J, Foret S, Maleszka R: **Nutritional control of reproductive status in honeybees via DNA methylation**. *Science* 2008, **319**(5871):1827-1830.

146. Herb BR, Wolschin F, Hansen KD, Aryee MJ, Langmead B, Irizarry R, Amdam GV, Feinberg AP: **Reversible switching between epigenetic states in honeybee behavioral subcastes**. *Nature neuroscience* 2012, **15**(10):1371-1373.

147. Glastad KM, Hunt BG, Yi SV, Goodisman MA: **DNA methylation in insects: on the brink of the epigenomic era**. *Insect molecular biology* 2011, **20**(5):553-565.

148. Bewick AJ, Vogel KJ, Moore AJ, Schmitz RJ: **Evolution of DNA Methylation across Insects**. *Molecular biology and evolution* 2017, **34**(3):654-665.

149. Walsh TK, Brisson JA, Robertson HM, Gordon K, Jaubert-Possamai S, Tagu D, Edwards OR: **A functional DNA methylation system in the pea aphid, Acyrthosiphon pisum**. *Insect molecular biology* 2010, **19 Suppl 2**:215-228.

150. Vandegehuchte MB, Kyndt T, Vanholme B, Haegeman A, Gheysen G, Janssen CR: **Occurrence of DNA methylation in Daphnia magna and influence of multigeneration Cd exposure**. *Environ Int* 2009, **35**(4):700-706.

151. Terrapon N, Li C, Robertson HM, Ji L, Meng X, Booth W, Chen Z, Childers CP, Glastad KM, Gokhale K *et al*: **Molecular traces of alternative social organization in a termite genome**. *Nature communications* 2014, **5**:3636.

152. Bonasio R, Zhang G, Ye C, Mutti NS, Fang X, Qin N, Donahue G, Yang P, Li Q, Li C *et al*: **Genomic comparison of the ants Camponotus floridanus and Harpegnathos saltator**. *Science* 2010, **329**(5995):1068-1071.

153. Wang Y, Jorda M, Jones PL, Maleszka R, Ling X, Robertson HM, Mizzen CA, Peinado MA, Robinson GE: **Functional CpG methylation system in a social insect**. *Science* 2006, **314**(5799):645-647.

154. Duncan EJ, Gluckman PD, Dearden PK: **Epigenetics, plasticity, and evolution: How do we link epigenetic change to phenotype?** *Journal of Experimental Zoology B Molecular and Developmental Evolution* 2014, **322**(4):208-220.

155. Andersen NM, xf, ller: **The Evolution of Wing Polymorphism in Water Striders (Gerridae): A Phylogenetic Approach**. *Oikos* 1993, **67**(3):433-443.

156. Andersen NM: **The semiaquatic bugs**, vol. 3. Klampenborg – Denmark: Scandinavian Science Pres Ltd; 1982.

157. Fairbairn DJ, King E: **Why do Californian striders fly?** *Journal of evolutionary biology* 2009, **22**(1):36-49.

158. Arnqvist G, Rowe L: **Sexual Conflict**: Princeton University Press; 2005.

159. McKay DJ, Klusza S, Penke TJ, Meers MP, Curry KP, McDaniel SL, Malek PY, Cooper SW, Tatomer DC, Lieb JD *et al*: **Interrogating the function of metazoan histones using engineered gene clusters**. *Developmental cell* 2015, **32**(3):373-386.

160. Conrad T, Cavalli FM, Holz H, Hallacli E, Kind J, Ilik I, Vaquerizas JM, Luscombe NM, Akhtar A: **The MOF chromobarrel domain controls genome-wide H4K16 acetylation and spreading of the MSL complex**. *Developmental cell* 2012, **22**(3):610-624.

161. Pushpavalli SN, Sarkar A, Ramaiah MJ, Chowdhury DR, Bhadra U, Pal-Bhadra M: **Drosophila MOF controls Checkpoint protein2 and regulates genomic stability during early embryogenesis**. *BMC molecular biology* 2013, **14**:1.

162. Rider SD, Jr., Srinivasan DG, Hilgarth RS: **Chromatin-remodelling proteins of the pea aphid, Acyrthosiphon pisum (Harris)**. *Insect molecular biology* 2010, **19 Suppl 2**:201-214.

163. Furuyama T, Banerjee R, Breen TR, Harte PJ: **SIR2 is required for polycomb silencing and is associated with an E(Z) histone methyltransferase complex**. *Current Biology* 2004, **14**(20):1812-1821.

164. Rogina B, Helfand SL: **Sir2 mediates longevity in the fly through a pathway related to calorie restriction**. *Proceedings of the National Academy of Sciences* 2004, **101**(45):15998-16003.

165. Tissenbaum HA, Guarente L: **Increased dosage of a sir-2 gene extends lifespan in Caenorhabditis elegans**. *Nature* 2001, **410**(6825):227-230.

166. List O, Togawa T, Tsuda M, Matsuo T, Elard L, Aigaki T: **Overexpression of grappa encoding a histone methyltransferase enhances stress resistance in Drosophila**. *Hereditas* 2009, **146**(1):19-28.

167. Bi JL, Felton GW: **Foliar oxidative stress and insect herbivory: Primary compounds, secondary metabolites, and reactive oxygen species as components of induced resistance**. *Journal of chemical ecology* 1995, **21**(10):1511-1530.

168. Mittapalli O, Neal JJ, Shukle RH: **Antioxidant defense response in a galling insect**. *Proceedings of the National Academy of Sciences* 2007, **104**(6):1889-1894.

169. Pardini RS: **Toxicity of oxygen from naturally occurring redox-active pro-oxidants**. *Archives of Insect Biochemistry and Physiology* 1995, **29**(2):101-118.

170. Corona M, Robinson GE: **Genes of the antioxidant system of the honey bee: annotation and phylogeny**. *Insect molecular biology* 2006, **15**(5):687-701.

171. Felton GW, Summers CB: **Antioxidant systems in insects**. *Archives of Insect Biochemistry and Physiology* 1995, **29**(2):187-197.

172. Shi GQ, Yu QY, Zhang Z: **Annotation and evolution of the antioxidant genes in the silkworm, Bombyx mori**. *Archives of Insect Biochemistry and Physiology* 2012, **79**(2):87-103.

173. Gnerre S, Maccallum I, Przybylski D, Ribeiro FJ, Burton JN, Walker BJ, Sharpe T, Hall G, Shea TP, Sykes S *et al*: **High-quality draft assemblies of mammalian genomes from massively parallel sequence data**. *Proceedings of the National Academy of Sciences* 2011, **108**(4):1513-1518.

174. **Atlas Gap Fill** [<https://www.hgsc.bcm.edu/software/>]

175. Cantarel BL, Korf I, Robb SM, Parra G, Ross E, Moore B, Holt C, Sanchez Alvarado A, Yandell M: **MAKER: an easy-to-use annotation pipeline designed for emerging model organism genomes**. *Genome research* 2008, **18**(1):188-196.

176. Stanke M, Diekhans M, Baertsch R, Haussler D: **Using native and syntenically mapped cDNA alignments to improve de novo gene finding**. *Bioinformatics* 2008, **24**(5):637-644.

177. Korf I: **Gene finding in novel genomes**. *BMC Bioinformatics* 2004, **5**:59.

178. **Gerris buenoi i5k NAL page** [<https://i5k.nal.usda.gov/Gerris_buenoi>]

179. Poelchau M, Childers C, Moore G, Tsavatapalli V, Evans J, Lee CY, Lin H, Lin JW, Hackett K: **The i5k Workspace@NAL--enabling genomic data access, visualization and curation of arthropod genomes**. *Nucleic acids research* 2015, **43**(Database issue):D714-719.

180. **NAL Apollo/Jbrowse** [<https://apollo.nal.usda.gov/gerbue/jbrowse/>]

181. **i5k Annotation Guidelines** [<https://i5k.nal.usda.gov/content/rules-web-apollo-annotation-i5k-pilot-project>]

182. **GFF3 toolkit** [<https://github.com/NAL-i5K/GFF3toolkit/>]

183. **Gerris buenoi Official Gene Set OGSv1.0** [<https://data.nal.usda.gov/dataset/gerris-buenoi-official-gene-set-v10>]

184. Gramates LS, Marygold SJ, Santos GD, Urbano JM, Antonazzo G, Matthews BB, Rey AJ, Tabone CJ, Crosby MA, Emmert DB *et al*: **FlyBase at 25: looking to the future**. *Nucleic acids research* 2017, **45**(D1):D663-D671.

185. **ClustalO** [<http://www.ebi.ac.uk/Tools/msa/clustalo/>]

186. Willis JH: **Structural cuticular proteins from arthropods: annotation, nomenclature, and sequence characteristics in the genomics era**. *Insect biochemistry and molecular biology* 2010, **40**(3):189-204.

187. Ioannidou ZS, Theodoropoulou MC, Papandreou NC, Willis JH, Hamodrakas SJ: **CutProtFam-Pred: detection and classification of putative structural cuticular proteins from sequence alone, based on profile hidden Markov models**. *Insect biochemistry and molecular biology* 2014, **52**:51-59.

188. Bird AP: **DNA methylation and the frequency of CpG in animal DNA**. *Nucleic acids research* 1980, **8**(7):1499-1504.

189. Weber M, Hellmann I, Stadler MB, Ramos L, Paabo S, Rebhan M, Schubeler D: **Distribution, silencing potential and evolutionary impact of promoter DNA methylation in the human genome**. *Nature genetics* 2007, **39**(4):457-466.

190. **Gerris buenoi i5k Blast** [<https://i5k.nal.usda.gov/webapp/blast/>]

191. **Gerris buenoi Web Apollo instance** [<https://apollo.nal.usda.gov/gerbue/selectTrack.jsp>]

192. **Phylogeny.fr** [<http://www.phylogeny.fr/>]

193. Wang L, Wang S, Li Y, Paradesi MS, Brown SJ: **BeetleBase: the model organism database for Tribolium castaneum**. *Nucleic acids research* 2007, **35**(Database issue):D476-479.

194. Wallace IM, O'Sullivan O, Higgins DG, Notredame C: **M-Coffee: combining multiple sequence alignment methods with T-Coffee**. *Nucleic acids research* 2006, **34**(6):1692-1699.

195. Capella-Gutierrez S, Silla-Martinez JM, Gabaldon T: **trimAl: a tool for automated alignment trimming in large-scale phylogenetic analyses**. *Bioinformatics* 2009, **25**(15):1972-1973.

196. Tamura K, Stecher G, Peterson D, Filipski A, Kumar S: **MEGA6: Molecular Evolutionary Genetics Analysis version 6.0**. *Molecular biology and evolution* 2013, **30**(12):2725-2729.

197. Jones DT, Taylor WR, Thornton JM: **The rapid generation of mutation data matrices from protein sequences**. *Computer Applications in the Biosciences* 1992, **8**(3):275-282.

198. Misra JR, Horner MA, Lam G, Thummel CS: **Transcriptional regulation of xenobiotic detoxification in Drosophila**. *Genes & development* 2011, **25**(17):1796-1806.

199. Norga KK, Gurganus MC, Dilda CL, Yamamoto A, Lyman RF, Patel PH, Rubin GM, Hoskins RA, Mackay TF, Bellen HJ: **Quantitative analysis of bristle number in Drosophila mutants identifies genes involved in neural development**. *Current Biology* 2003, **13**(16):1388-1396.

200. Gao N, Foster RG, Hardie J: **Two opsin genes from the vetch aphid, Megoura viciae**. *Insect molecular biology* 2000, **9**(2):197-202.

201. Wakakuwa M, Stewart F, Matsumoto Y, Matsunaga S, Arikawa K: **Physiological basis of phototaxis to near-infrared light in Nephotettix cincticeps**. *Journal of Comparative Physiology A* 2014, **200**(6):527-536.

202. Döring TF, Kirchner SM, Skorupski P, Hardie JIM: **Spectral sensitivity of the green photoreceptor of winged pea aphids**. *Physiological Entomology* 2011, **36**(4):392-396.

203. Ai J, Zhu Y, Duan J, Yu Q, Zhang G, Wan F, Xiang ZH: **Genome-wide analysis of cytochrome P450 monooxygenase genes in the silkworm, Bombyx mori**. *Gene* 2011, **480**(1-2):42-50.

204. The Honeybee Genome Sequencing C: **Insights into social insects from the genome of the honeybee Apis mellifera**. *Nature* 2006, **443**:931.

205. Zhu F, Moural TW, Shah K, Palli SR: **Integrated analysis of cytochrome P450 gene superfamily in the red flour beetle, Tribolium castaneum**. *BMC genomics* 2013, **14**:174.

206. **CYP450 database** [<http://drnelson.uthsc.edu/CytochromeP450.html>]

207. Lyko F, Foret S, Kucharski R, Wolf S, Falckenhayn C, Maleszka R: **The honey bee epigenomes: differential methylation of brain DNA in queens and workers**. *PLoS biology* 2010, **8**(11):e1000506.
