# Supplementary figures and images for: The genome of the water strider Gerris buenoi reveals expansions of gene repertoires associated with adaptations to life on the water
Source: BMC Genomics. 2018 Nov 21;19:832. doi: 10.1186/s12864-018-5163-2 (PMC6249893; doi:10.1186/s12864-018-5163-2)

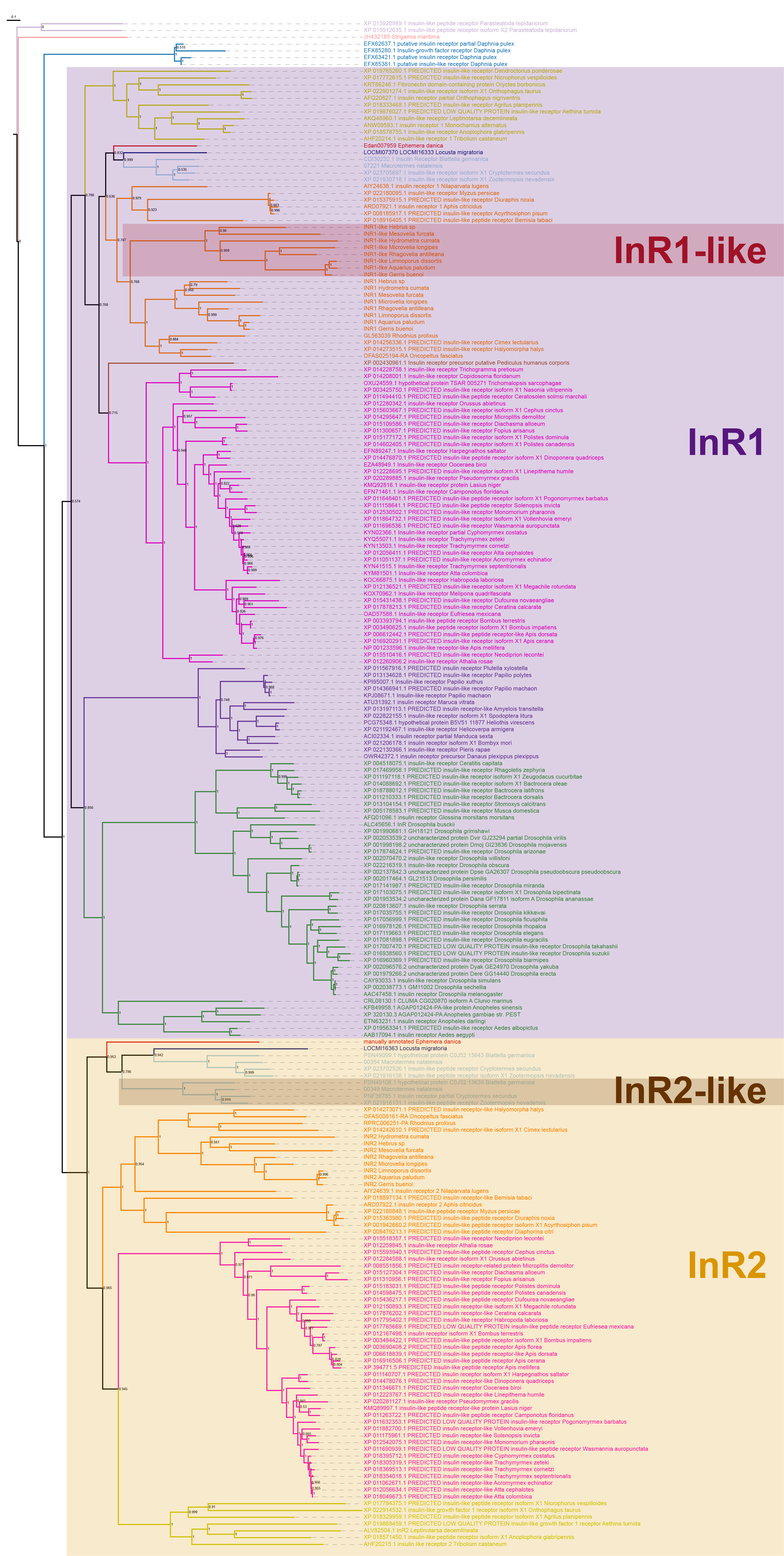

Supplement: Supplementary file 1 — Supplementary Online Information. Additional files 10 and 11. (ZIP 18100 kb) [file 12864_2018_5163_MOESM1_ESM.zip › Supplementary Figure 1.tif]

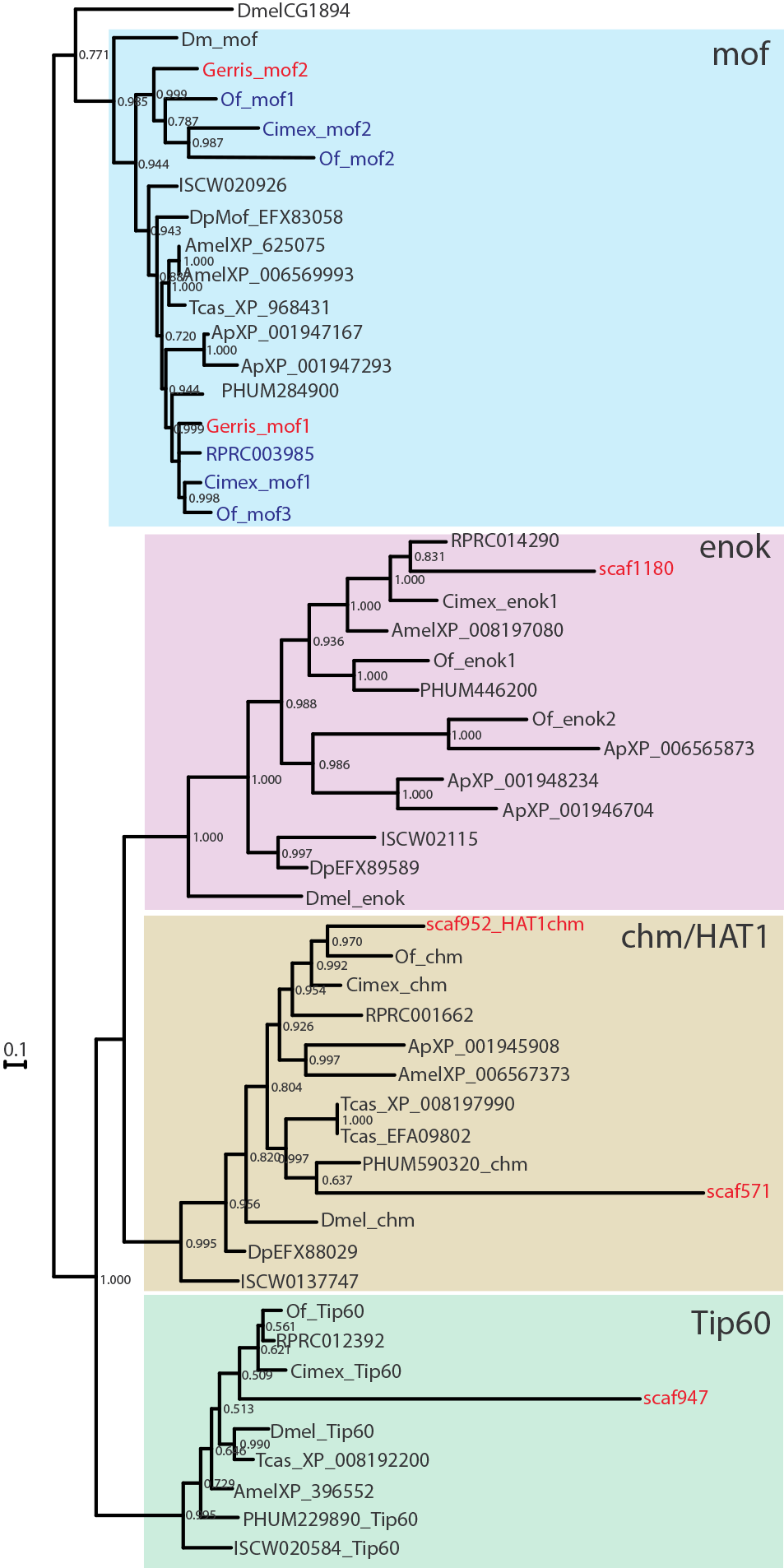

Supplement: Supplementary file 1 — Supplementary Online Information. Additional files 10 and 11. (ZIP 18100 kb) [file 12864_2018_5163_MOESM1_ESM.zip › Supplementary Figure 11.tif]

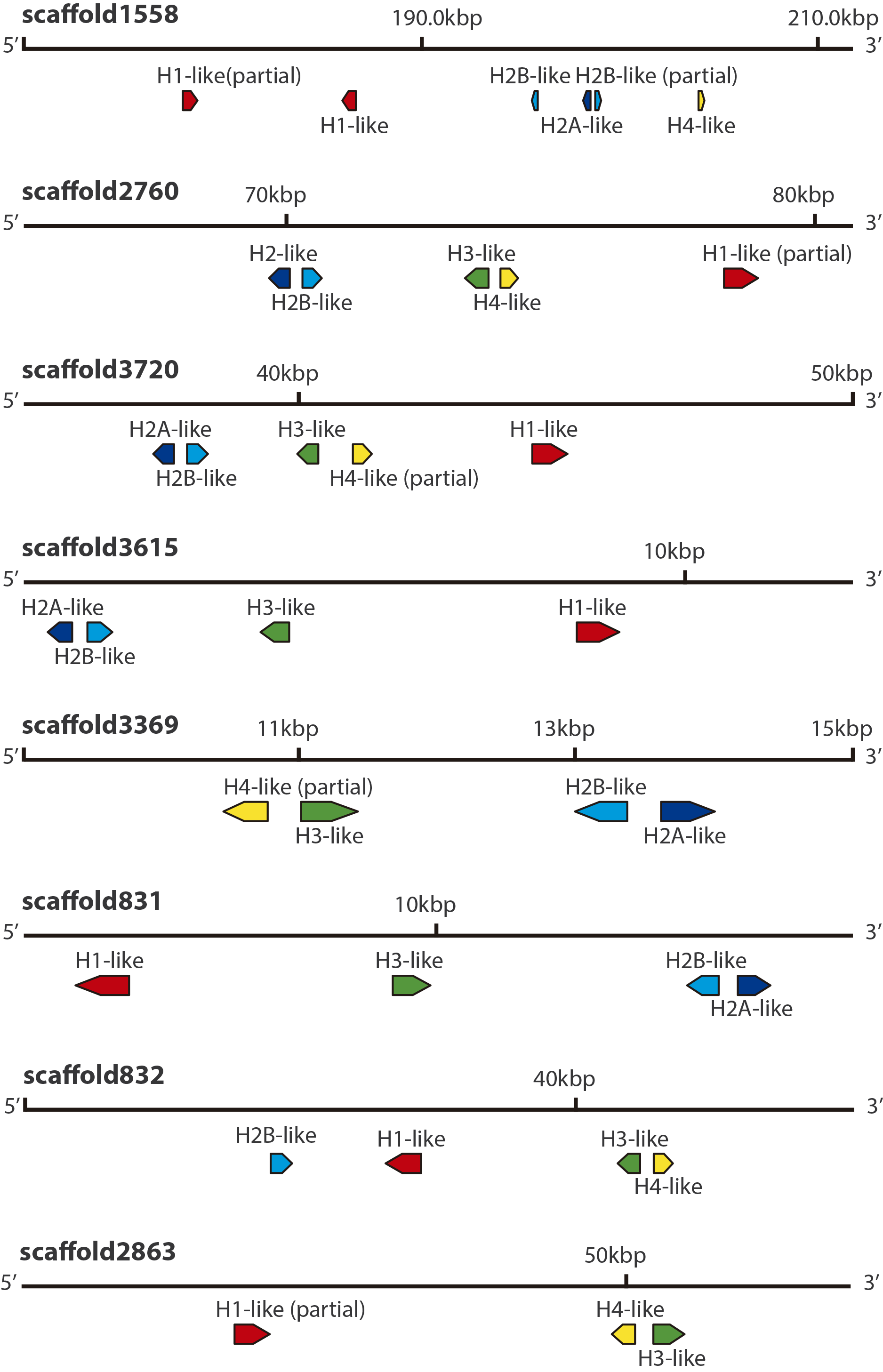

Supplement: Supplementary file 1 — Supplementary Online Information. Additional files 10 and 11. (ZIP 18100 kb) [file 12864_2018_5163_MOESM1_ESM.zip › Supplementary Figure 10.tif]

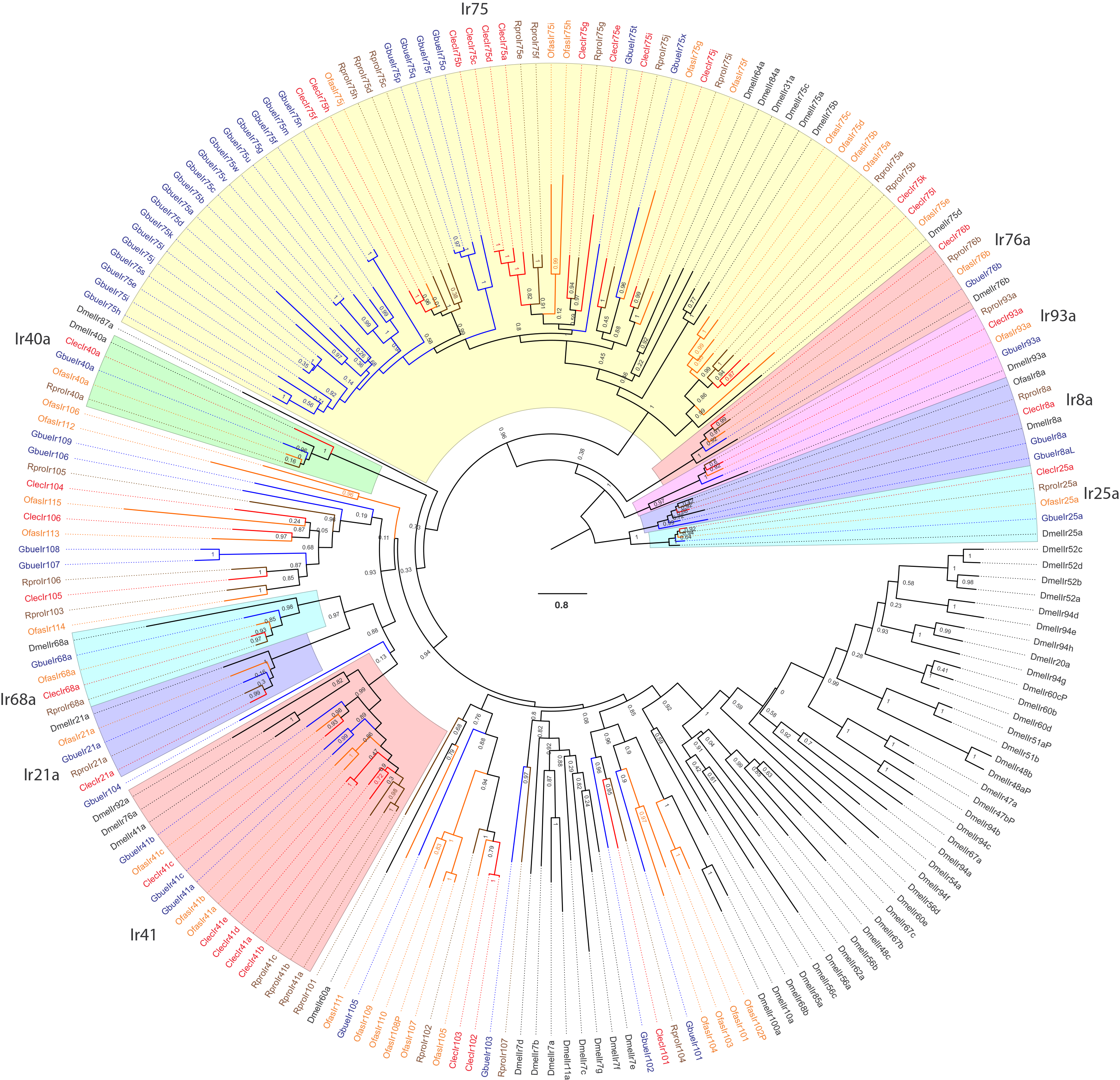

Supplement: Supplementary file 1 — Supplementary Online Information. Additional files 10 and 11. (ZIP 18100 kb) [file 12864_2018_5163_MOESM1_ESM.zip › Supplementary Figure 5c.tif]

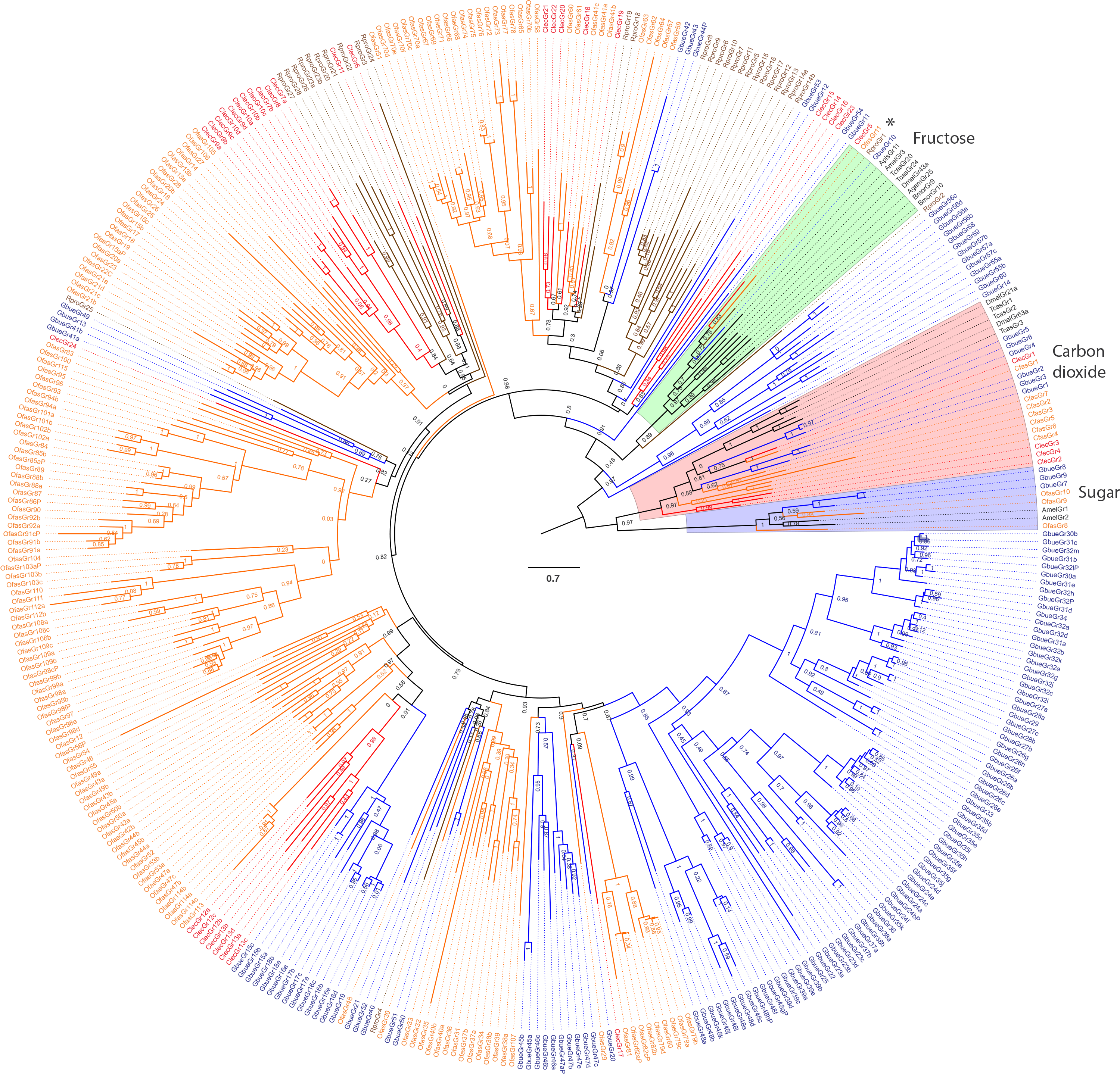

Supplement: Supplementary file 1 — Supplementary Online Information. Additional files 10 and 11. (ZIP 18100 kb) [file 12864_2018_5163_MOESM1_ESM.zip › Supplementary Figure 5b.tif]

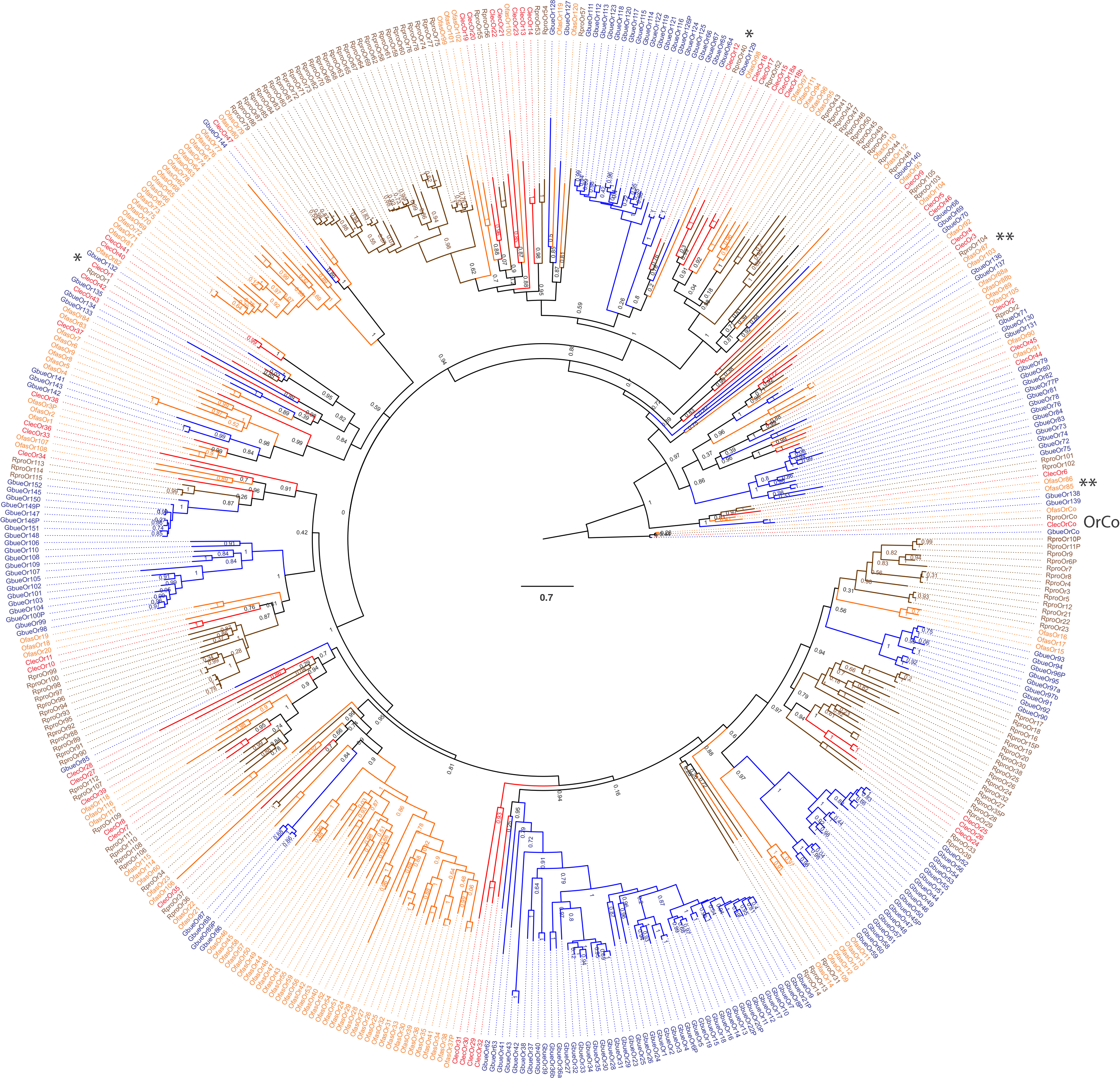

Supplement: Supplementary file 1 — Supplementary Online Information. Additional files 10 and 11. (ZIP 18100 kb) [file 12864_2018_5163_MOESM1_ESM.zip › Supplementary Figure 5a.tif]

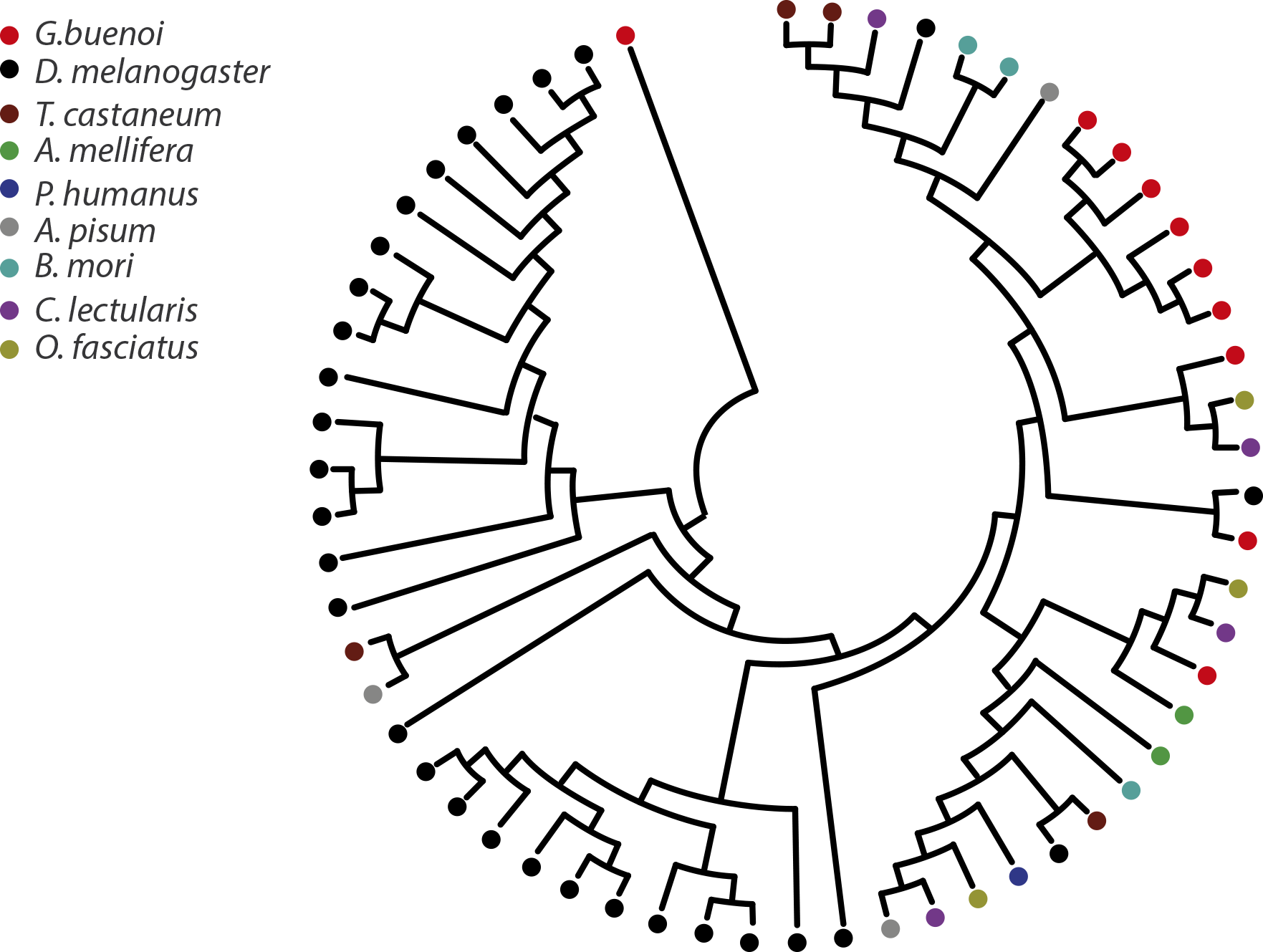

Supplement: Supplementary file 1 — Supplementary Online Information. Additional files 10 and 11. (ZIP 18100 kb) [file 12864_2018_5163_MOESM1_ESM.zip › Supplementary Figure 4.tif]

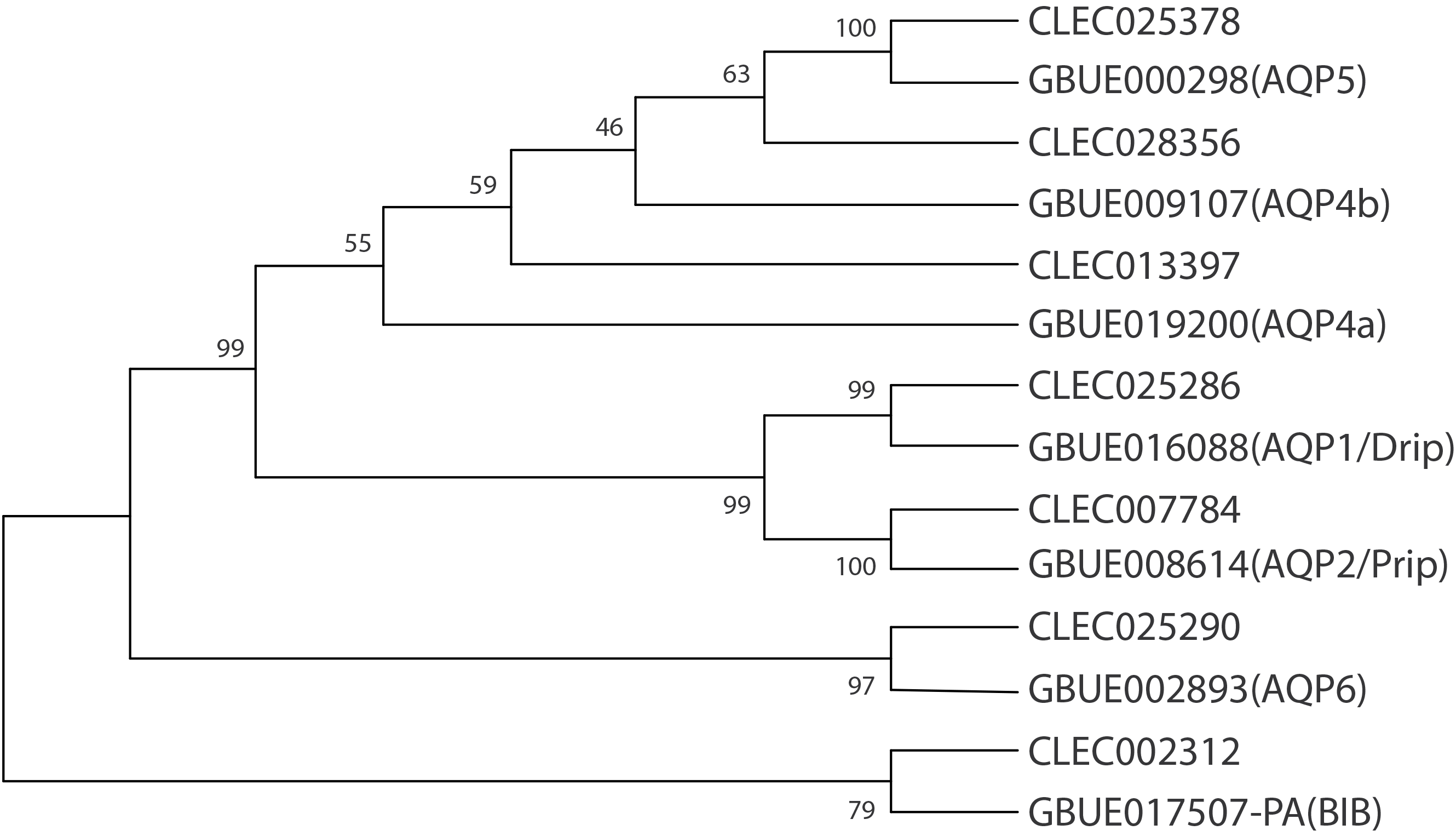

Supplement: Supplementary file 1 — Supplementary Online Information. Additional files 10 and 11. (ZIP 18100 kb) [file 12864_2018_5163_MOESM1_ESM.zip › Supplementary Figure 3.tif]

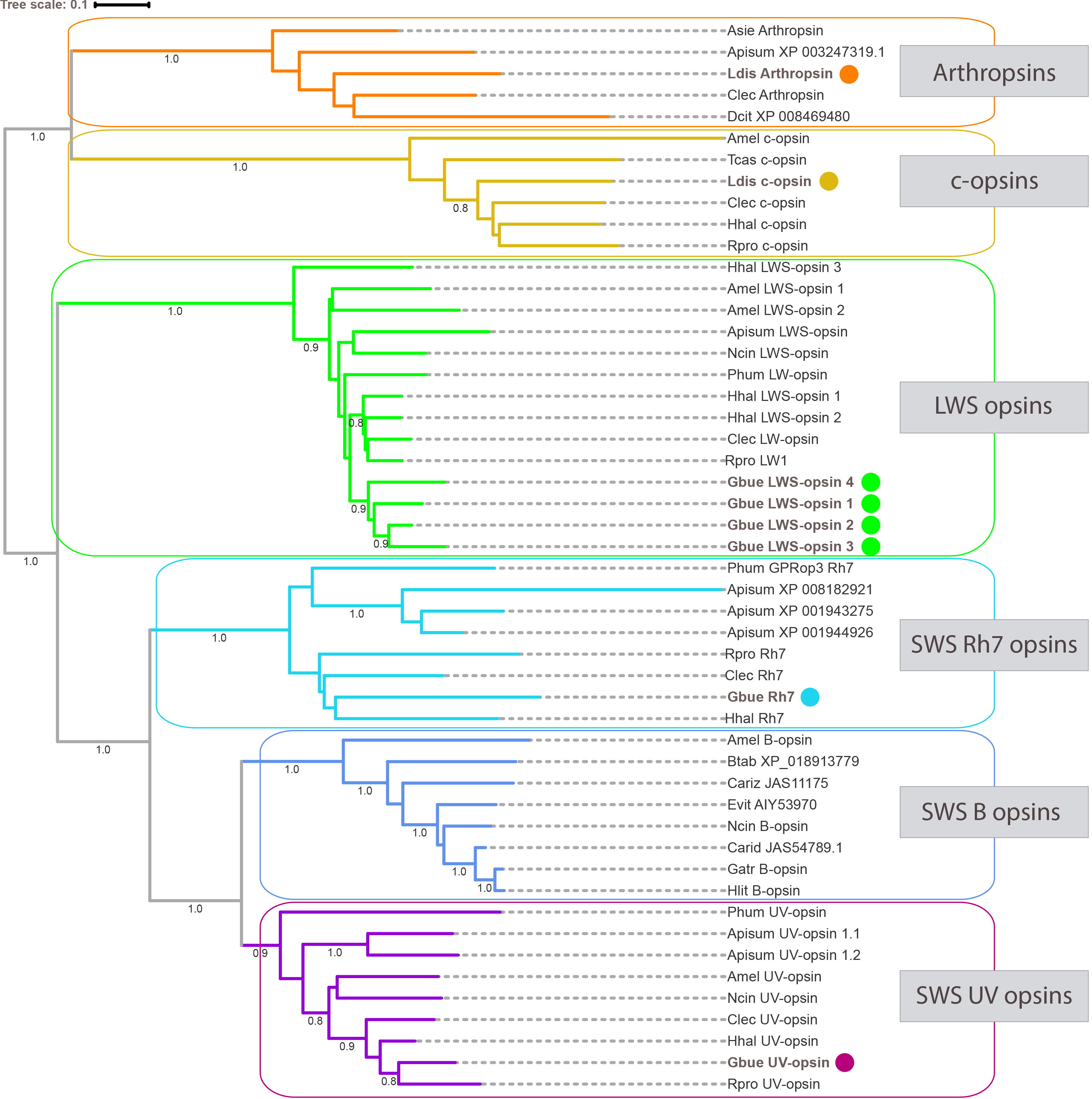

Supplement: Supplementary file 1 — Supplementary Online Information. Additional files 10 and 11. (ZIP 18100 kb) [file 12864_2018_5163_MOESM1_ESM.zip › Supplementary Figure 2.tif]

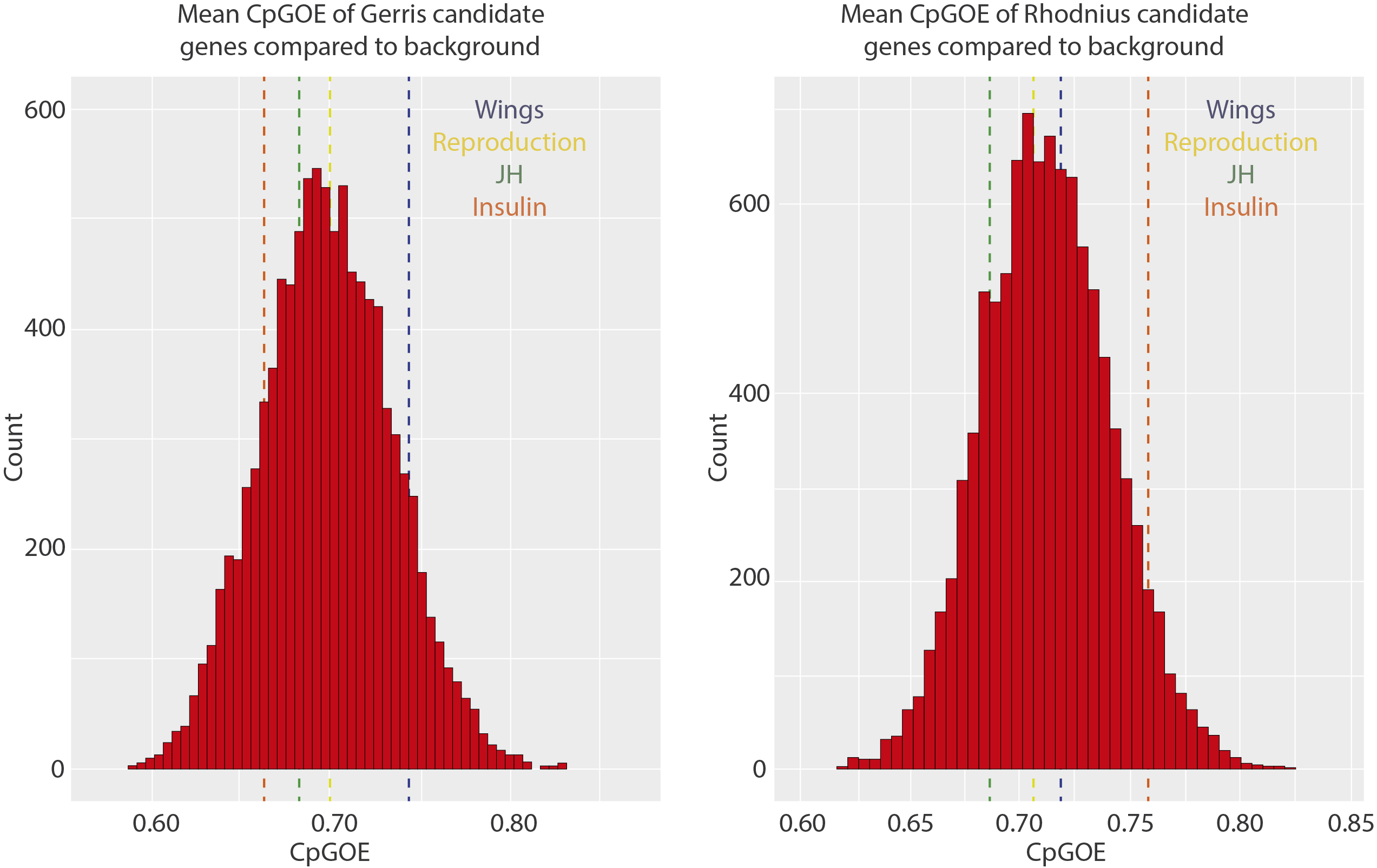

Supplement: Supplementary file 1 — Supplementary Online Information. Additional files 10 and 11. (ZIP 18100 kb) [file 12864_2018_5163_MOESM1_ESM.zip › Supplementary Figure 9.tif]

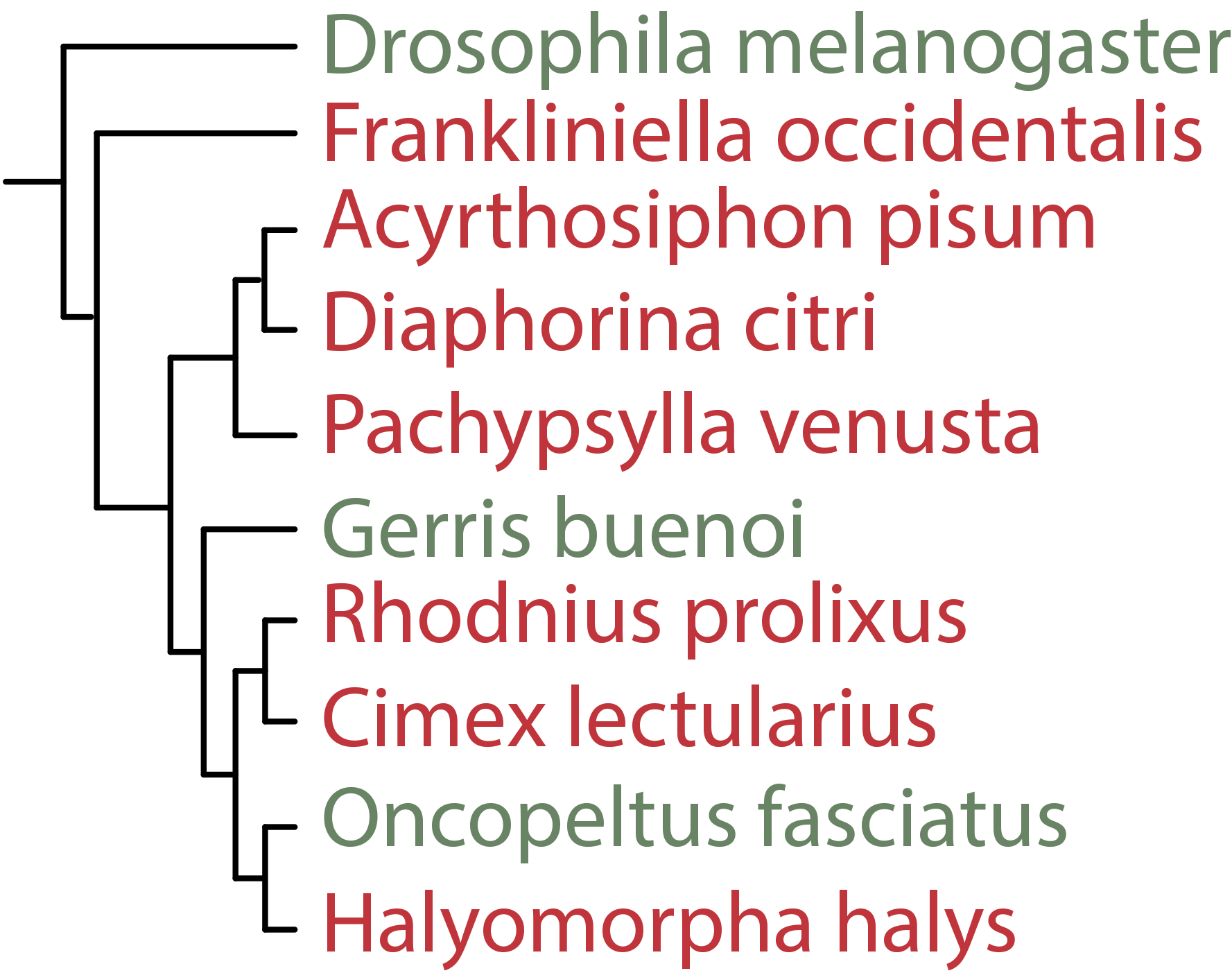

Supplement: Supplementary file 1 — Supplementary Online Information. Additional files 10 and 11. (ZIP 18100 kb) [file 12864_2018_5163_MOESM1_ESM.zip › Supplementary Figure 8.tif]

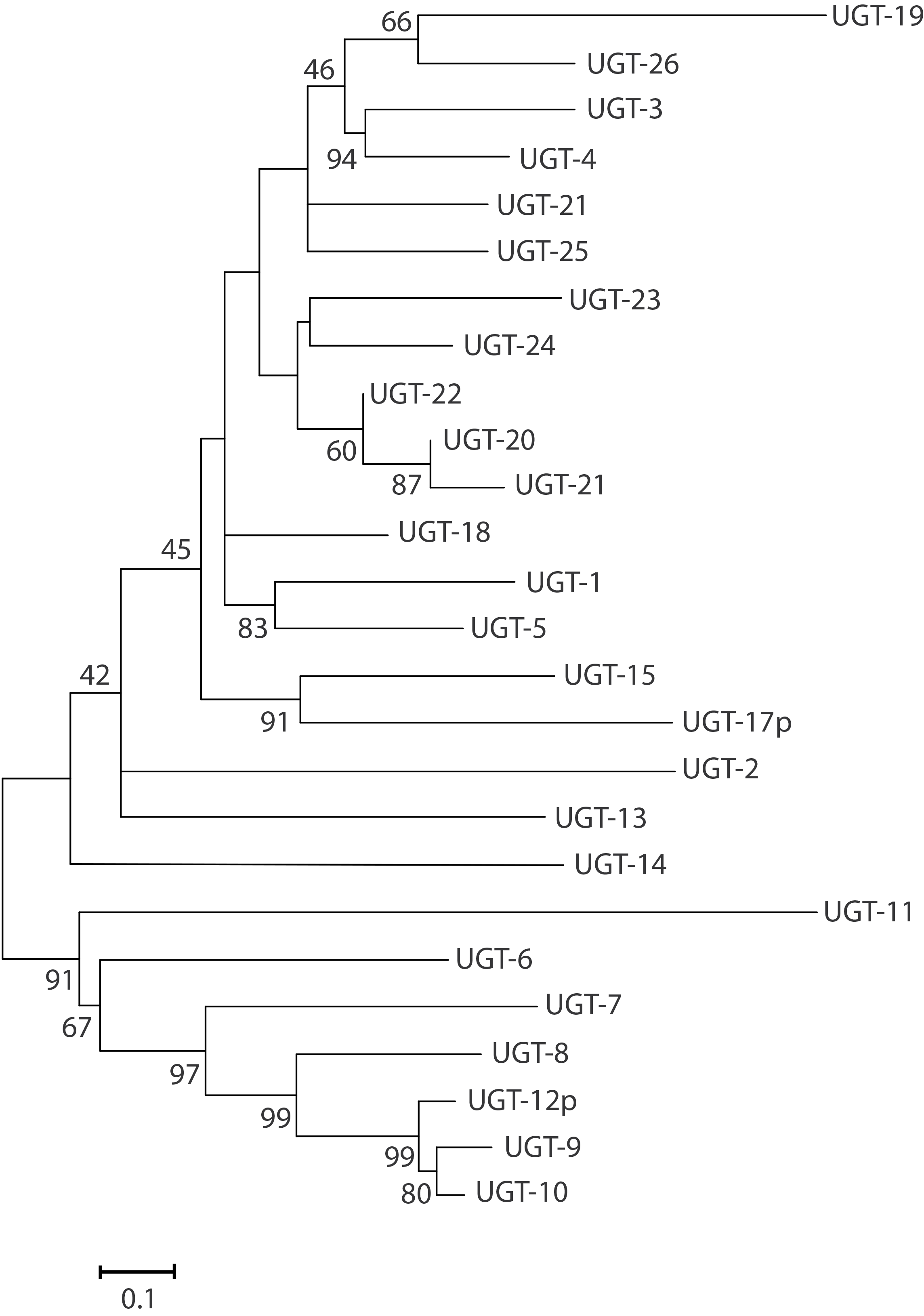

Supplement: Supplementary file 1 — Supplementary Online Information. Additional files 10 and 11. (ZIP 18100 kb) [file 12864_2018_5163_MOESM1_ESM.zip › Supplementary Figure 7.tif]

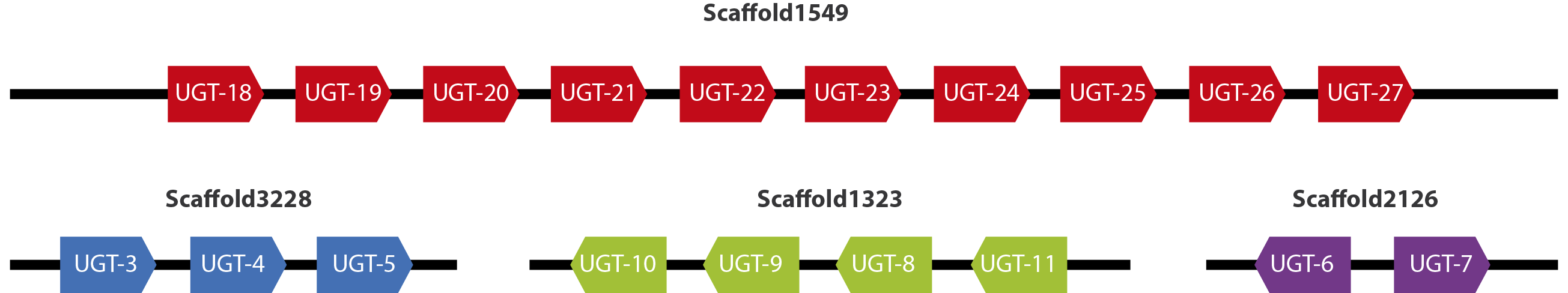

Supplement: Supplementary file 1 — Supplementary Online Information. Additional files 10 and 11. (ZIP 18100 kb) [file 12864_2018_5163_MOESM1_ESM.zip › Supplementary Figure 6.tif]
