# Supplementary material for: The genome of the water strider Gerris buenoi reveals expansions of gene repertoires associated with adaptations to life on the water
Source: BMC Genomics. 2018 Nov 21;19:832. doi: 10.1186/s12864-018-5163-2 (PMC6249893; doi:10.1186/s12864-018-5163-2)
Supplement: Supplementary file 4 — Nucleotide sequences of Insulin receptors genes annotated in Gerromorpha (Gerris buenoi, Aquarius paludum, Limnoporus dissortis, Rhagovelia antilleana, Microvelia longipes, Mesovelia furcata, Hydrometra cumata and Hebrus sp). (DOCX 40kb) [file 12864_2018_5163_MOESM4_ESM.docx]

>INR1-like_Gerris_buenoi

ATGGGTCGCTCCGTTTCGGTCGGTTTGAGTGCGGAAACCTCTAAAAATAACGACAGTTTAAGTATTTGCGGGGACGTCGAAATATGGAATCCACAAGACAAAGACGCGACCCGCTTGAAGAAATGCTCGGTGGTCGAAGGATACGTAAGACTGATGGAGGTCGACGCCGGGGAAGATTTTGTTTTTCCCGATCTGAGAGAGATTACCGGGTACCTGATGGTTTTCCGAGTGAGGGGTTTCGATTCCCTAGGTCGGGTGTTCCCTAAACTCGGTGTCATCCGTGGAAACGTGTTGTTCGCAAACTTCGCTTTGATAATTACCGAGTCTTTCGACTTGAAAACGATCGGTCTCACCTCTCTGAGTCACATTGTCAAGGGCAAAGTGTGGATCGCGTCCAATGTCAACCTCTGTTATGTCACCACTGTAGATTGGGACTCGATCGTCCGTGACAGCAGAAAGCATTACTTAGAAGGTAACAAAAGCCCGAACGAGTGTCCGGTGTGTAGGAACTGTACTAAGTCTCCTACTGGCAAGACGAGTTGTTGGAATTTGACTAGCTGCCAGGCGACGGCATGTCCGTCTACTTGTCCTAATAACTGTTACAACGAGACTACGTGCTGCCACGAGCATTGCCTGGGCGGGTGCTCGGGGGATAAAGCAGATAACTGCTACAGCTGTAAGCACTACAGGCTCGCAGACAAATGCATTAAGAGCTGCCCTGCAGGCACTTACGTGTACGCAGGGTGGAGATGTACTAATGAATCGGAGTGTTACGGGTTCCCCAAGACTCCTTCTAACAACCACTGGAAACCTCACGACGGACAATGTGTATTAGACTGTCCATTCGGTTACCACGAGCAGGAAGTCTACATCGGCGGTGCAAAGAGGTTCCAATGTCAGAAGTGTGAAGGTGGCTTCTGTCGTAAGGAATGTAACGGCGCTTCCATAAAGAACAAAAATGATTCTGTACTCATGTCCGGGTGCACTGTGGTACGAGGCAGTGTTGTAATCAGCCTGTCCGGGAGCGGCAAGGACATTATCTCCGAACTCGAGAACAATTTAAACACCGTCGAAGAGATAACCGGCCTTCTCAAGATTACCGACTCTTACCAACTGACTTCACTCAACTTCCTGAAGAATCTGAAATATATCCATGGAAACGAACGCTATAAATCCAAGTACAGCCTCGTCGTAGAAGGTAATAAAAATCTTAAACTGCTTTGGGATTTCGACAATAAAAGAACCAAACTAAAAATAAACGGGTTGGTTTATTTTGGAAATAATCCGAAGCTATGTGTCAACGTAATACAGCAGCTGCTCGACAAAGCCGATATACCGGCTTCAGATGCTTTTTACCCTAATATGACTAACGGTGTAAAAAGCGTATGTGACCTTAAAAACCTTACCACTTCGTACATTAACGTAACTTCCAACGCCGTCAATATTACATGGAAAGCCGAATATCACGACCTGAGGAAAGTAATGGGTTACGTTATATATATAAAAGAAACAATTTTTAAGAATGTCACCGTCTTTGACGGGAGAAACGAGTGTGGTAGAGGAGGTTGGACTGTTTACGATGTGGACGGTTCCGAGGCAGAGTACAAGATCGTCAGTGTCAAGCCCTACACTAGTTACGCTTTTTATGTCGAAACAGTGACCATCAATGCGGATTTCGGAGCCAAAAGTGAAATAAAATATTTTAACACGTTGCCCGGTATACCTAATCCACCTTCTTACGTGAATGTAATTACCAATTCGAGTTCGGAGCTTGTGATAACGTGGCCACCTCCGTTGGAGCCTAACGGTAATCTTTCTCATTATTTGTTATCGATTTACGAATTAGACGACAAATCCACTTTGAATAATGCCGTGGAGAATCACTGTGAGGGTAAAGACGCATATTTCGTGCCGAGTAACAGTACGAGGTACTCTGGATCCAGCGACAGGTTTAACCATTATCTCGATCGACATTTCACCAGTCAACTCTCCGACGAAGAGAAAGCCCGCCAAACTCAAGTGGCCATGCTTATCGAAGATAAGATGAACAACTACATCTATGTTAAAAACCCAAAAAGGCCGAAAAGAGACACTATATTTGTAAATGAAGCGGAACCTGTTATCTTCAATCATACCCTGCAGGAAACTACTGTTAAAACTCAGATTGACCATAGTAACAAAACTCAGTATAAGACCAACTATACTCCTGTTAGTGAAAATCAAAATCTCCCTATCATACAAAGAGACGATACGATTAAGAAAGTTATATTAGATCACGAAAAGATAGATAAAAGCATCACGTTAAAAACTGTTAAAAATTTGAAGCATTTCACTAAATACCGTGTAGAAGTTCAAGCATGCCGTCAGCGGACACAAAAAGAGCTGGATTCCTCTCTAGACAATCACTGCAGCGATTCGGCCAGTGAACTGATTAAAACATGCCCCTCTGTGACTGCCGATTCCATTCCCAACGAGAGTCTGTCACCCAAAGTCTTGAATGAGAACCCCGGGACAGTCGAGTTGAAGTGGGAAATTCCTAAAGACCCCAACGGAATTGTTCTCTTGTACACCGTCGAGTATAGGAGGAGTGGGTACAACCCCATTACCGAGATCGTCAGCCAGAAAGAGTTTTTAAAATCCAATAGTGTGTATATTTTAAAGAAACTAAGACCCGGTAATTACAGCGTCCGTGTGAGAGCGAGTTCTTCCAGCGGTGTGGGACCTTACACAACCTCCAAATATTTTTACATCAAAGACGACTCTTCTATTCACTCTTATATTTATTTAATTTTAGGCACGTTATTGTTTATTTTACCGCCTGCAATCGCTTACTGTGTTTATTACCTCAGGAAGACCGAGAAAAACAAACTTTTTATATCCGTAAACCCCGAGTACTCTCATAAAACTTACGTCACAGACGCCTGGGAGATACCTAGAAACCACATAGACCTTATCAAAGAAATAGGTCAGGGCTCCTTCGGCATGGTCTACGAGGGCCTGTTAAACAGCAAGCCTTGTGCCGTCAAGACCGTGAACACACAGGCTTCAGACGGGGAAAAGAAAGAATTTCTAAACGAAGCTTCCGTGATGAAAGCTTTCGATACGTATCACGTTGTCAAGTTGCTAGGAGTCGTGTCCCTCGGGGAACCGGTCTACGTCGTCATGGAACTGATGCCCAACGGCGATCTGAAATCGTTCTTACGCTCTCACAGGCCGGACGTATGTGACCCTCACCTCCGTCCCCCGTCAGTCAGAAGACTCCTCCAGATGTCCGCTGAAATAGCCGACGGTATGGCTTACCTCTCCGCCAAAAAGTACGTCCACCGAGACCTCGCCGCCAGGAACTGTATGGTCGCCGAAAACCTCACTGTTAAAATCGGGGATTTCGGTATGGCCCGAGACATCTACATCGGGGATTATTACAGGAAAGGCACTCGTGGGTTGATGCCCGTGAGATGGATGGCTCCGGAGAGCCTCAGGGACGGTATATTCCTGTCCGCTTCGGACGTGTGGAGCTACGGGGTGGTGCTTTACGAGATGGTCACACTGGCGGCTCAACCTTACCAGGGTTTGACTAACGACCAGGTGTTACGCTACATCATGGATGGGGGAGTCATGGAACGACCTGAGAACTGTTCTGACAAGATGTTCGATCTCATGAGGAGGTGCTGGAGGCAGAACCCCGGAGACAGACCGGGATTCCTGGAGATCGTGGAAACTCTCCACCAAGACGTCAGCCTAAACTTCCACGCTGTCTCGTTCTACGACAGCGACCAAGGCAAGCAGCACCGCAGG

>INR2_Gerris_buenoi

ATGACGTGGGTTGTGATGGTGTTGGTGGCCTGGCAGACCGCTTGCTTGGCGGCCATCACGGGCGACGGAGTTACAAACAAGGATGGCGAAGTTGCCAAAAAGACCGAAGGAGTTTTAACCAAGGGCGACGGAGTTTGTTCTTCCATAGACATTCGCAACGGCGTCGAAAACTTTAAATACCTTAATGGTTGCCGAGTCATCGAAGGCTACCTTCAAATTGTACTTTTAGAACATTCCACGCCCGATTTATTTGAGAACAAAACTTTCCCGGAATTGCGCGAAGTGACCGGATACGTGATTTTTTACCGAGTGAACGGTCTTAGATCTATAGGCCAGTTATTTCCAAACCTGAGTCTGATTCGTGGAGAGCGGCTGTTCATCGACTACGCGCTATTAATTACACAGATGCCTGTCCTCAGAGAGATTGGCCTAGTGGGATTAACCACCGTCCTCCGGGGATCTGTCGCCCTCTTTAATAATCCAGAACTGTGTTTCACCGACACGGTGGACTGGAGCCGAATAGTCAAGGGAGAGCCTTTTCTTTATAGAAAGACAAATCTGCCATGCCCTGCCTGCCCCAAGAGCTGTCCCACCAGTTGCTGGAACAGTACTCATTGTCAGGTGATAGATAAAGTGATTGCTGAAGCAGAATGCAACGAGCAATGTATTGCTGGCTGTACAGGACCTGGTCCCCACCAGTGCACTGCTTGCAAGCACCTGTTGCGCCAAGGAGTCTGCACCAACACCTGTGATGATAATGAGTTCACTTATCAGAAGAGACACTGTGTCACTGAAGAAAAATGCAAAAATATGACATTTTCTGGAATTGGAAAACGGTTTATTTTAGAAGAAAGTAGGCCTTGGTTTACTTGGGATAGGACATGCATACAGAACTGTCCAAAAGACTTTGAGAGGGATGAGAAAAAAGGATGTCGTAAGTGTTCTGGTGCATGTCGCAAAGTGTGCCCTGGACTGGTTGTTGACAGTACACAGAGTGCTCAGACACTTGAATTCTGCACACACATTACTGGTAACCTAGAGATACAGATTCAAAGCAACTCCTCAGTAAATATAACAGATGAGCTAGATAAGTTTCTTGGTTTAATTGAAGAGATAGAAGGCTACCTCAAAGTCATTCGTTCGTTTCCATTAGTCAATTTATACTTTTTTAGAAATCTTAGGGTTATCAGAGGTCTTCAAACTTCATCCAGTAGGAAAGAACTACTTCAAAAAGACAACCCTCCCCACAATTATGCATTTAGAGTGTTAGATAATCCAAACCTTCAGGAACTGTTTGACTGGAATCGTCCTGCTAGAAAAAACTTCACCATTGAGAACGGTCGTATTTTCTTTCATCTTAATCCTAAACTATGTTTAAAGTATGTTGAAGAGTTTATCCAAATTACTAATATCACAAATATTACTGATCTAGAAGTAGATAAAACATTTAATGAAAAAATGTTTGCATGTAATGTTAAAAATATATCTGTGTATGTAAGTGTTAAGACATCTAATAGCTTTGTAGTTGGGATAAAATCAGACATTTTAAATATTTCCAGCTCTGAACAAGATTGGGTAAGAGTTTTAGTTTATTACAAAAAAGCTCCATCTATGAATGTGACGTCGCATTATGATGATAATGTGTGTAAGGACGATGATTGGAAATTTAATGATTTTAAAACTGAGTATGGAGATGATGGAGATGGGACTCCTCTGATTCGTCCCCAGTTAATAACCCAGCTTGAACCATTCACTCAATATGCTTACTATGTAAGGACTTACAGCTTAGGATCTCCTATTCATTATGTTAGGACATTGCCAACTAAACCATCTTCTCCCGAAAACTTACAAGGTTACAGTGACTCAAGTGATAAAATTATTTTTGGTTGGTTACCTCCTTCCCACCCGAATGGTCTTCTTAAAGAGTACATTATTAGAATAGTTTTATTAGAAGATGACCAAGATCTACCGAAATTCAGGAATTATTGTGCCAATCCTGCTCATTTCACTGGGAATACAACTCCACCTCCGGACTTTAAAACTATTATTAAAGAAGACAAGAGTAAAGAGTTGACAATACCTAAAATGTTATGTGACCAATCTGATTTTGACAAATTCGGTAAAATTTCTGAATCATTTTTACAAGACCATCATATTAAATCATGTGAAAGATCTATGTATACGATAATAAATGAAAATGTACTTAAGCCGAAGTTTTCATCTAGATCAAAAGAGAGACGTTCCACAAAAAAAGCTAATCAAAATACAAGAGAGAATAAAGACGATGAAGATGTACATAGCTTTGGCAAAAATGGCACCACATACAATACTGATGGCACTATAAAAACAGAAACTATCTCAGTGAGTAGTAATGTTACGAGTTATACTCTTACAAATCTTATGCATTTTGGAAGATATCTTATAGAAATAAAGGTTTGTAGAGAAAAAGCTCCTGAGGAAGACATGAAATATTTTTTGGATTCCCAGTGTAGTGACATGTCTTTTATAACATACCGCACTCTAAAATCCTATTCAGCTGATATAGTGGATCATAATAGTATTGTAATACAAGTAACTAACAAAAGTGCTACAGTAAAATGGAAAAAACCCAGTAGTTACAATGGTGTTATTCTAAACTACATTTTAGAATATAAAAAAATAGATGAAGAAAATCCTAATTTACATTTTAAAGCAACTGAGTGTATTGCTGCATTTAGTAATAAATCATCAATTACTTCATTTGCACTGACAAATTTGAACTATGGCGAATATGCAGTTAGGATTCAAACCATATCCCTTGCAGGAAAGAGCCCTTTTTCTGGACCTGTTAATTTTTATATTGTTGATGATTCTTATTCAAATTGGATGGAAAAACTAGCTGTAATAATAGTTGTAACACTAGTATGTATATTTGGAGCTTTACTCAGTGCTAAAGGAATGGTTTATTATTATCAAAAAAAGTTAAAAGAAACTAATATATTAATCGAGACAATTAATCCAGAATACACAAGTGTAAACGGTTATATCGAAGACGATTGGGAGTTATCTAGAGATGACATTGTTTTAGTTAATGAAATAAAACAAGGGAATTTCGGAATGGTTTACGAGGGCTTACTGTTGCCTGAGAATAGAAAAGTGGCTGTAAAAACAGTCAAAGAAACTGCACCTCCTAGAGATAATTATGATTTTCTCAATGAAGCTAATGTGATGAAAGCTTTTAGTGGTGCACATCATGTGGTCAAACTGTTGGGTGTAGTTTCAAAAGGAACTCCTGCTCTTGTGGTTATGGAGCTCATGGTTTTGGGTGACTTAAAATCTTTTCTCAGACTTAGTAGAGATTCTCCTCAGTACCCTCCTCCATCAGCCTCAAGGCTCAACCTTATGGCTGCACAAATATCGGATGGAATGGTCTATTTAGAAGCTCTGAAATTTGTCCATCGAGATCTTGCAGCTAGAAACTGTATGGTTTCAGAGGACTTAACTGTGAAAATTGGTGACTTTGGAATGACTCGAGATATCTACGATACTGATTATTACAGAAAAGGAAACAAAGGGCTTTTACCTATCAGATGGATGGCTCCTGAAAGTCTCAATGATGGTGTATTTACCAGTTTTTCAGATGTCTGGAGTTTTGGAGTTGTTCTTTGGGAAATGGTTACATTGGCAACTCAACCATACCAAGGTATGTCCAATGAGGAAGTATTGCAGCACGTAATATCAGGAAACAAGCTTGACACTCCTGTTTACTGTCCACCTCTCCTAAAAATGATCATGCACTCTTGTTGGAAATGGAAGCCTAAACTTCGTCCTACATTTGTTCATATCCTCAATCTACTAGACCCAACCCTCAACAATGAATTCCGCTCAAATTCATATTATCACAGTGTTGAC

>INR1_Gerris_buenoi

ATGATGAAACAGGAGCTTAGGCGACATGATAAAACTTACAGTATCAAATGGCAAAGCGTTATGTTTGATCATTTCATCCAAGGTTCCAGTTTAATTAAAACGAGTGGAGTTTTATTTTTTGGAATTCTTTTATTCAGCTCTCTTGTAAATGGTTCCCATAAAGCAGATATTCCTTCGGCATTACGTATATATTCTGAAGTCGTTCATAATGACGAGGATGCCTCAGTGTGTCAAAGTAAAGACATTAGAAACACTGTGGACATGTTTGTTGAGCTTCAAGGATGCAAAGTCATTGAAGGATTTGTACAGATAGTGTTAATTGATAAGGCTGAACCAGCTGATTATGTAAATATATCGTTTCCTGAACTCCGTGAAATCACCGGTTATTTAGTTTTATATAGAGTCCAAGGTTTAAAATCTCTTAGCAAATTATTTCCTAATTTAAGCGTTATCCGAGGAAACACATTATTTTTGGACTATGCGTTGGTCGTGTACGAAATGCTACAGTTGCAGGATATTGGTCTATCATCTTTGACGAATATTTTAAGAGGTGGAGTTTACTTTGCCAAAAACCCGATGCTATGTTTTGCAGAAAGCATTGACTGGGATATAATCGCTCCTCATGGCAAAGGAGATCATCATATCATGGGAAATAAAAATAACTTTGAGTGCCCGTTATCTTGCTTCGCCTGTAAACATTTCCTTTATAACAACGAATGTGTTGAGAGATGCCCTCAAGGGACGTATGAATATTTGAATCGTAGGTGTGTTACTGATAAAGAATGCTTGAACATGCCGAAGCCTCGTGAAAACACCGACCACAACACTAAGAATAAACCGTGGAAACCTTTTAAAAAAGAATGTGTTCTCGAATGTCCACCTGGTCATGTTGAAAAACAGGTGTTCGTTAAAGGAGAGATGAGGATGGATTGCGAGAAATGCAAAGACAATATGGTGAGAGAATTGGAAGACAATTTCAACACGATCGAAGAGATTAATGATTATTTGAAGATAGTAAGATCTTTTCCTCTTATCTCTTTGAATTTCCTCCCTAATCTGAGGGAAATTCATGGTAGAAAACTTGAAAGTTCAAAGTATGCTTTAGTTGTTTTGGATAACCAAAATCTTATTGAACTGTGGGATTGGAATAATCGTTCGACTGGCTTGACCATAGGCAATGGTCGATTATTTTTTCATTTCAACCCAAAATTATGCTATGATAAAATTGAGGAGTTAAGAAAGAAGACTAATTTGTCAGCTCACTCGGATTTGGAAATTGCCAGAAATTCCAATGGTGATAAAACAGCATGTAACGTGGATAAACTCAATGTAACTGTGTATAAGAAAACAGCAGTTGCAGCTTTAATTAAGTGGAGACAGTTTGAAAATAACGATCCTAGAACTCTTTTGGGTTATGTTGTTTATTCTATTGAAGCGCCTGCCAAAAATATTTCTATATATGATAGACGAGATGCATGTGATGATGAGTGGAGAGTTGACGATGTCTCTGTTCAAGAAGATACAGATGCATCTGAAGAAAAGGAAGTCAATCATATTATAGCTCAACTCAAACCCTACACTCAGTATGCGTTTTATGTTAAAACCTACACCATCGCAACAGAAACGAGGGGCGCTCAGAGTCCCATAAAGTACTTTCGAACCGAGCCAGACATTCCGTCTGAGCCACATGGAATTTCAATACAATCCAATTCGAGCTCCGAATTAGTTCTTCGCTGGAAACCACCTACTCATCCGAATGGAAATGTAACTCACTTTATAGTTACGGCAGTGCTGGAGCCTGATAATGAAGAGGACGTGGAACATCAAAATTTTTGTTTAGAACCTTTGCTATTCCCAGACATTAGAAGGGTACCAAGTAAAAACCCCTTTCAGGATATGGTCGGGCCAAAAGATGAAAGGAACGGGTCTACTGATAACGAATCATGCTCTTGTAACCAAAAGTTTATTGACAATCAACTAAGGATGAAGGAAATTGCATTTGAAGATTATCTGCAAGATAATATTTTTATTAAAAGAGAGTCATATGAAGAATATCCTAATGGGAAAGAGAAGCGTGAAATACCGAAATCATTCTCTTCAGTATTTGACGAGTCCGATAATTCCATTGCTGATAGCTTGTTCGAAAATGAAAGTTCTGATAAAGATAACTACAGTGTAAATGGTGTTTTCAAGCGCTTCCGTCAAATTGTATTTGGAGAAAATTATTCAATCGTAATAAGAAATCTAAAGCATTTTTCTAAGTATAGAATTTCCATTCAAGCGTGTCGAGAAGTCGAAGAAAGTGAAGATAAATCTAAGATTCAAAACTGCAGTAAAGAAGGAGTTATTGCTGGCAGAACATTACCCATGAGTGACGCTGACAACTTGGACGGTGATCAAGTTTTTTGGGAAGTTTCAAATACAACTCATGGGATAGTAAAATTGAAGTGGGAAGAACCAGTCAATCCGAACAGTATGATATTAACTTACCAAATTGAATATTGGCGCACCGATATTGAAAATTACAAACCGACAGTTGAATGCATTTCAAGAAACCAATATAAAGCTTTAAACAAAGGATATATTTTAAAAAATCTCAGCCCTGGCAACTATAGTCTCAGAATACGAGCTTCGTCGTTGTCCGGGAATGGAATGTACACCAAAATTAATTACTTCTTCATTGAGGAAGATTCAGTATCTCCAGTATTACATACTATCATTATTGTCACTATAATTATGTTATTTATATTTATTATAATTATACCGTTTATTGTATTATATATTAGAAGACATATTGAATATGGCCGCAGTATAAAGCTTATTGCTTCAGTAAATCCCGAGTACGTTCCTTCTGGTAAAGTTTATACTCCTGACGATTGGGAAGTACCTCGAGAGAATGTAGAACTAATAAAAGAAATCGGTCAAGGCTCGTTCGGAATGGTTTATGAAGGCACACTTAAGGACACCGGCATTGAAAACTCAAGACCATGTGCTATTAAAACTGTGAGCTGCAATAGCAAGACTCAAGAACGTGATAGAATTGAATTCCTTAATGAAGCATCTGTCATGAAGGCTTTCAATACCCATCATGTAGTGAAATTGCTAGGTGTGGTTTCTCAAGGGCAGCCTACGTTAGTGATAATGGAGCTTATGGCTCATGGAGATTTAAAGGCATATTTAAGGTCTTGTCGTCCAGATGGAGGATACTTCCCTCAGACGCCACCAACCTTAAAGGACACCCTTAGAATGGCTGTTGAAATAGCAGATGGAATGGCTTACCTCGCTGCCAAAAAATTTGTGCATCGTGATTTAGCTGCAAGAAATTGTATGGTCACAGACGACCTTACGGTTAAAATCGGTGACTTCGGAATGACCCGTGACATTTATGAAACTGAATATTACAGAAAAGGAAGCGTTCGTTGGATGGCACCTGAATGCCTTAAGGATGGCGTATTTTCCAGTCTCTCCGATGTATGGAGCTATGGTGTAGTTCTCTATGAAATGGCCACATTAGCTTCTCAACCTTATCAAGGCCTTTCTAACGAGCAAGTATTGAAATTTGTCATTGACGGAGGTGTTATGGATAGACCTGATAACTGTCCAGACACTCTGTATCACACAATGTTGATGTGCTGGCAACAAAAAACTAGGAGCCGACCCTCTTTTATGAACCTCGTGGGCATGTTAAAGGAGCATTCGAACCCTGACTTCGCCGCCGTTTCATTTTACGACAGTGAAGAAGGACAAGAAATCAGGGGAATCGAAGTGACAGAAGAAACTCCCTTGTTCGTTTCGAGGGTTATCGAAGACTTTTCTCTCAGCGACGATGAATCGAGCAATCCTATAAAGAATTGTAGTAACACCGGTGACGAGGATGAGATTGACGACGCAGATGATTACGGAGATATTAGTTTAAGAAACACGAGTGACAGTTGGATCCGTAGTGAAGGTCCACCAGGCGGTGTAGAAGGCTTTGGAGTCCCCCATATGGTATTGTTAAAAAGTAATTCGACGGAGAAACAAAGTAAAGCGAGAGGAGACATCGAAGATTTTGAAATAGCACCGTCGAGTGTGAATTCAAACAGAAACAATGGTCGGATGACTCCAAACGGTTGTATTACGTATAACGATGTAAAAACAACCACGTGTTAA

>INR1-like_Aquarius_paludum

ATGGGCCGCTCTGTTATAATTTACCCCGGCGTTGGTTTGAATCCGGAATCTTCTAAAAGCAATGACGGTTTAAATATTTGTGGGGACATCGAGATATGGAGTCCACAAGACAAAGACGCGATCCGCTTGAAGAAATGCTCGGTGGTTGAAGGATACGTCAGGTTGATGGAGGTCGACGGTGGGGAAGATTTCAATTTTCCCGATCTCAGAGAGATCACCGGGTATCTGATGGTTTTTCGAGTCAAGGGATTCGATTCTCTTGGTCGGGTGTTCCCCAATCTAGGTGTCATCCGTGGAAACGTGCTCTTCGGAAATTTCGCTTTGATAATTACCGAGTCTTTCGACTTGAAAACAATCGGTCTCACTTCTCTAAGTCGCATAATTCAAGGCAAAGTTTGGGTCGCGTCCAATGTTAACCTGTGTTTTGTCAACACTGTAGATTGGGACTCGATAGGCCCTGTTAGCAGAAAGGATTACTTAGAAGGCAACAAAAGCCCGAACGAATGTCCAGTGTGTAGGAACTGTACAAAGTCTCCTACTGGAAAGATGAGTTGTTGGAATTCAACAAGCTGTCAGGTTACAAGATGCCCGTCTACTTGTCCCAATAACTGTTTCAATGAGAGTACATGCTGCCACGATCATTGTCTGGGTGGGTGCTCCGGTGATAAAGCAGACGAGTGCTACAGCTGTAAGCACTACAGGTTCGGTGATAGATGTGTCAAGAGTTGCCCTGTAGGCACTTATGTGTATGCAGGGTGGAGATGCACAAATGAATCGGAATGCTACGGCTTCCCCAAGACTCCATCGAATAACCATTGGAAACCACACGACGGACAATGTGTATTAGACTGTCCTTCTGGTTACCATGAGCAGGAAGTCTACATTGGGAGCGCAAAGAGATTCCAGTGTCAAAAGTGTGAAGGTGGCTACTGTCGTAAGGAATGTAATGGTGCTTCCATAAAAAACAAAAATGACTCTGTACTCATGTCAGGGTGCACTGTCGTTCGAGGCAGTGTTGTAATCAGCCTATCCGGAAGTGGCAAGGACATTATCTCCGAACTTGAAAACAATTTAAACACTATCGAAGAGATAACTGGCCTTCTTAAGATAACCGACTCTTACCAACTCACTTCACTTAACTTCCTGAAAAATCTCAAATACATACATGGAAATGAACGCTATAAATCCAAATACAGTCTTGTAGTAGAAGGTAATAAAAATCTTAAACTACTTTGGGATTTTGACAATAAAGAAACCAAACTAAAAATTAACGGGTTGGTTTATTTTGGAAATAATCCAAAACTATGTGTCAATGTAATACAGCAGCTACTTGACAAAGCTAATATACCTGCTTCAGAGGCTTTTTACCCAAATATGACTAACGGTGTAAAAAGTATATGTGATCTTAAAAACCTTACCACATCATATGTAAATGTTACTGCCAATAGAGTCAATATAACGTGGAAAGCAGAATATCACGACCTCAGAAAGGTTATGGGTTATGTTATTTATATTAAAGAAACAATTTTTAAGAATGTTACAGTCTTTGACGGAAGAAATGAATGTGGTAGAGGAGGTTGGACTGTTTACGATGTCGACGGTTCTGAGGCTGAGTACAAAATTATTAGTGTCAAGCCTTATACTAGTTACGCTTTTTATGTCGAAACAGTTACAATCAATGCGGATTTCGGAGCTAAAAGTGAAATAAAATATTTCAACACGCTGCCCGGTATTCCCAATCCACCTTCCTATGTGAATGTCATCACAAATTCAAGTTCAGAGCTTGTGATAACGTGGCCGCCTCCATTGGAACCTAATGGCAAATTGTCACATTATCTGTTATCAATTTATGAGTTGGATGACAAAGCCACGTTGAATAATGCTGTGGAGAATCATTGTGAGGGTAATGATGCATATTCCGTGCCGAGTAACAGCACTCGGTACTCTGGATCCAATGACAGGTTTAACCATTACCTCAATCGACATTTCACTAGTCAACTCTCCGATGAAGAGAAAGCACGTCAAACACAAGTGGCCATGCTTATCGAAGATAAGATGAACAATTACATATACGTCAAAAATCCTAAAAGGCCGAAAAGAGACACTATCTTTGTTAAAGAATCAGAACCTGTTACCTCCAATGATACCCTACAGGAAACTACTATCAAAACTCAAATTGACTTTAAAAACAAGACTCAGTATAAGACTGATCTGACTCCTGTTAGTAAAAATCAAAATCTTGCTATCATACCGAGAGATGATACGATCAAAAAAGTTATATTAGATCGGGAAAAGATTGATAAAAGCATAACATTAAAGTCTATTAAAAATTTGAAGCATTTCACTAAATACCGTGTAGAAGTTCAAGCATGCCGTCAGTTGACAGAAAATGAACAATATTCCTCTCTAAACAATCATTGCAGTGATTCAGCGACAGAACTAATTAAAACATGCCCCTCTTTGGATGCTGATTCTATTCCCGATAAGAGTCTGTTACCCAAAGTCTTGAACGAGAATCCTGGAACTGTTGAGTTGAAGTGGGAAATCCCTACAGATCCTAATGGAATCATTCTCTCCTACACCATTGAATATAGGAAGAGTGGGTACAATCCTATTACTGAGATCGTTAGCCAGAACGAGTTTTTTAAATCCAATAGTGTATATATTTTAAAGAAATTAAGACCTGGGAATTACAGCGTTCGTGTGAGAGCAAGTTCTTCCAGCGGTGTGGGACCTTACACATCCTCCAAATATTTTTATATCAAAGATGAAACTTCTATTCACTCTTATATTTATTTAATATTGGGTACTCTTTTGTTTATTTTACCTCCTGCAATTGCTTACTGTGTTTATTACCTTAGAAAGACAGAGAAGAACAAACTTTTTATATCTGTGAACCCTGAATACTCTCATAAGACTTATGTGGCCGACGCCTGGGAGATACCTAGAAACCACATTGATATTATTAAAGATATTGGTCAGGGCTCATTTGGCATGGTCTATGAAGGCCTGTTAAACAGCAAGCCTTGTGCAGTCAAGACAGTGAACACACAGGCCTCAGACGGTGAAAAGAAAGAGTTTC

>INR2_Aquarius_paludum

ATGAGGTGGATTGTGATGGTGTTGGTGGCCTGGCAGATCGTATGCTTGGCGGCCATCACAGACCACGGAGTTACAAACAAGGACGGCCAAGTTGCCAAAAAAGACGGAGGTTTTATCAAGGGCGACGGAGTTTGTTCTTCCATAGACATTCGCAACGGCGTCGAAAACTTTAAATACCTTAACGGTTGCAGGGTCATCGAGGGCTACCTTCAAATCGTACTTTTAGAACACTCCACCCCCGATTTATTTGAGAACAAAACTTACCCGGAATTGCGCGAAGTGACCGGATACGTGATCTTTTACCGGGTGAACGGCCTTAGATCGATTGGCCAGTTGTTTCCAAATCTGAGTCTGATACGTGGAGAGCGGCTGTTCATTGATTACGCGCTATTAATTACACAGATGCCTGTCCTCAGAGATATCAGCCTAGTAGGATTAACCACCATCCTCCGGGGATCTGTCGCCCTCTTTAATAATCCAGAACTGTGCTTCACGGACACAGTGGACTGGAGCCGAATAGTCAAGGGAGAGCCTTTCATTTATAGAAAGACAAATCTGCCCTGCCCTGCCTGCCCTAAGAGCTGTCCTACCAGTTGCTGGAACAGTACTCACTGTCAGGTGATGGATAAAGTGATTGCTGAAGCAGAATGCGACGAGCAATGTATTGCCGGCTGTACAGGACCTGGTCCACACCAGTGTACTGCTTGTAAGCATCTGTTGCGTCAAGGAGTCTGCACTAACACCTGTCAAGATAATGAGTTCACTTATCAGAAGAGACACTGTGTAACTGAAGAAAAATGCAAAAATATGACTTTTTCTGGAAGTGGGAAACGTTTTATTTTAGAGGAAAGTAGGCCTTGGTTTACTTGGGAAAGGTANNNNNNNNNNNNNNNNNNNNNNNNNNNNNNNNNNNNNNNNNNTGAGAAAGAAGGATGTCGCAAATGTTCTGGTGCATGTCGTAAAGTCTGCCCAGGGCTGGTTGTGGACAGCACACAAAGTGCTCAGACTCTTGAATTCTGCACACACATTACTGGTAACCTTGAGATACAGATTCAAAGCAACGCCTCGGTAAATATAACTGATGAACTTCATAAGTTTCTTGGTTTAATTGAAGAGATTGAAGGCTACCTCAAAGTCATTCGTTCATTTCCATTAGTGNNNNNNNNNNNNNNNNNNNNNNNNNNNNNNNNNNNNNNNNNNNNTCAAACCTCGTCTGTTAAAATAGGATTACTTTCAAAAGATAACTCTACTCACAATTATGCGTTTAGAGTGTTAGATAATCCAAACCTGCAGGAACTGTTTGACTGGAATCGCCCTGCTAAAAAAAACTTTACCATTGAGAATGGTCGCCTTTTCTTTCATCTTAATCCTAAACTATGTCTGAAGTATGTTAAAGAATTTATCCAAATTACTAATATCACAAATATTACTGATCTAGAAGTAGATGAAACATTTAATGAAAAAATGTTTGNNNNNNNNNNNNNNNNNNNNNNNNNNNNNNNNNNNNNNNNNNNNNNNNNNNNNNNNNNNNNNNNNNNNNNNNNNNNNNNNNNNNTGTTAAATATTTCCAGCTCTCAGCAAGATTGGGTGAGAGTTTTAGTTTATTACAAAAAAGCTCCATCTATGAATGTGACGTCGCATTATGATGATAATGTGTGTAAAGATGATGATTGGAAATTTAATGATTTCAAAACTGAGTATGGAGATGATGGAGATGGGACTCCACTGATTCGTCCCCAGTTGATAACCCAGCTAGAACCATACACTCAATATGCTTACTATGTAAGGACTTACAGCTTAGGCTCTCCTATTCATTATGTTCGTACATTACCAACTAAACCATCTTCACCTGAAAACTTACAAGCTTACAGTGACTCAAGTGATAAAATTATTTTTGGCTGGTTACCACCTTTTCACCCTAATGGTATTCTTAAAGAGTACATCATTAAAATTGTTTTGTTGGAAGATGATCAAGATCTCCCAAAATTTAGAAATTTTTGTGCCAATCCTGCTAATTTTCCTGGAAATACAACTCCACCTCCCGACTTTAAAACTATTATTAAAGAAGACAAGGTTAAAGAGTTAACAGTACCTAAAATATTATGTGACCAGTCGGATTTTGACAAATTTGGTAAAATTTCTGACTTGTATTTACAAGACCACCATATTAAATCATGTGAAAGATCAATGTACATAACAATAAACGAAAATGTGCTAAAGCCAAAGTTTTCATCTAGATCAAAAGAGAGACGCTCCACAAAACAGANNNNNNNNNNNNNNNGAGAGAACAAATACGATGAAGACGTACATAGCTTTGTCAAAAATGGCACCACATTCAATTCTGACGGCACTATAAAAACAGAAACTATCTCAGTGAGTAGTAACATGACAAGTTATACTCTTAAAAATCTTATGCATTTTGGAAGATATCTTATAGAAATAAAGGTTTGTAGAGAAAAAGCTCCTGAGGAAGACATGAAATATTTCTTGGATACCCAGTGTAGTGACATGTCTTTTATTACATATCGTACTTTGAAGTCCTACACAGCTGATCTAGTGGATCAGAACAGTATTTTAATAAAAGTGACTAATAAAAGTGCTACAGTAAAATGGAAAAAACCCAGTAACTACAATGGTGTTATTCTAAACTACATTTTAGAATATAAAAAAATTGATGAAGAAAATCCTAACTTACATTTTAAAGCAACTGAGTGTATTTCTGCATTTAATAATAAATCATCAATTACTTCATTTACACTGACCAATTTGAACTATGGCGAATATGCTGTTAGGATTCAAACAATATCCCTTGCAGGAAAGGGCTCCTTTTCCAGGCCTATTAATTTTTATATTGTTGATGATTCATATTCGAATTGGATGGAAAAACTCGCTGTAATAATAGTTGTTACTCTAATATGTATATTTGGGGCTTTGCTCAGTGCTAAAGGAATGGTTTATTATTATCAAAAAAAGTTAAAAGAAGCCAATATATTAATTGAAACAATTAATCCAGAATACACTAGTGTAAACGGTTATATAGAAGATGATTGGGAGCTGTCTAGAGATGACATTGTTTTCGTTAATGAAATAAAACAAGGCAATTTTGGAATGGTTTATGAAGGCTTGCTGTTGCCTGAGAATAGAAAAGTGGCTATAAAAACTGTCAAAGAAACTGCGTCCCCTAGAGATAATTATGATTTTCTAAATGAAGCTAATGTGATGAAAGCTTTTAGTGGTGCAAATCACGTGGTCAAACTGCTAGGTGTAGTTTCAAAAGGAACTCCTGCTCTTGTGGTTATGGAGCTCATGGTTTTGGGTGACTTAAAATCTTTTCTCAGACTAAGCAGAGATTCTCCTCAGTATCCTCCTCCATCACCCTCGAGGCTTAACCTAATGGCTGCACAGATAGCAGATGGTATGGTCTATTTAGAAGCTTTGAAATTTGTCCATCGAGATCTTGCGGCTAGAAACTGTATGGTTTCAGAGGATCTAACTGTGAAAATCGGTGACTTTGGAATGACACGAGATATCTATGATACTGATTATTACAGAAAAGGAAACAAAGGGCTTTTACCAATCAGATGGATGGCTCCTGAAAGTCTCAATGATGGAGTATTTACCAGTTTTTCAGACGTCTGGAGTTTTGGAGTAGTCCTCTGGGAAATGGTCACATTAGCAACTCAACCGTACCAAGGAATGTCTAACGAGGAAGTACTGCAGCATGTCATATCAGGGAACAAGCTCGACACTCCTGTTTACTGTCCACCCCTCCTTAAAATGATCATGCTCTCTTGTTGGAAATGGAAGCCTAAACTTCGTCCTACATTTGTTCATATCCTCAATCTACTAGACCCAACTCTAAACAATGAATTCCGCTCAAATTCATATTATCATAGAGTTGCCAGAGAAAGAACTCATCTTATAGAAGCAGACGATTATAGGGATAATGAACACACGTCTTTACTTCTTCTTCCCTCCTCTTCTTCTGTTGAATTCCACCCTTCTAATAACAATTAG

>INR1_Aquarius_paludum

ATGGCAGCTAATTCCGGACGTCTCACTATATTCAATTTGGAGATTTACGAAGGTTCTAGTTTAATTAAAACGAGTGGAGTTTTATTTTTTGGAATTATTTTATTCAGCTCTCTTGTAACTGCTTCCCATAAAGCAGATATTCCTTCGGCTTTACGTATTTATTCTGAAGTGGTCCATAATGATGATGATACCTCAGTGTGTCAAAGTAAAGACATTAGAAACACCGTGGACATGTTTGTTGAGCTTCAAGGATGCGAAGTCATTGAAGGATTTGTACAGATAGTGTTAATTGATAAGGCTGAACCAGCCGATTATGTAAATGTTTCATTTCCGGAACTTCGTGAGATAACCGGTTATTTAGTTTTATATAGAGTCCAAGGATTGAAATCTCTTAGCAAATTATTTCCTAATTTAAGCGTTATCCGAGGAAACACACTATTTTTGGACTACGCCTTGGTGGTGTACGAAATGTTACAGCTCCAGGATATTGGTCTAATATCTTTGACAAATATTTTAAGAGGTGGAGTTTATTTTGCCAAAAACCCCATGTTATGTTTTGCCGAAAGCATTGACTGGGATATAATCGCCCCACATGGCAAAGGAGATCATCATATTATGGGAAATAAAAATAACTTTGAGTGCCCACTATGTCCGCAAATAGTAAATGCCAACGAATCTTGCCCAAAACATGCATCTAGTGGAGAACCACTTTGTTGGAATATGAACCACTGTCAGAAAAGTTGCACTCTACAGTGTTCAGGAGGGTGTAACAGCGATCTAGATTGTTGTCATCCTCTATGTTTAGGAGGATGTCTTGACAAAAGTCCTCACTCTTGTTTTGCCTGTAAACATTTCCTGCACAACAATGAATGTGTCGAGCGATGCCCTGAAGGGACATATGAATATTTGAATCGCAGATGTGTAACTGATAAAGAGTGTTTAAACATGCCAAAGCCTCGAGAGAACACTGATCACAACACTAAAAATAAACCGTGGAAACCTTTTAAAAAAGAATGTGTTCTTGAATGTCCACCTGGGCATGTTGAGAAACAAATTTTTGTTAATGGAGAATTGAGGATGGATTGTGAGAAATGCAAAGGTATCTGTAAAAAAGAATGCGATGGGATGAATGTCGACAGTATAGCTGGGATTCAGAAATTGCGTGGATGTACAACAATTAAAGGGGCTCTTGAAATTCAAGTTAGAGGAAGTAACAATATGGTAAGAGAATTGGAAGAAAATTTCAACACAATTGAAGAGATCAATGATTATTTGAAGATTGTAAGATCCTTTCCTCTTATCTCTTTGAATTTCCTCCCTAAACTAAGGGAAATTCATGGTAGAAAACTTGAAAGTTCAAAGTATGCTTTAGTTGTTTTGGATAACCAAAATCTTATTGAATTGTGGGATTGGAATAATCGTTCAACTAGCTTGACCATAGGCAATGGTCGACTATTTTTTCATTTTAATCCAAAATTATGCTACGATAAAATTGAGGAATTAAGAAAGAAGACTAATTTATCAGCTCACTCAGACTTGGAAATTGCCAGAAATTCCAATGGGGATAAAACAGCTTGTAATGTGGAAAAACTGAATGTAACCGTGTATAAGAAAACAGCAGTGGCAGCTTTAATTAAGTGGAGACAGTTTGAAAATAATGATCCGAGAACTCTTTTGGGTTATGTTGTTTATTCTATTGAAGCTCCTGCCAAAAATGTTTCTATTTACGATAGACGAGATGCGTGTGATGATAAATGGAGAGTAGACGATGTTTCTGTTCAAGAAGATGCTGATGCATCTGAAGAGAAGGAGGTGAATCATATTATAGCTCAACTGAAACCCTACACTCAATACGCGTTCTACGTTAAAACCTACACCATTGCAACAGAGACAAGGGGTGCTCAGAGTCCGATAAAGTACTTCCGAACAGAACCAGACATTCCTTCTGAACCACATGGAATTTCAATACAATCTAATTCGAGCTCTGAATTAGTTCTTCGATGGAAACCTCCTACTAATCCTAATGGGAATGTGACTCACTTTATAGTTACTGCAGTGCTGGAGCCTGATAATGAAGAGGACGTGGAACAGCAAAACTTTTGTTTAGAACCTTTGTTGTTCCCAGACATTAGAAGGATACCAAGTAAAAACCCCTTTCAGGATATGGTCGGACCAAAAGATGAGAAGAATGAGTCTATTGATAATGAATCGTGCTCTTGTAACCAAAAATTTATTGACAATCAACTTAGGATGAAAGAAATTGCATTTGAAGATTATCTACAAGATAATATCTTCATTAAAAGAGAGACATATTATGAAGAGTCTCCTAGTGGAAGAGAAAAACGAGAAATATCAAAATCACCTTCTTCAGTGTTCGATGAGAGCGATAATACTATTCTTGATAATATGTTTGAAGATGAAAATTCTGACAAAGATAACTACAGTGTAAATGGTGTTTTCAAACGTTTCCGTCAAATTGTGTTCGGAGAAAATTATTCAATTGTTATAAGAAATTTAAAGCATTTTTCTAAGTATAGAATATCCATTCAAGCATGTCGAGAAGTCGAAGAAAGTGAAGATAAATCAAGGGTTCAAAACTGCAGTAAAGAAGGCGTTATTGCTGGAAGAACTTTACCCATGAGTGATGCTGACAACATAGATGGCGATCAGGTATTTTGGGAAGTTTCAAATACCACCCATGGAATAGTAAAATTGAAATGGGAAGAACCAGTTAATCCAAATAGTTTGATATTAACTTACCAAATTGAATACTGGCGTACCGATATTGAAAATTACAAACCAACAGTTGAATGTATTTCAAGAAGCCAATATAAAGCTCTAAACAAAGGATATGTTTTAAAAAATCTCAGCCCTGGTAACTATAGTCTAAGAATACGAGCTTCATCATTGTCCGGCAATGGAATGTACACTAAAATTAATTACTTTTTTATTGAGGAAGATTCAGTATCTCCAGTATTACAGACTACCATTATTGTCATTATAATTATGTTATTTATATTTATTATAATTATACCATTTATTGTATTTTATATTAGAAGACATATTGAATATGGCCGCAGTATAAAGCTTATTGCTTCAGTAAATCCTGAGTACGTTCCTTCTGGCAAAGTTTATACTCCTGACGATTGGGAAGTGCCTCGAGAGAATGTGGAACTTATAAAAGAAATAGGTCAAGGCTCATTTGGAATGGTTTATGAAGGCACACTTAAAGACACTGGTATTCAAAACTCAAGACCTTGTGCTATTAAAACTGTGAGCTGCAACAGCAAGACTCAAGAACGGGATAGAATTGAATTCCTTAATGAAGCTTCTGTTATGAAGGCTTTTAATACTCATCATGTAGTGAAATTGTTAGGTGTGGTTTCTCAAGGCCAACCTACTTTAGTAATAATGGAGCTTATGGCTCATGGAGATTTGAAGGCCTATTTAAGGTCTTGTCGACCAGATGGAGGATACTTCCCTCAGACACCACCAACCCTTAAGGATACTCTTAGAATGGCTGTTGAAATAGCAGATGGAATGGCTTACCTTGCTGCAAAAAAATTTGTACATCGTGACTTAGCTGCGAGAAATTGTATGGTTACAGACAACCTTACAGTTAAAATAGGCGATTTTGGAATGACACGTGACATTTATGAAACTGAGTATTATAGAAAAGGATCCAAAGGTCTTTTACCTGTTCGTTGGATGGCACCTGAATGCCTTAAGGATGGTGTATTTTCCAGTCTCTCTGATGTATGGAGCTATGGTGTTGTACTGTATGAAATGGCTACATTAGCTTCACAACCTTATCAAGGCCTTTCTAATGAGCAAGTATTAAAATTTGTCATTGATGGAGGTGTTATGGATCGACCTGATAACTGTCCTGACACCTTGTATCACACAATGTTGATGTGCTGGCAACAAAAACCTAGGAGCCGACCCTCATTTATGAACCTCGTGGGCATGTTAAAGGAGCATTCAAATCCTGACTTTGCTACTGTTTCATTTTATGACAGTGAAGAAGGACAAGAAATCAGGGGAATCGAAGTGACTGAAGAAACTCCTTTGTTTGTGTCAAGGGTTATTGAAGACTTTTCTCTTAGCGATGACGAATCAAGCAATCCTATAAAGAATTGTAGCAACACTGGTGATGAGGATGAGATTGACGATGCAGATGATTATGGAGATATTAGTTTAAGAAACACGAGTGATAGTTGGATCCGTAGTGAAGGTCCTCCAGGTGGTGTAGAAAGCTTTGGAGTCCCCCATATGATTTCTCGACATGGTAGCTTGTTGAAAAGTAACTCAACCGAGAAAGAATGCAAAGGGAGAGGAGATATTGAAGATTTTGAAATAGCTCCGTCGAGTGTGAATTCGAACAGAAACAATGGTCGAATGACTCCTAATGGTTGTATTTCGTATAATGATGTAAAAACAACCACGTGTTAA

>INR1-like_Limnoporus_dissortis

ATGGCCTGTTATTGCTTTATCGTCATGTCTAAATATATGTCTATCGGTCTGTTTATATTGTTTTGTCAAATGTGTCGTTCCGTTTCGGTCGGCCCTCCCATCGGTTCGAGTACGGAATCCTCTAAAAGTAACGACGGTTTAAGTATTTGTGGGGACGTTGAAATATGGAGTCCCCAAGACAAAGATGCGATCCGCTTGAAGAAGTGCTCGGTGGTAGAAGGATACGTCAGACTGATGGAGGTCGACGCCGGGGAAGATTTTAGCTTTCCCGATCTCAGAGAGATTACAGGGTATCTGTTGGTTTTTCGAGTAAAGGGTTTCGATTCGCTTGGTCAGGTGTTTCCCAAACTCGGTGTCATCCGTGGAAATGTGCTGTTCGGGAATTTCGCTTTGATAATTACCGAGTCTTTAGACTTGAAAACAATCGGTCTTACTTCTCTAAGTCGTATTATCAATGGAAAAGTGTGGATCGCATCTAATGTCAACCTCTGTTTTGTCAACACTGTAGATTGGAACTCAATTGGCCGTGTCAGCAGAAAGGATTATTTAGAAGGTAACAAAAGCCCGAACGAATGTCCGGTGTGTAGGAACTGTACGAAATCTCCCACTGGGAAGATGAGTTGTTGGAATTCAACAAGCTGCCAGGCAACAAGGTGTCCGTCTTCTTGTCCTGATAACTGTTACAACGAGACTACTTGCTGCCACCAACATTGTTTGGGTGGATGTTCGGGAGATAAAGAAAACGAGTGCTACAGCTGTAAGCACTTCAGGTTTGGAGAAAAATGTGTTAAGAGCTGTCCTGTAGGTACTTACGTATATGCAGGGTGGAGATGCACTAATGAATCGGAATGTTACGGTTTCCCCAAGACTCCTTCAAACAACCACTGGAAACCTCACAACGGACAATGTGTATTAGACTGTCCATCTGGTTACCACGAGCAAGAAGTCTACATTGGCAGTGTAAAAAGATTCCAGTGTCAAAAATGCGAGGGTGGCTTCTGTCGTAAGGAATGTAATGGTGCTTCCATAAAAAACAAAAATGATTCTGTACTCATGTCTGGGTGCACTGTTGTACGAGGCAGTGTCGTAATCAGCCTGTCTGGAAGTGGCAAGGACATTATCTCTGAACTCGAGAACAATTTAAACACAATTGAAGAAATAACCGGTCTTCTCAAGATAACAGACTCCTACCAACTGACTTCTCTCAACTTTCTGAAAAATCTGAAATACATTCATGGAAATGAGCGCTATAAATCCAAATACAGCCTCGTAGTAGAAGGTAATAAAAACCTTAAACTGCTTTGGGATTTTGACAACAAAGGAACCAAACTGAGTATAAATGGGTTGGTTTATTTTGGAAATAACCCAAAACTGTGTGTCAATGTAATTCAGCAGCTACTTGACAAAGCTAATATACCAGCTTCTGATGCATTTTACCCTAATATGACTAATGGTGTAAAAAGTGTATGTGACCTTAAAAATCTTACCACTTCATATGTTAATGTAACTTCTAATAGAGTCAATATAACGTGGAAAGCCGAATATCATGATCTGAGAAAGGTAATGGGTTATGTTATTTATATTAAAGAAACAGTTTTTAAGAATGTTACAGTTTTTGATGGGAAAAATGAGTGTGGTAGAGGAGGGTGGACTGTGTATGATGTGGACGGCTCTGAGGCCGAGTATAAAGTTATTAGCGTCAAGCCTTACACTAGTTATGCTTTTTATGTCGAAACTGTTACAATAAATGCTGATTTCGGAGCTAAAAGTGAAATTAAATATTTTAACACGTTGCCTGGTATCCCTAATCCACCATCATATGTGAATGTCATTACTAATTCAAGTTCGGAGCTTGTGATAACCTGGCCACCTCCGTTGGAGCCTAACGGTAATTTGTCTCACTATTTGTTATCAATTTACGAATTGGACGATAAATCCACATTGAATAATGCTGTAGAGAATCACTGTGAAGGCAAAGATGCGTATTCCGTGCCAAGCAACAGCACCAGGTACTCTGGAACCAATGACAGGTTTAACCATTACCTCAATAGACATTTCACCAGTCAACTCTCCGATGAAGAGAAAGCCCGTCAAACTCAAGTGGCCATGCTTATTGAAGACAAGATGAACAACTACATCTATGTCAAAAATCCAAAAAGGCCAAAAAGAGACACTGTCTTTGTTAAGGAATCAGAACCTGTTGTCTCCAATCATACCCTACAAGAAACTACTAAATCTCAAATTGACCTTAATATCATAACTCAGAATAAGACCGACCTTACTCCTCTTAGTGAAAAGCAAAATCTCCCTATCATTCCAAGAGATGATATGATTAAAAAAGTTATATTAGATCACGAAAAGATTGATAGAAGCATGACTTTAAAGACTATTAAAAATCTGAAGCATTTCACTAAATACCGTGTAGAAGTTCAAGCCTGCCGCCAGCTTACACAATATGAACAGGATTCCTCCCTGGACAATCACTGCAGTGATTCGGCGAGAGAACTAATCAAAACATGCCCCTCTATGTCTGCTGATTCTATTCCAAATGAGAGTCTGCTACCCAAAGTCTTGAATGAGAATCCAGGGAATGTTGAGTTGAAGTGGGAAATCCCTAAAGATCCCAATGGAATTATTCTTTCTTACACCATTGAATACAGGAGAAGTGGATACAATCCCATTACTGAGATCGTTAGCCAGAACGAATTTTTGAGATCCAATAGTGTGTATATTTTAAAGAAATTAAGACCAGGAAATTACAGTGTTCGTGTGAGAGCAAGTTCTTCAAGTGGTGTGGGACCTTATACATCATCCAAATATTTTTACATCAAAGATGACACTTCTATTCACTCTTATATTTACTTAATATTAGGCACTTTATTGTTTATTTTACCACCTGCAATTGCTTACTGTGTTTATTACCTCAGAAAGTCCGAGAAGAACAAACTTTTTATATCTGTAAACCCCGAGTACACCCATAAGACTTACGTAGCCGACGCCTGGGAGATCCCTAGAAACCACATTGATATTATCAAAGAAATAGGTCAGGGCTCATTTGGCATGGTCTACGAGGGTCTGTTAAATAGCAAACCTTGTGCGGTCAAGACTGTGAACACACAGGCTTCAGACTGTGAAAAGAAAGAATTCCTGAACGAAGCTTCTGTGATGAAAGCTTTTGATACATACCATGTTGTAAAGTTGTTGGGAGTCGTGTCACTTGGGGAACCAGTTTACGTTGTTATGGAACTGATGCCTAATGGTGATTTGAAATCATTCCTACGCTCTCACAGGCCAGATGTATGTGACCCTCATCTAAGACCCCCATCGGTCAGAAGACTTCTTCAAATGTCTGCAGAAATAGCAGACGGTATGGCTTACCTCTCTGCCAAAAAGTATGTCCACCGTGATCTTGCTGCCAGGAATTGTATGGTTGCAGAAAACCTAACTGTTAAAATAGGCGATTTTGGTATGGCCCGAGACATCTACATCGGGGACTATTACAGAAAAGGCACACGAGGGTTAATGCCCGTGAGATGGATGGCTCCGGAGAGTCTTAGGGACGGTATATTCCTGTCAGCTTCGGATGTGTGGAGCTATGGTGTGGTGCTTTATGAAATGGTAACACTAGCTGCTCAACCTTACCAGGGTTTGACTAACGACCAGGTGTTACGCTACATCATGGATGGGGGTGTCATGGAACGACCTGAGAACTGTTCCGACAAGATGTTTGATCTCATGAGAAGGTGCTGGAGACAAAACCCCAGCAACCGACCGGGTTTCCTGGAAATCGTTGAGACACTGCACCAAGACGTCAGTCTAAACTTTCATGCTGTCTCGTTTTATGACAGTGATCAAGGCAAGCAGCACCGCAGGCTAATCGAGCTCAATATTCAGTGTTCAAACAAACCTTTCTAA

>INR2_Limnoporus_dissortis

ATGACGTGGGTTGTGATGGTGTTGGTGGCCTGGCAGATCGCTTGCTTGGCGGCCATCACAGACGACGGAGTTACAAACAAGGACGGCAAAGTTGCCAAAAAAACCGATGGAGTTTTTACCAAGGGCGACGGAGTTTGTTCTTCCATAGACATTCGCAACGGCGTCGAAAACTTTAAATACCTTAACGGTTGCCGGGTCATCGAGGGCTACCTTCAAATTGTACTTTTAGAACATTCCACGCCCGATTTGTTCGAGAACAAAACCTACCCGGAATTACGCGAAGTGACCGGATATGTGATCTTTTACCGAGTGAACGGCCTTAGATCGATTGGCCAGTTATTTCCAAACCTAAGTTTAATCCGCGGAGAGCGGCTATTCATAGACTACGCCCTATTAATTACACAGATGCCTGTCCTCAGAGAAATCGGCCTGGTAGGATTAACCACCGTCCTTCGGGGATCTGTCGCCCTCTTTAATAATCCAGAACTGTGTTTCACGGACACGGTGGACTGGAGCCGAATAGTCAAGGGAGAGCCTTTCATCTATAGAAAGACAAATCTGCCGTGCCCTGCTTGCCCTAAGAACTGCCCTAACAGTTGTTGGAATAGTACTCACTGCCAGGTAACAGATAAGTCAATTGCTGAAGCTGAATGCGATGAGCAGTGTATTGCAGGTTGCACAGGACCTGGTCCCCATCAGTGCACTGCTTGCAAGCACCTATTGCGCCAAGGCGTTTGCACCAACACCTGTGAAGATAATGAGTTTACTTATCAGAAGAGACACTGTGTAACTGAAGAAAAATGCAAAAATATGACTTTTTCTGGAAGTGGAAAACGCTTTATTTTGGAGGAAAGTAGGCCTTGGTTTACTTGGGATAGGACTTGCATACAGAACTGTCCAAAAGATTTTGAGAGGGATGAGAAAAAAGGATGTCGTAAATGTTCTGGAGCGTGTCGCAAAGTATGCCCAGGACTGGTTGTGGACAGCACACAGAGTGCACAGACACTTGAATTCTGCACACACATTACTGGTAACCTTGAGATACAGATTCAAAGCAATGCCTCAGTGAATATAACAGACGAACTTTATAAATTTCTTGGTTTGATCGAAGAGATTGAAGGCTACCTTAAAATCATTCGTTCATTTCCGTTAGTCAATTTATACTTTTTTAGAAATCTTAGGGTTATCAAAGGTCTTCAAACTACATCCACTAAGAAAGGATTACTCCCAAAAGATAATCCCCCCCACAATTATGCATTTAGAGTGTTAGATAATCCAAACCTTCAGGAACTGTTTGATTGGAATCGTCCAGCTAGAAAAAACTTTACCATTGAGAATGGTCGTCTTTTCTTTCATCTTAATCCTAAATTATGTCTGAAATATGTTGAAGAGTTTATCCAAATTACTAATATCACAAACATTACTGATTTAGAAGTAGATAAAACATTTAATGAAAAAATGTTTGCATGCAATGTTAAAAATATATCTGTGTATGTAAGTGTTAAGACTTCTAATAGTTTTGTTGTTGGTATAAAATCAGACGTTTTAAATATTTCCAGCTCAGAACAAGATTGGGTGAGAGTTTTAGTTTACTACAAAAAAGCACCATCTATGAATGTGACATCACATTATGATGATAATGTGTGTAAAGACGATGATTGGAAATTTAATGATTTTAAAACTGAGTATGGAGATGATGGAGATGGGACTCCTCTAATTCGTCCCCAGTTGATAACCCAACTAGAACCATACACTCAATATGCTTACTATGTAAGGACTTACAGCTTAGGCTCACCGATTCATTATGTTCGTACATTGCCAACTAAACCATCTTCACCTGAAAACTTACAAGCTTACAGTGACTCAAGTGATAAAATTATTTTTGGCTGGTTACCACCTTCCAACCCAAATGGTATTCTTAAAGAGTACATAGTTAAGATAGTTTTGCTAGAAGATGATCAAGATCTACCGAAATTCAGAAATTTTTGTGCCAATCCTGCTAATTTTCCTGGAAATACAACTCCACCTCCTGACTTTCAAACTATTATCAAAGAAGACAAGAGTAAAGAGATGACAATACCTAAATTGTTATGTGACCAATCCGATTTTGACAAATTGGGTAAAATTTCGGAATTGTTTTTACAAGACCATCATCACATTAAATCATGTGAAAGATCTATGTATACAATAATAAATGAAAATGTACTTAAACCAAAGTTTTCATCTAGATCAAAAGAACGACGTTCCACAAAAATTTCAAATCAAAATACAATTGATACNNNNNNNNNNNAATATTTTTTAGATTCTCAGTGTAGTGACATGTCATTTATAACATATCGTACTTTGAAGTCCTATACAGCTGACCTAGTGGATCAGAATAGTATTGTAATAAAAGTAACAAATAAAAGTGCTATAGTAAAATGGAAAAAACCCAGTAACTACAACGGTGTTATTCTAAACTATATTTTAGAATATAAAAAAATTGATGAAGATAATCCTAACTTACATTTCAAAACAACCGAGTGCATTTCTGCTTTTAGTAATAAATCATCAATTACTTCATTTACGCTTACAAATTTGAACTATGGCAAGTATGCTGTTAGAATTCAAACAATATCCCTTGCAGGAAAGGGCCCTTTTTCTGGTCCTATTGATTTTTATATTGTTGATGATTCATATTCAAATTGGATGGAAAAACTTGCTGTAATCATAGTTGTTACGCTAATATGTATATTCGGAGCTTTGCTCAGTGCTAAAGGAATGGTGTATTATTATCAAAAGANNNNNNNNNNNGTTAATGAAATAAAACAAGGGAATTTCGGAATGGTGTATGAGGGCTTACTGTTGCCGGAGAATAGAAAAGTGGCTATAAAAACTGTCAAAGAAACTGCATCTCCTAGAGATAATTATGATTTTCTCAATGAAGCTAATGTGATGAAAGCTTTTAGTGGTGCACATCATGTGGTCAAATTGTTAGGTGTTGTTTCAAAAGGAACTCCTGCNNNNNNNNNNNNTTCAAGGCTTAACCTTATGGCTGCACAGATAGCAGACGGTATGGTCTATTTAGAAGCTCTTAAATTTGTCCATCGAGATCTTGCTGCTAGAAACTGTATGGTTTCAGAAGATTTAACTGTAAAAATCGGTGACTTTGGAATGACTCGAGATATCTACGATACTGATTATTACAGAAAAGGAAACAAAGGACTTTTACCAATCAGGTGGATGGCTCCTGAAAGTCTCAATGATGGTGTATTTACCAGTTTTTCAGATGTCTGGAGTTTTGGAGTAGTCCTTTGGGAAATGGTAACATTAGCAACTCAGCCATACCAAGGTATGTCAAACGAGGAAGTGTTGCAGCATGTCATATCAGGGAACAAGCTTGACACACCTGTTTACTGTCCACCTCTCCTTAAAATGATCATGATATCTTGTTGGAAA

>INR1_Limnoporus_dissortis

ATGACGAAACAGGAGCTTAGGCAACATGATATAAAAACTACCAAAGTCAAATGGCGAAGCGTTATACTTAGTGATTGGATCAAAGGCTCAACTTTAAATAAAACGAGTGGACTTTTGTTTTTTGGAATAATTTTCTTAAGCTCTGTTGTAACTGCTACCCATATACCAGATATTCCTTCGGCTCTTCGGATTTATTCGCAAGTGGATTATTATGATGATGATGCATCAGTGTGTCAAAGCAAGGATATTAGAAACACTGTGGACATGTTTGCTGAGCTTGAAGGTTGCAAGGTCATTGAAGGATTTGTTCAGATTGTGTTAATTGATAAAGCTGAACCAGCTGATTATGCTAATATTACATTTCCTGAACTGCGTGAAATTACTGGGTATTTGCTTTTGTACAGAGTCCAAGGTTTAAAATCTCTTAGCAAATTGTTCCCAAATTTAAGCGTTATTCGAGGAAACACACTATTTTTGGACTATGCCTTGGTCGTTTACGAAATGCTCCAACTGCAGGATATTGGTCTACAATCTTTGACTAACATTCTAAGAGGTGGAGTTTACTTTGCCAAAAACCCTATGCTGTGCTTTGCTGAAAGTATTGACTGGGATTTGATTGCCCCTCATGGCAAAGGAGACCATCATATCATGGGGAATAAAAATAACTTTGAGTGCCCACTATGTCCTCAAATGGTGAATGCTAACGAGTCTTGCCCAAAACATGCATCTAGCGGAGAACCACTTTGCTGGAATATGAATCACTGCCAGAAAAGTTGTACTCTACAGTGCTCAGGAGGGTGTAACGCTGATCGAGACTGTTGTCATCCTTTATGTTTAGGAGGATGTCTGGACCAAACTCCCCACTCTTGTTTTGCTTGCAAACATTTCCTGTTTAACAATGAATGTGTTGAACGATGCCCTGAAGGGACTTACGAATATCTGAATCGTAGATGTGTTACTGATAAAGAATGTTTAAACATGCCAAAGCCTCGAGAAATAACAGATCATAACACTAGAAATAAACCATGGAAACCATTTAATAAAGAATGTGTTATTGAATGTCCGCCTGGGCATGTTGAGAAACAAATATTTGTTAATGGTGAGATAAGGATGGACTGCGAGAAATGCAAAGGTATCTGCAAAAAAGAATGTGATGGGATGAACGTTAATAGTATAGCTGGAATTCAGAAGTTGAGAGGATGTACAACAATTAAAGGTGCCCTTGAAATTCAGATTAGAGGAAGTAACAATCTGGTAAGGGAATTGGAAGACAATTTCAACACGATTGAAGAGATTAATGATTATTTGAAGATTGTTAGATCATTTCCTCTTATTTCTTTGAATTTTCTCCCTAACCTGAAGGTGATTCATGGTAGAAAACTTGAAAGTTCAAAGTATGCTTTAGTTGTTTTGGATAACCAAAATCTTATGGAATTGTGGGATTTTAAAAATCGTTCATCCAGCTTGACCATAGGCAATGGTCGATTATTTTTTCATTTTAATCCAAAATTATGCTATGGCAAAATTGAGAGGCTAAGAAAGATAACTAATTTGTCTGCTCACTCAGATTTGGAAATCGCCAGAAACTCTAACGGCGATAAAACATCATGTAATGTGTATAGACTGAATGTGACTGTGTACAAGAAAACATCTGTGGCAGCATTAATTAAGTGGAGACAATTTGAAAATAATGATCCTAGATCTCTCTTAGGTTACGTAGTTTATTCTATTGAAGCGCCTTCCAGAAATATCTCCATTTATGATAGACGAGATGCCTGTGATGATAAGTGGAGAGTGGACGATGTTTCAGTTCAAGATGATAGTGACACATCTGATGAAAACGAGGTTAATCATATTATTGCTCAACTGAAACCCTACACCCAATATGCATTTTATGTTAAAACCTATACCATTGCAACTGAAACAAGAGGTGCTCAGAGCCCCATAACGTACTTTCGTACAGAACCAGACATTCCTTCTGAACCATATGGAATTTCAATACAATCAAATTCAAGCTCTGAATTAGTTCTTCACTGGAAACCTCCTACTAATCCTAATGGAAATGTAACTCACTTTATAGTTACGGCAGTGCTGGAGCCTGATGAAGAGGACGTGCAACACCAAAATTTTTGTTTAGAACCTTTGTTGTTCCCTGAAATTAGAAGGATGCCAAGTAAAACCCCTTTTCAAGAAGTTGTTGGACCAAAAGATGAAAGGAATGGATCTACTGATAACGAATCATGCTCTTGTAACCAAAAGTTAATTGACAATCAACTTAGAATGAAGGAAATTGCTTTTGAAGATTATCTACAAGATAATATCTTTATAAAAAGAGAGTCATATTATGACGATTATTCTAGTGGAATAGAAAAGCGAGAAATACCAAAATCATCCTCTATGGTGTTTGCAGAGTCTAATACTGATATTCCTGATTATAAATTTGAAAATGAAAGTTCTAATAAAGATAACTGCAGTGTAAATGGTGTTTTCAAGCGTTTCCGTCAAATTGTGTTTGGCGAGAATTATTCGATTGTTATAAGAAATTTAAAGCATTTTTCTAAGTATAGAATTTCCATTCAAGCATGCCGAGAAATTGAAGATTATGAAGATGTTACCAAGGTTCCAAACTGCAGTAAAGAAGGAGTTATTGCTGGCAGAACTTTACCCATGAGAAATGCTGACAACATAGATGGTGATCAAGTTTATTGGGTAGTTTTAAATACCAGCCATGGGATAGTAAAATTGAAATGGGAAGAACCAGCTAATCCAAATAGTATGATATTAACATACCAAATTGAATATTGGCGCACCGATATTGAAAACTACAAACCTACTGTTGAATGCATTTCAAGAAATCAATATATAGCTTTAAATAAAGGATATATTTTAAAAAATCTCAGCCCAGGTAACTACAGTCTCAGAATACGAGCTTCGTCACTGTTTGGGAATGGAAAGTACACTAAAATTAATTTCTTCTTTATTGAGGATGATTCTGTATCTTCAGTATTAAGGACTACAATTATTGTCTTTATCATTTTGATAGTTATACTTATTGTAATTTTACCGTTTGCTGTATTTTATATTAGAAGACATTTGCAATATGGCCGCACTATAACGCTTATTGCTTCAGTAAATCCTGAATACGTTCCATCAGGTAAAGTTTATACTCCTGATGATTGGGAAGTTCCTCGAGAGAATGTGGAGCTAATAAAAGAAATTGGTCAAGGTTCTTTTGGAATGGTTTATGAAGGCACATTTAAGGAAACGGGCATGCCAACCTCAAAACGGTGTGCTATTAAAACAGTGAGCTGCAATAGCAAAACTCAAGAACGTGATAGACTAGAATTTCTAAACGAAGCTTCCGTCATGAAGGCTTTTAATACCCATCATGTAGTAAAATTGCTTGGTGTGGTTTCTCAAGGCCAACCTACGTTGGTGATAATGGAACTTATGGCTCTTGGAGATTTAAAGGCTTATTTAAGGTCTTGTCGACCAATATACTTCCCTCAGGCACCACATACATTAAAGGATACTCTTAGAATGGCTGTTGAAATAGCAGATGGAATGGCTTACCTCGCTGCAAAGAAATTTGTGCATCGTGACTTAGCTGCAAGAAATTGTATGGTTACAGACAACCTTACTGTTAAAATTGGTGATTTTGGAATGACACGAGACATTTACGAAACTGAATATTATAGAAAAGATGTAGGAACCAAAGGTCTACTGCCTGTTCGTTGGATGGCGCCTGAATGCCTCAAGGATGGTGTATTTTCCAGTCTCTCTGATGTATGGAGCTATGGTGTTGTTCTGTATGAAATGGCCACATTAGGATCTCAACCATATCAGGGCCTTTCTAATGAGCAAGTGTTGACATTCGTCATTGATGGAGGTGTTATGGATCGACCTGATAACTGCCCCGACACTTTGTATACCACAATGTTAATGTGCTGGCAACAAGAACCTAGAGGCCGACCTCAATTTATGAGTCTTGTGAGCATGTTAAAGGAGCATGCAAATCCTGACTTCGGTACGGTTTCATTTTATGAAAGCGAAGAAGGACAAGAAATCAGGGGAGTTGATGTGACAGAAGAAACTCCCTTGTTTGAGACAATAGTTGTTGACTTTTCTTTTAGCGATGATGAATCAAGCCATACCATGAAGATTTCAAGTAATACTGGTGACGAAGATGAAATTGACGATGCGGATGACTATAGAGATATCACTTTAAGAAACACGAGTGATAGTTGGATTCGCAGTGAAGTTCCATCAGGCGTTGAAGACCCCCATATGAGTTCACATCAAGGTAGCTTGTTACCTAGCAATTCAACTGGAGAGGAACCACAACAAGGAAGAGGAGACATTGAGGATTTTGAAATAGCTCCGTCGACTTCGGGTTCCAGCAGAAACAATGGTCGAATATCACCTGATGGTTGTGTTACGTATACTATAGTAAGAACAACCCCGTTTTAA

>INR1-like_Rhagovelia_antilleana

CAGTCTATTTGCCCTTTAGCTTGCCCCAAAAGCTGCAGTGAAGAGGGTGAATGCTGTCATCCCTTATGCTTAGGGGGTTGTTGGGGTACAGGTCCGGATAAGTGTTTCAGCTGTAGAAACTATGTGTTTCACGAAACGTGTATTCAAAGTTGTCCTAAAGATACCTTTATTTATTCAGGTTGGAGGTGTATACTTGAGTCTGAATGTTATGCTTTGCCTAAATCTCCTTCAAACAAATTCTGGAAGCCACACAACGGTCAGTGTTTATTGGATTGCCCACCTGGATACCATGAACAGGAAATTTTTATTAACGGCCAAAAAAGGTTTCAATGTCGTCGATGCGAAGGAGGTATGTGTAGGAAGGAATGCCGGGGCGCTTTGATTTCTAGTGTGGAGGATTCTAGGAAGATGCAAGGTTGCACTGTAATCCGTGGGGGCATTATGATCAGTCTCTCGGGTCATGGCAAGAATATTGTCCCACAGTTAGAGAGCAGCCTTAGTGGTATCGAAGAAATCTCTGACTATTTAAAGATTTCTGATTCTTATCAACTCACCTCATTAAATTTTCTCAAAAATCTTAAATATATTCATGGTTATGAGAGGTATAAATCCAAGTACAGTTTGATTGTTATCGGTAATAAGAACTTGGAGTTACTTTGGGATTTCAAGAATAAAGGTAGTGGTCTGAAAATAAATGGATCTGTTTATTTTAGGAGTAATCCAAAACTTTGTTTGAATAAAATACAAGAACTGTTGGACGAAGCGAAAGTCTCGGTCTCTGACTCCTTCTATCCTAGTTTGACTAATGGAGTAAAGAATGTTTGTGACTTAACTGAGCTTATTGTAAATGTTAAAAGTGTAACATCTACATCTGCGACTGTATTATGGCGACCTTTTGAGTTCCATGATATGAGGAAACTGATGGGCTATGTCATATATGTTAAACAAGCTGGATTTGTTAATGTTACTGTGTTTGATGGGAGAAATGAGTGTGGAGGGGGAGATTGGAAAGTTTATGATGTATCTTTTTTTGATTGTTGTGCAGATAATTTCACCATAACAGGCCTTTTGCCTTTTACTAGGTATGCTTTTTATATGAAAACTGTAACAGTTAATGCAGATTTTGGTGCTCAGAGTGTCATAAAATATTTTAGCACTCTTCCTGGTATCCCTTTCGCACCTTTTGAATTGACAGTAGATATTAATTCATATTCTGAAATCGCGTTAACGTGGGTACCACCCATTGCTAATGGTATATTATCTCATTATTTGGTTTCTGTTCACAAAGTTGAGGATCTGTTTACAGATATTGACTATTGTGAAGAAAGTGGCGCATACCCAGTGCAGAGTAACAGTTCTAAATATTTAGAATCTGATTCTGACCTCAACGACTACACAGACGGTGCAAAACAAGTTCTTTATGAATCTATGGAGTTATCAGACGAACAAAGAAGACGCTTGAATGACATTCACTTTGAAAATAAACTGCAAAATTATATTTATGTAAAATATACTAGAACAAAAAGAAATGCCTTGGTTTCAAAAGAATCTGAATCCAGCTTAGCTTCTCTTGTGTCATCTGATTCTCATTCTGGCAACTCCACTTATATCTCTAATGATATGGACTATAAACCAGATGGGGACACTGTTTTACTAAACAAAATTGTTTATGAACCTAGTCTTACTGTGTCAAATTTAAAGCATTTTACTCATTACTTAGTAGAAGTCCGAGCATGCCGCCAACTTTTATCTAATGAATCTTATAAAAGCTTTGATAAATATTGTAGTCCAAAGAGAAAGAGGGAAGTGCAGACATTTGCTTCAGTTGTAGCGGACTCCATTGCTGATGAATATCCATTACTGGAAGTATCTAATGACGAACCTGGCATGGTTACACTGAAGTGGAGTGAACCTGTTGATCCTAATGGCATGACCCTTTCCTATACTATTGAGTACAGGAGAAATGGTTACAGTTCTACTAAGGAGTGCATCAGTCAAAAGCAGTTTTTCAAAGCAGGAAAATGTCATGTTATTAAAAATCTAAGTCCTGGTAATTACAGCGTGCGAATTAGAGCCAGCTCGATGAATGGTGCAGGCCCTTATACATCTTTGAAATATTTCTTTATTAAAGACGATGCTTCCATTCATACTTACATTTACTTGGTGATCGGTACACTCCTCTTTATACTTCCTCCTTCCATAGCTTATTGTATTTATCTTAGGAAAACAGAAAAGAACAAACTATTTATATCAGTCAACCCTGAATATATTCACAAAACTTACGTTCCTGATGCTTGGGAAATCCCAAGGAGTCAAATAGATTTGATCAAAGAAATCGGTCAAGGTTCTTTCGGAATGGTTTATGAAGGCCTGCTAAATAGTAAACCTTGTGCTGTGAAAACTGTCAACGCCCAGGCTTCTGAGAGTGAGAAAAAGGAATTTCTGAATGAAGCTTCTGTTATGAAAGCCTTCGATACATTTCATGTAGTAAAATTACTCGGTGTAGTTTCTTTCAGTGAACCGGTTTTTGTTATTATGGAATTAATGCCTAATGGAGATTTGAAATCATTCTTGAGAGCTCACCGCCCTGATGTATGTGATCCCCATGTGCGTCCTCCTTCTCTCAGAAGACTTTTGCAAATGTCGGCAGAAATAGCTGATGGGATGGCTTATTTATCTGCTAAGAAGTATGTTCACAGAGATTTAGCCGCCCGTAATTGTATGGTTACTGAAAATTTAACTGTTAAAATAGGTGATTTCGGAATGGCGAGAGATATATATGTCGGAGATTATTATAGAAAAGGGACGAGAGGATTAATGCCTGTTAGATGGATGGCACCTGAAAGTCTCAGAGACGGTATTTTTCTCTCGGCTTCTGACGTATGGAGCTACGGAGTTGTTTTATATGAAATGGTCACCTTGGCTGCTCAGCCATATCCAGGCCTTACTAATGACCAAGTTCTTCGTTATGTCATGGATGGTGGGATTATCGAAAGACCTGAGAACTGCCCGGATAAGATGTTTATACTGATGAAGAAATGTTGGAAGCATAATCCTAACAATCGACCTATGTTTCTTGAAATTGTTGAAATGCTTCATGATGATGCCAGCCTCAATTTTCATGGAGTTTCTTTCTACGATAGTGATGAAGGGAAGGAGCACCGCAGATCAGCTGAAGCTAACACTGAAGTAGCCATAAG

>INR2_Rhagovelia_antilleana

GTGGCGGTGTTGGTGGCCTGGCAGATCGTTTGCTCGGCGGCCAGCACGGAAGGGGTCTGCCCCTCATTGGATATTCGAAACTCAGCCAAGAATCTTCAAAAACTTGCTGGTTGCCGTGTCATAGAAGGTTTCCTGCAAGTTGTCCTCATGGATCACGCCGTATTTTCAGATTTTCAAGGCATTTCCTTTCCAGAACTACGCGAAGTTACGGAGTACGTTATATTTTATCGAGTGGCCGGTCTTCGGTCTGTCGGTCAGTTGTTTCCAAATCTCAGTATAATAAGAGGAGACGAACTGTTCTTGGATTATGCTCTCAGTATCGTACAGATGCCACAACTGAAAGAGATCGGCATGGTCGGTCTTACTTCCATTCTACGAGGATCCGTCGCTCTTATTAGTAATCCAGTTCTCTGCTATACAGACACAGTGAACTGGAGTCATATTACCAAAGGGGAACCTATGGTAAGCAAGAAAACTAGACTGCCATGTCCTGCCTGCCCTAGACATTGTCAGAACTGCTGGAATAAGACTCACTGCCAGGCTGTTGGAAAACCATTATTGGCTGGTTGCCACGAGGAATGTGTTGGAGGATGTACTGGTCCTGAGCCATCTCAGTGCACTGCTTGCAAGAACCTTCTGCTGCACGGTGTTTGCACCAATTCCTGCCATCGTGATCAGTTTACTTACCTCAATAGACACTGTGTATCTGAGGAGGAATGCAAGAACATGCATAACACATTATCTCGCCCTGGAAAGCATCTCCCTCATTATGCAGAAGAGAAAAAACCTTGGTTCACATGGAATGGAACATGCATTCAGTCCTGCCCCAGAGGCTATGAACGAGATGACACTTCAGGATGTCGAAAGTGTCCTGGGGTATGCCGAAAGGTATGTCCTGGGACTACTGTTGATAGTATTCAAACTGCACAGAGCTTTCACCATTGCACCCATGTATCAGGTAACCTTGAAATTCAAATGCCTAGAGGAAGTCCAGAACTTATAGGAGAAGAATTGGAAAAAGGCCTTGGTCTTATAGAAGAAATTGAGGGCCATCTCAAGGTCATCCGTTCTTTCCCTCTTGTTAATCTTTACTTCTTTAAAAATCTCAGAGTTATTAAAGGGAAACCTTCATTCAATGAAAAAAGTTATAACTACTCCTTCCGTGTCCATGATAATCAAAACCTGCAAGAACTTTTTGATTGGAATCGCCCAGCTGGCAAGAATTTCACCATTGAAAACGGTCGTCTCTTTTTCCATTTTAATCCTAAACTATGTTTAAAATATATAGAAGAATTTGCTCAAATCTCCAACATTACTAACATTACAGATTTAGAAGTAGATAAAACTTTTAATGAAAAGATGTTTGCTTGCAATGTAGAGAACATACCATTACATGTAGGTGTAAAAACTGCTACTAGTTTTGTACTTGTCATTGAGAAATCTGACTATGTCAACATTACTAACTCTGAACAGGATTGGGTAAGGGTTTTGGTTTATTACAAAGAAGCTCCGTTTACAAATATCACTTCTTATTATGATAATGATGCATGCCGGGATGACGGCTGGAGGTTCAATGATGTAAAGACATATCCTGGGGAAGGAGATGAAAAAGTTATTGGGGATCTGTATCATCTAATAGCTCAGCTCAAACCTTACACACAATATGCTTTTTACGTTAGGACCTACAGCCTTGGAAGAGAAGTTGGCTCTCCTATACATTATGTTCGTACTTTACCAACAAAGCCATCTTCTCCTGAAAACTTGCTTGCTTACAGCAACTCTAGTAATGAGATCCTTATCAGTTGGTCACCTCCTTCTTACCCTAATGGAAAACTTAAAGCTTATGTTATTCGAGGAGTCCTACTTGAGGATGACCATGAGCTCCCTGCTAAACGAGATTTCTGCCAGCACGCTATACTTAACTTTCCTGAGGATACTACACCACCTCCAGATTTTATGACCTTAATAACTAGTAACAAAGAGGATTGTTGTGATAAAGGTAAAGATAGTTTAGAACCTAAGTTAATTTGTGATCAATCAGGGTTTGAAAAGAATGGTAAAATTTCTACGTTATTTTTCCATGATTCTCGAATTAAATCTTGTGAAAGATATATGTATACCCTTATCAATGAAAATGTTCTTAAGCCAAAATTCTCATCAAAGCAGTCCAAAGAGAGACGTTCAGCTATGAAACCTTTCAATGATATGACCTCTCATTTCAGTGATGAAATAGAGGGAGAAACAACCGAGTCATACAATAGTGATGGTACATTAAAAAGATTTACTGAGGTAGTAAATAATACCACCAAATTCACTTCTCTTAAAAATCTCATGCACTTTGGCAGGTATCTTATTGAAATTAAGGCTTGTCGAGAAAAAGTACCTGAGGAAGAAACAAAATATTTTGTCAACTCTCAATGTAGTGATACTACTTTTGTGACTTTTAGAACTAAGAAGTCCCTTCTGGCTGATAGAATAGACCAGAAAAGTATTATAGTAAAAGTGACAAACAAGAGTGCTTTAATTACTTGGAAGAAACCGAGTTATTACAATGGCATCATACTTAGTTATAAAGTAGAGCATAAAAGTATCGACAAAGACAATATTAGACCTATAATAGACTGCATTCCTGCCTACCCCAATTCATCTTATACAGTATCATTTTCATTGCATAATTTAATTTTTGGGAACTATGCTATAAGAGTCAGGTTAATTTCTTTAGCTGGTGAAGGCCCTTTTTCCATTCCTGTAAATTTTTCCATTGTTGATGATTCATATTCAAACATGATGGAAAAGCTTGCTGTGATAATATGTGTTACTATTATAGGTTTATTGGGAGCATTGCTTGGTGCCAGAGGCTTCGTGTACTATTATAAAAAAAGGTTAAAGGACAAGAATATCCTGATAGAAACTATTAACCCTGAATATACTAGTGTGACGGGATATTTAGAAGACAACTGGGAGTTATCTAGAGATGACATAGTATTGGTAAAAGAGATAAAGCAAGGTAATTTTGGGATGGTTTATGAGGGATTGCTCCTTCCTGAAAATCGCAGAGTAGCAGTAAAAACAGTTAAAGAAACTGCTTCTCCCCAAGATAATTATGAATTTTTGAATGAAGCCAATGTGATGAAAGCTTTCAGTGGTGCCCATCATGTTGTAAAATTGTTGGGAGTTGTATCGAGAGGAACTCCAGCTTTAGTTGTTATGGAGCTTATGGTGTTGGGAGACTTGAAGTCTTTCTTAAGACTAAGTAGAGATTCTCCTCAATATCCTCCTCCTTCACCTTCAAGACTAAATCTGATGGCTGCTCAAATAGCTGATGGAATGGTTTACTTGGAAGCCCTTAAATATGTTCACCGTGATCTTGCTGCAAGGAACTGTATGGTTTCAGATGACCTTACAGTAAAGATAGGTGACTTTGGTATGACAAGAGACATATATGATACAGATTATTATAGAAAAGGAAACAAAGGGCTTTTACCTATAAGATGGATGGCTCCAGAAAGTCTTAATGATGGTATATTTACCAGTTCTTCCGATGTTTGGAGTTATGGAGTGGTACTCTGGGAAATGGTTACCTTGGCTACACAGCCTTATCAAGGAATGTCTAACGAAGAGGTACTCCAACATGTAGTCTCAGGGAATAAATTAGATAGTCCCGTATACTGCCCTCCACAGCTAAAGATAATTATGATTTCCTGTTGGAAGTGGAAACCTAAACTTCGTCCAACATTTGCCCATATTTTGAATCTCCTCGAACCAGCACTTAACAATGAGTTCCGATCTGTTTCTTACTATCACGGTGTTGACAGGCACGAGAGTCAGAATTATGCTGATGAAAGTAGAGACAGTGAACACACATCTTTACTCCTTTTACCAAGCTCTTCATCGGTCGAGTTCCACCCTTCTCATAGTTCTAACCATGAGTGGAACATGGACACTAAACAATAA

>INR1_Rhagovelia_antilleana

AGAGTTCAAGGTCTAAGATCTCTTGGCAAACTATTTCCTAATTTAACTGTTATAAGAGGAAATACATTATTTTTAAATTATGCTCTGGTTGTTTATGAAATGCTGCAGTTACAGGACATTGGTCTTATTTCTTTAACAAACATTCTGAGAGGAGCTGTCTATTTTGCAAAGAATCCTATGCTATGTTTCGTTGAAACAATTGATTGGGATTTAATTGCTCCTCATAGTAAAGGAGAACATCATATTATGGGAAACAAGAAAAGCATTGAATGCCCTCTGTGTCCTCCAACAGTAAATAATAATCAATCTTGCCCTAAACAAGCCTTTAGTGGAGAACTCCTTTGTTGGAATATGAATAATTGCCAAAAAAGTTGTCCATCAAATTGTTATGGAACTTGCAATTTAGAGGGAGAATGCTGTCACACTTCATGTTTAGGTGGTTGTACAGGCAAAACTGAAAATTCTTGCTATGCATGTAAACATTTTTTGTTTAACGAGAGATGTGTTGAAAACTGCCCTACCGGAACAAATGAGTATTTAAATAGTAGATGTGTATCTGACAGTGAATGTTATAAAATGCCTAAACCCCGAGAAAGAACTGATTCAAAAACTGATAACAGGCATTGGAAACCTTTCAAAGGGAAATGCATTCTTGAATGTCCCCCAGGATATCTTGAAAAAGAATCCTCACTCAATGGAAAACTGAGGACAACATGTGAAAAATGTAAAGGTATATGCAAGAAAGAATGCCCTGCAGCTAATGTGGATAGCATTGCTACTATCCAAAAACTTAAAGGCTGCACCTCCATCAAAGGTGCCCTTGAAATTCAAGTTAGAGGAGGTAATATGGTAAGAGAATTGGAAGAAAATTTGAATACTATCGAGGAAATAGATGATTATCTAAAGGTTGTTAGATCATTTCCTCTTATATCACTTAATTTCTTACCTCAGCTGAAAGAAATTCATGGATACAAACTTGAAAGTTCTAAGTATGCTCTGGTTGTCTTAGATAACCAAAATCTTGCAGAGTTGTGGGATTGGAATCATCGCTCCACTGGTTTAAAAATTGGTAATGGGAGATTATTTTTTCATTTTAATCCCAAATTGTGCTTGGAAAAAATTGAAGATTTGAGAAAAAAAACTAATTTACCTGTCCACTTAGATTTGGAAATTGCAAGTAATTCAAATGGAGATAAAACAGCTTGTAATGTTGATGAACTTGTTGTGACTGTTTATAAAAAATCTCCAATGGCAGCATTAATTAGGTGGAGGCAATTTGAAAACCATGATCCAAGAACTCTCTTAGGGTATGTTGTGTACTCTACTGAAGCACCCAACAGAAATGTTTCCATGTATGATGGACGAGATGCTTGTGGTGATGATGGATGGAGAGTTGATGATGTTTCTGTTAGGGAAGAGCATGACGTTCCTGTAAGGGAAGAGCATGATGTTTCTGTAAAGGATGAGCATGATAATTCTGAAGAAAAAGAATTTAATTACATTATTACTCAATTAAAACCTTACACTCAATATGCTTTTTACGTTAAAACATATACCATTGCTACTGAAACACGAGGTGCTCTGAGTCCAATTCAGTATTTTCGTACAGAACCTTATATACCATCTGAACCTCGTTCTATTACAATTTATTCTAATTCGAGTTCTGAGCTAGTTCTTCATTGGAAACCTCCATTGCATCCTAATGGTAATGTTACTCATTACATTGTTTCGGCCGTTTTGGATCCTGATAATGACTTGCTAGACCAAAGAAATTATTGTTTGGAACCTTTAATATTTCCTGATACAAAAAGAATATCCATGAAAAAACTTAATCGAGACTCACAGGAGTTTAATGAGGAGAATATATCTGCTGATAGTGACTCCTGTTTCTGTAATCGAAAACTGATTGATAATAAACAAAGAGAGAAAGAAATACATTTTGAAGACCACTTGCAAAATCAAATATACATCAAAAGATATACTCCTAGTTCTTCAAAAGACAAACGAGCAGTTGGAACTGCACCATTTGATATTGCACATGAAGAACCTGCAGAAAATGAAAATTATATTGTTAATGGTGTTTATGAGCGTTTCCACCAGGTTGTCTATGGAAATAATTTTACATTTGTTATCCGAAATTTGAGACATTTTTCTCTTTATCAAATTGCAATTCAAGCATGTCGAGAATGGGAAGAAGATGAAGATAGAAGTAAACTTACTAATTGTAGTAAAGAAGGGATTATTGTTGGCAGAACTTTGCCTATGAGTGGCGCTGATAATATTGATAGCCAGCATGTATTCTGGGAAGTTTCTAATACAACCCATGGTATAGTTAAACTAAAATGGGAGGAACCCAATAACCCAAACAGTATGATTGTTACATATCAAGTTGAGTATTGGCGGACTGACATTGAAAATTACAAACCTACTGTTGAGTGCATTTCCAGAAGTCAGTTTAAGTCTATGGGCAATTCTTATGTTTTGAAAAATCTCAGCCCTGGAAATTATAGTCTGAGAATAAGAGCTACATCTTTGTCTGGTAATGGACAGTATACAAAAATCAGTTACTTTTACATTGAGGAGGACTCAGTTTCACCTGTATTGCAGACTACTATAATTGTTATAATTATTATGTTGCTTCTGTTTATAATTATTGTGCCATTCATTGTATTTTATGTGAGAAGGCATATTGAATATGGCCCGAATATAAAACTTATTGCTTCAGTAAATCCTGAGTATGTTCCATCTGGTAAAGTATACACCCCTGATGACTGGGAGGTTCCTAGAGAAAATGTGGAGCTTATCAGAGAAATTGGTCAAGGCTCATTTGGAATGGTTTATGAAGGATCACTTAAAACACCTGATTTACCTACTCTAATGCGTTGTGCTATTAAAACAGTTAGCTGCAATAGTAAAACCCAAGAAAGAGATAGAATTGAATTCCTAAACGAAGCGTCAGTAATGAAGGCATTCAACACCCACCATGTTGTAAAGCTGCTGGGTGTGGTATCACAAGGTCAACCTACCTTAGTTATAATGGAATTGATGGCTAATGGTGATTTAAAGGCATATTTGCGGTCCTGTCGACCTGACAGTGGTTATTTCCCTCAAACTCCACCTTCTCTTAAGGATACTCTAAGGATGGCTGTAGAGATTGCTGATGGTATGGCATATCTTGCTGCTAAAAAATTTGTCCACCGGGATTTAGCTGCCAGAAACTGTATGGTAACTGATGAATTAACTGTGAAGATTGGTGACTTTGGCATGACTCGTGATATTTATGAAACTGAATATTATAGAAAAGGTTCAAAAGGCCTTTTGCCAGTAAGATGGATGGCTCCTGAATGTCTCAAAGATGGTGTGTTTTCAAGCCTATCTGATGTTTGGAGCTACGGAGTTGTGCTCTATGAAATGGCCACCTTAGCTTCTCAGCCGTATCAAGGCCTTTCTAATGAGCAGGTATTGAAGTTTGTTATTGATGGTGGCGTTATGGACCGTCCTGATAATTGTCCTGATGCTCTGTATCATGTTATGTTAATGTGTTGGCAACAAAAACCTCGAGGCAGACCTTCATTTATGAGCCTAGTTGGAATGCTTAAAGAACATTCAAATCCTAATTTTGCTTTGGTATCATTTTATGATAGTGAAGAAGGGCAAGAAATAAGAGGAATAGAAGCAACTGAAGAAACACCCCTGTTTGTGTCGAGGGTTATTGAAGATTTTTCTCTCAGTGATGATGATTCTAGCAACCCAATCAAAAATTCCAGTAATACTGGTGATGAAGACGAAGTGGATGATGCTGATGATTACGGTGACATTAGTTTAAAAAATACTAGTGACAGTTGGATTCGTAGTGTTGGTCCACAAAACGGTGACGAATTTGATGCAGCACAAATGCCAATGATTTCTACCCATGCAGACTTATTGAGGTATCCAAGTAGTAGTCAAATAGATGAGACAGAAGCTGATAGAGAGAATTCAAATTTTGAAGTACTATCTCCATCCAGTAATAATATGAATAGAAATAATGGCCGCATAACTCCTAATGGTTGTATTAAATATAACGATGTAAAAACTACTGAATGTTAG

>INR1-like_Microvelia_longipes

ATGAGTTTTCATTCACTTGGTTGGTTTTGTATCTTCTACATTCTCCCCTGTTACATTTGTTTGGACTTTGACCAAATAGGAATCACAAAATCAGTCAGTCATTTCAATATTTGTTCTAATATTGAAATCTGGGGACCAGAGGATGCGGCTGTTTCACGTCTCAAGAACTGTACTGTGGTTGAAGGCTATGTCCGCCTCATGCAGTTTGATACCAAACTCGATCCAGACATCAAAGTCAGTTTTCCAAATTTAATGGAAATCACTGGGTATTTGATGGTGTTTAGATTGAGTGGCGCCGAATCACTTGGTGAAATATTTCCTAGTTTGTCTGTTGTACGAGGAGACATTCTTTTTGAAAGTTATGCTATTGTTATTTTTGAAACATTTAGTTTGAAAACCATAGGATTAACTGCATTAAGAGAAGTCATCGATGGTGATTTGTTTATTGCTTCCAATGTCAACTTATGCTTTGTAGATACTCTGGATTGGAGTTCTATAGCTTCAGGAAATAGAAAGCATTACATAGCTGGAAACAAGAACCCTAACGAGTGCCCAGTTTGTGATGACTGTCCCAAGTTAAATGGGAAAGTATCTTGTTGGAGTCATGTTGACTGCCAGACAGTTTGTCCTTCTTCTTGCACCAAGAGTTGTACTGATGATAAAGAATGCTGTCACTCATTGTGTCTCGGGGGTTGCTGGGGCATGGGAGCCGATAAATGTTTTAGTTGCAAACACTATCGATTTAAAGACACTTGTGTCGATTCTTGCCCTCGAGGGACATACATCTTCTCAGGGTGGAGATGCTTACTGGAATCTGAATGTTACAACATGAAACCGGTAAATGATAAATATTGGAAACCTCATGATGGGCAGTGTGTATTAGACTGCCCGCCTCAATACTACCAGCAAGAAATACTCAATAATAGAGAAAAGCAGAAAAAATATGTGTGTCGCAAATGTGAGGGAGGAACTTGTAGGAAGGAGTGCCAGGGAGCTCTCATCTCTAATGTAGCAGACTCTGAGAGGATGAGAGGCTGCACACACATCAAAGGAGGTGTTCTCATCAGTCTCTCTGGACATGGTAAAAATATATTACCCCAACTTGAGAAAAATCTTAAAGACATTCAAGAGATATCGGAATACCTCAAAATATCAGATTCATATCAACTCACTTCTCTAAACTTTTTGAGGAATCTGAAATATATATGGGGAGAGGAGAAGTATAAGGGTAAATACAGTTTGATTGTTATAGGGAATAAAAATTTAGAGTTATTATGGGATTTTAACACCAAGAGAAGCAGCTTGAAAATCAATGGTGCTGTTTATTTCAGAAGTAATCCAAAATTATGCCCGAGTGAAATTGAGAAACTGGTCAATGAAACTGGTGTTTCTCCTGCTGATTCATTTTATCCTCTCCAAACTAATGGGGTTAAAAATGTTTGTAACCTTCGCGAGCTTTCTGTTTCAGTTGAAAATATAACTTCTAATTCTACTAGCATATCCTGGTCACAATTTGATTATGTTGACCTGAGGAAATTATTGGGTTATGTTATTTCTATAAAGGAAGCTCTGTATGATAACGTGACTGTATTGGATGGTAGAAACGAGTGCGGGGGAGGGGACTGGAAGGAGTATGACGTTTCTTCAGATAACTATACGATTAGAAATCTCAAACCTTTCTCTAGATATGCCTTTTATGTTAAAACTCAGACTGTGAATGCTGATTCAGGAGCTAGGAGTCCCATTACATATTTTAATACTCTACCTAGTAAACCAACTATACCTCTCGCTTTAACTGTTAAAACTAACTCTAGTTCAGAGATGACTATGTCATGGGCGCCTCCCCTGTTTCCTTATGGAATTATTTCGCATTATTTAATATCTATTTATAGAATTGCAGATTCTGCAATCGACGGTGATTACTGTGAAAATAAAGTTTCTTACAGCATGCCAACTAATAATTCTAAATACGTAGAACTAGATTCAGGTTTTAACGATTACTCAGATGATACAAAGCAAAATTCTTATGAATCTAGTGAACTCTCTGATGAACAGCGGAGACGTCTCAATGATATACATTTTGAGAATAAACTGCAAAACTATATTTATGTCAAAAGACCTAGAATAAAAAGGTATATATTATCTAATGAAACAGAAAACAATGTTTCCTTTGACTCTAAAAGTGATCAACTAGTATTTATGAAAGATAAAGTTGTTAACAGAACTTTCTTTTATGTTTCGGGCCTAAAACATTTTACCAAGTACATGGTGGAACTAAGAGCTTGCAGAGAGTTGATGAAAGATGAATATCGTTTTAATGCAGAAGATTACTGCAGTGATCCCTCTTATAGATATATTAGAACCCATCCTCTTCCTTCAGCCGACCTCATCCCTGATAAATATTTATTATTTGAGGTTTCTAGAAAAGATCATGGAACAGTTTCATTGAAATGGGAAGAACCTGCAGAACCCAATGGAAACGTCACTTCTTACACCATTGAATACAAGAGAAGTGGATATTCCTCCAATTCTGAGTGTGTCAATCAGACTCAGTATAAATCAGGTAAATCCTATATTCTGAGACAACTGAGACCTGGTAACTACAGTGTCCGCATCAGAGCCAACTCTCTTAGCGGTGAAGGTCCGTTTACTTCTCCTAAATATTTCTTCATCGAAGATGATACGTCTATCTTTGCTTACTTATATTTGATATGGGGGACCCTCTTCGTTATTATGCCTGTTTCTATCGCTTACTGTGTTTATTTTTTCAAGAAAACAGAAAAGAATAAATTATTTATATCTGTGAATCCTGAATATTCACACAAGACCTATGTTCCAGACGCTTGGGAAATCCCCAGAAATCAGATAGATTTAATTAAAGAAATCGGACAAGGTTCCTTTGGTATGGTGTATGAGGGCTTGTTGAATAACAAGCCTTGTGCTGTTAAAACAGTCAATGCCCAGGCTTCTGAGGGGGAGAAGAAAGAGTTTTTGAATGAAGCCTGCGTGATGAAAGCTTTTGATACTTACCATGTTGTCAAGTTGCTTGGTGTTGTTTCCTTTAGCGAGCCTGTGTATGTTGTTATGGAATTAATGCCTAATGGAGATTTGAAGTCATTTCTGCGATCCCACCGCCCTGACGTCTGTGATCCTCATGTCAACCCTCCTTCTATGAGAAGACTTCTGCAAATGGCTGCTGAAATCGCTGATGGAATGGCATATCTGTCTGCTAAGAAGTATGTCCACCGGGATCTCGCAGCTCGTAATTGTATGGTCGCAGAAAATTTAACTGTTAAAATAGGTGATTTCGGGATGGCTAGGGATATTTATGTTGGCGATTATTATAGAAAAGGTACTCGAGGCTTAATGCCTGTGAGGTGGATGGCTCCAGAGAGTCTGAAAGATGGAATATTCTTATCTGCTTCTGATGTTTGGAGTTATGGAGTTGTCCTTTATGAAATGGTTACTTTAGCCGCTCAGCCTTATCAAGGATTGACCAACGACCAAGTTTTACGATACATAATGGACGGGGGTGTCATGGAACGGCCAGAGAACTGTGCCGATAAAATGTGGAATCAAATGAAAATGTGTTGGAGACAAAACCCAGGAGATCGGCCTACGTTCCTCGATATCGTGGAAATGTTGCATGAAGATGCGAGTCTTAGCTTCCATACAGTCTCTTTCTACGACAGTGAAGAAGGCAAGGAGCACCGGAGAAATGCCGAGGCTGCTTTAGCTCTAGCCCACAGGTCC

>INR2_Microvelia_longipes

ATGTCGTGGCTTGTGCTGGTGTTGGTGGCCTGGCAGATAGTTTGCTCGGCGGCCAGCACTCAAGGTGTTTGTGGTTCCATGGATATTCGAAATCACCCGTCAAAACTTCGTCAACTCTCCGGGTGTCGTGTTATAGAGGGGTTCCTACATATCGTCCTTATGGACAATGCAAATGCAGCAGACTTTCAAGGAATTTCGTTTCCAGAACTCCGCGAAGTGACGGAGTTTGTAATTTTTTATCGAGTAGCCGGTTTGAGATCAATCGGACAATTGTTTCCAAATTTGAGTCTAATTCGCGGAGACGAATTGTTCCTAGATTATGCTCTCAGTATTGTTCAAATGCAAAGTCTTCAAGAAATTGGTCTTGTGGGGCTTACAACAATCCTACGAGGATCTGTTGCACTCATCAGTAATCCAGTTTTATGCCACACGGACACTATCAACTGGAGCCTGATAGCTAAGGGAGAGCCGTTGATCAGTAAGAAGAGTAGAATTGCCTGTCCAGCCTGCCCTAGGCACTGCCAGAGTAATTATTGCTGGAACAAAACACATTGCCAGGTTATTGGGAAATCATTTGCTGAGAGGAATGAAGGATGTCATGAGGAGTGTGTGGGAGGCTGTACTGGACCAGAACCTAATCAGTGTACAGCTTGCAAGAACCTCCTCCTCCAGGGAGTCTGCACTAATGCCTGTCATCAGGATCAGTTTACTTATTTAAATCGGCACTGTGTCACATTAGAGGAATGCAAAAATATGCACAAGACGTTATCGCGACCTGGCAAGCACTCCAATCCTGATGATATGAAACCTTGGTTTACGTGGAATGGAACGTGCATAAATTCCTGTCCAAGAGGCTTGGAGAGAGACGATAAACTTGGGTGTCGTAAGTGTAAAGGAGTGTGCAGAAAAGTCTGTGGCGGCATCACTGTGGACAGCATCCAAACTGCACAACTCCTCCAACGCTGTACTCATATCGCCGGCAACTTAGAGATTCAGATACAATCTGGAACTCCGGAACTTATTGGAGATGAACTTGAAAAAGGTCTGGGTCAGATTGAAGAAATAGAAGGTTCTCTTAAAGTCATCCGATCTTTCCCACTAGTAAATCTTTACTTCTTTAAAAACCTCAGGGTTATAAAAGGCAAGGATACCTCTAATGAAAATAATTTAAGTTCATTTCGAGTTCACGACAATCAAAATCTTCAAGAATTGTTTGACTGGAACCGCCCTGCTAAAAAGAACTTTACTATCGAAAATGGTCGTCTGTTTTTTCATTTTAACCCAAAATTATGTTTAAAATATGTGGAAGAAATGGCTCAAATAGCGAATATAACGAACATTACTGATTTAGAAGTGGATCAAACGTTTAATGAAAAAATGTTTGCTTGCAATGTGAAAGATATCCCAGTTCATGTAGGGGTTAAATCTGCCAACAGTTTTGTATTAGTTATAGGAAAATCAGATATTGTCAACGTGTCGAGCTCTGAACAAGATTGGGTCCGGGTTTTGGTATACTATAAAGAAGCTCCATCTCAGAATATAACTTCGCACTATGATGATAATGCGTGCCGGGATGACGGATGGAAGTTTAACGACGTGAAGACACCGTCGTCATACTTGGTAGACGACGGCGACGGAGCGACAACTGGTGATCTTTATCACTTGATAGCTCAATTGAAACCCTATACACAGTACGCTTATTACGTTAGGACTTATAGTTTTGGACGAGAAGTTGGATCCCCGATCCATTATGTGCGTACACTTCCAACCAAACCATCTTCACCTGGAAACATCCTAGCTTATAGCAATTCTAGCAACGAAATAATGCTTGGATGGTCTTCTCCTATAAACCCTAACGGAATGCTAAAACAATATATCATTCGTGGTATTCTGCTAGAGGATGATCATTACTTACCTATGCACAGAAATTTCTGTCTAAACCCAATTCACAATTTCCACGAAGAGACTACTATACCTCCTGAGTTTATGACACTAATCAAAGGTAAAAAAGAAGAATGTTGTGATAAAGGNNNNNNNNNNNNNNNNNNNNNNNNNNNNNNNNNNNNNNNNNNNNNNNNNNNNNNNNNNNNNNNNNNNNNNNNNNNNNNNNNNNNNNNNNNNNNNNNNNNNNNNNNNNNNNNNNNNNNNNNNNNNNNNNNNNNNNNNNNNNNNNNNNNNNNNNNNNNNNNNNNNNNNNNNNNNNNNNNNNNNNNNNNNNNNNNNNNNNNNNNNNNNNNNNNNNNNNNNNNNNNNAGGAGGTCTACTTCTGTGATAGATAGAAACCCATCCAACAATTTTGATGATTTTGATGGACTTGAAGATACAACTTATAACAGTGATGGGACTTTAAAAACCTTTACTATTTCCGTTAATAATAAAACTAAATTTACAACTCTTAGTAAACTTTTGCATTACGGAAGGTATCTTATTGAGATTAAGGCTTGTAGAGAAAAGGTCCCTGAAGAGGAAAACAAATATTTTTTGGACTCTCAATGTAGCGATACTTCGTTTGTTACGTTTCGTACTCAAAAATCTCCGGGAGCTGATGTAATAGATCAAAAAAGTATCAAAATAAAAGTCTCCAACAAAACTGCTACAGTGACGTGGCGAAAGCCGAGTCTTTACAATGGTGTCATTCTTAATTATTATTTAGAGTATAAGCCTGTTGATAAAGATAACATCAGACCTAAAATTGAGTGCATTCCTGCACTTCCGAACTCAACCTCTAACACAGCTTCATTTTCTTTGCACAATCTTAATTTTGGCAACTATGCTGTTCGACTAAGGGTGGTATCTCTCGCTGGAGAGGGACCATTTTCTGGCTCGGAGTCTTTTTCTATTATTGATGATTCTAATTTAAGCTGGTTAGAGATACTTGCTGTGATAATGTTAGTTGCAGTTATAGCAGTACTTGTAGTTTTCGTTGGTGCTAAAGCCATCGTATTTTATTATCAGAAAAAAATGAAGGGAAATAACATTCTTATAGAAACAATTAATCCAGAATATACAAGTGTAAATGGGTATGTTGAAGACGACTGGGAGTTATCCAGGGAAGATATAATATTAGTTAAAGAAATAAAACAGGGTAATTTCGGTATGGTGTACGAAGGTCTCCTTCTGCCAGAGAATCGTAAAGTGGCTGTGAAAACTGTGAAAGAAACTGCGTCTCCTAGAGACAATTCTGATTTTTTGAACGAAGCAAACATCATGAAAACATTTAGTAGTGCTCATCATGTTGTTAAATTATTAGGTGTGGTCTCTAAAGGAAATCCTGCTTTGGTTGTTATGGAGCTGATGGTGTTGGGAGATTTAAAATCTTTTCTAAGGTTAAGTCGAGATTCACCGCAGTATCCTCCTCCTTCTCCTGCAAGATTGAATTTGATGGCTGCACAAATAGCTGATGGAATGGTTTACTTAGAAGCCTTGAAATTTGTCCACAGGGATCTGGCTGCTAGGAATTGCATGGTGTCAGAAGATCTAACTGTCAAAATTGGCGATTTTGGTATGACTAGAGATATCTATGATACTGATTACTATAGAAAAGGTAATAAAGGACTTCTTCCTATCAGATGGATGGCTCCTGAAAGTTTGAATGATGGTGTATTTACAAGTTGTTCTGATGTATGGAGCTACGGTGTTGTACTGTGGGAGATGGTAACACTAGCCACACAACCTTACCAAGGTATGTCAAATGAAGAAGTTCTTACGTATGTTGTCTCTGGAAATAAATTAGATATTCCTATTTACTGCCCTCCACTTTTGAAAAAGCTCATGGTCAGCTGTTGGAAATGGAAATCTAAATTAAGGCCAACGTTTGTCAATATCATAAATATTTTGGAACCA

>INR1_Microvelia_longipes

ATGAAGATAATGACGAAACCGGGGCATAGGCCTCATGTTGGCAGATACTTTAATATTGGACTGCAGAACGTTATGTACTGGAAAGGTGGATGTAATATTGACAGTTTCTTATTCCTAGTTGTTTTGTCAACATGTTTTATCAAAGCGTCCAGTCATGACATTTCATCCTTGATCAGTAATAGTCCTAGCTATAATCATGTCGATAATGACGATCCCTCAGTTTGTTCGAGTAAAGACATTAGAAATACCGTCAGTATGTTTGAGCAACTACAAGGTTGTAAGGTTGTTGAAGGATTCGTACAGATAGTATTAATAGATAAGGCTGACTCATCGGACTACTCCAATGTATCCTTCCCTGAGTTGAGAGAAATCACTGGATATTTATTACTTTATAGGGTTCAAGGGCTAACAACCCTCGGAAAATTGTTCCCGAATTTGACTGTTATTAGAGGAAATACTTTATTTTTAAATTATGCTTTAGTTGTATACGAGATGCTTCAATTACAGGATATTGGTCTCAACTCACTTACTAATATTATAAGAGGTGCTGTGTATTTTGCCAAAAATCCTATGTTGTGCTATGTTACTACAGTTGATTGGGATATCATAGCTCCGTATGGCAAGGGAGAACATCATATCATGGGAAACAAGAGAACAAATGAATGTCCTCTGTGCTCTCCATCATGTCCTAAGAGTATTACTAGTGGTCAGCATGTATGTTGGAACCTTGATGCCTGTCAGAAAATTTGTCCTAACTGCAATAATGGAACCTGTTACCTAGAAGGAGAATGTTGCCACCCATCTTGTATAGGAGGATGTTCTGATAAAACATCAAAATCTTGTTTTGCTTGTAGAAATTTTCTTTATAATAATGACTGTCTTGATAGTTGTCCTCAGGGCACATACAAGTACTTAAATAGAAGGTGTGTTTCTGAAAAAGAATGCTATAATTTACCGAAACCTCGTGAAATCACTGATAATAAAGTTAAACAGAAACCTTGGAAACCATTCAAAGACAAATGTCTACTTGAGTGTCCTTCTGGTTATGTTGAAAAAGATGAAAGAATTAATGGAAGGACAAGAACTATTTGTGAAAAATGCCGGGGTATTTGTAAAAAAGAATGTCCTGGAGCCAATGTAGACAGTATTGCTACTATTCAAAAACTCAAAGGATGTACTTCAATTAAAGGAGCATTAGAAATTCAAATCAGAGGAGGCAATATTGTGAAGGAGTTGGAAGAAAATCTGAAAACCATTGAAGAAATTGATGAATATCTTAAAATTGTCCGCTCTTATCCTTTGATATCTCTAAATTTTCTGTCACATTTGAATGTCATTCATGGATATAAACTGGAAAACAATAAGTATGCTCTAGTAGTATTGGACAATGCTAATCTTGTTGAGCTATGGGATTGGTCTCACCGTCAAACTGGTCTACAAATAGAAAATGGTCGATTATTCTTTCATTTTAATCCCAAACTTTGTTATGAAAAAATTGACACCTTAAGAAACAAAACTAATTTATCGACACATTCTGATTTAGAAATAGCAAGGAATTCTAATGGAGACCAAACTTCTTGCAATGTTGAAGAGCTCCAAGTTAGTGTTTATAAAAAGACATCCATGGCTGTTTTAATTCAATGGAGACAGTTTGAGAATCATGACCGTAGGACTCTTCTTGGTTATGTTGTTTATTCCGTTGAGGCTCCTTATAGAAACGTTTCTATGTATGACAGAAGAGATGCATGTGGAGATGATATGTGGCGAGTTGATGATGTTTCGATAAAAGAAGACCTAGATGCGTCAGAGGAAAAAGAAGTCAACCATATCATTGCTCAGTTAAAACCTTATACTCAGTATGCTTATTATGTTAAAACTTATACGATTGCTACTGAAACTAGAGGTGCTCAGAGTCCTATACAGTATTTTAGAACTGATCCTTACACTCCAACAGAGCCTCACTCGATCACAATATTTCCTAACTCAAGTTCTCAACTACTGATTCATTGGAAACCTCCGTTAAAACCAAATGGAAATGTTACTCATTATATTATTAAAGCTCAGTTGGTCCCAGATGATGAAAAATTTCTTGAAGAAAGAAACCACTGTTTGGAACCACTGGTGTTTCCTGATGTTAAAAGCGTACAAATGAAAGAAACAAATGAACAAGTTGAAGATATGTATGAGGAAAAAGTAATATCTCCCGACAGCGAGTCATGTTCATGCAATAGAAAACTTATTGACAATAGGTTGAGGGAGAAAGAAATTCATTTTGAAGACCATCTTCAGAATTCTATTTATATTAAAAGGGATAAGAGAGATAAGAGAGCTGTATCTAAAATGTCATCATTTCCTTTTGAAAGTGAAGAGGATAACGTTGAATCTGAGGAAGAACATTTTTCAACTAATGGAGTTTACTTAAGGTTCCGTCAAGTTGTGTATTTTAAAACATCATTTGTCATTAGAGATCTAAAGCATTTTTCTCATTATAAAATATCTATCCAAGCATGCAGAGAGTGGGAAGAGGATGAAGATAGATCAAAAAAAAATTGTAGCAAAGAGGGAGTTATAGCTGGTCGAACATTACCCTTAGAGAATTCAGATGATATAGATAGTAATGAGATTGTTTGGGAAGTGTCAAATACCACTCATGGAATCGTCAGAATCAAATGGCAAGAGCCTGCTAATCCTAACAGTGTTATATTAACTTACCAGGTTCAATATTGGAAAACTGACCTGGAAAATTATAAACCCACTGAAGAATGTATTACAAGGAAACAGTTCAAGTTACTTGGAAATTGGTATACTCTCAAGAACCTGAGTCCTGGCAATTATAGTTTTAGAATCAGAGCTACTAGTTTGTACAAACCTGGCAATTTCACCAAAATCAGTTACTTTTTCATTGAGGAAGACTATGTATCTCCAGTCGTGCAAACAACCCTCCTCATCGTGTGTTTGATGCTTTTATTTTTGTTTGCAAGTATTCCTATTATTATTTGCTTTCTCAAAAGATATTACCAGATGTTTGACTATAACACCAGATTGTTTGCTTCAGTTAATCCTGAATACGTTCCTTCTGAAAAAGTTTACACTGCAGATAGCTGGGAGGTGCCGAGAGATAGTGTTAAATTACTACACGAGTTAGGACATGGTTCATTTGGAATGGTGTATGAAGGAAAACTAATGACTAGTGGCTGCCCTCAAGGGATTCCTTGTGCTGTCAAAACTGTCAATAGTAACAGTAAAACTAGGAATGAATTCCTAAATGAAGCTTCAATTATGAAGGCTTTTCATACACAGCACGTCATCAAACTGTTGGGTGTAGTATCCCATGGACAACCTACTCTCGTTATAATGGAGTTAATGGAGAAAGGAGATTTAAAAGCGTACCTACGGTCTTGTAGAAAAGACAGTGGGGTCGTGCCCCAACCTGTCCTTACCGTTAAGTCTTTGCTGAGGATGGCTGTGGAAGTGGCTGATGGAATGGCTTATCTAGCTGCTAAGAAGTTTGTACACCGTGACTTAGCTGCGAGAAACTGCATGGTATCAGGAGACCTTACTGTTAAAGTTGGTGACTTTGGTATGACACGAGACATATATGAAACAGAATATTATAGGAAAGGAACAAAAGGTCTATTACCTGTTCGTTGGATGGCTCCTGAAAGTCTTCGTGACGGAGTATTTTCTTCTCTATCAGATGTTTGGAGCTACGGTGTTGTGCTGTATGAAATGACCACTCTTGCCTCCCAACCCTACCAGGGATTAGCCAATGAGCAGGTTTTAAAGTATGTAGTAGAGGGAGGGATAATGGAAAGACCTACTAACTGTGACGAGTCGTTATACACTCTAATGATGATGTGTTGGCAGCAGTCACCCAAGGATCGTCCTTCATTCTTGAACATTGTTTCCATGTTAAAAGATCATTCCGGTCCTGATTTCCCTTCCGTTTCCTTCTACGATAGTGAAGAAGGGCAAGAGATCAGAGTTTTAGAAGCCAACGAAGAGACTCCTCTTATTCTCCAGTTCTCTTTTAAATCTGAATCCGAAGTTTTGTCTTGTTCCAATCAAGAGGACAATAGCCGACAAAAAGTTGATAATGGCGGGGCTGAAAGCTACGGTGATATTAATTTAAGATATGCCAGTGAAAAATGGATAAGAGAAATAGATGATACATTATTAAAAGAATTTGAAACAGACTGTGAAGATTCAACTGTTTCTCCTTGCCTGAGAATCAAATCTCCCGAGAATCTTTCTAACAAAATGGAAGAAGCCAGTTGCAGTTCCGTTAGAATTAAGAATTAA

>INR1-like_Mesovelia_furcata

TTATTAAACTATGGAGATAAAGATTTTTCATTTCCGAATTTGAGGGAAATCACTGATTACCTTTTCATATTCCGCGTAAGTGGCTTACGATCTGTCGGTGATTTATTTCCTAATCTCTCTGTTATTAGAGGAAACAATCTCTTTCAAGGTTTTTCTTTCCTTATTTTCGAGAACGTGCTGTTGGAAAATTTGGGACTCTCTTCCCTAACTAAAATTATCAAGGGAAGTGTGTTCATTGCTAAGAACCCGATCCTTTGCTACATTAACACCATCAAATGGAATATAATTACCAGCCCTGAAACGATAAATTACATAGCAAATAACAGAGCTGTGAGCGAATGCCCCTCGTGCTCTGATGAATGTCCTGCTGTAGATGACAGAAGTGTATGCTGGTCCCATAGTAAGTGCCAGAGACTATGTCCATCAGATTGTTCCTCCTGCACATCTGACGGTAAACAATGCTGCCATAGTGGCTGCTTGGGTGGCTGTGATATCTTACAGACTAACCAGTGTGATTCATGTTCACGAGTCTTGAGTGGTGATAAGTGTGAGAGTTCTTGCTCTGCTGTTAACGGTGTAGAATTCATGAACTGGCGTTGCATCAAGAAAAAAGATTGTTACACAAAGCCTGGAAGCTTAAATAGAGTTCCCATGAAGCTCTACCAGTCTCAGTGCTTGACTGACTGCCCTGTAGGTTTCACTGAGGAAACTGTTCTAGTTGATGGCTTGTCACGGCTTGAGTGCAAACGATGCCCTGGTGGAAAGTGTCGCAAAGTTTGTGAAGTGTTTTGGACTATAAAAACAGTCGAGGATGCTCAGAAATTAAAAGGTTGTGTTGTTATTAAAGGTGCCTTGGTTCTTCAGGTGTCTGGAAACATTAAAGGTGAGTTGGAAGACAGCTTGTCTTCTATTGAAGAGATCACTTATTACCTGAAAGTTACGGAATCTTTCCAGTTGACATCTCTGAATTTCTTAAGGAATTTGAAAAAAATTCACGGATTAAAGAGAGAGCAAATGCATTATAGCTTAGTTGTTATGGAAAATAAAAATCTCGAGCTTCTTTGGGATTGGGACCATCGAAATTCTACTTTGCAGATAAATGGATCAATATTTTTCCACTTCAACCCAAAATTGTGTAAAGACCAAATCGACAGGTTGATCAAGGAAACTGGAGTGATTGACACCGGCAATTCCTTTGTTTCTTCTCTTACTCATGGAGTAAAAACTGTTTGTAATATAACTGAACTGGAAATTGTTGATCAGATTATTACTCCTCAGTCTGCGACAATAGTTTGGAAACAGTTTGAATTCCATGATTTGCGTAAACTGCTTGGGTATGTCATCTACTATAAAGAGGCTCAGCATAGAAATGTTACTATCTATGATGGGCGCTATGTGTGTGGCGGGGGTGATTGGCACAGGCTTGACAACATATCGTACACAGAGCAAATTGTTATCAATCCCCATCTTACTCGTAATTTGTCCGGGCCAGCATATAAGCCAGTCCCGTCCAACCATTCAATCTCTTTAATGCGTCTCAAGCCATTCACCCAATATGCCTATTTTGTGAAGACCTTAACAGTTCGAGCTGACAGGGAGGCTCAAAGTGCTATTCATTACTTTGAAACTTTACCTGATTCACCATCTAAACCCCAGTACCTAACTGCTGTATCAAATTCCTCTTCACAAATAGCACTATCATGGAAACCCCCTCTACATCCCAATGGCTTGGTCACTCATTACGCCGTGACAGTTTATATTCTTCGTGACATTGAAGATACAGAGCTTGATATGTGTCGTGATGAAAACAAGGAAAAATACAATGCCACCTATGATGTTGGCAGTTTTCAGTCTAAAACAGCTGGAGAGAGTCCTGAGACTGAGTTTTATTATTACATGAGAGATGATGGTCGTCCTCTAACCGATGAAGAAAGAAACCGCGCCCGAAACATGGCAATATATTTTGAGGATATGCTTCAGAATAGAATCTATATCAAAAGATCAAATGCCACTGCTTCCCCTCGTCCACGCCGCTCTATATTTGCGGAGCTGTATAAAGGCAAAGAAGAGATTGGCAAATATCTCGTTTGGGGCACGTCTTTTCTTCTGTCTAATCTAACACACTTCACTCGTTACGAAGTTGAGGTTCGTGCTTGTAGGGCCTGGCAAGCTGGTGAAAATCAAACCAGTGATCGAAATTGCAGTATTGAATGGGCTTCAGCTCAGGTACGAACTCTTCCCATGGAGGATGCTGATGGTCTGGATGGTACATATCCCACAGCAACTGTTTCAAATTCAACTTCCGGCAGTGTCAATGTTAGGTGGCAGGAACCAACCATTCCTAATGGATTTATTAAGTACTTTGTTGTGGAGGTGAAACGTCTTGGCCCAGATGCTACAATTCCTGTATGTGTATCACGGAAAGAATACTTATCTAGTGGCAGATCTTTCACAATTCGCCACTTAGCACCTGGTAACTATTCCCTTCGGGTATGGGCTGATTCTCTTCATGGACTTGGATCACCATCTGAGATTGTTACCTTCGTCATCCCAGATGAAAACCATTTGGCAACTTACCTCCTTTGGATTTTTGGTTCAGTTGTCAGTATTGCTCCATTTGTGATTGGAGTTACTGTGTGGCTAATACATCGAGCTGATGAACGTCGCCTGACAAAAATGATTATTTCTTTGAATCCTGAATATGAAGCAAGTCAGCGTCCAGGATATGTCCAAGATGACTGGGAGATTCCAAGAGCAAATGTTGAACTTCTTGAAGAAATCGGTCAAGGCTCGTTTGGCAAGGTTTATGAAGGTACTTTAGTTGTTGGAGACTCTGTAATGAGGTGTGCTATAAAAACTATCACTGAAGGAGCAAGTGAGCGTGAAAAAGCAGAGTTTTTGAACGAAGCCTCTGTCATGAAAGCATTTACTCCATATCATGTTGTGAGACTCCTGGGTGTGGTGTCTGTTGGCTGCCCTGTCTTCGTTGTGATGGAACTAATGACATGCGGCGATTTAAAATCTTACCTCCGCTCTCGCCGGCCAGATGCTGCTGACCCAACTGTCAGACCCCCATCGCTTAGGCGCCTATTGCAAATGGCAGCTGAAATTGCTGATGGTATGGCTTATTTGTCATCAAAGAAGTATGTTCATCGAGATCTTGCTGCTAGGAATTGCATGGTTGCTGAGGATCTAACTGTTAAAATAGGAGATTTTGGCATGGCCAGGGACATATATGAAGGAGACTATTACAGAAAGGGAACAAAAGGTTTAATGCCAGTCAGGTGGATGGCCCCGGAAAGTCTTCGAGACGGTATTTTTCTTACGCTGTCTGATGTCTGGTCTTATGGAGTAGTCCTGTACGAAATGGTTACTCTTGCTGCTCAACCTTATCAGGGACTCACAAATGATCAAGTTGTGAGATACATCTTGGATGGAGGTACTATGGAGCGCCCTGAGAACTGTCCTGAGAAGTTGCATGTTCTGATGCGGCAGTGTTGGAGGCAGAATCCATGTGACCGGCCTACATTCCTCCAGTTGGTCACAGCTTTGCATCAAGACACATCCCACTCATTCCGTCAGGTGTCATTCTATGACTCAGAGGAAGGACGGCAAGCTAGAACATCTGAGGATGCATCAGGGACATTGGAAATATGA

>INR2_Mesovelia_furcata

ATGGATATCCGCAACTCGCCCCGCCAGCTGGAGACGCTGCGCAACTGCTCAGTCATCGACGGCTTCCTCCAGGTCGTCCTCATCGATGGCTCCTTTTCACGGCGTTCCGTTCGAGAGTCCGATTACGACGGACTCGTCTTCCCAAAGCTTAGGGAGGTTACTGGCTATGTCATATTTAACAGCGTATCAAAACTTCGATCCCTGGGCCAGCTGTTTCCGAATCTTAGTGTTATCCGAGGTGATTTCCTGTTCATGAATTTTGCCCTCTTCGTCTTCAAGATGCCAGACCTACAAGAAATCGGACTCTCGTCTCTCACAACCATCGGTCGGGGTTCTGTTGCCATCGTTAACAATCCAAAACTATGCTATGCGAGCACTGTGGACTGGAACCTCATAAATGTTGGTGAACACTATCTGAATTTGCGCAATCTCTCCTGTCCGCCATGTCCCAAGGACTGTCCTAACAACAACTGTTGGAATCAAACAACATGTCAGTTGACTTCAAACAAGTCCCAAAAATGCCATCCGGAGTGTCTTGGAGGCTGTTGGGGACCAGGAGCTGATCAATGTAATGCCTGCAAACATTTTCGTGTTGACAATACTTGCAGCAAATCTTGTTCAACTGACAGGTACAACTATCTGGATCGCTTCTGTGTAACCAAGAAAGAATGTCTGGCTGTCGAAACAAATATGTCAAAACGACCAAGACATTGGCTCGTGGGAGGAGGGAAGCCGTGGTTTCCCTGGAACGGTCTGTGCATATTTGAATGCCCATCCGGCTATGAGCGAGTACCAGAAGGATGCCGCCCATGTCCCGAGCCTTGTCCTCGTATCTGTCCACCAGCACGCATCGATTCAGCTCAAGCAGCACAGAGACTCCGTTCTTGCAATTTCATTGACGGGAATTTACACATCGAAATCATATCCGGAAGTAAAAGCTTCATAGCTGAAGAACTGAGACTGGCTCTCAAAGATGTCTCGGTCATAAGTGGAATTCTAACTATCTCTAACTCGTATCCAATTGAGAACCTTTACTTCTTTGAGAATCTTGTCGCCATCAATGGAAATCACTCGAACAACACATTTTCTCTAATTGTCAAAGACAATCCTAACCTTCAAGAGCTTTTCAACTGGGACAGGCCAGCCGGACGAAGAATTAACATAGAAAATGGCAGAATACTCATTCATTTCAATCCAAAACTCTGTCCTCAATACATCGAAGACCTCACAGAAGTTTCTAAAATATATAATATCACAAACTTTGTGGTTTCCAAAAATGCCAACGGTGTCAATTACTTGTGCAATGTCCTGAACTTGACTATAGATATTGAATTCAAAACTGCTTTGACTGTATGTCTTGTGATTCATCGACCAACTTTTACAAATAACACTTCACATGATTGGTTAAGGTTGATGGTATACTACAAAGAAGCTCCAGAAAAGAATGTTACGTCTCAGTTCAATAGTGATGCTTGCAATAATGATGGGTGGAAAATTAATGATGTAGATAACTTAGCTGTAGGATCTGAAACAGTCACACATTTACTCGCTCATCTAAAACCTGATACTCAGTATGCCTATTTCATTAAAATATACAGTCCTAACAACAGTGGAGGCCATAGTAAAATTTATTATGTACGGACATTGCCCTTCCGTCCTTCAATACCTCTTGACATCCAGGCTTATAGCAATTCCAGTGAAGAAATAATGGTCCGGTGGAAGCCCCCTGCGGAACCCAATGGAAAACTAAAAACTTATATAATCAGGGGGTACCTGTTGAATTATGAGGAAGAAATCAGCCTCAATCCGAATATGTGCCTTCATCCTTTACCATACATGGATGATTCTATGATCCAGCCAAACTTCCAGCAGCAATCGGTAGAAAGAACAGATATCATCAGCAGGAAACACCCAAATGATTTTGAATATCTCTGTGGCCGCAATTCTTTGATGAAACCATCAAAATATCTTTCTAGATTTGCTTACAATGGCACTATTCAAACCTGCGAAGATGCTGTATTCAATGTGATTGAAACGGAACTGATGAAATTACCGAATCCGTACAACACTCCTCAATCATCAAAATATTACCCTTTGCTCTCCGAGGAAGATACATCTGTGGACGAATCCGTGGAGTCGTACAATTATGATGGATCTTTCAGGTATTTCATCAAACGGTTCAATCAAAGTACTCGCATTGCCACACTGACCGGCTTGAAGCACTTCAGCACGTATGTTATTGAAGTCATGGCTTGTAGAATGAGCATCACTGAAGATTCTGACTTTGACGATGATAAATTTATCCCTCTCAAAGATGGAATTAACACTCAATGCAGTCACTCAGTAATGTTCACACTGAGAACTAAAAAGTCTAAAATGGCGGATGTTTTAAATGCTGACCATGTTGGTGTGAGAGTTATGAATAAGAGTGTTGAAATTGCCTGGAAGAAACCGAGTAGATTCAATGGAGCCATTGTTAGTTATAATCTTCAGTTGAAACGTAGTGATCACGAAAACGCAAAAGCTCAAGAAGAGTGCATATCCGATGGTTTAAATGAAGAGGAACTAAAATATGTCATAAAGAGCCTCGACTTTGGTCTCTACATGGTACGACTTCGAGCTAATTCTCTTGCAGGGGAAGGTCCTTGGTCTCCGTGGGTTCAGTTTGGCATCATTGATGAATCTAATAGTTTGTTCAAAGCAATCTCTGCTATTTTAATTTCAGTCACCTTTGCCTTGATCATATGCGCTTTGGTTGGGGTTAAAATTCTGAGATACTTTATGAAGAAGAAGAACAGTGGGTCAAAGAAGTTAATCAAAACTGTGAATCCTGAATACAGCAGCTTGGCTACAGGCTATGTTGAGGATGAATGGGAAGTATCCCGAGATGACATACAATTAGTGAAAGAAATAAAACGAGGAAATTTTGGAATGGTTTCTGAAGGAATGCTTCTGCCAGAAAATCGTCAAGTTGCAGTAAAGACAATGATAGAGTCTGCTTCAGTCCGTGATCAAACAGAATTTTTGAAAGAAGCAACCATTATGAAATCTTTTAGCAAAGCTCACCATGTCGTCAAGTTAATAGGCATTGTGTCTCGTGGCCATCCGATTTTGGTTATTATGGAACTTATGGCTTTTGGAGATTTAAAGTCTTTTCTCCGACAAACTAGGACATGGCCTCCTCCACCAAGTGCGGCCCATCTTCATCTGATGGCAGGTCAAATAGCTGATGGTATGGTGTACTTAGAAGCATACAAATTTGTCCATCGTGATCTGGCAGCTAGAAACTGTATGGTTTCTCAAGATTTGACTGTGAAAATCGGGGATTTCGGCATGACTCGAGATGTCTATGAGACTGACTACTATAGAAAAGGAGATAAAGGATTGCTTCCAATCCGGTGGATGGCTCCCGAAAGTCTTAATGATGGAGTGTTTAGCACAGCAAGTGATATTTGGAGCTATGGAGTAGTTCTCTGGGAGATGGTTACTTTGGCTGCTCAACCATACCAAGGCTTATCAAACCAACAGGTGCTATCTTATGTGGTGTCTGGTAACAAATTAGAGACACCTCCCTACTGCCCACCGCTGCTGCGTGAGCTGATGTCGGCCTGTTGGAAATGGAAGCCGAGAGCAAGGCCGTCATTTGCTGAAATCCTCAATCGCCTGGAGCCTCATCTCCCCGACGAGTTTAGAGCCGTTTCATACTACCACAGTCCTGAAGGCGTAGCAGCACGCACCGACCCCATACCTGAACCAACCAATCCTATCTCAGAAGATCTCTCTGAACACTCCCTTCTTCTTGTTCCTGCATCATCAACTGTTGAATTTACCTCTCGTTCCAATGGACCATTGTAA

>INR1_Mesovelia_furcata

GAAGTTATTGAAGGATTTTTACAAATCGTCCTCATTGACAATGCCGAGGCTCGTGACTTTGAGAACATATCTTTCCCGGAATTACGGGAAATCACTGGATACTTGCTACTGTACAGAGTCCAGGGCCTGCGTTCCCTGGGCAATCTGTTTCCAAACCTTACTGTCATCAGAGGCAACACTCTTATGCTCGATTATGCCTTCATGATCTTCGAGATGCTCCAACTCCAGGAAATTGGTTTATCATCCCTGACTGATATTATAAGAGGATCTGTTTATTTCACCAAAAACCCCATGCTATGTTTTGTTGATACCATTGATTGGGATACCATAGGCCCAAGTGGAAAGGGCGACCATCATATCATTGGTAATAAACCCCAAGAAGATTGTCCTGTTTGTCCTAACAGCTTAACACAACCTTGCCCTAAACATGCTATGACTGGACACTCACTATGTTGGAATATGCATCATTGTCAAAAATCTTGTCAACCGGAATGCATTGGTAACTGTGATGAGAATGGTGCATGCTGCCATAAAAACTGCCTAGGTGGATGCTATGGCCCTAATTCTGACCAATGTTTTGCTTGCCGACATGTTCTACATCGTAACCATTGTGTTGAAAAATGCCCAAATGATACTTTTATGTATTTAGGCAGAAGATGCATCAGTGATGAAGAATGTTATCGCATGCCAAAACCCATGGAAATTACAGATCATAATGTACGACCAAGACCCTACAAGCCTTTTAAAAATTTGTGTGTCATAGATTGTCCATCAGGATTTCAAGAGAAAGAATACAACATCAGTGGAACAGTAAAATATGTCTGTTCTGATTGTCATGGTGTCTGTAAAAAAGAATGTAATGGTGCAAATATCGATAGCATTGCTAGTGCTCAAGAACTTAGGGGTTGTGCTATTATAAAAGGAGCTTTAGAAATACAAATTAGAGCTGGTAATGTCATTAAAGAGTTGGAGGATAATTTAAATTCTGTTGAAGAAATTGAGGATTATTTAAAAATTGTTCGATCTTATCCTCTATTATCTTTGAATTTCCTACCTAATTTAAAAGTTATCCATGGTAAAAAGCTTGAAAGTGGCAAGTATTCATTAGTAGTACTTGATAATCAGAATCTTGCTGAATTGTGGGACTGGAAGAACCGGACAGATTTACATGTTGGAGGTGGTAGACTTTTCTTTCATTTTAATCCTAAGCTGTGTTACAACAAAATTGATGATCTTCGTAAAAAGCTGGGATTGCCCGAACACAATGATCTTGAAATTGCTAGGAATTCTAATGGCGATAAAACTGCATGTATTGTTGAGAAATTAAATGTGTCTGTTTATTCAAAAACATCGAAAGCTGCTCTTATCAAGTGGAAACAGTTTGAAAATCACGATTCAAGAACTCTTCTTGGCTATGTTGTGTACTTAATTGAAGCCCCTTTTAAGAATATTACCATCTATGACGGAAGGGATGCCTGTGGCGGCGATGGGTGGAGAGTCGATGATGTATCCTTAGGAGATTATGAAAAGCCAACTGAAATAAATCATGTACTTACTATGTTAAAACCATACACTCAATATGCTTTGTATGTGAAGACCTATACAATTGCAAGTGAAACCCGTGGTGCTCAGAGTCCCATTGAATACTTCCGAACCAATCCTTTCACTCCATCAGAACCTCAGAGTATATCGATATATTCAAACTCCAGTTCTCAATTAGTTCTTCATTGGCAACCTCCATTGCAACCCAATGGTAACGTCACCCACTATGTTGTTGTTGCATTGTTGGAAAGGGACAGTGAAGAATATTCTTTAAAAAACAGAAACTACTGCTTGGAGCCTGTAAATTATGATTCACGATCTAGTCGAGTATATACAAAGAATACCATTGATACTCCAGTTGAGAAGCAGGATGTTGAAGAAAGTGACTCTTGTCCATGCAATCGCAAACAATTTGACAATAAACAGCAGCGGGAAAAGGAGATAAAGTTTGAAGATCATCTGCAGAATGTTATCTATGTTAAAAGGACAAAAAGGAGCTTATTCTCCTCTGCACAAAACACGTGGATGGATAAAAATGAAGAAACAGAAATCTTATCAAATGTTGAAGTTGATGGTATGTACGAGAAGTTTCGTGTTGTTGTTTATGGAACTTCGTATGTGATTAGGAAGCTTAAGCACTATTCTTTGTATAATGTCGCTGTCCGAGCTTGCCGAGAATTGGAAGAAGAAGAAGACGATCCCAGAGTTGACAATTGCAGTAAACAAAAAATTACTGCTGATCGTACATTACCTTTAGAGCATGCAGATGACATAGATTCTCAATCACTTGCTTGGGAAGTGTCAAACGTCACTGTAGGAATGGTTAAATTAAAATGGGAGGAGCCACCAAATCCAAATAGCGC

>INR1-like_Hydrometra_cumata

ATACCCGGTAATTTTATTTGTAAAAAATGTGAAAACGGAAAGTGTGGTCGTGAATGTAAACCAAGAAAAATTATATCCAGTAAGGAAGATATAGAGAGTTTGAAGGGTTGCAAGTATATTAAAGGGCCTCTAGTTATCAATCTCTCTGGTGATTATATGATTGAGCTTGAGCATAATTTAAATGAGATTGAGGTCATTAGGGATTACCTTGCTGTTAAAGATTCATATCATATTACATCATTTAACTTCCTGAAAAATTTGAAAGAAATTAGAGGTGAACATAGGGATAATAATTATACATTAGAAGTGTTTGCAAACAAAAACTTGGAATTTTTGTGGGATTGGGAGCACAAGAATGGAAAGTTAAAAATAAATGGACCTGTATATTTTCATTCAAATCCTAAACTTTGCCTCTCAAATATCAAGGAGCTCATCACTTTGTCAGAAAAAATTAATGTAAATGAATCATCGGTCTCAAATCTTACAAATGGTGTTCAAGCTGTTTGCTTGATCACTGAATTAAAAGTCAATATAACACACATAGAGGACAAGAGAGTAATTCTTCAGTGGAGTCCATTCAATTATCATGATCCAAGAATTCTTTTGGGTTATAAGGTGCACTACAAAGAAGCTATTCATAATGTTACTATTTATGATGAGAGATCAGTTTGTGGCAAAGATGATTGGGTTGTAGTTGATATTAAAGGTAACCAGAATAATGCTACTCTAGAAAATTTTCGTCCCTTTACTCGGTATGCCATATTTGTCAGAACTAGTACTATTTTAGCTGGCAAAGATGAGCAAAGCCAGCTTCAATATTTTATGACTGAACCTTCAACTCCGTCAATTCCTCGTAACGTAAAATTATTTCCAACTAATCATTCCTCCTTGTATGTCTCCTGGGAGTCACCTGCCCATCCTAATGGCAACATTTCTAACTATCTTGTATCAGTGTATTTGGAGAGTTTCTTATTTGATAACCATGGCTGTTCTCAAGAAAATGAGGTGAATTTAAATACTGCCAAGCTAAATAATATTTTTATAACTGAGGAGTCCAAGGTTGAAGAAAAATATCATATTTATATTCCAGGGAAGAGCTCAAAGATGAATGCCCCGGAGAAAAGAAACTATGAAGTTAGTAAAATAATTGCAAATTCGTTTGAAAATAAGCTCATGAATTGTGTTTTTATCAAAACTACTAAAAAGAAACGTAGCATTAGCAATATTCCTGATTCTTCTACTTTGGTCGCACAAAAAACTGTAAAACATACAAATGCAAGTTTTGATAAACTGAGACACTTTACCCCCTATAGGGTAGTTGTTCAGGCCTGCCAAGAAGAACGATGTGGCAATAAAACTTCACGAATACAATTCACTTTGCCTCATCCTGATGCTGATAAGTTGAATGATTCATATCCTTTTATTACTATGTCAAATAGAACAGGAACTGTTATTCTCTCTTGGGAAAAACCCTACTTAGTGAATGGCTTTGTCAAAGCCTATATTATTGAATACAAAACTGTTGACAGCCATAGTGCCACTCCAATTTGTTTCACTGAGAAAGAATTTGCAAAAACTAAGGCATTTACACTAAGCAGTTTGAGTCCTGGAAACTACAGTATCCGCTTGAGGATTCTTTCTTCACAAGGGCTGGGCGAATTCACTTCCTGGAAATTCATATATATACCAGAAGAACCTCTTCTTTACCAATATATTTACTACATAATTGCAGCTGTAGTTAGTATTTGCATTTTTTTGCCACTAATCTGTGCAGGCATGTGGTTTTATATTTACAAGACTGGAAAAGATAAGCTTATAATATCTATTAATCCAGAATATGTCACAAGCTCTTATATACCAGACAACTGGGAAATCCCTAGATCTCAAGTTGAACTTATAAAAGAAATAGGTCAGGGATCTTTTGGTATGGTTTATGAAGGGACATTGAACTTGGATGGAAGTTCACCTGTACGCTGTGCTGTTAAAACTGTCAACACCCAAGCTTCTGAAAGAGAGAAAATGGAATTTCTAAATGAAGCTTCAGTAATGAAGGCTTTTGACACTTATCATGTTGTGAAGTTGTTGGGAGTAGTTTCCCAAGGTGAACCTGCATTTGTTGTTATGGAACTGATGACAACTGGGGATCTAAAATCATTTTTACGCTCGTGTCGTCCAGAAGTGGCAGAACCACATGTGCGGCCTCCAACTTTACGTCAGTTGTTACAGATGTCAGCAGAAATAGCTGATGGCATGGCGTATTTATCTGCCAAGAAGTATGTCCATAGAGATCTAGCTGCTCGTAACTGTATGGTGTCAGAAAATCTGACTGTGAAAATTGGTGATTTTGGCATGGCCAGAGATGTCTATGAAGGAGATTATTATAGGAAGGGTAGTAAGGGGCTGATGCCTGTCAGATGGATGGCACCAGAAAGTCTCCGTGAGGGAATTTTCTTATCCCTGTCAGATGTTTGGAGCTTTGGGGTTGTACTGTATGAGATGGTAACTCTTGCCGCTCAGCCTTACCAAGGACTCTCTAATGACCAAGTTCTTAGATACATTCTTGATGGTGGAATTATTGAACGCCCAGAGGACTGTCCTGATAAAGTGTATATCCTGATGAGGAAATGTTGGCGACAAAAGCCCCTAAACCGACCTTCGTTTTCGGACATTGTTTCCTATCTTCATGAGGATGTTTCTTTTGCATTTCGAAATGTCTCATTTTATGATAGTGATGAAGGTAAAGAAATACGTAGGAATTTACAAGAATCTTGCGAAACTCGACAAGATTGA

>INR2_Hydrometra_cumata

TCAAGAGGAGGTTTCTTTTTTCATTTTAATCCTAAGCTTTGCCTTCAGGTTATAAAAAAATTGGGGGTCACAGTTAATTATACCAAATGGACTGAGGTCGAAGTAAGTACCAACACAAATGGGGTTGAGTCTGCTTGTAACATTATGAATACAACGGTTGAGGTTAGTTTTCGGGATGTAAATAATTTCACTCTACGGATTGAGCGCCCAAAAGATGGTCAAATTTGGGATAGATTCATGGTCTTTTATAAGGAAGCTCCGTTTAAAAACATTACCTCACACTATGAGGATGGATGTAGTAAGAATGTTTGGAAATTGAACGATTTTGTGGAAGATGAAAGAGATGGGTTTTTCAATGTGACTATTACCTCACTTCGACCATTCACTCAATATGCATATTTTGTGAGAACATATTTTGTTAAAGGAGATGTGGGACATACTCCTATTCGTTATATCAGAACAATGCCATCAAGGCCATCCTCCCCAGTCAACATAGTTGGTTATAGTAACTCTAGTGATGAGATTGTTCTCTCGTGGAGTCGACCTATTCACCAAAATGGAAATTTAACTCTGTACATAATTATGGGTACTTTACTGAAGGAAAAAGGTATATCTGTTTACAAAGAAAGTGATGCTTGTTTACACAGGAAATCTCTTGATACAAATATCCCAGCACATATTATAGAAGCTGTTGAAAAAGGAAGAATGCTAGTTGATAATACAAATCAAGTGTTTGAAGATGATCCAGTTTGTGATTCTTCTTCTGGCAGTCATCTTTTGTCTCAACCATCTCCTTTTTACGTTGATGATTCATCGCCAAGTTTGGACAAGTGTATGACCCAGTATTTGAATGAGGCAATGATTAAACCAAGGCTATCATTTCTTCTTTTGCCTGACCAGGTAACTGAAAAACCTGTGGTACCTGATACTAGTATAACCATGCCCAATCTTTCAGAGGGTATTCAAGAGAGTGGTGAGATAGATGAAAGCTTAGGTGAATCTGATGAAAGCTTTGAAAAGTATGTATCTTATGGCATTAATAGTGTATCTATCAATGGGCTAAAACATTACAGCACTTATTTGATAAAAATGAAGGCTTGTAGAAGAATGGAACCAGGAGAAAGTTTGCACAGGTTTTATTCATCTGACTGCAGTTTTCCATACATACTAACATTGAGAACACAGAAAGTGTCAAGTGTCAATTATGATGTAATAAATGCTGAAAGTATAATTGCTGAAACTGTGAATAGAAGTGCATTCATCTCTTGGAAAGCTCCTGAAAACCTGAATGGCATTGTTGTAGGATACACCATTGAAATCAACAATACAAACAAACAAACCTCTCGTACACCTTGTATTTCTGCTGAAAATTTTACAATTTATCATGGCAAGAATATCGAGGTCAATGACTATGGCACTTACACAATCAGGATTAAAACTTTTACTACAGCTGGAGAAGGTCAGTTTTCAAACAAATATGTGTTTTCTGTTGAAGAAGATTCATATGCTTTTGTAACCGCGCTTGTTACAGTTCTCGTTTCTGTTGTTATAATTCTAGCTTTATTGGGTGTTATTATAATCAGGATGTTGAAATATTTTTATAAAAAGAGGTTTCAGGAGAAAAATGTTCTGTTCAAAACCATTAATCCTGAGTATGTTAGTGTATCTGGTGGATATCTTGAAGATGATTGGGAAATTTCTAGGGAAGACGTGGAGCTTGTGAGAGAAATAAAGCAGGGCAACTTTGGAGTGGTTTATGAAGGATTGTTGCTTCCTGAAAATAAACGAGTAGCTGTGAAGACATTGATAGGAGGAGGTGGAGATCGAGAAGGAGAGTTTCTCAAAGAGGCCTCTGTGATGAAGTCTTTTAGTGAAGCGCACCATGTGGTGAAGCTACTTGGTGTTGTGTCTCGAGGTCAGCCAGCTATGGTTGTCATGGAGCTGATGGCGCTTGGTGATCTCAAGTCCTTCCTGAGGATGACTCAGGACTCTGCCCAGTACCCTCCACCTACTCCTGCTAGGTTTCATCACATGGCCGGTCAGATTGCTGATGGCCTAGTCTATCTAGAAGCTTACAAATTTGTACACAGGGATCTTGCTGCAAGAAACTGCATGGTTTCAGAAGACTTGACAGTTAAGATTGGTGACTTCGGCATGACGAGGGATATATATGAGACAGACTACTATAGGAAGGGTAACAAAGGTTTGCTGCCCATCAGATGGATGGCACCAGAGAGCCTCAATGATGGAGTGTTTA

>INR1_Hydrometra_cumata

ATGACAGGGCGTACTCCTAAGAATCACTCTCATGGAGATAATTTCAAACCGAGGGAAAGTTTTTCAATGCTCCAGTGGTCTAGTCGAGGAGAAGACAAATGGAGAACGTACTTTTTTATTTTTGGACTTTCCCTTTTGTTGCAAGTGTCCCTATTATCGACTTCTTCAGTTCCCTTCAAGACATCAAGTGAACATGTCATGAAGAGAGAATCAGGAGTATGCCAGAGTATGGATATCAGGAATTCCGTGGCTATGTTTTCAGAACTTACAGGGTGCAAAGTTGTTGAAGGATTTGTTCGAATTGTCCTGATTGATAAAGCTGACCCTATGGATTATACCAATATATCATTTCCGGAGTTGCGTGAGATATCTAGTTATCTTCTCATGTATAGAGTCCAAGGGTTGAGATCGATCGGGAAATTATTCCCGAATCTGGTTGTCATCAGAGGAAATACTTTGTTTCGTGACTATGCCTTGGTAGCCTATGAAATGCTTCAGTTACAGGAAGTGGGTCTGTCATCCTTAATGAGTATCACCAGGGGAGCTGTCTATTTTGCCAAAAATCCAATGTTATGCTTTGTTGAGACTATTGAGTGGGATGTTATAGCACCTAATGGAAAAGAAGAACACCATATCAAAGGAAATAAGCTTCAGGTTGAATGTCCACACTGTCCATCCAGCATTAATGGAACTCAACCATGCCCGAAACATTCAACTTCTGGAAGTTTACTTTGTTGGAACATGCATCACTGCCAGAGGTTTTGTCCTGAAAACTGTGCTGGATTTTGTGATAATGAAGGGGAGTGCTGTCATCCCAGCTGTCTTGGGGGCTGCACTGGTCCTGGGCCCTCATACTGTTTTGCCTGTAAAAATGTTTCGTTTCAAAATTTGACCACTGGTTTGATTGATTGTTATGACAAATGCCCACCTGGAACGTATGAGTACTTGAATCGCCGCTGCATTTATAGGTCAGAATGCTATAACATGCCTCGGCCTCGAGAAGTAATTGGTGTAAGAGATTATCCCTGGAAACCATTTAATGGAAAATGCATTCTGGAATGTCCATCTGGTTATTTGGAAGAGGATGACATCACTACCCCAGGTCATCCTGGATATGTATGTCGCCGGTGTAAAGGTATTTGTAAAAAGGAATGCCCTGGAGCTAGTGTTGACAGTACCGACACCCTACAAAAATTGAGAGGCTGCACTTCAATTAAAGGATTCTTAGAGATTCAAATCAGAGGAGCCAACATGGTAAAAGAACTGGAGGATAGTATGCAAATGATTGAGGAAATTGACGACTACTTGAAAATCGTTCGATCTTTTCCTGTTATCTCACTGAACTTTTTACCTAAATTGAGAGAAATTCATGGATACAGATTAGAAAGCTCCAAGTATGCCCTGGTGGTCCTGGATAATCAGAATTTGGCGGAGCTTTGGGATTGGAAACACGGAGAATCTGGTTTGAAAATAGAAAATGGCCGTCTGTTTTTTCATTTTAATCCAAAATTGTGTTTTGATAAAATTGAAGATTTCAGAAAAAAAGTAGGACTTCCTTCTCACACTGATTTAGAAATAGCCAGGAATTCAAACGGAGATAAAACTGCTTGTGTTGTTGAGCAGTTATTTGTGAAAGTTGATACGAAGTTACCCACAATTGTTTTGATTAAATGGAAACATTTTGAACACCACGATCCCAGAACTCTTCTGGGATATGTTGTTTATTCTATTGAAGCTCCTTACAAAAATGTTTCTATATTTGATGGAAGAGATGCCTGCGGTGGAGACGGATGGAGAGTTGATGATGTTTCCCTCCCTGATGAAACTGAATCAAGCAGCTATGGCAAAAATTCTAGTGTTGAAGTTAGTTACATGTTACCTCACTTGAAGCCATTTACCCAGTATGCATTTTACATAACTACCTATACCATTGCCAGTGAAAGGAAAGGCGCACAAAGTGACATACAGTACTTCCGCACTAATCCTTCTATTCCTTCAGAGCCTCNNNNNNNNNNNNNNNNNNNNNNNNNNNNNNNNNNNNNNNNNNNNNNNNNNNNNNNNNNNATCACTCATTACATTGTTGAAGCATTTTTGGTCTCTGATGAAGATGAAAGCTTGTCTGAGTATCGTAACTTTTGCAACGATCCTCTAAGCTCTCTCACAGATTCAAGACGATTAATTCCTAGTAAGAACATGGAGCCAATCATGGAACCTTCTTCAATAGATAGTCAAGAGAAGAATTCTTGCTCTTGCAACAAAAAGACTTTGGATTCAAAACAAGAACGTGAGAAAGAGATTAATTTTGAAGATCAGCTGCAAAATTTAATTTTTGTGAAAAGACTGTCTTCAAATGGAACTAGAAAGAAAAGAAGTTTTTCTTCCTCTGATGATCTGAATGGAAATCCTTATATTTCTGAAACTCTTGAGCAAGGGCAACTAATTAATGGCGTTTACAAAACTTTTGCCAAAGTAGTGAATGGAACTTCATATGTTATAAAAAACTTGAAGCATTACTCTCTGTACAATGTTGCTGTTCGGGCTTGTAGAGAGTTAGAGGAAGATGAAGCGAAAAACTCCACTATAAATTGTAGTGCTAAAGCATTGATGTCTGATAGAACATTACCTCTAGTGAATGCTGATGATATTGATAGTCGATCTCTAAATTGGGAAGTATCAAATACTACTCAGGGTATGGTCAAGCTGAAATGGGATGAGCCCGTGAACCCAAATAGCTTGATTCTAAATTATCAAGTTGAATATTTCAGAACGGACATTGAGAATTCTTATCCTTTTATCCAATGCATCCCCAGGAAGTTCTTTAAAGATATGGGAAATTCCTATACACTGACAAGTTTAAGTCCTGGTAATTACAGTCTCCGGGTACGAGCAACTTCTCTGTCTGGGCATGGCGAGTACACCATCATTAATTTCTATATCAAGGATGAATCCATATCTGTATTTGTGCAAACCATAATAATTCTTCTGATCATTCTGGTGTTCACATTTTTAATAGTATTTTCAATCATGTATATTCGTAAGCAGATTGGGAGCATTCCTAACATGAAGTTAATTGCTGAAGTTAATCCAGAGTATGTTCCATCAGTGTATATGCCTGATGACTGGGAAGTTCCTCGTGACCAAATAGAATTGATCAGAGAAATAGGACAAGGGTCTTTTGGAATGGTTTATGAGGGTGTCTTGAGAAGTTCAGGGACAAGCCCTAAACGATGTGCTGTTAAAACTGTGAACAACAATGCTACTGAAAGAGAAAGGGTTGAATTTCTCAACGAAGCCTCAGTTATGAAAGCTTTCAATACCCATCATGTGGTGCGCCTTTTGGGTGTTGTCTCTCAAGCTCAACCAACTTTAGTTATTATGGAGTTGATGGCCAATGGTGACTTGAAGGCCTACCTAAGATCATGTCGACCAGATCCTTCTTCAAATCCTCAGGCAAGACCACCAACTTTGAAGGCAACTCTGCGTATGGCTGTGGAAATTGCAGATGGTATGGCTTATTTAGCTGCCAAGAAGTTTGTACACCGTGACCTTGCTGCTAGAAACTGCATGGTGACTGATGAGCTAACAGTTAAAATTGGTGACTTTGGAATGACCCGTGATATTTATGAAACAGAGTATTATCGCAAAGGTTCCAAAGGACTTCTACCTGTGCGATGGATGGCTCCAGAAAGTCTTAAAGATGGCGTTTTTTCAAGTCGTTCAGATGTCTGGAGCTATGGAATTGTGTTGTATGAAATGGCTACGCTTGCTTCCCAACCCTATCAGGGACTCTCTAACGATCAAGTTCTGAGATATGTGATTGATGGAGGGGTCATGGATAAGCCTGAAAATTGCCCAGATACATTATATCGTGTCATGGGGATGTGTTGGCAGCATAAACCAAAGCAGAGGCCTACATTCATGGATTTGGTTGGCATGCTACACGAAAATTCAGATCCAAACTTTTCAATTGTTTCTTTTTATGATAGTGAGGAAGGTCAAGATATAAGAGGCATTGGTGAGACGGAAGACACCCCTCTTGGTATTTCCAGGGATATTGAAGATTTTTCGTTGAGTGATGATGACATGAGTAATGATGTAGTTAAAGATGGTCCAAATCAAATTGATGATGATGATGAAGAAGAAGAAAATGCAGATGACTATGGTGATATGGGTATAAAGAATATTAATGATTCAAACTCTTATCGGAGCACTGGTGTTGATATAGATGTTGATTTTGTCACCCCTCGTCCTAGCACCAGTTCAAACCAAAGTAATGGGAGGATCACACCAAATGGTATTATTAGACAGACTGAGGTTAAAACCACTAAGTGTTAA

>INR1-like_Hebrus_sp

TGTCCAGAACATTGTTCTAGTTGTAACGACCATGGTTGCTGTCATTCAAATTGTTTGGGGGGCTGTTTTGGACCTGACGAAAACCAATGTTTTGCATGTAGAAATTACTTGTCAAACGGTAAGTGTCAAAACATATGTCCTTCTGAAACATTTAAGTATTTAGAAAGGCGGTGTATTTCTGAATCAGAGTGTCATAATATCGATAAACCGCTGTATCAGTTCTATCAAAGTTGGAAGCCTTTGAACGACACATGTACATTAGACTGTCCGTTGGGATACCACGAACAAGAAACATTAGTCAACAGTCGAAAGCGACTAGAGTGTCTGAAATGTGAAAGAGGCAAATGCAGAAAAGAATGTCCATCTGCCATTGTGACAAGTGCTGAAGATGCTGAAAAATTGAGGGACTGCACTGTTATTAAGGGGGCTTTAATAATAAGAGTTTCAGGAAGTACTAATATAATTGAGCAGTTGGAGCTTAATTTAGGAGGCATAGAAGAAATAACCGATTACCTTAAAGTTATAGACTCGTTTCAATTGACTTCTCTTAACTTCCTTAAGAACTTGAAAAAGATCAATGGTGTAAAACGTGAACGTTCTAAATATAGTCTGGTTGTTATTGAAAATAAAAATTTAGAACTTCTTTGGGATTGGGAACACAGGGAAAGTGAACTCAGGATTAATGGTTCTTTATTATTTCACTTGAATCCAAAATTGTGTTTGACTAAAATCAAAGAATTGATTAAACAAACTGGTGTTACCGACCTGTCAAATTCTTTTCTTCCTGATCTCACTAATGGAGTGAAAATGGTTTGTAATGTTACTAAATTAGACGTCAAGGTGTATAACATCAATTCAACATCTGTGGTTATTGTCTGGGATCAGTTTGTTATTTATGATGGAAGGAAGTTATTGGGATACAGCCTTTACTTGAAAGAAGCTCCGTTCAAAAACATATCAATCTACGACAGGAACGCTTGCGGAGGTGGTGGATGGAACGTCAACAACGTCTCTCCCTCTGAAGGTAAATTGGTCAACTACACTGTTAACCACTTGAAACCATACACCCAGTACGCTTTTTATGTCAAAACATTCACTGTCAGAATTGACAGAGAGGCCCAGAGTGAGATCAACTATTTCAGAACTTTTCCCGGTTATCCGTTCAATGTTAGGAGTTTAAAAGCTGAATCCAATTCGTCGAGTCAACTGTCCGTCTCTTGGAAACCTTCTGCCAAACTGAACGGCAACGTAACCCATTACAGGATATCTGTATTTGTTGTCGAAGACTCGGATCTGACCGAGGACAGTTGCGCCGACAGGAAGAAATTTTCATTCAACAACACTTTAAAAAGTTCAACGGATGTCTCAACGGAGGAGCACAACTTCTATTTGTACTCGCATGGTATGGATCACTTGGATGTATTGACAGACGAAGAGAGAGAAAGGACCAGGCAGAGAGCTATAGATTTTGAAGATCAAATCCACAACCAAATTTATATTAAAAGAGGTAAAGAGAGGAAAAGAAGGTCTGTTGATGACAACAACAACAGCCGACCAGTATTTGAGGAATTAGAAAGGGAAAAGGAAATAATATTGAACAAAAGAGGAGGTGATCTCCTCTTTACAGATGTTGTGATGAAAACATCTTATCATATTGACAATTTAAAACATTTTACTAAATATAAAATAGTAGTTGAAGCGTGTAGAGAATGGGAACCCGGTGAGGACACAAGTTTATTGACTAATTGCAGTTCAGGGGTTGAAGTGTTTGAAAGGACCCTGCCGCTGGCGGGTGCCGACGTCCTCTCATCCGATTTCCCTCTTGTCTTTGTCTCAAACAAAACACTCGGCAGCGTCGCCTTCAAATGGAGAGATCCTTTGGACACCAACGGTTTCATCAAGTCTTACATTATTGAGTACAAGAAAGTTGAACTCTCAACTTACATAGTCAATTGCATAAGCTACAAACAGTATTTGTTGCACAACAGTTTCACCGTACAGAATCTGAGCCCGGGTAATTACAGCGTGCGTTTGAAGGTGGTTACGGTGAAAGGGGAAGGCCCTTACACCCCCCTTAAATATTTCTTCATTCAAAGCCAATCCCTGTTCACCCTTTACATCTACTGGGTGATCGGGGCGTTGGTCTGCGTTTCCCCTCCTGTTTTGTTATTCGCGTGGTACTACATCCGCAGCGCCAGTCAAAAGAGTAAACTAATTATAACTGTCAACCCAGATTATGCCACTAGCTCTTATATAGCCGACAGTTGGGAGATACCGAGATCGCAAATAGAACTCCTGAACGAGATAGGGCAGGGTTCGTTTGGCATGGTCTACGAAGGGACGCTGAACCGAGACGGGGAGGCGTGGAAAAGATGCGCCATTAAAACGGTCAACACCCAGGCGTCGGAGCGTGAGAAGATGGAGTTCCTGAATGAAGCGTCCGTTATGAAAGCGTTTGACACTTACCACGTGGTCAAGTTGCTGGGCGTTGTATCAAAGGGGGAACCTCCTTTTGTCGTTATGGAATTGATGACCAACGGAGATCTGAAGACATTCTTGAGGTCTTGCCGACCCGACTCCGGGGACCCCCACGTGCGCCCTCCCCCCTTCAACAGGCTGTTGCAGATGTCGGCGGAAATAGCCGACGGCATGGCCTACCTGTCGGCCAAAAAATACGTGCACAGGGACCTGGCCGCTCGCAACTGCATGGTGGCCGAAGATTTGACTGTGAAGATCGGTGACTTTGGAATGGCGAGGGACATCTACGAAGGGGACTACTACAGGAAGGGGTCAAAGGGGCTGATGCCCGTGAGGTGGATGGCCCCAGAGAGCCTGAGAGACGGCATATTCCTCACCCTGTCGGACGTTTGGAGTTATGGTATCGTCCTGTACGAGATAGTGACCCTGGCGTGCCAGCCGTACCAGGGCCTGACCAACGACCAGGTCCTGCGCTACATAACAGAAGGAGGGATCATAGAACGGCCCGAGGGTTGCCCCGACCGCCTCTACGCCCTAATGAGGAAGTGCTGGCGACAGAAGCCATCCAATCGACCCTCCTTTTTACAATTGGTCGCTCACCTCCACCAGGACGTCAGCCTCAATTTCACATCTGTTTCATTTTATGACAGCCCAGAGGGAAAAGAACTGAGAAAGTCTTTAGACACCAACCAACTCCTTTAA

>INR2_Hebrus_sp

ATGAGGCTGGAGGCGTGGTGGGTGTCGATCCTGTGGTTGGCCTCCCTTGGCCGCTCCAGCCCTCCGGGGATCTGCCCCAGCATGGACATCAGGAACTCCGCCTCCGAGCTACGCCTACTTGAACCATGTCGCGTCATTGAAGGATTCCTGCAGGTGGTGTTAATAGACAACGCCCAGGAATCCGACTTCGAGGGCCTGGTGTTCCCCCGGCTGAGGGAGGTGACCGGCTTCGTCATCTTCTACAACGTGGTCGGGTTGAGGTCCCTTGGCCAGCTCTTCCCCAACCTGAGCGTCATCAGGGGCGACACCCTCTTCCTCAACTTCGCCCTCTCCGTCTTCAACCTCCCCGACCTTCAGGAGATCTCTTTGGTCTCCCTGACAACCATTATCAGAGGATCAGTGGCCATTGTTAATAATCCGAAGCTCTGTTTCGTGAAAACAGTTAACTGGGACCTGATTGGCAAAGGAGACCACTACTTCAGCCTGAGGGTCCACAGCTGCCCGTCTTGCTCAAAGGAATGTGACAACTGTTGGAACAGAACTCACTGCCAGATAACTGATAATAGCTCATTCCAATGTCATCAAGAGTGTGTCGGAGGCTGTACAGGTCCTGGACCCAACCAGTGCATTGCTTGCAGACACTTCCTTGTGAATGGAACTTGCACTAAGCATTGTCAACCAAATGAGTATAGTTACTTGAACCGACACTGTGTCTCTAAAGAATCATGCCTGGAAATGCACAAAACACTCTCGAGACCAGGGAAGGACACACCCCCTTCTGCTTTGAAGGACATGATGTGGTTCACGTGGAACGGGAACTGCGTTCAGTCGTGCCCGCAGGGGCTCGAGAGAGACCGCGACAAGGGCTGTCGCAAGTGCAAAGGGACCTGCAGGAAGGTTTGCCTGGGCATTAACGTGGACAGCATTCAAGCAGCTCAAACCCTTGAATACTGCACCCACATTAACGGATCGCTGGCGATTCAAATAAAGACCGGAGATCCGAGTGTCATTGCTGAAGAGCTGAGGAAGGGCCTTGGTTTGATTGAAGTCATCGAAGGTCAGCTTCGTGTAATACGTTCTTTTCCGCTCATAGACTTATCATTTCTGAAAAGTCTCAGAATTATTAAAGGGGATACCCGTTTTAGCGATGTGAAGGACAATTATTCTTTTGTCGTCCAAGATAATCAAAACTTACAAGAGCTTCTGGACTGGAACCGGCCAATTGGGAAAAATTTTAAGATCATCAACGGTAGGCTACTCGTTCATTTTAATCCAAAGTTATGCCCTCATTACATCGAGGAACTGGCCGAGATAGCCAAAATAAGTAACATCACTGACCTTGAAGTCGCCAGAAATTCAGTCGGAGTTAATTACTTATGCAATGTTCTTAATTTAACAGTTAATGTTGAATCAAAAACAGAAAATAGTTTCATTCTAGTTATTGAAAAACCAATTTTTTTGAACACTTCTCCAGAAAGTCAGGATTGGTTAAGGTTCAGCGTTCATTACAAAGAAGCTCCTCACAAAAATATTACTTCCCAATTTGATAACGATGCTTGCAGTGATGATGGTTGGAGAATCAGTGATGTCAATTCTCCGATCGAAAGAAGTTTTAACGGAAAACCAGAGAAAACTATTATCAATCACTTGATAGCTCGCCTAAAACCCTATACACAATACGCTTTTTTTGTTAAAATTTACAGCGTTGGTTATGCTGGCGGTCATAGTATGATCCAGTATGTAAGGACTCTACCGTCCAAGCCTTCGTATCCAGAAAATGTTCAAGCATTCAGCAAATCTTCTAGTGAGATCGTTGTAAGGTGGAGCCCTCCTGTCAACCCAAATGGAATTTTAAAACATTATGTCATCCACGGTGAGATGTTGGAGTACGATGAAAGCATTATTTTCAACAAGGATTTTTGTCACCTGTCCCTTCCCTATACTCCAGAAGATTCTACAATCCCTCCGAACTTCCTCACGCAGTCCGAATTTGAAAAGGAACCATGTTGCAGAAGTAACAAGAAGAGTTTACTTACCCCCATCTCTTGTGACCAGAATTCTATGGAGAGATCTCTCAAGTCATATTCATCTTTTTATTACGACGGGTCTGTGGATAGTTGTGAAAAATTGTTGTTTAAAAAAATAGAGAACTCTGTTACTCAGAACGAGAAAACCCTTAAACCAAAAGTGACCCTCTACCAATCAAAAGTTAAACGTTCTGATCTCAACGTGTCAAACAATTCTTATTATAATTCATTTCTTCATGAAAATGACACTGTTCTTGATAATTCTGAAGGATTCAACAAAGATGGTACATACAAGTTTTTTACTAAGTATATTCATCCCAGCATCAATTTATTCACGATTAATAGTTTAAAACATTTTAGTTCTTATTCAATTGAAATAAAGGCTTGTCGAGAAAAAGTTCCTGAAGAAACAGATAAAAATTTTGCTGAAAGTGAATGCAGTCACTCATCTGTATTTACATTAAAAACTAAGAAATCTTCTTCAGTTGATTTCATTGAAACCAAAAGTATTATAGTTCAAGTTACTAATAAAACTGCCAAGATATTTTGGAGGAAGCCCAATGAGTACAATGGTATTATTGTAAGTTATCATGTTGAGTTTCGTCGTATTAATTTAGAAAATGCGAAACCACAGAGCGATTGTATTTCTGGTAAGGAATTTGAAATCAGTAAAGGTCATTTTATCAAAGGTTTAGATTTTGGCCAATACACATTGAGAATAAGAGCTTTAACTCTTGCTGGAGAAGGCCCATTTTCTCCATTTATCAATTTCACGATAGAAGATAATTCTTACACATTTTTCAAAGCTATAATTATTATTGTTGTCTTGGTTTCTTTTATTGTGATTTCCCTTGCTGTTGTTGGAGCTAAAACTATAAGTTATTTTCATGAAAAGAGGCTAAAAGAAAAGAATATTTTATACAAAACAGTTAATCCTGAGTACAGTAGTGTGGCTACCGGATATGTTGAAGATGAATGGGAACTATCCAGGGATGATGTCCAACTAATCAGAGAAATCAAACAAGGCAACTTTGGCATGGTGTACGAAGGATTGCTATTGCCTGAAAACCGTAGAGTAGCAGTCAAAACTGTCATTGAAACGGCACCTGGCAGGGACCAAACAGAATTTTTGAAAGAGGCCACTGTCATGAAGTCTTTTAGCAATGCCCACCATGTTGTTAAACTGATCGGCATTGTCTCCAGGGGTCAGCCGATTTTAGTTATCATGGAACTAATGGCGTTGGGCGATTTGAAAACCTTCTTGAGGAATAGTCGCGACTCGCCTCACTATCCCCCACCTTCGGCATCTCAGCTGTCGCTGATGGCTGCTCAAATTGCCGACGGGATGGTCTACCTGGAAGCTTATAAGTTTGTCCACAGGGATCTAGCCGCGAGGAACTGTATGGTCTCCGAGGATTTGACCGTGAAGATCGGAGACTTTGGAATGACCCGCGACATCTACGAAACCGATTACTACAGGAAAGGGAACAAGGGGCTTTTGCCGATTCGTTGGATGGCGCCCGAGAGCTTGAACGATGGGATTTTCACCAGCCATTCGGATGTTTGGAGCTTCGGGGTCGTGCTTTGGGAAATGGTTACGCTTGCTTCTCAGCCGTACCAGGGCATGTCGAATGAACAAGTGTTGCAGTATGTTGTGTCCGGAAACAAACTGGACGCTCCGGTCTACTGCCCGAGTCATTTGAAGCACATAATGTTCAGCTGTTGGAAATGGAAGGCAAAACTTAGGCCTTCGTTCGTCAACATTCTCGATCAACTCGATCCGTATGTTGGCGAAGGATTTCGCATGGTATCTTTTTACCACAGCAGCGAAGGGATCAAGTCCAGAGACTTAATGTCAAACGGAGGAAATGACAATGAAGAGCAAACTTCACTTTTATTAGTCCCAAGTTCGTCCACTGTGGAATTCCACACTTCCAGAACTGTGAACTCATAA

>INR1_Hebrus_sp

GAAGTGAATGATTTTATAAATATTAAGCCAAACCCTTATAAAACTTTCAAAGATAAGTGTGTTCTTGATTGTCCATCTGGATATCAAGAAAAAGGAAGTATCTTTCAAGGCCGCAATATTACCGTTTGCCATGAGTGTAAAGGAATTTGTAAGAAAGAGTGTCCAGGAGCGAGTGTGGACAGTATTGCTTCAGTGCAAAAGTTGCGTGGTTGTACGTCTATTAAAGGAGCTTTAGAGATTCAGATCAGAGGAGCCAACATGGTAAAAGAACTTGAAGAAAATATGAATATGATTGAAGAAATAGATGATTATTTGAAAATTGTCAGGTCTTTTCCTCTTATTTCATTAAATTTTTTACCCAGGCTCAAAGAAATCCATGGAAGAAAGTTGGAGAGTAAAAAGTATGCTTTAATGGTTTTGGATAATCAAAACTTGGCAGAGCTTTGGGATTGGAAACATCGATCACCTGATTTAAAAATTAGAAATGGAAGACTATTTTTTCATTTTAATCCAAAACTTTGTTTGGATAAAATAGAGGAACTTCGTACTAGAGTTGGACTTTCAAATCACACAGATTTGGAAATTGCTCCTAATTCCAACGGCGATAAAACTGCTTGTAAAGTGGATGAATTAGCTGTTACTGTTTATAGGAAGTCATCAAAAGTTGCTTTGATTAAATGGAATCAGTTCAAAAATTATGATCCCAGGACTCTTCTGGGATATGTTGTATATTCTATTGAAGCACCCCATAAAAATGTAACAGTTTACGATGGTCGAGATGCCTGCGGGGGTGATGGATGGAGGGTGCATGATGTATCAGTTCGAGCTGATGTTCCCTTGATGTCACCAAGTGACTCTTCTAATGCCGAACTTGATTTTATACTGACTCGATTGAAGCCTTATACTCAGTATGCGTTTTATGTGAAAACTTATACAATTGCTAGTGAAACAAGAGGAGCCCAAAGTCCTATTCAATATTTTAGAACTAGTCCTGATGTTCCTTCTGAACCTAGATCAATATCTATTTATTCAAACTCCAGCTCTGAACTTGTTTTACACTGGCGACCTCCGCTCCACCCTAATGGCAATGTAACCCATTACATCGTTACTGCTTTATTAGAGAGGGACAGTGAAGAATATTCATTGGAAAACCGGAATTATTGTTTAGAACCAATTACTATAAGATCTTCACCAGGGATTCTTAAAAAGAATTTTGAACCTCAGTTAGATTTATCTGATGATGTGATCGATGATTCTGAATCCTGTCCCTGCACCCAAAAACAGTTTGATACTAAGCAGCAAAGGGAAAATGAAATTCACTTTGAAGATCATTTACAAAATCAAGTATATGTGAAAAGAGAAAAACGCGATGTTAGTAATATGTTTGGTGATTTTGAAAATGAAGAATCTGATTCAAAGGAATCTGCTGAAGAACGGAATGTTAAAGTCAAAGGAGTGTACGAAAAATTTCATCAGGTAGTTTATGGGACTTCATTCATCATACATAATTTAAAACATTACTCTTTGTACAATGTTGCTGTTCAAGCATGTCGTGAATGGGAAGATGATGAAGAGACAAATAAACTGACAAATTGTAGTAAAGAAGGAATAATTGCTGAACGAACTCTCCCTTTGGCTAATGCAGATGACATGGACAGTCTACTCTTAACCTGGGAAGTTTCTAACACCACTCACGGTGTTGTAAAATTAAAATGGGAAGAACCACCAAACCCTAACAGTGTTATTGTAACTTATCAGATAGAATATTGGCGTTCGGACATTGAAAATTATAAACCCATAGTTGAATGTATATCCAGAAGCCAGTTTAAGTTAAAAGGGAACACTCATACTTTGAAAAACCTAAGCCCAGGAAATTACAGCTTTAGAATGAGAGCAACTTCTATGGCTGGAAATGGAAATTATACCAGCATTAACATTTTTTATATAGAGGAGCATTCTATTCCTCCTTTACACATTGTGCTGATAATTTCAATCATGATAATTATATTTATATGCTGTGCTATATTTACATTCTTTTACGTTAGAAGGCAATGCGGAAATATTCCAAATATGAAGTTGATAGCTGAAGTTAATCCAGAGTACATTCCTTCTGTTTACATGGCTGATGACTGGGAAGTTGCTCGTGATGAAGTTGAATTAATCAGAGAAATTGGCCAAGGTTCTTTTGGAATGGTTTATGAAGGTATAATGAAACCATCTGGCTCAGTGCCAAGACGTTGTGCTGTCAAGACAGTCAACAGTAATGCATCTGAAAGAGAAAGAGTTGAATTTCTAAACGAAGCTTCTGTAATGAAGGCCTTTAACACTCATCATGTCGTGAGGCTTTTAGGAGTGGTATCACGTGGTCAGCCTACTTTAGTCATCATGGAACTAATGGCTAATGGTGATCTTAAAGCATATTTGCGTTCTTGTCGTCCTGATACTACAACAAATCCTAATATCAAACCACCTACTTTGAAAGCAACCTTGCGAATGGCTGTAGAGATAGCTGATGGAATGGCATATTTAGCTGCTAAAAAGTTTGTTCATCGAGATTTGGCTGCTAGAAACTGCATGGTGACAGATGAACTTACTGTGAAGATAGGTGACTTCGGTATGACTCGGGATATCTACGAAACTGAATACTATCGTAAAGGATCCAAAGGATTGCTCCCTGTAAGATGGATGGCTCCTGAAAGCCTGAAGGATGGTGTATTTTCTAGCCGTTCCGATGTTTGGAGTTATGGTGTTGTTCTCTATGAGATGGCCACCTTGGCATCACAGCCATATCAAGGTCTTTCCAATGATCAAGTGTTGAGGTTTGTGATTGATGGAGGCATTATGGACAAACCTGAAAACTGTCCTGATACACTCTATCACGTGATGAAAATGTGTTGGCAACACAAACCCAAACAGCGGCCCACATTTATGGACCTTGTTGGGATGCTCCATGAAAATGCCAATCCTAATTTCTCTATGGTTTCATTTTATGACAGTGATGAAGGCCAAGATTTTAGAGGTGTGGGAGAAACTGAAGACACTCCATTGGGAGTGTCTAGGGATATTGAAGATTTTTCTCTGAGTGATGATGAGTCTTGTAATGGTGTGAATAAAATTGATAATAATGCAATAGATGATGATGAAGATGAAGATGCTGAGGATTATGTAGACAGTGGTTTAAAAAACACTGTTGATTCTTATTCCAATAGAAGTAATGGTGTTGATATTGACGACTTTGTTCTACCTTTACCTAGTACAAGCTCAAGTAGAAGTAATGGTAGAATTACTCCTAATGGTTTTATTAAACACACTCAAGTGAAGACTACTAAGTGTTAG
