# Supplementary material for: The genome of the water strider Gerris buenoi reveals expansions of gene repertoires associated with adaptations to life on the water
Source: BMC Genomics. 2018 Nov 21;19:832. doi: 10.1186/s12864-018-5163-2 (PMC6249893; doi:10.1186/s12864-018-5163-2)
Supplement: Supplementary file 8 — Protein sequences of annotated chemoreceptors in Gerris buenoi : 155 Or, 135 Gr and 45 IR genes. (DOCX 83 kb) [file 12864_2018_5163_MOESM8_ESM.docx]

***Gerris buenoi* chemoreceptor proteins**

Suffixes after the gene/protein names are: C – C-terminus missing; F – genome assembly sequence fixed with raw reads or other repairs; I – internal region missing; J – model spans two or more scaffolds; N – N-terminus missing; P – pseudogene (Z – stop codon and X – frameshift or other major problem).

155 GbueOr protein sequences

135 GbueGr protein sequences

45 GbueIR protein sequences

**155 GbueOr proteins**

>GbueOrCo

MEKSKVKMHGLVGDLWPNIRLMQLTGHWLLEYREDNGGMMRLLRAVYCWIITFLMFSQFAFVLCYLILETYDADQMAAATITLLFFAHSVTKFAYFGLRSKYFYRTLSAWNQVNSHPLFAESNARHRSTAVSRMRKLLMIIGGGTIFSVVSWVVVTFLGEPYKQIPDPDNVNETMSIEVPQLMVDSWYPWDAKNGMSFFLSFIYQLIWLLLALSHANLLDIIFCSFVIFACEQLKHLKEILQPLMELSAALDSVVPNTADLFRSTSTASNLPLMNGENGNGVGTDFDIRGIYSNQRDFSGFQPGGVVSMGNGGVGPNGLTKKQELLVRSAIKYWVERHKHVVKFVSSIGDSYGPALLLHMLTSTVTLTLLAYQATKIEGVDVYAASVIGYLLYTLGQVFVFCIHGNELIEESSSVMEAAYSCHWYDGSEEAKTFVQIVCQQCQKSLTVSGAKFFTVSLDLFASVLGAVVTYFMVLVQLK

>GbueOr1

MLDGLRIALSKGVVQVLGVELNRYHEQLLFDYSKEKLLRPGMLYFSGSLMRIAPEHIFLTVGYNLFLLTTTLTTFILIAFTALFLSMDFKELIMLGLLVVLQLISITSHINYAYKRSDVLKMMQIQMALQGQRVADVSTSAKPLWMFFKKYWAPLMWISWGTYLVAVPAYRYFVLSDAFSLPFPMWFPFETRNLTGFLLAFFFQILSVNCFCVQAITLLPCFFYITMCITKEFQVLIDSLQLIHQDLVVEFQKQLGIQICSHSAMSKFYLYSDPGFVACLNKRLKKIVDRHTLILKGVDTMQTTIAVPLTINLGSAAFVVGFALCFLASESEDFSNKVSIGIITGINTFIIAIICFVGQHITNVGEKVRSELYFVNWYNWPEPMKRSLRIMLERTLNPPTLGAAYFDIKANCDTFATILNSAYSLFNLLLAFKK

>GbueOr2

MLVDLRIYLSKRVLQSLGVQLNRYPEQLLLGFLEQSQMRPVMFYFSGSFMKLAPEYILLTVGYNLFLLTITLTTFILIVCTVLFLSMDFSEYIVVGLLAVMHLLATTCHIHYIFNRLHLIELLKNLIELQDADHVSASPDTLWLFFKKYWAPSCWGSWAAYTVTVPAYRYFVLSDTYSLPFPMWLPFETKHLSGFLVAFIFQLLSINCFCVQAITAIPAFLFITTSIMKEFQVLIDSLKSFHSDTVDEFQKQLGTQVCPRSANKMFALYRDPEFVACLTERLHQIVNHHDLILKSVDSMQTLIGVPLTVILGLGAFIVGFALGFIFSDSEDFSNKVAIGVITVVNTLGGAIVCLVGQQITDAGEKVREELYFVNWYNWPGHMKRSLRIMLARTLKPPTIGAAFIDTKANHATFATILNSAYSFVNLLLAFKK

>GbueOr3

MLEDLRIALSGRVDQFLGADLSTYPEKLLFDFFEQCAKHPYLLYLSGFFMRLAPDHIFLTVGYNMFLLTITLTTFTLIVCTVLFISLDFSDLIIVGLLFFMELVVIIAHINYAFKRPELSIMIKNLMAMQGKHDDISASNDPIRMLVIKFCAPFVWGSWAVYMIAIPAYRYFVLSDAFSLPFPMWFPFETQNLKGFLVAFFFQILSINGVCVQIISTIPLFMFMTMFIMQEFQLLIDSLQSFHEDLVDEFQRQLGIRVRTHSTESMFALYRNPEFVACLKKRLHQIADRHDLILRGVDSVQKTIGVPLAAILVLGAFVVGFAIFLLVSGSEDLSIKVTISTVTLINTLIGVMLCFAGQQITDEGERVWEELYFVNWYDWPEPMKKSLRIMLARALKPPTLKATYVDITANFHTFANMLNSAYSFVNLLLAFQK

>GbueOr4

MEDLRRALSERVVQLIGADLDRYQETVSLDFLEQKLARPSLLYVSGSFMRLTPDHILLTVGYNLFLFTSLFTSLILIVCTFFIVNVNFSHVVVLGHFVVLTVNGLTAHLFYGFKRSSRAELIRLLKVLHKDRIDDEISASSKPSWILFNKYWPLITWGSWGSFMVLVPAYRYFGLSDPFYLPFPTWFPFDTRNTMGFSVALIFQMLAVYCFCTQIVTLIPAFLYLTMCVLKEFQVLIESLQLFHKDMVSEFQKKLGIQVCSRTGKSMFSLYRDPEFVACLNKRLQQIVRRHSLILKCVETMQRVIGVPLTILLGQGSFGIGFAVCFLVSDSDDYSIKVGIIAITIINITVKAVVCYAGQKIMDAGERVREELYFVNWYDWPEPMKRSLRIMLARTLHPPCLSAAYIDIKPNYATFATIMNGVYSFVNLMLAFKK

>GbueOr5

MMEDLRKALSERVVQLMDADLIQEGLMLDYVEQKLLRPVLLYASGSFIRITPENVFLAVGYNLFLLTIMLTTLILIAFTIFFLRIEFSEVIVFGHLVVLTIDVLTVHLFYLLNRSQGVELISIVKMFQEGREGDEMSASSKPLWMLFNRYWAPLLYTSWGAYVVVVPAYHYFGMSDPFFLPFPMWYPFDTQNLTGFVVALIFQLLIVYCIGSQIVTLILLLVYVTTCLLKEFQVLIESLQSFHEDTVSEFQKKLGIQVCSRSAESMFALYRDPEFVACLKERLQQIVRRHTLILKCTEVIQILIGIPLTVVMGQSSSGIGFAVVFLVSDSDDFSNKLGIAIVTVVDIFIVGLLCFVGQKITDMGEMVREELYFVNWYDWPEPMKRSLRIMLEKTLNPPTLCAAYIDIKLNYVTFATMVNGTYSFVNLMLAFKK

>GbueOr6P

MMENLRRALSERLVQFMDVDLIKENLILDYLESKLLQPVLLYASGSFMRFTPEHVFLAVGYNLFLLTIMLTTLILIAFTILFLRIEXFGHLVVLTTDMLTLHLFYLLNRSQGEELISIVKMFQEGREGDEISASSKPLWMLFNRYWAPLLYTSWGAYIVVVPTYHYXGMSDPFFLPFPMWYPFDTQNLTGFIVAFIFQLLIVYCIGSQIVTLILLLVYVTMCLLKEFQVLIESLSFHDDMVSEFQKKLGIQVCSHSAESMFTLYRDPEFVACLKERLQQIVRRHTLILXCTEVIQILVAIPLTVVMGQSSSGIGFAVVFLVSDSDDFSNKLGIAIVTVVDIFIVGLLCFVGQKITDMGEMVREELYFMNWYDWPEPIKRSLRIMLERTLNPLTLCAAYIDIKPNYVTFATMVNGTYSFVNLMLAFKK

>GbueOr7

MFENLKTSLSKKVIEFLELDLETHPEDSVLRFWEIKIGYYIYVSGSYIKVDPDHIFLFLAYNVYLFATMMITYFLIGYTLLFHTPDFFSIVSLIHLIMLITLGIVMHVFLMLKRQPRNIFSETILRLEQTGTRQIEDSSASETMRLFFSKFWGPVFWFWWFLFASSQPLYQYFINSNAFVLPIELWFPFDTHNTFGFIAAFLFELLLINSYCVQAITVVPGFYHMCEMLMIEFSKLIDSLKTMHEDTLSEFKNTEEGRDYKPTTSSFDLYKDPKFLECLRRRYNRIIIHHQTVSSLFENLQEVSGVPLLITFSSGAIIFALGICIIVTTNDYALRIIFAILLTINIFIGGVASHLGQQITDLSESLREELYFTNWYDWPEDMKRSLRIMLARTLEPLGLCTAFVSINSNHDSFATMINSAYSFINVLLAFENQ

>GbueOr8P

MFENLKKRLSKTVCEYLHLEDHLEGAVLRFWMSEVGFYAFVSGSFITVDPDHIFFFLAYNLYLFMVPLIAYFLIAYTLLFHTRDFVKAVSLVHLAVAINMGIATHVHLIFKRKHRATYISTMLELKQTGMTRQVNPVSFSSGTSATKRMRVFFSKFWPPVLWFWWFLFACSQPVFQYFINSKAFVLPFELWFPFNTENTMGFLVAFIFELLVINSYCVQAITNIPAFWYLCEMMIEEYSKLIESLKTLHQDTVSDFKNSEEGRDYKPTISSFDLYRDPKFLECLRRKFNQIILHHQTVISSFEDLQVMCGVPLTIIFGSGAIIFALGICIVVTNVDYFLRKIVTIIIIVNIFIGGIISYLGQRITDVSETLREELYFTNWYDWPEDMKRSLRIMLARTLEPLTLTSSFVTIKPNYDTFATMINSAYSFMNVLLAFEQQ

>GbueOr9

MFENLKKRLSNRVVEYLDLCLEDQSEGLALRFWMSRIGYYTFVSGSFMKVDPDHIFIFLAYNFYLLVVPLITCCLIAYTLVFYTRDFIKAVSLIHLVVAVAIAVVTHLNLMFNRSHRNAYIETMLKLEETGLTRQVEHSTARKIMKLFITKFWPPLLWFWWFLFATSQPVFQYFINSKPFVLPFELWFPFDTHNTVGFLVAFIFELLVINSYCVQAITNVPGFWYLCDLMIDEYSNLIDSLKTLHQDTVSEFKNTEEGRDYKPTISSFDLYRDAKFLECLRSRYNRIILHHQTIISLFEDLQAISGLPLTIIFSCGSIIFALGICIVVTSGDYSLRIIFTILISINIFITAIISYLGQRITDLGENLREELYFTNWYDWPEDMKRSLRIMLARTLEPLALTSSLVTIKPNYDTFATMINSAYSFMNVLLAFEQQ

>GbueOr10F

MFENVKKRLSKIVTECLDLGLETHQSKIGYYTYTSGSYIKVDPDHIFLFLAYNVYLFVTILLTYFSIAYILLFHTPDFVNAVSLIHLIMLITLAIVMHVFLMFNRKPRNAFTATILSLQQTGTRQIEGSSASETMRLFFSKFWGPLFWFWWFLFASSQPLYQYFINSNAFVLAIELWFPFDTHNTFGFLAAVLFELLVINSYCVQAITNIPGFWFMCETLIDEYMKLIDSLKTFNEDTLSEFKNTEEGRDYKPTTSSFDLYRDPKFLECLRRRYNRIIIHHQTVISLFENLQAISGVPLSIIFGSGATIFALGLFIVVSTSDYSLRIIFTILLIINIFIGGIVSYLGQQLTNMSESLREELYFTNWYDWPEDMKRSLRIMLAKTLKPLELSSAFVTIKANHDSFATMINSAYTFINVLLAFENQ

>GbueOr11

MFENLRQRLSKRVSLFINVHLEDHPEDIVFGYWESKLGYYIYASGSFIRVDPDHIFIFLAYNFYLFVIQFITYYLIGYTLLFHPGGFVHVVSLVHLGVIMIVATTTHIHMMFNREHGAIYIERMLKLENTGTRRQINDSFTRENMRLFFTTFWPPIVWFSWFFFAVSQPLYQYFINSKAFVLPFELWFPFDTHNTLGFLAAFIYELQVINSYCMQAVTNVPAFLFLCNMMIDEYTMLIDALKGLHQDTVSEFKTTEEGQDYKPTISSFDLYRDPKFLECLRRRYNLIILHHQTIISLYEDLQMVSAVPLTVIFGVGGLVIALGVSAVISEYGDISFKIIFTIVVLINLFVAGIISYLGQRLTDLSENLREELYFTNWYDWPEDMKRSLRIMLARTLEPLELTSAFVTIKPNYDTYATMINTAYSIINILLAFQK

>GbueOr12I

MFENLKKRLSKIVCECINVHLEDHPEDMVFGFWESKLGFYTYVSGSYIRVDPEHIFIFLAYNLYLLVTVIIAYFLIAYTLLFHTRDFVKAVSLIHLVVAVTMGIVAHLHLMFNREHLAVYIETMLRLEQNGMTRPVEDLTTRENMRLFFTKYWPPVFWFSWFLFASSQPVYHYYINSKAFVLPIELWFPFGTDNNVGFLGAFLFELLVINCYCVQAITNIPGFLFLCNMMIDEHMMLIDSLKALHQDTVSDFKNTEEGRDYKPTISSFDLYRDPKFLECLRRMINSAYSFMNVLLAFEQ

>GbueOr13

MFENLRKRLSKKLSEFLKVNLKDHPEDIVFGFWERKLGYYIYTSGSYIRVDADHIFIFLAYNLYMFMTPFITYFLIGYTLLFYPGDFLNVVSLVHLGVLMIIVTFIHIHVMFNREYGVLYVIDTVQKLENTFTRRQPKDSLTREKLRLFFTKFWPPVVWFCWFFYASSQPAYQYFINSEAFVLPFQLWFPFDTHNALGFIGAFVFELSVVNCVCSQAITNIPAFLYLCNMMIDEYTMLIDSLKTLHQDTVSEFKDTEEGRDYKPTISSFDLYKDPKFLECLRRRFKLIILYHQTIVSLCERLQIVCSVPLTVILGMGGFLVALGICVVLSAFSDFSLKLTFTIVVIVNLSVVGIVCYLGQRLTNLGENLREELYFTNWYDWPEDMKRSLRIMLARTLEPPTLSSAYFTVKPNFETYAAIINTAYSMINLLLAFQK

>GbueOr14I

MFENLRKCLSKRVSEFLTVNLEDHPEDIVFGFWEKKLGYYIYASGSYMRLDADHIFIFLAYNLYMFVTPLITYFLTGYTLFFYPGDFINVVSLAHIGGFMVIVTSAHMHVMFNREYGVLYVIDTVLKVEHTFTRRQPKDSYARENIRLLFTKFWPPVVWFTWFFYASSQPAYQYFINSKSFVLPFQLWFPFDTQNALGFIGAFVFELLVVNCVCSQTTTNVLALLYLCNMIIDEYTMLIDSLKTLHQDTVSEFKDTEEGRDYKPTMSSFDLYRDPKFLQCLRRRFNHIILYHQTIIIINTAYSMMNLLLAFQK

>GbueOr15FC

MFENFRERLSKRVGEFINVHHEEHPEELVFDFWETKLGHYFYASGSFMRIDPDHVFIFLAYNFYLFVTPFISHFLIAWTLLFYPVDFADVISLVHIGIILLLGVTVQIHMIFNREHGNIYIETILRLESNRTRRKPGESSTREKLRLFFTKYWPLVMYFSWFFYISSQPVYQYFINSRAFVLTLELWFPFDTHNILGFLAAFLYQLLMANCFVAQAITNIPGFLYLCNMMIDEYSILIDSLRTLHEDTVTEFRNTEHGRDYKSSISSFDLYRDAMFLQCLRRRYNRIIHHHQTIISLYEHLQAVSGVPLTVISGEGGLIVAFGISVIISGFGDYLFKVIFTTVDVMNLFIGAMICFLGQRLTDLGESVREELYFTNWYDWPEDMKRSLRIMLARTLHPPALTSAYFTIKPNFSTYAT

>GbueOr16F

MFENFRKRLSKRMSEFINVHQEEHPEELVFDFWETKLGHYFYASGSYLRIDPDNIFIFLAYNFYLFVTPLITYLLMVCTLLFSPFDFSNFVSLVHIGILLLLGVTVHIHMMFNREHGNIYIETILRLESNRTRRKPGESSTRENLRLFFTKYWPLVMYFSWFLYISSQPVYQYFINSRAYVLTLELWFPFDTHNILGFLAAFLYEMLTVNCFISQAVTNIPAFLYLCNMMIDEYSILIDSLRTLHEDTVTEFRNTEHGRDYKSSISSFDLYRDAMFLHCLRRRYKRIIHHHQTIISLYELLQVLSGVPLTVILSAGGFIVAFGISALISGFGDYLFKVIFTTVVILNLFIGAIICYLGQRLTNLGESVREELYFTNWYDWPEDMKRSLRIMLASTLQPTSLTSAYLTIRPNFDTYATIVNAYTLNSFDLNAWL

>GbueOr17FC

MFENLRKRLSKKVCERLNVHLEDPTEDIMHGFWQSRLGYYFYTSGSMIKVDPDHIFLFLAYNFHLFAAVVIAYFLIAYTLLFYIRDFVKMVSLIHLVMAITIGIVTHVHLIFKREHGSTYLQTMLELQRTETHRRPKDSFVRKNIRLFFNKFWPAGFWFWWFLFASCQPAYQYLMNSEAFVLPIELWFPFDTHSTSGFLVAFLFELLVINCYCVQAITNIPAFWFLCNMMIDEYSVLIDCLRTFLCVLEFGHRVSTVSEFKNTEEGRDYKPTMSSFDLYRDPKFLECLRRRYKRIILRHQTVVSLFGDLQALCGNPLTVLFGFGATVFALGICIIVSSADYSLRIVFTILIIIHIFIGGIVSYLGQQLTDMGENLREELYFTNWYDWPEDMKRSLRIMLANTLEPPALTSSFVTIKPNYDTYAT

>GbueOr18FIC

MLENFKNRLSKRVSEFINVHQEEHPEELVFDFWETKLGHYNFNASGSYLRIDPDNIFIFLAYNFYLFVTPLITYLLMVCTLLFYPFDFSNFVSLVHIGILLFIGLTVQIHMTFNRELGNIYIETILRLESNRTRHKTEESSTRENLRLFFTKYWTKVMYFSWFLYISSQPVYQYFINSRAYVLTLELWFPIDTHNILGFLAAFLYQLLMANCFVVQAITNMPAFLYLSNMILDEYSILIDSLRTLHEDTLTEFRNTEQGRDYKSSISSFDLYRDAMFLQCLRRRYNRIINHHQIIIRGVGDYLFKVIFTTVIILNLFIGGMLCYLGQQLIDLGETVREELYFTNWYDWPEDMKRSLRIMLARTLQPTSLTSAYFTIRPNFDTYAT

>GbueOr19I

MLENFRKRLSKRVGEFINVHQEEHPEETVFDFWETKLGYSFYVSGSFMRIDPDHIYIFLAYNFCLFVTPLIMFGLIACTFLFDSVDFVNAINLFHLGLLMAVGLTVQTHMIFHREHGNIYIETILRLESDRTRRKNEKSSTRENLRLFFTKCFSPAMWFSWFLYVSSQPVYQYLINSRAFVLTVELWFPFDTHNILGFLAAFLFEVMMVNCFSAQPVTNIPAFLYLCNMLIDEYYILIDSLRTLHEDTLTEFRNTELGRDYKSSMSSFDLYRDAMFLRCLRRRYKQIIHHHQTIIRMVNTAYSFMNVLLAFQQ

>GbueOr20P

MFENLWKRLSKRVPEFLNVNQADXEDKALVIWESKLGFAIZASGSFMSIHPGHIIIFFTYNFFILVAPIITYFLMANTLFFYPGDFSNSISLIQLGVLLFIVTVVHIHMMFNREHGVKFVISLIFTKFWPPVYWSIWFLYAISQSVYQFFINSKAFVLPFELWFPFDTHNTZGFLGAFLFELIVXNCYCVQANTNILALLYMCNTITDEYLMLIDSLZTLHQDTVTEFKTTGEGWDYKPMISSFDLYRDAKFLECLRTRYNRIILHHQTVIR

>GbueOr21P

MFENLKKRLSKKVCKYLDLHLEDNSEEIAVQFWKTKIGICTFVSGSFMRCDPDHIFFFLAYNFYLLXYFLIAYKLLFHTRDFVKAVSLIHLVIAITIAIVTQVHLLFNRNHRSAYIETMLRLEQIGKKRQVEHTSVREILRLLFFKIWPPAFWFSWFLFASSQPVYQYFNNSKPFVLPFELWFPFDTQNTIGFLVAFIFELLVINCYCVQAITNIPGFWYLCELMIDEYSKLIDSLKTLHQDTVSEFKNTEEGRDYKPTISSFDLYRDPKFLECLRSRYNRIIFHHQTVIRSFENLQGISGVPLTVIFGSGATILSLGICIIVTTDDYSLRIIFTILIIINFFIGAIISYLGQQLTDLGETLREELYFTNWYDWPEDMKRSLRIILARSLEPLALTSSYVTIQPNYDTYASMINSAYSFMNVLLAFEQQ

>GbueOr22PC

IHIGMLLLIVTAVHIHMLFNREYGVKFTETIVHLELFZVPKDSZGKPKDSFKRERIRLIFTKFWPPFYWFIWLLYAISQPAYQYFIKSRAFVLPFELWFPFDTHNKLGFLGAFLFELFVINCYCVQAITCIPALLYMCNTITDEYLMLIESLKTLHQDTVSEFKTTGEGRDYKPTISSFDFYRDAKFLECLRRRYNRIILHHLNVIRMFEDLQACAGVPFTMILGMGSVIIALGISLIISDYGDFSLKIIFAIIIVLNLAGAGIICCLPQRLTDLGETLREELYFTNWYDWPEDMKRSLRIMLARTLEHLALTSAFFDIKPNFDTYAT

>GbueOr23

MPTLRVPVCKLLRSYLLIDYERGCDDNFYQLFERELGEKTLIYSGSFVKLTPENVWWFIAHNAYVLVTSAIMLFLSIFTCIYADIDPASAVLLIHMAAVMLITIICHRCNTIHRLHCARYSKTFAELHSKLSFDNKSRTHRIALTFLKYYWPKLFWSSWSLYMICIPTYSYFINSNPYVLPFKLWFPFDTKNRSGFIVAYIFEILGVNTLCLQVMTLVPFFYLISIIIVVEYDTLVTSLHSLHERSLSEFKKSWHSPESWPIQSTFDLYKEPEFIYYLERNFRQIILHHGAILRMIENVQKLSGDGVLAVLGFGALPIGASAYFLVSDIGDGLMKVTFAILFIIYIFIGGIICNGEELREELYFTNWYDWPDGMKRSLRIMLAKTLVAPALTATFFTITPNNDAFATMLNGAYTFVNLAALAPGSN

>GbueOr24

MNRGAWRERLSNLFGFSSFQNLRLSLSRKFCSFINLDLERYPEEPFCKYFEAKMGNNIYFSGTYMRVSPQHILSTVFYNLYLIVTPTIVFVLIACTWIFRPIDMSESVLLFYLFVLMLLSFTTHINMWLHRKQAAIYHEILIILENSNRPEDIKSYDRETVRIFFTKYWPILIWGSWAIVMILSPIYSYFFEKKSFDLPFPLWFPFEVNNFAGFLVAYIFENLAINCFCVQTITLIPGLLHGCNCLITQYNMLISSVITLHDDSVSEYYKIRASSSELDSQDLYRNPQFNICLQKCFKQIFDHHQTIFSLQVDCMQVASRVPLSLVLGLGSLIIGATAVFLVRDIGDLSLKVAFTSLFVVNTFGIGLVCYLGEWVHSLGEKLREELYFTNWYDWPEEMKRSLRIMLAQLLKPPTLTAAYIRVKPSYATFAKIMKGAYTFINLMFVFKDYSKLTSNPSSHPPS

>GbueOr25JIC

MLEQFKILLCEKIIKYLHSEFYGVSLEPIYDYFVRRMNKIIYFSGSFMKVEPKYVLWVVMYNTLLAVIPFLVYILAAMTLIKVDLELPALIFLGHFICLVFITGMEHIYVTIHSAEGIKYGTILVGWEKLNRDYYSETNLRAKLQKFFLKYFPLMVWGTWGLYMGIMPVFDYYVNSKPYCLPFPLWFPFDTTRPIGFLAGYIFEILSVNGVCVQVITGVPTFVYITTIIFAEYNVLNNSLRTLHVDALSTFKTKYKSSDPKPSGYELYKDQEFLFCLRKHFKEIIEHHQSIIRDLSVKLIFGGLLIVNFFIIGVLCYLGEQIGEKVREELYFTNWYDWPEDMKRTLRVMLARTLYPPTLSGAFITITPNYEAFATVKTTQISQQLL

>GbueOr26C

MLKKFKYILSKEISDFFKGDKDSENDFYLFFKSKMGNKIYVSGSFMRRDPDFILGTVVYNLSLIVTKLILLILTAVTLKFRRNELLDLYVVVNFSALILVAFTLHVQALINRRKTIRFGEHLLKRDRTDRYDRTSNDNRLIFKTFAQFWPYMISSTWFLFFIGNPIYSYVFYNSIALPISLTFPYNTRNCMGFLTSYVFEMYAGFALCVQMITNVPTFAYIYNLLISEYKVLVSSVASLHEDCTIEFYKKWDGRSPSSDQNMLDLYRDPEFVVCLQRCFKHIMDHHKTILRLQECVQTISCVPLTFILGLDSLIVAATGSLLVTVKDKELSLKIVLIGFLIMKASFIWLICFAGQQIKTLGEKVREELYLTNWYDWPEGMKRSLRIMMARTLNPPVITCCYLDIRPNYALFAT

>GbueOr27

MLLNLRVTLSNGVCKFLNVDLEECPEKRIMKYLGSTIGLLALRNNGIYFEIDPENILKFLGYSISLAFTSLLCMVLIICTFSFRDVDLSNTIVLSHLAIIMFLSLVTQLHIGYYRVEGKLLVEQIILWERPCQTMSPQAELALKFFRYYWAPIVWTSWAIFMTISPLLDYYMKSLSYNLPFPLWFPFDTQNTFGFLLALLFEIYAVQTFCAFFIFSVPLFFFLSIKAIAKYEKTIEAIKSMHKDSLAKFKALRLAGEDNPIFDPITDEDYFDLYKEPKFQAICEETFSEISRSHQELLRCIKLLRIVSNVPLTVVLWLGSLIIGAAICFIVIDVGDLTLKVSFTVFGSVDTAGLWVLCYYGAQIYYLGEEVRQELYFTNWYHWPEGMKRSLRIMMARILSPPSLSAAYVDVKPDFETFAKMMNSAYSFINLLYAFKN

>GbueOr28

MLRAFRIALSAKLRKAFRVDSDPDHDKRLFDWLDNMIGPLYLAIGGYFWKLDPEHVIQLVCYNISLYAVATVFIVLFICTLMFKDVEITSLSVLFNFTCGISLFLVFHIDLFLRRRLEITSIFYQIIILSELKQQSKNQSFEVKLERVIRVYWAPLVWTTWGCLMVSIPVSDYLLYGRAYSLPFPLWFPFETNNPLGYAAAYVFEIYAVHFFCSFAIASVPIFFLICNVALNAYNSLIESIRSMHDDALAMYKLQLGERRNDSTDPATDEEYFDLYKDRVFQSCCERCLGQINILHLKISSVIYDINGAASLIITLALTIAYLAIGAAFCLAVTDAVDMTMKVTFTATGIMDLIFIWFVCHLSQILIAKGEEVREALYFTNWYHWPEGMKRSLRLMLVRSLYPPHFSASFFDIKADYITFAAVVNSTYTFVNLVYALTH

>GbueOr29F

MLRDYRIWLSAKLREALRVDLDQADEERIFEWLDNNIGPFYMAVGGYFSKIDPDHVIQLVCYNISLYLVAFVFIVLFFCTLMFEELDITSLCVLFNTSWGVFLFLICHLDLFFHRFEVVGVGNQLLTLFKLMRRSQNQSSLEERILKVFRVYWAPLVWTTWACFMVSKPVFDYLSNGRVYSLPFPLWFPFETNNSLGYAAAYVFEIYTAHFYCSFIISSVPMFIAITFAILIAYNSLIDSIRSLHDDALAMYKLQLGERTNDSTDPATDEEYFDLYQDRVFQSCCERRLRRINILQLQISRVINDLKRRIYLPVTMALSISYMAVGAAFCLAVIDTGDITVKMTFTATGIVDLLLIWFICHSSQTIITMGEKVREELYFTNWYHWPEVMKRSLRLMLVRSHYPPHFSTAYLDIKADYITFAALVKSTYSFVNLVYTFTH

>GbueOr30JC

MLRDFRIALSAKLRETLRVDSDPGTDKCMFDFLDYKIGPLYMAVGGFFSKLDPEHVIQLVCYNISLYLVAAVFIILFICTLIFKELEITSLAVMYSVAMWLFMFLIAHMDFFFHRSELTAIYKQMIDWVDLNRRPQDQPFSEEKLLKVIRVYWAPLVWTTWGCFMVSIPVLDYLSNGRTYSLPFPLWFPFKTDNPLGYVAAYVIEIYAVHAICSFIIACAPLYFVVCYVILEEFDSLIDSIRSMHEDALAMYKLQLGERTNVSTDPATDEEYFDLYKDRVFQSCCERLLRQINVRHLHIFRVTKEINRIISILVTLALTLAYTATGAALSLGAIGNGNIKLKLTFSATGLMDLLLIWVICHLSQKLKIKGEKVREELYFTNWYHWPEVMKRSLRLMLVRTLYPPYFSTAYLDVKADYITLTA

>GbueOr31C

MLRDYRIWLSAKLREVLRVDLDRADEDRVFDWLDNMIGPFYMAVGGYFSKIDPDHVIQLVCYNISFYLVASVFVVLSICTLMFEELDIASLSVLFNSSLGVLLFLIYHLDFFFHRFEIVGIGNQAINLFELTRRPQNQSASEERLLKVFRVYWAPLVWTTWACFLVSKPVFDYLSNGRLYSLPCHLWFPFETNNSLGYAAAYVFEIYAAHFCCSFIISSVPMFIAISFAILKAYNSLIDSIRSLHDDALAMYKLQ

>GbueOr32C

MFRIALSNKLCEALRVDLDAAADERVFGFLDNMIGLFYLAIGGFFYKIDPEHVIELVCYNISLYLVSFLFVILFICTLIYRELEVTSLSVLLNFTIGILMFITVHIDFFLHWLQMKGILKQIINWAEFKQRPTIQSAFETFSFKFVRVYWAPLVWTTWGFFMVSTPVFDYLSNGRAYSLPFPLWFPFETNNPLGYAAAYVFEMYAVHFFCSFVIACVPLFFLACYAISNEYDSLKDSIQSVHDDALAMYKLQLGERTNVSSDPATDEEYFDLYKDRDFQHCCERCLRQINIQQLQIS

>GbueOr33I

MFQNALRAKLCEVLRVNLDPAYDERVFGFLDYMIGLFHMAIGGFFYKIDADNVVRLVCYNISLYLVSTLFVILFICTLVFKDLEITSFSILLNFTVGTSMALAIHLYFFFHRCEMAGLINQIINWVEVKPRPKGQSSFETFSFKFSHVYWAPLVWTMWAGYVVMTPVFDYLLNGRAYTLPFLLWFPFKTNIPLGYAAAYVFEIYAVYFFSSLINACVPLFFLICYAFLNVYDSLLDSIRSLHEDALAMYKLQLGERTNVSTNPATDEEYSDLYKDRVFQYCCERCLRQINIQHAEIIGIGDITLKVTFTAIGIMDLLLVWFICHLSQLLMLKGEKVREELYFTNWYHWPEGMKRSLRLMLVRSLYPPHFSAAYLDIKADYITFAAVVNSTYSFINLVYAFTH

>GbueOr34

MFRITLSTKLCEILRVNLDPAYDKRVFGFLDDMIGMLNITISGLLYKISPEIVVRLVCYNISLYLVSALFVVLFICTLIFKELEITSLSVLLNFTVGTLMVLAIHIQFFLNRFEVTRALNHVINWVELKPPQKGRSSFDTFSFRFTRVYWAPLVRTMWAGYVVFTPVFDYLSKDQPYSLPFPLWFPFKTNNNLGFTAAYVFEIYTVHFFSSFIVASVPLFFLVCYAILNEYDSLLDSIRSLHEVALAMYKLQLGERTNVSTDPATDEEFFDLYKDRVFQYCCERCLEQINNHHVEIIRFMKDVKRIIYLTVTAALTCGSIFVGPALCLAVIGIGDTTLRVTFTAIGIMDLLLIWFVCHFSQMLIVQGEKVREVLYFTNWYHWPEVMKRSLRLMLVRSLYPPHFSAAYLDIKADYITFAAVVNSTYTFINLVYAFTQ

>GbueOr35

MFRTTLKDKLCKGLKVDLDPAYDERVFGFLDNMIGLFIISIGGLFYKIDPEHVIRLVCYNISLYLGAVLFDVLFIFTLIYEELEITSLSVLLNFSVGTLMALGNHVEFFLHRFQATRAINQIINWVELKPRQKVQSLYDTFSFKFTRVYWAPLIWTMWAGYMVFTPVFDYLSNGRAYSLPFPLWFPFKTDNPLGFAAAYVFEIYVVHFFSSYIVACVPLFFLVCYSILNEYDSLIDSVRSLHEDALAMYKLQPGDRTNVLTYVATDEEYFDLYKDRVFQSCCERCLRQIIIRYSEIIRFTKNVNKIILLTITMALTCAAVFVGPALCLAVIDIGDFTLKVIFTAVGILDLLLIWFICHFSQLIKVQGEKFREELYFTNWYHWPEGMKRSLRIMLVRSLYPPHLSAAYLDIKADYITFSTVVNSTYTVINLVYAFSQ

>GbueOr36a

MFLNLRSRLSRKLCRMFGVDLVKDPEQVLECCLIKSLGSVVFYSSGFYMRIDSHNVFFYIIYNMILILLPGIFLVLFFCSLLFESHRLIDLIFPFYVLLLMIMKFYGRIHLGIRRSKTVEMVKMIVHWESLNRCPRKETILERIALQFLRYFWAPTLWLLWVSYLVSYPLIQYYFYSNPYTIPLLLWFPFSTQNPTGFWAAYIFELFAATSNCAVGVYGSSLYLHTALTIRHEYNILIDSIRSLQEDALAKLILKRTGQEVNVTVLDVDNVNLFKDPAYISCLEETFQQIVDHHQTLIRFIETYDKIMCVPLTIVFGLGSSLIGVSLFILVIDIGDFTLKACSACFSLLEIIAVWILCYYGQQISLLGENVREELYFTNWYHWPEGMKRSLRIMLARSLKQPILTAAYMNWGANYETFSMVVNGAYSLINLLHAFKHRTD

>GbueOr36b

MESRLTMFYSLRSSLSLWLCRVLGLDLEINREEVFRKGLVRILGPLVFYSSGFFMEIGPRSVILYICCNLMLLGVPALHLTQIVCSLMLKSYDWLTMIILFHLAILMILTLYGRVCFEISRADTVRMVDMIVRWEDMNHSPEVPSRVEKATKLFFRYYWAVFTWIIWTTVMISYTIAQYLTNLNVYTLPFPLWFPFETDHAAGFVVAYIFELSAAYTNSAVANYAYVSYFQTALTIRHEYKFLVNSIRSLKNDAFTMTMQKKAGRVRFKIMTEEDYFDVYQDPTYVSCLEHLFYRIIDHHQDIIRFIETYDKIMCVPLTIVFGLGSSLIGVSLFILVIDIGDFTLKACSACFSLLEIIAVWILCYYGQQISLLGENVREELYFTNWYHWPEGMKRSLRIMLARSLKQPILTAAYMNWGANYETFSMVVNGAYSLINLLHAFKHRTD

>GbueOr37C

MLDSMRNRFSRTLCERYKANLNEEEALMDYVKSLLGNALFFNGAYLSFKPQSVLVFVFFHTYMIVSESCALVLIANTIYDSQLDMGNMIVIMHLTLLLIISTSVHLSFVSRRYNISEVCKIIKKLSESDRFVDESSKKFMEFVRKAIVNWWPIMVAFALGSILCLKPVYDYSVNSKITIPFPLSFPFDTNTPIGITVGFVFEILIVSSIGLQLNGGLTMYILIQQNIVFEFNNLITSIQNIQTDSILSMNAQNKKYLKRENLEINQPVSVKEFYNLYNDPTFSACYEKKFLDVIKHHHTILRMFFHHQQIAGAAILVVIALSSSMVAVSIYCIMSVRRSKFGFESKHLHFSSSRSICHLGNLLLWRTDIVF

>GbueOr38C

MLLISFWISRALSICFLSIQFYESHKKMFETMRDELSKKMCKRFMVSLEDEDEVMDLRKSDYGGKLLSISGVYISFKPQSIFFFTIYNLFLIIAEVVSIISMGYSIYSLQLDVDDFIIEFFLVILIILSISLHWPFVINRYDMTEAFKLIINLKNQDRVVDEQQIKVLELVNKMSVVIWPILITLSMISIYCSRTFLKYFINTKLILPYPIAFPFDTETTVGFTVGCCLEVIPIYCISAQLNGGLSTYTMTQQRIVSELHILINSIKGLQKDSLVLMKARSKQDMTKEDVDIKEPESVEEFYNLYNDPTFSTCYEQKLIDLVKHHHKILRLLCHHQIYGGVPLLTLLTVASSSMVGIPIYCILSEESTLSLKLTMALIFCPFALSIWVICNYGQQTASLSAEIRYELYFTNWIHCSESVKKTLRILQANTLNPFGIGGTGFTIKANYETFTT

>GbueOr39I

MPSIMDLRFILSEKLCKILSANENEEDNISAYLCSKFGYLFHISGCFLKLKPDSIFLFIIYNLFLSFFYISGTIYSIYSLTYQPITNLTDAALILQFGLAMLVCGFVQVSMLACRGDCYQIYFIWSHLDSSKRQIDIEVEKKLEVARKLSVYYWPSIWIPCWILVLILRPLYEYFVNSKQFEVPFHLYCPYDNAFAILILYFYEILSVYAAAAQINIGFPLFVYAYERSLAELKMLITSIEHLEREALNEFNRKRKLCEIISPTTLKDFYYLYKQPEFLELYSNGLDRIIKHHQQIDKVLGYYKRISGIPLLLILSLTPFTIGGAADVDFGLKVGAILFTLVEVCGVWFFCDIGQELNNKIMQVSYKFINLLYAFQQQQKK

>GbueOr40F

MFSIMALRLKLSEKLCKILSANENVEDNISEYIYSKFSYLFHINGCFLKLKPDSIFMFIFYNLCLSFLYISGTIYSIYSLMFQPITNLTDVVLISHFGLGMILSGSLQVTFLACRGDCYQIYFIWSHLDSSKRQIDIEVEKRLELSRKLSVYYWPSIWIPCWMLVFILRPLYEYFINSKQFEVPFRLYCPYDNAFAILILYLYEILSIYSLMIQLNIGLPLFVYTFERSLAELRMLITSIENLEREALNEFNRKRKLCDKISPKTLKDFYYLYKQPEFLKLYSNGLDRIIKYHQQIYRVLEYCKKVCGMSLLIIFSITPITIGGDVDIGLKVGAILIALLELCGVWFLCHFGQELNNKLSQVRNAVYNTRWPDFTEDAKKSTRILQEVTLKPLKIRETVFGVQLDLNLFSNMMQVSYKFVNLLYAFHQQQQEN

>GbueOr41C

MIMKSLKLFVYHKIINFISTSVEEDEISYIRVSKDFGKLFIFNGCFMRLTLNSCMFVLLYNLLISIVLWAGTFLVGYTLIVKHNDFKMHEMVFLMNIGVLVVLSAIIHTTFFVRRIDAIILYRLMKEADKYREIDKEDLAESERIWKLSCRIWVVLIMIACPSLFLPSVFIDKEADLDNSTGHLPLPIRFPFDTTHPFEFWAASIFQIVMVIFIAMELSAALPAYLFVQKIVLELRSIIAAIKTVHDKSLQMTMKLKGASFMARWRKINLDNDSSKENNFHYYLYKDPIFLECYEKNFRDIIIHHQIILKIFSHYQHYGGIAILFVIGFSSFMIAISMYCIMSNDVSIGVKVTTIFVIIPQCLNIWVVCYYGEQTSTLDDGGNDCEWSMWWVHTVAQVAGASNGQVTAPPASILSHLALIGTNHYTKDQPSQ

>GbueOr42C

MFSVNKYLDGLTEMDSDGIGKLIKKDFGSYRFITPIYWDFSKKRRHVVLPYTLFIFSGLFLFLVSCIFLACQFNNMMLATRGAVQGSFALVAIAPFFTFIKYRKSITCIYGLLAGGFGRYGDFPEGEEDGFYKKMDAFKTKLKRAWLIIFCFITVFYCLYDKMEAVIVPSEEIIHTEGVNKNFHIPLWLPFDAEEPIIFFPTFLVLLYLSASTGFGIMVLSYAFVIFGQRIINQIERLLYAIDHLEERSVCQWEKITSDSTKIYNLNELYKNRQFVALHKMCLRQTVVHHQLIIK

>GbueOr43C

MFSVNKFLDGLTEMDSDVVGKLIKKDFGFYRFITPIYWDFSKKRRHLVMTCTLLLFSGLFIFLLSCIGVAFQYENVMLATRGAMHCSFILVDIAPFITFIRYRRCITYIYGLLAGGFGRYGDFPEGEEDGFYKKMDTFKTKLKRAWLIIFCFTVFFYCLYDKLEAFIMPEEEFAQIIGVNRKFPVPIWLPFDSEAPVVFYPILIIMLFLTVKTAFGIMGLSYAFVIFGQRIINEIERLLYAIDHLEERSIHQWKIIMSNSTNIGSLNELYKNRQFVALHKMCLRQTIVHHQLIIK

>GbueOr44

MDLLDRFTREDDPDVTEGIFKDFGNTLYYLGIFRSVAKDKVAVTVIFYTLVSLLFMFFIGAMSYTVYLAMPILSEIITLVHLALLIFLALIMVHAAFLKKPTIDRLYRYFYVELNRFSYTYDPIIVSFQTMRAKYTKLITKTALFLISAAGIIVILLEPLLNVFFDEEIIKIKGVLYGVPIPMYWPLDNENYFLGFILPCIVQLTASVAVVGMISGMVCIYSSVCLHIYFQLKVLLHNIKTLEDHVKRAFQQDNHGQVKLSPSSLETLRYYNEFVRDIVQHHILLIDCFEHVNEIGKWILLTLFLSGGIDIAMAAILVFFVEARIGNLVTAIYFSLTEILMFGAVTFMSDMLTDMSMEIRTEVYMLEWYLMRREVKQSLRIILACSQKPLSFKAGNLIPVDKEMFRSIMQTAYSFINLAAAVKS

>GbueOr45P

MGLLDRFTREDDPSLVEGIRNEFGKVLYFLGVFRSMSKEDVVETLLFYSFATFMIVYYIFIFCGSVYVCMPMLAEVIPFIHFILLSILTLAGVHAVFFKKPSLDILYQYFYKETCEFFYSFDPVCVSLQTTRDKYSKIITKVITVMIIACGLSIIFLEPFVKFLLGEEYDRIEGLLYGVPVPIYWPLNHQDYFFGLILPNIIQVLFCGLAVGFLAGIIVLFLNLCLHLYFQLKVLLHNIKSLEEHVKRSVGLENTPKNSTVSSQQALDRYKKIIQDIVRHHIMLIDCYRLVNDASKWIFLVLFLSGGIDIAMAGIMVFFVEGRLATKITAVFICFTEIIMFAAVTFASDLLTNMSLEICEEIYMLNWYTMKEEVKQSLRIIMTSSXQPLCFIAADIVPVNKEMFSXMMQSAYSFINLIAAVKSE

>GbueOr46JI

MGWLERFSREDDPIVVEGIDKEFGKVLYYLGVFRSVSKENVLGTLVFFSCTTILFMFFICIFMITVYLSISELADIISLIHLTLLISLALVAIHGALMKKLSLDILYRYFYGELNRFFYTDDSFNVSLLEKRNKYSKLITRVVTVMILSAGLTFVILEPLLKIILGEDNARVDGLLYEIPIPMYWPIDHDNYLLSFILPTILELLLCGGMSAMISGAVLIYFSYCLHIYIQLKCLLHNIKTLDEHVKTSILQDFGDGVKIFSSSSIALNYYNKFVKDIVQHHVLLVEARIGSLMTAVYFSFMEIIIFGAITFASDLLTEKVSGLEMQTEIYMTKWYLMREEVKKSLMIIMTCSQRPLCIMAGNLIPVDREMFTSVRIYSFINLIAALKSE

>GbueOr47

MGFLDRYAREDDPSVIEGIENEFGKVLYFMGVFRSMSKENIVGTLLFYSYVTFMIIYYLYIFLGTIYVCMPVLAEVITFVHLMLLLILTAAGFHAVFLRKPSLDILYQYFYKETCEFFYSFDPVCVSLQTTRAKYSKILTVVIILMILAAGLSVIFLEPVLKLLLDEEYDKIEGLLYGVPVPCFWPLDNEDYILGFIIPLLSQFMICNLLVGIISGIIVLFLNLCLHLYFQLKALLHNIKSLDEHVKRSVGLENTPKKFDALSHETLDHYYKFIQDIVRHHIILMDCFYHINEASKWIFLVLFLSGGTDIGMAGIMVFFVSRLATKITAVFICFTEIIMFAAVTFTSDLLTNMSEEISEEIYMLDWYMMREEVKKSLLIIMTSSQRPLCFRAADIVPVDKEMFSSMMQSAYSFINLIAAFKSE

>GbueOr48C

MGLLDRFVKEDVPSVVEGMRNEFGNFLYYLGVFRSQSKENILGTVIFYSYTTFMIIYFEFIFCCTVYISMPMLSEMITFVHLILLLILSLVGVHAVFFKKSDLDTLYFYFYKDTRKYFYEYDPVCVSFQRTRDKYSMMITKAITVMIVAAGLSVIFLEPFLKFLLDEKNERIDALLYGIPIPTYWPLDHDDYVLGFVLPSAAQLLVCSSVVGLIAGIVVLFLNICLHIYYQLKVLLHNIKSLDEHVQRSASCENIRQKFTPLSQDALDRYKTFIRDIVQHHIILIDCFRRVTHASKWIFLVLFLSGGIDIALAGIMVFFVSRLATKITAVFISFTEIIMFAAVTFASDLLTNMSLEICEEIYMLNWYSMREEVKKSLMIIMTSSQRPLCFRA

>GbueOr49C

MGWLERLSREDDPIVTEGIDKEFGKVLYYLGIFRSVSKENVLGTLVFYLCTSALLTFFVTFFMITVYLSISELADVISLIHLILLISLALVSIHAVLMRKLSLDILYEYFYGELNRFFYTYDSVAVSLLEKRNKYSKLITKVVTVMILSAGVTVVILEPFLKFLLGEDNARVDGLLYEIPIPMYWPIDHDNYLLSFILPTILELLLCGGTAAIISGAVLIYLNYCLHIFTQLKCLLHNIKTLDEHVKTSILQDFGDGVKISSSSSIALNYYNKFVKDIVQHHVLLIDCFRHVNNAGKWMFLALFLSGGIDIAMAGIMVFFIEGRLATKITAVFICFTEIVMFAAATFASDLLTNMSLEICEEIYMLNWYTMREEVKQSLRIIMTSSQRPLCFKAADIIPVDKEMFSS

>GbueOr50

MGWLERLSREDDPIVVEGIDKEFGKVLYYLGVFRSASKKNILGTLVFYLCITVLLMFFVSFFMITVYLSISELADIISLIHLTLLISLSLASIHAVSMKKLNIDILYRYFYGELNRFFYTYDSVAVSLLEKRNKYSKLITKVVTVMILSAGVTVVILEPLLKIILREDNARVDGLLYEIPIPMYWPIDHDNYLLSFVLPTLLELLLCGVAASIISGAVLIYLNYCLHIFIQLKCLLHNIKTLDEHVKMSIHQDFEGRMEFSSMSNVALNYYNKFIKDIVQHHVSLIDCFRHVNTAGKWIFLTLFLSGGIDIAMAGIMVFFIEGRLATKITAIFICFTEIVMFAAATFASDLLTNMSLEICEEIYMLNWYTMREEVKQSLAIIMTSSQRPLCFKAGDIVPVDKEMFSSMMQSAYSFINLIAAVKS

>GbueOr51C

MGLLDWFIEEDDVVVNEGMRREFGNLLYYFGVIRSTSKENIKGTLLFYFFIMFVFVSFDSIFLYTVYCALPVLSEVISTGHLVLLVFLALIVAYNVYTNKTLINKFYRHIFLDLIRFSYAYDPVIISFQNEMQTYTKWATKAVVFIIVTAGFFLVLLDPLLNIWFDEEVDKIDGVVYGIPIPIYWPLDHEDYLLGFILPSFLELICVVTVVGITSGSVCLYMNACLHIYFELKVLLHNITSIDQHVARSILEDTKGGKNIDISSLEALHYYNNFIADIVQHHIMLIKCFDLVNEIGKWILLTIFLSGGLDLAMAAIVVLFAEARVGSTVTAIYFSFMEIIIFGAITFASDLLTEMGLEIQTEIYMIKWYVMREEVKKSLMIIMTCSQRPLCIRAGNLIPVDREMFSS

>GbueOr52

MGLLHWFIKEDDPVANEGIRHEFGNLMYYFGIARSTSKENIRGTLVFYFCTVFIFTLFILIYLCTIYLALPILSEVIRVTHLVLLLFLALVVAHAVFSKKTLIEKLYRNFYLDLTPFSYTYDSAILSFHEQRERYVQWATKAMIFIITTGGIILMVLDPLLNLWFDEEVVQIEGVLYGIPIPVYWPLDHEDYFVGFVLPSFLEMICCVTVIGILSGAVCLYMSICFHIYFELKVLLHNIASIDQHVARSILEDTRGKRNMTISSLDYYNDFITDIVQHHIIIRNCFDLVNELGIWVLLTLLLSGGVDIAMAAIMILFVEARIGSMVTAVYFSFMEIVLFGAVTFVSDLLTEKGLEMQTKIYMTKWYVMREEVKKSLMIIMTCSQRPLCIRAGNLTPVDKEMFASILRTAYSFINLVAAVKSKQT

>GbueOr53

MGLLDWITEEDDPVASEGIRSEFGNMMYYTGIFRSTSKENIKGTLLFYACVMSLFAFFVLIVQYTVYLALPILSEVISMAHLVLLIFLALVVAYSFFSKKPSVDKLYRHFYSDLIRFSYSYDPVIISYHKKRETLIKWASKGVLFIITIGGIILMVLDPFLNLWFDEEVVQIEGVLYGIPIPIFWPLNHEDYFLGFLLPTFLEMACCVAVMGMATGAVCLYMSACLHIYFELKVLLHNIASIDQHVARSVLEDGKGRSNIETSYLEEVEYYNNFITDIVQHHIILHNCFKLVNDVGMWMLLTIFISGGIDIAMAAIMVLFVEARIGSLMTAVYFSFMEIIIFGAITLASDVLTEMGLEMQTEIYMIKWYLMREEVKKSLMIIMTCSQKPLCIRAGNLIPVNKEMFSSILQTAYSFINLIAAVK

>GbueOr54IC

MDFINWFTKEDDPVANEGIRHEFGNLMYYFGIVRSTSKENIKGTLLFYFCVMSVFAFFVLMLQYTIYLALPILSEVISMAHLVLLVFLALVVAYSFFTKNTLIEKLYRNFYLDLTPFSYTYDPVIISFHKQKERYVQWATKAVIFIITTGGIILMVLDPFLNLWFDEEVVQIKGVLYGIPIPIYWPLDHEDYFLGFLLPSFLEMACCVTVIGVTSGAVCLYMSICLHIYFELKVLLHNIASIDQHVARSILEDTKGKCNIETSSLEALKYYNKFITDIVQHHIILRNCFELVNEVGKWMLLTIFLSGGIDIAMAAIMVLFGLEMQTEIYMIKWYLMREEVKKSLMIIMTCSQRPLCIRAGNLIPVDKEMFSS

>GbueOr55C

MGLLDWFTEEDDPVASEGIRSEFGNMMYYLGIFRSTSKENIKGTLLFYAGVMFIFIFYIFLYLYTIYLALPILSEVISMAHLMLLICLTTVVTYSLFSRKTSVDKAYRHFYSDLVPFSYTYDPVITSYHRQREKLIKWASKGVLFIIMTGGIILMVLDPLINLWFDEEVVQIEGVLYGIPIPLYWPLDHEDLFLGFLLPTFLELVCSVAVVGITSGAVCLYMSACLHIYFELKVLLHNIASIDQHVARSVLEDRKGRSDVETSSLEYYNNFISDIVQHHAIIINCFDLVNDVGMWMLMGIFISGGIDIAMAAIMVLFVEARIGSLMTAVYFSLMEVFIFGVVTFASDLLTEM

>GbueOr56

MLSDISFTKSMGLLHRFIKEDDPVANEGIRHEFGNLMYYFGIVRSTSKENIKGTLVFYFCTMFIFTFFVLVYLSTIYFALPVLSEVINAIHLVLLLFLAYVAAYAVFSKKTLIEKLYRNFYLDLTPFSYTYDPVIISFHKQRERYVQWATKAMIFIITTGGIILMVLDPLLNLWFDEEVVQIEGVLYGIPIPIFWPLDHEDYFLGFFLPSFLEMACGVPVVGILTGAGCLYMSVCLHIYCELKVLLHNIASIDQHVARSILEDTKGKTNIETSSLEEVEYYNNFITDVVQHHIIISNCFDLMNEIGIWILLTLLLSGGVDIAMAAIMILFVEARIGSMVTAAYFSIMEIVLFGAITFVSDLLTEKGLEIQTEIYMIKWYLMREEVKKSLMIIMTCSQRPLCIRAGNLVPVDKEMFSSILQTAYSFINLVAAVKSKQT

>GbueOr57F

MGLSKWLASEEDVKTHAAVDSVFGSWISKSGFHLKYTEDSVLVNYARIIYFVMEGILHIIICISAIISVYPELGDTFNQIHILLLLFLGWFMIFIAVKNKKSLERVATLIHSLEINMKNEKDEKIKEMIKEKEQYKKIVNYAVPKIFVCGALLYISFGWIASFIDGQYYNYDLNLILLAPVVTYWPSMGGYIILQYILPNCLQVATIFVAAFVMGGGVTFYIITMIYIRFYLRFIKYRAENMDEICREINQQRKKEGIYEELRTTQSMFIKETVVLHITVKKCTRYFNQVFSMIMLCIFVTGGLDMGLAAIAVLFYEGNMAYKLEAVVLSLMELSIFGCMALSADHISHLSEEVSFSFYSANWFDWSQDVKKMMLIVMSCSQKPIIIEAGPVITVNKEMFSALLQSTYSVINLYIASRSN

>GbueOr58

MGLSKWLASEEDVKAHAAVDSVFGSWLSTSGFHLKYTEDSVLATYGRIIYYAMLITFHLISISCAIIIVYPELDDTINQIHVVLLIFLGWYILFVAVKYKKSIELVATLIYSMEINMENEKDEKIKEMIKEKDLYKKIADYSIPRLFINVGLLYVSFGWISSLIEGHFYNYDQNIIQFVPVNIYWPSMGGYIIPQYILPNFIQVTSIFITTLFFGGGVTFYIITMIYIRLNLRIIKHKAENMDEICREINQQRKKEGIFEELRTTQSRFIKETVLHHITVKKCTRYFNQVFSMIMLLIFVTGGLDMGLAAVLVLFYEGNMAYKLEAVSLAASELFIFACMALSADHISNLSEEVCFSFYSANWFDWSQDVKKMMLIVMSCSQNPIIIRAGPMITVNKEMFTALLQSTYSVINLYIASRSK

>GbueOr59

MGVSKWLASGEDEEIHSVVEKIYSRWLSTTGFYLKYSEDSVLSSYTKIIYYYLSGLIHLTLISHAIILVYPDLGDMFNQIHVLSLLLLCSSILLTSIRYKKGMGLIATLMLRLDIEFDSMTDEKIKSMKTEMEFYKRILIYFIPKAFVIAFCLYMAAGLTASLIDGHFYDGNILRLASVNTYWPSMGGYIVLEYILPNVLQGMSMFLSVEFMAGACIYYLVTMLYLQYYLRVIKHRLKNFDSISKEINRKRAEEGMVEELMMTQTRLIKEIIQHHKIIKTCCEHFNEAVSGVMQLIFLSGGIDMGLSVILVLFYEGNVAYKLEAIVLSMMELCIFACLCFTADHIGSLSEEISLSFYSAEWFGWSQDVKKMMIIVMSCTQKAIKFHAGPMFAIDIEMFASLLQSTYSVINLYIASKST

>GbueOr60

MGLSKWLASEEDVEIHRAVEKVYSRWLSKSGFYLIYSEDSVLANFAIIIYYALSGVIHFSFISYAIIIMFPDLGDMFNQIHVISLLLLSYSIILSAIRNKKNIEICAKLIHNLEIEFENIKDEHIINMKKEGECYKRIVVYNIPITFLAALILYVTAGIIASLIDGDVHDDTILRLAAVNTYWPAMGGYIIFEFILPNVLQIVTMFYSVLFMSGCFIFYLVTVIYFRHYLSAIKHRLMNLDAISREINKKRKEEGIVEELRITQAELIKEIVQYHLTIKKCSKHFDECISTIMQFIFLSGGIDLGMSAILVLFYEGNLAYKLEAIVLSFMELFIFTCICFSADHIGSLSEDVSFNFYSAEWFDWSPDVKKMMMIVMSCSQRPIKFNAGPMFTVNAEMFASLIQSTYTVINLYIASKST

>GbueOr61

MGLSRWLASEEDVETHAVVDSIFGRWLSTSGFHLKYTEDSVLANYAMIIYYAMAGLLHVTIITSAIIIVYPELGDTFNQMHILLLLFLSCYMLFAAVKYKKSIELVATLIYSMEINIENEKDENIKEMIREKELYKKIVNYAVPKMFVYAAFLYIFFGWIASFIDGQYYNYDQNLILLAPVNTYWPSMGGYIILQYILPNCFQVISIFITIFVMGGGVTFYIITMIYIRFHLRIIKHRAENMDEICREINQQRKKEGIFEELRTTQSRFIKETVLHHITVKKCTRYFNQVFSMTMLCIFVTGGLDMGLAAVLVLFYEGNMAYKLEAIVLSLMELLIFGCMALSADHISHLSEEVSFSFYSANWFDWSQDVKKMMLIVMSCSQKPIIIRAGPMITVNKEMFAALLQSTYSVVNLYIASRSK

>GbueOr62C

MWFLVNRYLDYLTANDSYDANQVVIKDLGKLRFVIPFYWDMNTRTKKWFRLIYFIFIVVGFFYFITMCIVSIFHAPNLTFVVKFFNLIFLFATAMFKSWSYFRSRRSISILYKYINDGFFIYNDFEEEKNFAEALEKFEKQLMKFGPITFYVITSLYMCYEPLSNYLEGNTESIINGINIKLPLPMWTPYDSSTDISFWITVALIVIYSYMCGCVVMTGTLAFALDGERIINQIEVLIFATDQLEARALRLWYLDMDRKNDQPPEKRDLNSIYEDKKFAAIYKECLKKLIIHHTIIIRSCRAFLDIFSIPISIVTLMGSLLLAISLFCLADDNTPITFKIISGYLVFMEIAIMGMMCWYGEMIASKFQEFRVAIYNTNWTYTDKEAKTVVQMIQIGLMREAKMKEGIFGVALDLRLFAN

>GbueOr63

MFSAVHRFLDWLTEFDDDQSLKMLWNDSGGYLGLICPFSWDLRRPARKRTLMVMIFVVTQLMSMEFMLIMKIIKSLDIGVDVFCETIGILVFNIIAFVPYPINLIVRRKLCPMVRMIKDCLRYDDFPDDTEKRIRSRIDQVNKFNRILCLPLYLIGVFMPVFISVDNNYREIDAFPDWFDKRLPIPMWLPFSTENSVVFVLAVGYMVLNDILVCQALYGLCNLNFNFCMRVVIELELLIEALNKQEERSYNIHSSMISKRNDLDKGQNQASDITDKDKSSHTNNKESCTTSDPHKTEMINNKTVIDNNDTIKNIYIPLSDKFETGNNILPKESQIEKIINKSAKKPKKAKINNIGSVPEQNITRSYSKRKLSHSVTQVLQDPTLIRLYELYGNLNYRKCYHTCFKKTIKHHKLMIEINNRFFEIAGLIYFFVLYISGFLMASTIYSAFDDRRTIIFRLFSVALTVNVFVFTSSLCVVGEKIKSSFSDIRFAIYSTQWTDADESIKRTIRIFLMRSQNPEELKCGIIKTSLNHSFIFSMLRMCYSFVNLIRLLKKNGIIE

>GbueOr64

MQSDKSYNLKLLLGLVGILKPSSLSPILWNMYQATFFLIVFIYLPLVAMGAFTSNNLLKIAQELAFFISGSHISFIAIMIYFNRENILETFGKLEVLLTEQRKNHVHKAFIDQAYYSFSIFFKGYLLTYVFFGVATYPLPFLPSVTNTDRLLFPAWFPWDIRNLTNYIITIGLQTILLSILVVVIIGQYPLAVFMVYHIDAQINILMSDLERLDGSSRQRLIKCIKHHQFILRMVGVFKQAFSLGFIFDISLAPIQTCVANITSVLMDDPIFYFYVSYFILLICQPLFVSWCGQMLTSSSERIGYSLLHSSWYQTYSKEYRKDLIIFFTMLKRPCYFDVWRVFVLDLKSYSTFSSAAYSYFTILK

>GbueOr65

MILSSSSKYLDLSSKLEKRERMISLPIEGFFTLPKCCSQDELGNMHSDKSYHLKLLLGLVGILKPSSLSPILWKMYRGSVFLIVLIYLPLVAMGAFTSKNLLKMTQELAFFISGSHISFIAIMIYLNRENILETFGKLEILLTEQRKNNVHKAFIDQAHYSFSIFFKWYLLTYVFFGIVTYPLPFLPSVNNTDRLLFPAWFPWDIRNLTNYLITIGLQTILLSILVVVIIGQYPLAVFMVYHIDAQINILMSDLTRLDGSSRQQLIECIKHHQFILRMVGVFKQAFSLGFIFDISLAPIQTCAAVYNITSVPMDDPIFYFYISYFLIAISQPLFVSWCGQMLTSSSERIGYSLLHSSWYQTYSKEYRKDLIIFFTMLKKPCYFDVWRVFVLDLKSYSTFSSATYSYFTLLKVIEQKKAFEIN

>GbueOr66

MEMEKSFETIRFLMGFIGLIKPDRISQIYWNIYRLLLLLSGVTFIPLAFAEAIRSKNLLHLTASTVYITSALHNYFNSIRFYMKREAVLKILDGLEEISFKQRNDPDSMKIINNACSSSFWFIKYYLYSFGLFSFCILPILFFPSKSNENNLVFPALFPWNLDNKILYVISITIQFYIVGCMIAMLISEYPFVVFVVNNIEAQINILKGKLQRLDGSSRPELINCIRHHQVILRLVRQLNKLSLGFAFEVSIAPFQTCLAGYNTSKVGLDDPMFYNHMMYFVLTLIQPLYICWCSQKLKNSSESIGDCLFTSRWHQKYSKEYRKDLLFIFTMLKESCTLSVWKVFILDYKTYSHFLSAAYSYFTVLKVIEKKKGI

>GbueOr67

MEMEKSFETMRYLMGSIGLIKPDRISQIYWNIYRLLLLLSGVTFIPLAFAEAIRSKNLLHLTASTVYITSALHNYFNSIRFYMKREAVLKILDGLEDISFQQRNDPDSIKIVNNACSSSFWFIKYYLYSFLLFSFCILPILFFPSKSNENNLVFPALFPWNLDNKVFYLISITIQFYILGCILAMLISQYPFVVFVVNNIEAQINILKGKLQRLDGSSRPELIKCIQHHQVILRLVRQLNKLTLGFAFEVSIAPFQTCLAGYNTSKVGLDDPMFYNHMMYFVLSLVQPLFTCWCGQKLKHSSESIGDSLFTSRWHQKYSKEYRKDLLFIFTMLKESCTLSVWKVFILDYKTYSHFLSASYSYFTLLKVMEKKKGI

>GbueOr68C

MMGFASSIIFIASDTNDEITDTCIAASVVLLCTLSNFKMYSYKMWKKKIDSFFDYFEQIKNDRMFAKTVSLMKYYFIMVAFSLSMWNIAPFFSGSGKFPTPLWFPFKLEGFNYTIAYLFHICLSFTVCGTQTALDMVFVLMASSIFVRVRYLNNELKRVDKKDQKVNWNLNKNPVKFDKLRYQLNLHKECILETSALGKISNYIFLLQIAQTVFMACFLIYALSEVKAQSDLIIVMMCIFATFLELFCFCWIGQIVSNEFEEVHYNLFWRLEEDNLSLKQMKSIHIAQSLTVKTKNIEASLFTLSLHTFMM

>GbueOr69I

MFKVVKILLKLTTVTPFSREPNYVYLVRSIYTYLFLFLSIVLCAKFALDNKHITERCIAFTFAMSSLNGIVKVTVERYNRRNIDEIIDVIKRETKGGFAETTRKIARFYFNMILIIVATWNVYPLIDNKKRSTIPIWFPFQIENTLNYCIGYVFVLGIFFYQSTIHFSTECVVMLAVAEISTSVDTLKEQAEEFGERLHYKKLSEDYIIKPDPELKRVINLFFKKHIEIIRLTNIVTGMTKIIFLVQVVQSILMSCTFAYAVSKAIRESLSYYMFLRTVTKN

>GbueOr70C

MALYFTSHLSKCMKWIKIIHIFILKFTGLLLCDFENEFTAGLRTAAHILWMLFCFYIVIMNLRSDHLSVTDKIICLVNILTNISGIIKLVVFSKGKHRLKKVTDEVFHPYWNVRNSYFLLLVMLYYQSAFFVIILWGLAPLILGIDLLPFLKWIPFQPESDTHKIFKFIFAVILIIMIIVTNLSGDVSYMLIASLICEKAKMARKVLGEFSEQKTKSSVDATIRSFVKSHVDILRTVSLVENYMNILFLHQVVATTLTVCLITLSLDEAEGLFDVVKLSVALVVVLFELSLFCIFGQSIHDELELLHTTTFWTDWYTHSLAQKKSILICQAYTCREVWLKGGAIIKVSLRTLLA

>GbueOr71

MPRMEDSIFLKYRKILRFMGVTIPDHFTKRGILIQLYYFGVIFTMSMNALCLFISIFTRGVPMSDRSISLVCFLMCGSAVFKLIILQTRSYELLYLFDALNTPGAARSTWVLRKTNTISSIYTSCIIVLASGWCAYPFVVKKLKLPIPYWLPFVTKTVQQRMGVFMFVTSTMLFACGTHESTDTLLLMIAAQICRRFDSVNDTLQTLGEYQPLAKGDFGSEKVVERSAMGLMIVKDATMSRDETLLKECIREHVDVIKLVRHFDEVLDIIFFVQVIQSTLVSVMTFFAIFQVDNLYDELPKFVTIIMATYLQFYMYCWFGEEISFHSDDLHKAVYISKWYKCNRRVMKSIVVMETFTKTHFKLEGGHLFQMSLHTFLLVLEQSFSYLMVLRAVTRKTNQMALDAAEEARLAALLY

>GbueOr72I

MNVKAMIDFAFVILTIREGLPKRIKTFAFLFVLSCKISGIYFLVMNIRHRFQDLNDASMSVVFASMFFVTTIKYLVFVKYNHHVVLKLDQVFSKEMSKFSDLTINICKLFFSMGIGANIVWNLIPFFDNELHITLPYYLPFQMNNYVFSIFYVFACITAALISFIQVPTDCAYMLLSARIYDDLGIVKNKLRNLHESVITNEIDSTTTQLRFSQIVILLIEAIKNDLNVNTLKAFICLSWAMYQLFWICWFGQKITDKFEALGNELLWSGWEGCSVSHQRCVLIAQAMFSQPMVITGASLFQVSFKTLMWMVVNKSLSVAFILYSS

>GbueOr73FI

MYKDIKFKNVMDFSFKILNIKDDRSFFLKIFYWLTIFVQVLGVISLCLHIYFRSSKLSDTSIAVVFICSGIVTLVKLITFIKYRNYVSYRIDLVFENEPSKFTKTTQMICKGFILMGISGNVLWTLIPLIEMKMGLPTPYFIPYGNTNIVSYVLSYLYSTVTVSLIFFIQVPLDSAYMLMSAGICDEVDFVVGKIQNLRFYVYNNSRKAKIVQLKLYEVIILHRSVLTEFKSLAKIMDYIVLFQILQSIIISCFMGFTLTQAMSESLSYFMFLRTVSTNRQKFIDNDLDI

>GbueOr74FI

MYKDIEFKNIMDFSFKILNINDERSFFLKILYWLIISFEVFGVISLSLHIYYKSTKLSDTSIAVVFICSGSVSIVKLITFTKYRNYVSYRIDQVFQNEPSKFTKSTQMICKGFFFMGISGNVLWALVPLMEMKMRLPTPYFIPYGDSNIVTYVLSYLYSTVTVSLMSVIQVPLDSAYMLMSAAICDEVDYVVRKMQQLRFYVHNNSRQTKIIQIKLYEIIILHRSVMTEFKSLAQIMDYIVLFQILQFIIISCFMGFTLTQFESLRPELFWSGWQHCSLKHQKSILIAQEMLNKPMIITGGVFKVSLNTFQWAMSESLSYFMFLRTVSTNRQKLIDNVLDI

>GbueOr75I

MELKVIIDFILSVLSVRDKFHKLIQFMLYHSILFILWSSAVSLMVRIIHNNNTLGDMGTSVAFIIGFIASTNKLNTLIRHKDYIGVTLDKIFLKSSTFSKWTQTICKAFFILAFLANVSWSVIVPLLENMRFPTPYYVPFYRENKYVLGAVYVYGVISTALLSIVQIPFDSIFMLLTAGICDNVDKVIDEIRQLKQYINESDKNSKQINLKLIGIVIHQRHCISDFKMLADLMDSIVCFQILQSIAISCFMSFVLSQFEALGHEIFWSGWEECSVHHQKLILIAQAMFSQPMVITGASIFQVSLKTLMWSMKQSVSVYMFLRTVEQNRSKF

>GbueOr76

MKYRDFINFALILITVRKHFSTTIDNLIFTIVLLIPVICGILCVEFIMYSSPTMTDLSLAIVVGCATLNSIFKLCIFRMNRIQVSEYIEDIFSTKPSKSTQVTINFTKLFIMGAFACDFFWDIGPFFGKELTLPMTYYIPLAYKNVAVYIVSFIFCVLSLQLMTLMQMLLDSTFMLMSARICDQADEIISNIRMLRKYLLKNTTNNSNIHQKLSEIIELHNNCLRDFRIVANIMDLIVLFQIVQAILFSCFLTLTLSETDSSGDIARCSVVFLINFYQLFGMCFFGQKMTDKLELIHYELFWCGWEDFLLSDKKSILIAMSSTVKPMIIQAGSIFDVSFETFMWVMEQSVSFYMFMRTVAIIEKK

>GbueOr77P

MNLMFIILIIREYFSRWLEKLLFWFWVVVLLKSAFFAGQFIRYNRYKWRTRVLLLVSLZLZZNFAMKSWIEWMTCSLFTTHFQKILZKYSKLVELLIYSLAILIFIWNILPIIKGEKILPSPHYIPLANSSETMYVICYTFSSSALSFAAIAQVSADAIYMLFASNLIVKTTKLLNIINQHKTIVEQTGKDYHNIRKINSIHNQHKCLKZVIRFNYVNKANNLFLYLKITYEDNKVSCFZLISLXLEIIHYELFWCGWEEYSLSHQKSILITMSYSVKPMILQGGXIFDISYETYMWVMEKSLZFYMFLRNVANVEK

>GbueOr78I

MKSLKDTMNIIFIGLTIREYFPRRFEKFLFRFWLGLLIVHAFLAGQFIRYNTAKLADASIAVVIMCAALVSITKYVTLVKYRFEIMDKNDEIFTVQDKFSEMTIKLAGLFVYSLLGFVVMWNMLPILQGEKILPSPHYIPLANSSETMYILCYTFSVSALSIAAISQVAADSIYMLLASNLIVKTSKLLNTVKQHRAIVEHHEKDKEIISHFISIHNHHIECLAETSGDVIKSVGGIVLGFYQLFSICFFGQKITDELEKIHHELFWCRWDLCSLSHKKSILIAMGFTVKPMELKGGSLYRISYKTFMWAMRESLSFYMFLRTVSNRGKVT

>GbueOr79

MKSLKNTINLMFIVLIVREYFPRWFKKFLFWFWLGLLSASAYFAGQFIRYNRSKIIDSSLAVVVLCTALVSITNYVTLVRNRAEILGKMDVIFNVDDKFSEMTIKLVEMFIYSAFFVIIFWNMLPIIQDNQILPSPNYIPLANSSQTMYIFSNMFSCSAICITALAQISADSIYMLLASNLIVKASKLLKIIKKHKIIVEQDGKDDEIIKTFISMHNQHTDCLKDFELLGKMMDPIFLTHVLQSIVLSCFLAFCFTKGEATGDIVKTFGILILEFYHLFIVCFFGQKITDKLELIHNELFWSGWEDCLLSHKKSILIAMSYNVKPMILQGGSIFDISYETFMWTMEQSLSFYMFLRTVANIEKK

>GbueOr80

MKSLKNTINLMFIVLIVREYFPRWLEKFLFWFWLGLLSASAYFAGQFIRYNRSKMIDSSIAVVILCTALVAITKYVTLVRFRAEILGKMDEIFHVEDKFSEITIKLVEMFIYSVIFVMILWNMLPILQGNHILPSPHYIPLANSSLTVYIFSYMFSCLAICITALAQINADGIYMLLASNLIVKASKLLKIIKKHKIIVEQDGKDDEIIKTFISMHNQHTDCLKDFELLGKMMDPIFLMQVLQSIVLSCFTAFCFTMTEETGEIIKSALCLVLGFYQLFIVCFFGQKITDKLELIHYELLWSGWEDCSLFHKKSILITMSYSVKPMILQGGSIFDISYETFMWTMEQSLSFYMFLRTVANIEKI

>GbueOr81F

MKSVKRIMNLLFIILTIREYFPRWLEKLLFWFWLGIPLISAFFAGQFIRYNRLNMADSSIAVVIICGALVSLTKFVTLVKFRSEILDKMDDMFTIYNKFSENTLKLVELFIYSLSILIFMWNMLPILKGEKTLPSPHYIPLANSSQTMYIICYTFSATALSFAAIAQVSADAIYMLLASNLIVKTTKLLNFINQNITIVEQTGKDDDNIQKIIAIHNQHNECLKDFELIGRMMDPIFLTQVLQSIILSCFLAFCFTKTEETGEIIKSALCLVLGFYQLFIICFFGQKITDGLALIQNELFWSGWEKQSLSHQKSMHITMSYSVKPMILQGGSIFDISYETYMWAMEKSVSFYMFLRTVANIEKTIE

>GbueOr82FC

MKSLKHIINLRFIVLAIRDNFSPWLQKFLNWFWLGLLFASGCFAGQLICYNYNRSKITESSVAVVVICGALVSISKYVTLVKFRSEILDKMDEIFTVHDKFTEMTIKLVELFLYSLGFILVIWIMVPLFQGGKMFPLPYHIPIANTSQIMYILSYAYCSLTLIIYCMANLSADGIYMLLASNLIVKTSKLLKIIKQQKTIVKSTGKDDEIKRHFISIHNHHTECLKDFELLSKIMDPIFLVQITHSIISSCFMAFSFTKADETGDVIKLVGILLLGFYQLFSICFFGQKITDTLELVHYELFWSGWEDCSPSHKKSILITMSYSVKPMILQGGSIFDISHETFMW

>GbueOr83FIC

MFNLSFIGENKLKNIKYTDMLKFIFIIFTVRPYFSKIKEKLLYAIILNLQVLSLLLCIQYIIYHSSKISELSMPIIMFCAYGNAIVKLTTYVKYRKNVIGPMDEIFTSKPTRFTQMTINFSKLLIWGSLACNWFWNIAPFFSKELTLQVPFHIPLAYKSKMWYTFFYIFCVISVLVMAAAQIYLDSLYMLLSARICNKGDTIISKIKALRKYILQDNNTNNRDHIDRKMSEIIKIHSQCVNESSGEIMRCFIVLVVTFYQLFVICFFGQTITNKLELIHYELFWSRWDLCSIKHRKSILITLGYTVKPLVITGGSLYQVSFNTFMW

>GbueOr84I

MKYRDFINFGLIVISVRKYFSKTIDKFLFTIVLFIQVISLILSVEFLMYSSPTMSEISMDIVVSCAISNAIIKLCIFIKNRNQVIEYLDEIFSTKPSKFTQMTINFTKLLITGSFVCNVFWNIGPFFGNELTLPMTYYIPLAYKSKGLFIFFFIFCVVSLLGMTVIQIFLDSTFMLMSGRICDQADEIIYNIRMLRKYLLKNTTNNYNIHRKLSEIVELHNDCVRDFRILAKIMDPIFLFQIIQSIMLACFLTLSLSQLESIHYELFWSRWDLCSIKYKKCIVITMSFTFKPLVIKGGSLYELSYKTFLWAMRESLSFYMFLRTVANRDKK

>GbueOr85

MFNIMNILKAFRAEESKESIKEGYDRSKGYVMRLGGLYPNLNGGIQNLTILHSFTIFGLMSFFVFSFLKTATLTNNSFVFNEVIHFSVIIFLPLSCTVNTLLHRRDINAMFEIIGNDFFKYKDNIWDSVSALNRNRQLVKYFLAGWGLFVGGAVFLNDICYLLSDVFKQNYDKVVDGVHYNMLIPKWSPVRIVEYIGIPMELCGQAFFIFGITAIDAPFVAITVELYMQFCELKKSVKYIRERTLAKFDKLKINKNKMANLKIMEDEFMADCFLECLKENVQHHQIIIKCFNHLQNWISFQLMTVYSAGAILICFSALTITEKNVDLSLLISTLMILLAEVVHVFVYCALGELIIESSTDVREELYFTDWYKFKPRVKTTMLIMQTRLFHPLKINFAGLIWASLSSFSQVLSSSYSYFNLVRATREQ

>GbueOr86

MRTLHLLLSKMDDYDKVDKTTRDQGVEIGIIKAVYRSLVGVYFLPLGNKLEKAVAVYGIINRILLSSVATYTMLINVDFLTIFFESAFVLIAIVGIELSILIAVIYLESLRRMLYVVGTDCYVYENEATTAREIKDNNQFNIYIKKVTKIYNGYLFFSIGFYVVSTPFIQSDILGNYNHNLPLMLYIPFETDESYIGYFIATVFIFTAAWFTCMGVVALDLGCYTMLLHLEHQLKILNGKVKCLEVRAMRRMGIKESAINKYKGRLWNNKKFNQNMWLCLRETIRHHHKIIRFYKLIQKSHGNNYLIVMSNGSLLIAMAGYLLTGNLDNSIVVKAFGAVIATLIHMGMYCMFGQRSLELMENLFDSPWYHCDEHFKKSLLIMMSMAHNPLKLKSNLLKHPLTLETFNDVSYVVSTAYSFLNLIRQM

>GbueOr87C

MDDYDKVDKATRDRGVKIGIIKAAYRSLEGVYFLPLGNKLDKAVAVYGIIIMVSICCLAMYTMLINIHFLTIFIESAFVWIAVAGVELSILNGLIYLENVSRMLYVVGTDCYVYENEATTGREINGDIKFNIYIRKLTKIYKGYFIFTVGSYTTLTPFIQSDDILGNYNHNLPLMVYLPFETDESYIGYFIATVFIFTAAWFMYVGVVGLDLGCYMMLLHLEHQLRILNGKVKCLEVRAMRRMGIKESAINKYKGRLWNNNKFNQNMWLCLRENIRHHQSIIRFYKLIQKTHNNNYLINVDISIVVKAGVTVLSMIIHIGMYCMFGQRSLELIENLYDSPWYHCDAKFKKSLLIMMAMAHKPLELKSNFLQHPLTLKTFN

>GbueOr88C

MIKDYENDKTTAANRRRIKKTFDLSLMDDYDKIDKATRDRVVKIGFKKAMYHGLVGLHFLPLGSKLEKVVAVYGNIVMISICCVATYTMLINVEFLNIFSEASFLLVALAGIELSVLNSLVNLKNVSRMLFVVGTDCYVYENEETTASEINDNIQFNTYITKLTKIYKGLMIFTVGSQLILPPFMQSNTLGTYNKNLPLMLYTPIETEESYVGYFIVMAFYLAGGLFTYIGAIALDLGCCLMLLHLEHQLRILNGKVKCLEVRAMRRMGIKESAINKYKGRLWNNKEFNQNMWLCLRENIRHHQNIIRFYNLIQISHNNNYLITMGNGSLLVAMTGYLLTGNPEIALAVKAFVMLFTVLIHIGLYCMYGQR

>GbueOr89PC

MDDYAKVDKTTRDRGVEIGIIKAVYRSLVGVYFLPLGNKLEKAVAVYGIINRILLSSVATYTMLINVDFLTIFFESAFVLIAIVGIELSILIAVIYLESLRRMLYVVGTDCYVYENEATTAREIKDNNQFNIYIKKVTKIYNGYLFFSIGFYVVSTPFIQSGILGNYNHNLPLMLYMPFETDESYIGYFIATVFIFTAAMFTCMGVVALDLGCYTMLLHLEHQLRILNGIVKCLEVRAMRRMGIKZSAINKYKVRLWNNKEFNQNMWLCLRENIRHHQNIIRFYKLIKKKHNNNYLIVMSNGSLLIAMAGYLCTGNLDNSIVVKAFGAVMATLTHMGMYCMFGQRVTDQVLHY

>GbueOr90NI

MINKIFEDLTSEDSVHVFGLLYKEYWYLLQISGYFITKPIHILFLFINICILLHHTVLFSMSIYYTYPDIFVVSDMILFTNIVSAVLIILISVIIKRKHFIRCIKLTLSDGYFDYGPTTSIDRNKFFEPFSKQIYKFKVLYPIFLVVSSIAIFISVPVLDFFTGHLFVGGCVLIVDLPCIMMFQHISTYLIAELSLLKQTLNDLDGRASRLYLTKHGKKPTNMTEHDYQQCYYDCFKQNIVHFHKINTLFNELQELCSVAVFLFFLISAILCSTAAVGIISSCEVREAIYEINWYDASKRLKTSLLLMQVTTLRPLILTGLGLIPADYHTFSTIMNTSFSYLNLMLAAQD

>GbueOr91NI

LLHHVVLFYITMYHVYPDIFVVSDMILYTSMGSAVAVILISIIIKRKHFVRCIKLSLVDGFFDYGPTINVDKNTFFEPFLKQIYRFKVIFPTIIVISAIPIFFSVPILDFFTGHETEESHNPNINTRALFTIWYPFRTDEGSLFIFVNILELLVGSSIFIIDLSVLLVLDRLSTHIVWELLLLKETLNDLNGRASRLYLLKHGKKPRNTTGHDYEQCYYDCFKQNIIHFHKINTLFNEMQELYSWPVFVFFLICAILCSTAAVGIVNSSEIREAIYEVRWYDASKRFKTSLLIMQVTTQRPLTLTGLGLIAADYVTFSTILNTTFSYLNLLLAAQE

>GbueOr92FI

MFSKIFQDLTSEDSDEVFDIIHKEYWYMLQISGFFITKPIQILLPIINIFLLVYHIVLFSMSIYYVYPDIFVMSDMLLYTLMGIAVLIVLISIMLKRKHFVRCIKLTLVDGFYDYGPSINFDRNKFFEPFYKQINRFKTDEGSLFIFVNILELLVGSSIFIIDLSVLLVLDRLSTHIVWELLLLKETLNDLNGRASRLYLLKHGKKPRNTTGRDYEECYYDCFKQNIIHFHKINTLFNEMQEVCSWAVLVFFLVSAILCSTTAIGIINPDTRIGVQVKSVILYILLIFTCGYICYKGEYIKSLSSDIREAIYETSWYDSSKRLKTSVLIMQVTTLRPLTLTGLGMIAADYNTFSTIMQTSFSYLNLLLAAQD

>GbueOr93I

MGFKEIISDITSEDEDELYDFFHREYWYFLQMSGLFINPSKPVFFIFPIIHLYLIVHHMILCVISPIHVYPDIFLVFDAVQYFILLFGTLVSFIVITLNRKHLAICQKALLHGIHEYEPSFIFDKNKFFEPWIKEIRFFKGAYPLAVGIGSFVIVTSESLLDFLTGYKKQTYDNPNVNTRSLIIIWYPFRTDEGILFYIVNALLVSTGATMFLTVVPAIILYNQITTNICAELSFLMLSIKKLRERTSSLYLSKHGKKHRNITDSDYEDCLYSCFKQNIEHFHKIIQFFKTAQNIFSLVLLLFFTSSALLLATAGIGLVNCENARHVLYDFDWYNYSSKRLTDAILMMQMSTLKPLTISGAGLIDADYSTFTKVINTSFSYLNLLLASKR

>GbueOr94

MGFKEIIWDMTSEDEDKLFDVFHREFWYFLQISGIYVNPSKPIFLIFPFIHIYLMVHHIILCVISPIHVYPDIFLVFDAVQYFILLFGTLVSFIVITLNRKHLAICQKALLHGIHEYEPSFIFDKNKFFEPWIKEIRFFKVAYAVAVGIGLIVILTSEFLLDFLTGYKKQSYDNPYINTRALIIIWYPFRTDEGIMLYIVNALLVSAGATVFLTIVPVIILYNQITTNICAELSFLMLSIKKLRERTSSLYLSKYGKKHRNITDSDYEDCLYSCFKQNIEHFHKIIQSFKTVQSLCSLTLLFCFLTCALLFSTAGIGLVSGQTRIGVQIKSAFIYILEVGLVAFLCWKGEQVRSMSELTRHVLYDFDWYNYSSKRLTDAILMMQMSTLKPLTISGGGLIAADYSTFTKIINTSFSYMNLLLASK

>GbueOr95F

MGLKKIISDMTSEDEDELYDILHREFWYFIKISGIYVNPSKPILLIFPFINFYMLVHHIILAVFTSIHVYPDLFVVFDCLQCLMLLLSILFALIMLVLNRKNLVISQKQILRGLYEYGPTLNVERKKFFAPWIKKTSFFKFVLPAVVGIGQIVVFTSESISDFLTGYKRHTYDNPHINTRAVIIVWYPFRTDEGIIFYIVNALQVLTGASVFLVGVQGTIIYIQLTNNICAELSFLMLSIKKLRERASSLYLTKYGKKHRNITDTDYEDCLYSCFTQNTEHFHRILKFFKAVQTLYSLILLIIFLSCAFLFATAGIGLLSDQTRIGFQIKSGITCIVEVGVVATICWKGEQVRSMSELTRQVLYDFDWYNYSSKRLTGAILMMQMSTLKPLTLSGAGLIAADYSTFTKIMNSSFSYFNLLQASK

>GbueOr96FIP

MGFKEIIWDMTSEDEDKLYDVLHREFWYFLQISGIYVNPSKPIFLIFPFIYFYLMVHHIILAVFTMIQVYPDIFVVFDCVQYFTLLLGILFGLIMLILNRKNLVICQKQILRGLYEYGPTLNVERKKILRSEDKTNTFFXGIMLYIVNALLVSAGATVFLTIAPVIILYNQITTNICAELSFLMLSIKKLRERTSSLYLSKYGKKHRNITDSDYEDCLYSCFKQNIEHFHKIIQFFKTVQSLCSLILLSIFLSCALLFSTAGIGLVSDQTRICVQIKSAFICILEVGVVALLCWKGEQVRSDMSELTRHVLYDFDWYNYSSKRLTDAILMMQMSTLKPLTISGAGLIAADYKTFTKVINTSFSYMNLLLASK

>GbueOr97aJI

MWLKELISDLTSEDEDKLYGYIHKEFWYFLQLSGLYIDPSKPIFFICHFLHIVIHSIHLTQFVITSIKVYPEIFVICDCMNFFFLAVTTFSIMIFFNMNRKYIAQSQKAMLYEYYDYGPTFSVDRKKFFQPWTKAMHFFKTDEGIIFYFVNAFQLCHGASVCLIAVPAITIYHQLTTNISAELSFLMLSMNKIRERTSSLYLSKYGKKHRNITDSDYEDCLYSCFKQNIEHFHKIIQLHAATQNISSVCLFLFFFASALLFSTAGIGLVNDDTRIGVQVKSGILSLVEIGVLAVLCWKGEQVRALSEDTRDVIYEIDWYNASKKFNSSMLIMQMSTLKPFSISGVGLVTADYSTFSTIINTSFSYLNLLLASK

>GbueOr97bJI

MWLKELISDMTSEDEDKLYGYIHEEFWYFLQLAGFYIDPSKPIFFIFPLLHIVIHSIHLTQLLITSIKVYPEIFVMCDCINFFFLAVTSLCIFVFLNLHRKSMAQSQKAMLYDYYDYGPTFSVDRKQFFQPWKRALRFFKTDEGIIFYFVNAFQLCHGASVCLIAVPAITIYHQLTTNISAELSFLMLSMNKIRERTSSLYLSKYGKKHRNITDSDYEDCLYSCFKQNIEHFHKIIQLHAATQNISSVCLFLFFFASALLFSTAGIGLVNDDTRIGVQVKSGILSLVEIGVLAVLCWKGEQVRALSEDTRDVIYEIDWYNASKKFNSSMLIMQMSTLKPFSISGVGLVTADYSTFSTIINTSFSYLNLLLASK

>GbueOr98F

MKHLHFIPKDKKVKYFRYCGLFLKICGFLSGSNTRLSFITPILGIIILSGALHFGIFVYLEHNIIEVSDIAEYAHLSQLLFLLFTFLVFMHINKEDLIDIYHSTLKGLYRYPWGNWTYEINIRTGAEREIKLFYSIYPTAVLIACIQEILRHAYDPSRQPNEPCFKGWVPLPVDSRIVTSYQILMMFSTSLVSTTYFTFVASIMERLTSEIEVLKEVFRDPNYIAINTNYTIKQILKCCVEHQLIIYKYSDMAHNVLGRSLLLAFLNGSILLCTMGSLIPDSNLPIGIKLAVGVILAAELLAVACICYYGQKIYDSSDGLCFSIYQMNWYELKPSERKILITIMIRSQRKLYITGGGIKNYNFQTFAEILTTAYSYMGMLRTIRQP

>GbueOr99

MVLLCSTWRGHCPCPQIFSTFFVVLQVLYSKKYLRKVKYFRHSGLFLKICGLMSGSNSRLSFLTPILGIIIISGVLHFGIFVYLEHNIIEMSDIAQYAHLSQLFFLLFTFLALMHINKENLIDIYHSTSKGLYRYPWGNWTHEINIRTGAEKEIKLFYSIYPTAVVISSTHEMLRHACDPSRQPNEPWFKGWVPLPVDSRILTAYQILMIFSISLASTTYFTYVASVMERLTSEIEVLKSVLRDPHYIAINTNYPIQQILKYCVQHQQIIYKYSDMTFNVLGGSLLFAFVNGSILLCTMGSLIPDSHLPLGIKLAVGVILSAELLAVACICYFGQKMYDSSEDLCFSIYQMRWYDLKPCERKMVITIMIRSQRKLHLSGGGIKDYNFETFSEILTTAYSYMGMLRAVKQP

>GbueOr100P

KKVKYFRHCGWFLKICGLMSGSNTRLSFITPILGIIILSGALHFEIFVYLEYKTIELSDIIQYSHLSQLFFLIFTFLTFMHINKEDLIDIYYSTLKGLYRYPWGNWTHEINIRNGAENKIKLFYSIYPLAICIGCSHEIFRHAYKPSRQPNDPWFKGWFPIPADSRIISAYQFXFIAGVVSTAYFTYIALVMERLTSEIEVLKWVLRDPHYIAINTNYTIKEILKSCVQHQQIIYKFADMTVNFLEVSLLIAFVNRSILLCTIGSVITVSVSNLPIGIKLAVGVMLSAELLAVASVCYYGQKIYDSSDDLCFSIYQMRWYDLKPRERKMAITVMIRSQRKLHLSGGGIKDYNFETFSEILTTAYSYMGMLRAIRQP

>GbueOr101NI

KSVKYFKLSGIFLKMCGLLSDSNNFSTYISSFYIISLASAIFHFGILVYLEFSSMELMDIIEYSHLCQLYMLLISVLTFMYIEKKDLIDIYLNTSAGLYTYPWGKWTKEINIRTETERKVKLFFLFYPTISLMGATQQLARHANDPARLPSDPWFKGWVPFPVDSRIVTLYQILITVTAYLSSSVYFTYVAAVMGRLAGEMGILKTVLRDPHYIAINTKYTIEEILKCCVQHQHIIYDNSVPTGVKVAIGTLLISEVFASFCVCYYGQKIYDSSDDLCFIIYQMRWYDLKPHERKILIIIMIRSQRKLILTGGGIKIFNLQAFTEILTTSYSYMSMLRAVRQS

>GbueOr102NI

KKVKYFRLSGLFLRICGLMSDSNKFTPYITSFHLTIMGTSIFHFGVVVYKQISSMDLIDVVEYSHLCQLFFLLFSILTFMHIKKKIVVHVYLSTLKGLYRYSWGNWTNQINIKTEAEKGIKLSFSLYISITLIGTLQQLARHCFDPTRTSTDPWFKGWVPFQINSKIVTIYQICVTFGGFLSSSVYFTYIAVVIERLVGEMEILKSVLRDPHYISINTKLTINQILKCCVQHQHIIYKYFESVNNILKMGLLSVFINLTLLLSTMGLLIMSEDLCFTIYQMRWYDLKPYDRKILVIILISSQKHLILKGGGFVTFNLEAFREILTSAYSYMSMLRAIRQ

>GbueOr103NC

SKVDNFRYCGLFLKMCGLLSVSNKPLSLITQIQCILIASGSLHFGIFVYLECRTMELPDLIEYFHLCQLFFLLFTMLAFMYIKKTYLIDTYLSTLKGLYRYPWGHWTDEINIRFEAEKRIKLFYLIFFLPASLGSIQQMVRHANDPSRQPNDPWFKGWVPIPVDSKIVTVYQITVTLTGCIACTSYFTYIATVIERLTSEIEVLKSILRDPNYIAVNTDYTINQILKCCVQHQQIIFKYANMIQNVLSASLLSCFVNGTLLLCSMGLLETDESVPFGTKIAIGIMCMSELIAVVFVCYYGQKIYDSSGDLCFSIYQMKWYDMKPEERKLLITIMITCQRGLYLTGGGIKVYNFETLRE

>GbueOr104FC

MDANFSAQRKVKYFKYCGLFLKSCGLLNGSNTWLSFIALILSIIGVSGSLHFGIFVYLEYKTIELSDIIQYSHMSQLFFLISSFLAVTYFKKEDVIDIYLSTLKGLYRYPWGNWTYEINIRTGAENGIKLFYSIYPPVAFIGGIQIILRHACDPSRKSNDPLLNGWAPIPLDSRILTVYQITMIFSVDLICITYYTYVTVVVERLTSEIEVLKSILRDPHYIVINTNYTMKQILKCCVEHQQIIYKYADTTFNVLNFCLLFLFINGSFLLCTLGLQLTESNIPIGIQLGIGVMFLAELLSLGCLCYYGQKMYDSSGDICFSIYQMRWYDLKPSERKMVITIMIRSQRKLHLSGGGVKDYNFKTFSE

>GbueOr105FIC

MFEKKNLLSHVTKFKYSGIYMQSCGFLGLLNVEHSQTLPLMYFHATILGLASFHFGYGGYEKRYDWDFGELVEAFHSAIINFTGFCVFLMIVKQRENCFYLFHKILDGIYQYPWGPWKEETALRFKSEQTVKYLCLGYPAIFLNGRLVEYVRHFWTQNRQPYEPFFKGWVPYSEDTIYTLICVSVFHGAIFSCFFFTASCICTFFGVTCERFLTEVEVLKIVLRDPHYIVETQGVSFNEVIKCCIKHHNAINKLMRTVFEGMLIAFFINMTFLLAAIALLITVSLIHKNSQRKQTGDLCNTIYQMKWYDLKPQDRKQLVTIMIRTQEPMTLSGGGIKVFNLETYTE

>GbueOr106NI

LILEGNTLRAIDIVETIHFSSHLQSGLMFSLFLYYNSERFKKIDDQVLTGIYHYPWGQWDEEVEIRADSQKRIKRLTRQVVVVSLLGFWPMMFKNFILKPNEQYPLIIIAWIPFAFNTLPKYLVAYFYQLIGCLLCLMAWFSIITLLIENGEHFVSHTKILNSALKNMIKLGGKNELESSVKYCVTHHQTTIDSRVTSLEKIPLLLMLPVESVYPFIFCWYGQRIYDESEYTRDAIYDMEWYTLPPHQRKDFIIIMIRCQKPITISGGGLMKLSLAAFQEILTTSYSYLNMISAVN

>GbueOr107N

IIYMEFDNLQKVENTHWTFLYLTLSLLAHQTFSGTNDLILLDKEITSGLYSYSWGRSKEEINLMKKAEKKHTTFARFYFWIVHTAFVFQVIRSSFFNQKRQGWDLLYYSWQPYPIDNWPGYIAGNTYQIVTGLCCTLSITSFFAFISACSEHLIAQIKILKYVLTTYIVPNEDSDYGHLDALKECIIHQQTLYRICSVFQRKMSHSLLLVYINFGVILCTTVYIMIDSTTSLDIILSFIFVLLPEVLLPFSLSWYCQKIYDESEELKDVIYSLDWMSLKPKQRNDLICIMTRCQRPVILKGSGITQCSLKSFQEILTASYSYLNMLRAVRQ

>GbueOr108N

VGGKYTGNIKYIMKCRMLDKGTWLSRITSICIICVLTSGSLHYSSVVYSDYEKLDVLEIMENIHWGILFFTTAQMSQQMFKGSGTMIWLDKQISHGIFQYSPYRSACHTQILDRAQSKHTFLARIFEIMTFGGASLQFVRAAFFNPKRSGWDLFYRGWLPFEVNSPLTYIIGNCFQLLVAVFICLTIIGFVNSFMGVSEHLVAQFYILKDVLRNTKTLAKTPEDVPNIIRLCVLHQQTIYTFFEKFQSYLSYMLLWMFINFAFILCTIGFLLTDKKSTPEVMLSFIMILIPELIIPFTFCWYSQMIFDESSGIREAVNDMEWYTLPPQYRKSLVLILTRSQKPLALTGGRLKKYSLESYQEILTTAYNLLTMLRVVRN

>GbueOr109NF

QGWDLLYHSWQPFQVNSWLTYVIGNCYQILTGICCCLVVMSFFPYTTACSEHMIGQLKILKYVITTSQLLKNKERSNTILRECVKHHMCLYEAFNGMYKIMSYSLILVYINFGAILCTTVYVMTDPNTTLDVILSFTFILIPEILIPFLFSWYCQKIHDESDEIKDTIYSIDWMSLKPKQRKDLIFIMIRCQKPIIFKGWGIANLSLKSFQELLVASYSYVNMLRAINSNEESKNTL

>GbueOr110NIC

IYYNSQNLMLHEIVENLHYLVHVVSAIVMSFTMFYGSDKLIWLDEMVTSGIYIYPRGCTTGELQVRTEANRKITSASKALIAIPLSGILLMYIRRFFQKQYKYPLFNEDGWYPFELTWITYPIAFVYQFVWLIPATLTWISTLCIFIALSEHLLSHLKIVNYWFSNSYKGNNKNEEFADNIKNCIDHHSILTDEETPIIYKLVYFEAFLANICFPLTFCWYGQKIYDECRNTTDAVYDMNWYELPPRFRKNLLFIMQRGHNAWILKGGGILPFSMMTFKE

>GbueOr111NC

LRKHDEGGSYMTRMLVRFVGIWSETGSVFAVRIACMLSFSAYMSVATFSYLYPTDDGIITYGVTLSMIVYGFFSIIKAIWFYYNSKKIGWLIHEVERILSENKSHEEEFMKAANNSTYLTILLICMTCSVLGVETLHCLFVSSNDSRMMFNLYSFIDPTRYYFLAWAFQTYYWMCVPVFLVTFNLLLRFTLVSVANLSVLESHFRAAPRNLSRFVNLHTDIISFVKVMNEVLGPAILCELTMGSILTCVFAYMSVESMTLQEKCISYTMFCCELFIPWFICHCGSILEEKSKALTDAVYSSAWIELKPKQRKDIIILMITTNKPLQIKLSRTHIINYAYFSS

>GbueOr112NC

LRKFDEGGFNMTRMLVRFLGIWSETGSVLAVRIACMLSFSAYISVATFSLLNPSDDGIVTYGVNLIMIVYGFLTTIKTIWFFYSSKKIGWLIIELERITCENISLEESHEQEFIKAANISNYLTMSLISMMFVVFGVETLYCLFLSSNDSRMMFNLYSFIDPTRYYMLAWTFQYFYIIWPVPEFLITFHLLLRFTLLSVANLSVLESHLRSAPTNLSRSVNLHTDIISFAKVMNEVLGPAILCELTMGSILTCVLAFMCVE

>GbueOr113NIC

FLSYDEGELLNVTRWLVRFLGIWSETRSILAMRIACTLSLSAYALVGTLSFLYPTGDDIITYGITLTMLTFSFLTIIKAFWLFYNSKKIGWLLNELERITIKNINENKSLEQEFIKAANTPKVRTILLFGLMSVVFVIETLYCLFISSNDSRMMFNLYSFIDPTSITKVVNEVLGPAITSELTMGSILTCVCAYMCVGSRTLQEKGVSYTLLCCELLVPWFICHYGTILEEKSKALTDAVYSSGWIELKPKQRKDIIILMITTNKPLQIKLSRTHIINYAYFSS

>GbueOr114NI

LIRKHQNNLFSVTRTILRCLGIWSEEGSILVMRIVCILSLSAYMIVGTISFLHRTDDDIVTFGITLCMAVCGFLGIVKALWYFYNSKKIGWLIHEMERITIENTSKDKSHRQEFIKAVDIPKVNTFFLLFMVAIVLGIETLYCLFLSSNDSRMMFNLYSFIDPTRYYFLALAFQYFYWIWPVNILIITFNLLMCFIMVSVANLSVLECYLRSAPSNLSQIVNLHTDIISFVKVMNEVLKPAIICELTIGILTFCISAFMCVEVIASLCDKSVFPMLHLFVALLFLGLFAIMEVFWKKSLFKCPIHFSLCFSV

>GbueOr115NC

LIGNYDNNLFSVTRTILRYLGIWSEEGSILVMRIVCMLSLSAYMIVGTISFLLRTDDDLITYGITLCMVVCGFFGMVKALWCFYNSEKIGWLINEMERITIENISKDKPHRQEFIKAVNMTKVLTILLLSTMFTVLGLEALYCLFLSSNNSRIMFNLYSFIDPQRYYFLALSFQYFYWIWPLNMFIITFNLLISSTMVSVANLSVLECYLRSAPSNLSRTVNLHTDIIRF

>GbueOr116NF

SSNKGKILNVTRLLLSFVGIWSEGETAFLTRTFCMMSLSAYTLVGMITFCYHSNEDVVTYGVTLGMSVFGIQSIVKTMWCFYNSEKIGWLVIELERITIENLSKENTYRQEFTKAVNNPKILSVIVIAMMGSVYLMETLNCLFFTNNEHRLMFNLFSFIDPTRYHYVALLFQYFYLMWPTPIFVTTLNLLLSFTMLIVANLSVLETYFRSAPSNISRSIDLHNDIIRLVRVMNEVLRPVLISELASSSVITCTCAFMFVKSTTMEQKIISYSVMICVVVPPWFICLCGSLLEDKSAKLTEAVYSCRWTELKPKQRKEIIILMIATNRPLQIKLSRTHVVNFAYYTSFIQMSYSFFTLLLSMDSDGCDDQK

>GbueOr117NIC

LIGNNENNLFSVTKTILRYLGIWSEEGSILVMRIVCMLSLSAYMIVGTISFLHPTDDDIVTFGVTLCMVVVGFFGMVKALWYYFLALAFQYFYWIWPVNMFIITFILLISSTMVSVANLSVLECYLRSTPSNLSRTVNLHTDIIRFIKVINEVLKPAIICELAIGIFTYCISAFMCIESVTLEEKCISYSTFVCCLVVPWFTCHCGSILEEKSAKLTEAVYSSRWTELKPKQRKEIXFIQITYSFFTLLLSVKSN

>GbueOr118NF

LLKHDETKLLNVTRILVRFLGIWSEEGSVLAMRIACMLSLSACMIVGTISSLYPTDEGTTTYGMALAVGAVGYFGIIKTFWCYYNSKKIGWLIKQLERITSENISQEESHRKEFNKVVNMTKLLTVILLIIITVVYVMETLYCLFLSSNDSRMMFNLYSFIDPTRYYSLALAFQYVYWTWGIPNFLLSYNLLICFTMLSVANISVLEGHLRSSPLNLSRSVNLHTDIISFVKVMNKILRPVIICELTQGSILTCVSAYMCIKSITLQEKCISYGVLCGELLVPWFICHCGAILEDKSMALTDAVYSSGWTELKPKQRKKVIILMIATNKPLQIKISMTHIINYNYYSSFIQMSYSFLTLLLSFKSN

>GbueOr119N

LFRDDQSKLLNVTRILLRFVGIWAEEGSILGMRIVCTLSLSAYLVVGIISFLHRPDEEFITYGVTLSMSVFGLFSLIKTLWCFYSSEKIGWLINEIERITAENMTKEETHREEFRKATNTTKVLTISTLSMLFIQVVFANLQSLFLNNNDTRMLFNLYSFIDPTRYYFLALAFQYFYIIWPVTVFIIIFNMLICFTMLSVANISVLERYLRSAPSNLSRSVNLHADIIRFVKVMNEVLRPAVIFELTMGCILTCIAASMCVKSITLQQKCTCYPLFCVLLIVPWFICHCGSILEEKSMKLTDAVYSSRWTELKPKQRKEIIILMIATNRPLQIKLSSTHIINYDYYTSFIQMTYSFFTLLLSLNSN

>GbueOr120NIF

LLKQDESKLLKETRMLVRFLGIWSEEESTLVMRMTCTLSLSVCMIVGTISFLYPTDEDSINYGVALAISTLGFLGIIKNFWCYYNSKKIGWLIKQLERITSENISQEESHGKLFIKVVNIIKLLTVIMLVMISVEIVIESLYCLFISSNDSRMMFNLYSFIDPTRYHSLALAFQYFYWTWTVPNFIITYNLLICFAMLSVANLSVLEGHLRSAPSNLSRCVNLHTDIINVVKVMNEVLRPVIICELTLGSILTCVAAYMCVESKALTDAVYSSAWIELKPKQRKEIIILMIASNKPLQIKLSSTHIINYNYYTSFIQMTYSFFTLLLSLKSN

>GbueOr121NI

CFYNSEKIGWLVNELERITIENISKEKSHREEFTKAVNNPKILSVSLIIMIISFYSMDTLKRYFFSTEPQLMFNLYSIIDLSRHYFLALLFQYFYMMWPTAVYVLTLNLLMSSTMLAVANLSVLETYFRSAPSNLSRSIDLHNDIIRLVRVMNEVLRPLLIFELASSSILTCICAFMSVESTTVEQKCISYSAAICVAIAPWFICRSGSILEEKFIQMSYSFFTLLLSVNSK

>GbueOr122NF

LLRYDPRKFLSVTRILLRFLGIWSEEGSSLVMRIVCILSLSVYTIAGMISFLCRTDEEILTFGLTLSMSSFGFLSIIKAFWFYNSKKIGWLINELERITFENISKDKTHYIEFNNASNISKVITSLLVCMLFIVFVVETLYCLFLSSNDSRIMFNRYSFIDPTRYYFLALAFQYFYLIWSIPTFIITFNLIMCFSMLCVANLSVLECYLRSAPSNLSCTVGLHTSIIRIVKVMNEVLRPVIICDLAIASTIACACAYIGVKGNTMRDKSNFCAVLFSQLGISWFICQCGSNLEEKRKTLTEAVYSSRWLELNSKQRKEIIILMIATNRPLQIKLCSTHIINCSYYTSFIQITYSFFTLLLSKNSN

>GbueOr123NC

FFYNSKKIGWLINELERITTKNISEEKSHEEEIMRAANTTKLLTVILIFTICGIYVFETIHCVFLSSDDSRMLFNMHSFIDPTRYYLVALTVQYFNLIMPIPLFVITVNMIMCFTMLSVANLSVLESNLRSAPSNLSRSVKLHTDIISFVKVVNEVLRPAIICELTLGFILTCVCAYMCVESMTLQEKCTSYAILCCELCVPWFICHCGSILEEKSESLTDAVYSSGWTELMPKQRKDIIILMIATNRPLQIKLSCTHIVNYTYYTS

>GbueOr124NI

YYLALCFQHFYWTWTLPVFITTLDMLMSFTMITAANLSVLEKYFQSAPSNLSRSIDLHNDIIRSITTEEKFISYSAAICELVIPWFLCQCGSILEEKSATLTDAVYSSRWTELKPKQRKEIIILMIATNRPLQIKLSTTHIVNYTYYTSFIQLSYSFFTLLLSINSN

>GbueOr125N

LERSDEEKYLKMSKIILNFTGIWSKRGSTTTMRITSVTSLSVCLCVGVLSLLWPADQDVTTFGVSIMIVAAGFFNGVKVLWYSYNSTKMGWLVNELERITEVNVATEKRHEVELTKASKIPDLLTALLTCMIMMIFVLETIYSVFITRQEHRVMFNLYTFIDPKRYYFVAICVQYFYIIWAAAGVIINFNLLLSFTMLIVANLSTLEKYLRSAPHKINRSVNLHTDVIRFVRVMNEVLQPAIISELALSCVISCMCAYMCVKSTNFQHKMLAYVMMCTESTIPWFICYCGSILEEKSGKLTDAVYNSGWTSLRPKQRKETVILMIATNRPLHIRITNTHIINFTYYTGFIQVTYSFFTLLISLKESN

>GbueOr126P

SKIIHVPYILIFFAGITTRHYFFNXRFFCIMSLSAYLLVGMISFWYHTNEDIITFGVTLGMTIAGFHNLVKTLWXNNEHRLMFNLYSFIDPTRXYYQALFFQFFYWIWPIPIFITTLNLLMYYEILRFVRVLNEVLRPILISELTSGSMLTCICTYSCVKSTTIQQKCITYSLLLCQFVIPWFICRCGSILEEKSTKLTAAVYSSRWIELKPKQRKEIIILMIATNRPLQIKLSRTHIVNFAYYTSYIQLSYSFLTLALSLDS

>GbueOr127

MKSHKIPLLAFKSHLRVIGINVNSENASKFGKFADNFKVCYCLLCYCYLIVGFTHTVLKRDEDFYNCLMYLIPTVIFLIKYLQMINSKKSIKEFIDEIDNTYRNEEEENKKYIDEADKKIKMGIHFLIVGYFISIGAILVPSYIRRLLGRNLSKAPLTMFTPFDPATEYLNVVVFQFCVLLGIAHVALMTYVTIFTFTLQMRAQVQVIGSVFRSVQYDEEESITRSGYEEQNVNNIVKIKKCILYHARVLRLHSISNEMLSSVFSLEILVSSIQSCLTLYQATRSASYINEFYIHSLVMYLWSYIICWCGSQIESLTSELLNFCYGTVWYRLKPKDRKMILTFMTAANVPLHFNYRGIFNINLEQYITILKGSYSYYTVINGLEGV

>GbueOr128

MMEYKDPLYYPKLLMIVTGFETNRSIKARIRSAVLTCFYIIGLIVGMFCHSYISFDQLRDHNNSEPAVYACVVFHIAVKYLIWHLRHGKIRALMDKIDQNYRNIIKDTDNNEIMDKAVAKCYKFSKVFVTCIIIVYLNHINVLVNHMLDPENEFEMPYLSYVPFDTHKHYYWAVTFQYIICGSPFPLACMQYISLLSFTILISAEMDVLVEYFQSLKTSFSNNKKDAREKLIEGIERYRDIISAFFSFFFSFIKIVTLKIILFVSLTFMLYKVIPKTSTFIPINDPHFLIHMSTFLVTVALPFFHCWCGQKIVNSSSRILEGVYGCGWEQAPISDRKLVLVCMTMAGKDLNLNWKLFIYMDMERYSMLIQGAYSFFNLIRNVNS

>GbueOr129FN

LFRTIIIFSFMQKVNINIWIFSDFISRCKSDNSFFFLKVIYNNILLPNYFFYFRYNTFCSMIIYARAQADILKTSLNMLQGFKSSALNRADSDSSQNPIEDITLDSLSSMTEIREHSLKECASLHAYIIEMVSDINKVFGDALILEIIISSLQSCMVLFQCSKTSFTDAKVITLISLLLCTTSMPLSICWCGQLVRSIGEEIRESIYDVRWYEANPKARKGIITLQIQANKLMSLNARGMFHIDISTFMMTMQTAYSYFTLLRTMDN

>GbueOr130I

MAKLDEFIRVRVFFAFSGIYLYPNPPRWMAPVCLAIICIVVLLSLINTICTVMAAITFDSDFSDRLIGLESVFMSSLGVVKVLIFYRHRDQVASLLNNVGYFISKTFTSKIVRVTMISFGLSMFGVPGFWTINALAFGHFKTPMPYWFPFEINTIERFVPAFILGVVSLVSTVYTNTLIDNSVLLIVSQLCARVLVLRKSLQKLGNRHENIGKKLKVPKEVVEGDMTASFAESDDDLDLIDGLSVITRDSDELLIKDCIKEHIEIIRFEKLNFACYANSWYLMTPKLRKSQAIMQEYTKQAFKFRGLYMFNMSHQIFLRVIQDSVSYFMFLRTFTM

>GbueOr131NI

MINGFCTIAGMVNLTSNFADRLIALESAFVSGLGATKIVMFYASTQSVSRLIHELGGFKADTDIDKRVVRVVKAYTASMTGVPIFWMVSALLSGEMRTPMPYWFPFEVDNVSKFVPVFTLGSVALFSLCYTNTIVDTAILLMISQLCTKLLVLRLSLENLGKKRHLTQNSDNINRLNHPNLLPMRKSKYQQLLINKTLHKHEKRTSNKINNLTIEEKQNKTEDGIRSLEHITTDKFHEKEIPRFTGIQLILSDSPMELLKQCIKYHNETMEIEDASKDGMKFIPIFCAQYIQYFIYCWIGEQLTVHCEKVRESAYCSDWYECSPSERKSLFLLLEYTKKSYVFKGCYVFSMSHSTFLKIIQDSLSYFMFLRTFVSKS

>GbueOr132I

MKSNHVDLKYFKMLGFWDLVMTGSRKSLISFSIILGLFSLHVTVQFFDSLIGKYDFSLFTEKFAVNLTMFESSVKMAYYIYQRETLKHLSNIFDEKLLKVSKIRKEKADEILGHAGAKINSAIRAFVIMIFSTVSVWNMLPLIECIRNNIFGKLQITPSWYPFDATVPPINCFIYLYEFGIMVYCATLLYLVNCLFYSYALYVTAQIQVLNDCLSNIEVMARETLDIKKPISLDPVERSQKQLVMHKLLHECISDHDAILKVQMSGNASFVASLKFVMYLIFGLMELLVYSWGGQIIYDQSGEVYRSLYDSGWEDCSTAYRKDLLFAMTRAKKQECLTAGKFYNVNLASFTQIVKASYSFYSFLHGSEKETG

>GbueOr133F

MAMMCNKYVEHEFLLLNNVFYEMFGISGFDLFMSGCPRRSYRLLRVAFFTYFLFINMPIYFISQIYHIFFTMPDLTMYILNLGIFLNAVCGFVKGVIFFVKISKMERLCLKFDCFIVNRVNSELALKLLKEKAKFLRRLSKIFLILPYSVFIVWCIWPVIESEAVLTFFGITLEKTRLNKILPSVYPVDMTVEPNYTLIYIFEVLSGVSGLTYLMPVDMFFISVCEMYITQVEILKKTIAEKINLKSWKEGCKDFDITCVVKEHIILLKHLKDIGTRLSFILGIQWLTSITSICSIILSFSSKSGQEDFSYFEFMSARLKLCMYFGVIMAELLLYCIIISRIKQANSSIGDAIYCQDWPNIPDNSFRLHTHMIITRSQHPVDISALGLVKIDLDSIIDVIRISYSYYTFLRELN

>GbueOr134

MKEKDHHFNFESVNNVFYKVFGISGLELFLKNGPEIKYRRVKTIFLGFFLFTVFPFYMLMHVVSFFSDGINLQIVAINVTIFLNASGGFVKCIYFIIIREKMESLCVKFEEFKSAKTDYKLAYDTMSSMSLWISKIIKITLYLNFIVFLCWASFPLPWSKTVKSWLKIESTSVDYNITRRLGLPNKLIPCYYPFDTDPSPVFEIVYFLELITAVSGLTYMMPFDFFFITVCQMFCSHIKVFNNAIINNINDTNFTINDVIDEHVTILKKMKLINEILSPILGMLWINSVISLCGLILSFSAGLREELGYFEEFIFKMRLYTFFLLLLGELLFYCILCSNIKHANLSIGATVWHSKWYNKDKDYVLACRMIILRSHKEVNIKAMGILKIDLSTFIDVLRLSYTYFTFLREIYK

>GbueOr135NIF

MGFRHEDFLSSRYRQEYAAKILTRKYKLLKKMQTFFYSGCFLNVAFWCIDPLLRASFHVLPPNYLMVTITNPYIQVAVAIFEAYHLLFLITHFTVVILYFLFAINMYVAQMKILNECLIDRTKETTFKLISEDSEEITGSIKNPKPISTKDYISDHQKLIKRESTHFALYQSNWYENDPKYRKIILLSMSTLWPTNPITAMGFADIELETFIAVVRMSYTFYTFLRETVGK

>GbueOr136

MNLRDKVLTILERVSKDKVKRGYTFHKGVLLLLGYDWWSSSYREMPVWYVPAHLIYFAYQIFNQMGLFIFGMYFVYKMEVKSDEDLTDLVKNVILCMALLASSAKAVFIFYKRHTIKQLIDYMEGLGELPSTTPYLERALTASVGYLGVVYLNALTWSIYSGIFKGGELPILAIYPWDTDNSFSGYIAGYGVQVMASVFCALSHGSLDTLMPVSMGVVCQHVKALRLLISRLGTESHHIDTKIISSFVELHNKVLRGVELMNETAGFLFMTQVFYIITHACIIIFLVVRVNDFASIVVNVIPMLTAAYTQLLLYCYFGQMLTSQFDDLRDDVRNNEWYSSSEKTKRSLIIIGEVCKRSIGLEGGGGVFAASHHTYLQSLQESVSYFLFLKTMTGQDE

>GbueOr137IC

MKMDLTGRLLGFLERVSADSVGRGFWFHKAVFRLLVFHLWDRQVSFWRRVLKLLYFSCQILSQVAMFAFGMHFVFSMQVQSDGDLAKLVKNVTLCLGLFASSYKTMFMFYKRHSVERLITYLEELGEFPTTKPHLRQAAVSSVLYMGLALINGIASSIYTPLIKGGEFAVRAIYPWDTEHSFVGKAAGWLVQWSSSLFCAAYHGCVDSLVPVCIGVLCQHVQVLKYSITNLGTGHRSHDDKIISDFVDLHNKVLRVNDFGSTVANVVPMLIASYSQLLLYSFFGQKLTSQMEELREAVTGGKWYSCSKEVKSSLSIIGEMCKRPVGLQGSVFSANYPTYIQ

>GbueOr138

MGWFRKLIDGVRDDEPVLTFQVMGMKTNGTWPLESNTHAAVKFLYRLYFNFTFTCLVLTSVLSLSAVMAKMSLGSLASTVDILTMFATAAFKMIIMKNNEENMLNILKEIQSFSRNSVYIVTGWSLLVAAHVYLSQDSYLFSLLHICCFHINQIGHGLQAVDKLPSNKVAKNNNKLEGLKKLAAFHAKILNLLKTVEYNWRSMLVADLFHCIMSLSFGLFNTSVTSSLPARIKTYFFITYSFFHEFLNSYFGEMLENMVSEWLYEMDWLSMSIDERKHVLFMMTRAQRPVKINAWQVYDFSYPMFMELTKALLSYYMVLKTLLDKSTI

>GbueOr139I

MMSCFRTRIIDGVREDEPALTFQLIGMKIAGIWPLDDDTHTIFKYLYRLYSTFTFICLSLSTVLSFKAVTNKMSLGLLANTVDILTAFVTAAFKMIIMERNKENMLNILKEAQSFPRNSIVFLVTGWSLLFAAHIYLSQDCYFYLLIHICCFHINQIGHGLQAVDKLPSSKVAKTNNKLEGLKKLAAFHAKILNVTTSLTGQIKTYCFITYGFFHEFLNSFFGEMLENMVSEWLYEMDWLSMSIEERRHVLYMMTRAQRPVKINAWQVYDFSYPMFMELTKALVSYYMVLETLLENSKK

>GbueOr140

MLIEEEINAMISEMKEGGLWMKPRSRSIINSFRLGLNIMFWLVNIFSMYLSENASMLAMCMIYLSATTYVALQSLIVVMKHDTLEQILMILATVASRKTPWMRNIIIKDSNFSKRIISSVNFVVYLLFYLYFYLPLIIDTVLMFLGRQEPFKIPLPYSALLGSAPQRSLYYYLITISSIAYLQMMIRYFITILSLFCHLIVYAVAEIKIFKRLILTLGLDDKENIKVPFDLEAALREVVFFHSRIIKFIRLLKEYYGLVFAVQTIISSISICLCFYGFTVFWGKRYTMMAIFNFSSGVTHLYLMFIICFLGQYLETNSEELYGALYETDWYKASIPVRRNLIMMMRQAEKPLNINYRGTSTLNAILFMDIVNTSYSYFMLLRSID

>GbueOr141N

SWITDNPVSSQILVNQALCLAIDLWFVVRWIAIKFHSREFSKIINDIEKMTATSREHVPEATDKEMTERWKRCKIAFFLFTVQPFAFCLHWLFWPLINRLIFGELEFIIDNTSFVDKNTFYFDLYYLGEVCDFLPALYCVTVYESFFITSIEMLKGHYRILSKELQAMDLQARATDLKNKVTMSYCVKHHQEIIRISLKLKNLTSTILICHSFATVVIICVSVFALTQLVSELSHSKFMNFVELSGGAVFHFFIISQICEQLTAESLNTPLEVYKTRWYEPHFKMSDRKSMLLMMTMTNMPIVMGGFVPIGLSSFLTMMKTAFSYYNFLTAVQ

>GbueOr142N

LTKYARENVKEIVDEEIVYRTKRCWLAFYLFTFQPFLFVLHFIIRPFIDATFYNRPEARFFECYSFIKRETWMQFFMLWSFEGIHFLGSVTIIIVYEGLFFTMLEWILGHIEILKKELPLIEFTNLNEKLKSNGQDSVKQYTISIDQAIKHHQSILKVSRDLKSASAFAIQGTCVGSGIMTMLTIAALTVSFTEFGKGKLINMVEIAFAHVFHLFLLSFMCEELTYQCGELYKHIYNTKWYNGTKSQKMKLRIMMTMTNHPVTLGGFIIVGLANFIAIMKTGFSYYNYLIAMD

>GbueOr143NI

MIDNRFFLELTENARENVKEIVEKEIVYRTKRCWLAFYLFTFQPFLFLIHFSIRPFIDATFHNRPEARIFECYSFIKRETWMQFFMLWSFEAIHFFGSVSIIIIYEGLFFTMLEWILGHIEILKKELRLIDFTDLNNKSKNNGKIPEEQYTLKIDQAIKHHQCILKASRDLKSASAFAIQGTCVGSGIMTMLTIAALTVSIMKTGFSYYNYLVSME

>GbueOr144

MEEFLERQGRNRLIALASGFMGPDRTRFKDKWYRKIYMFYKYFTDVSVWVNFLTVLSHFLRPEIPLIERCLGGFPLCTLSLMVIRITYLHFSRPKLVDLIQGYMNSFKNDPDPMHLEMEMARGRKFMRPFPIILFMCNVASMAPWCGAPFLLQYLTGERKMSVPADFPVEFEKSPNFEIITFIQILGATLTPMKVIASDDLFYALAFFQLAQFRHLKHNFGIMFKDINISKDGQASFQGQSKDRLDERMEARLRQWIIHHQETSALFAKLQGIFSFVFLITFGLQTLVLCLTAFIVAVAPLDRVTIVFCAFYVLGNALELLFLCRVGDFIIIESGELTEGLAGKNWFVGSKKVMTLLKICLTRCYRPHKMSAFGVFPLDTDTFKSIIISSYSYFTMLLKIKK

>GbueOr145C

MNERRKLKIDDWLEKGIMFLKVSCVYIDKTEWWGEPLQYVSIVFNLSSVICLTYSTYVYMNEIDKLATAAHHWVIMATVVMLARHYRKNRDVIDKFVMEMRTTFMYGDWFPEEEMDKIQMRRSDWNRIAVRSLWGACIYTYINVAALGMLEKYIGDSEFFVFFPCWVPLNLDNLASYIIFVIWQSVCIQTAIFVIFGLLASQFYVYSFSLSELMVLLAALKRLKELDNEFETKMAIKQIVIHHQTFLKSFKELHTFLGLLNLVIITCGVAVMTITGFALISDNTSVKMKFLGIMVVQLVFTYTSCRLSQDITDAGFEVRQACYRTEWMSMDRSSVRNLQLMAERAIRPLSFIALTGHELSLEMYMS

>GbueOr146P

MNEKRKLKIDDWLDKGVMFLKISCFYLDKTEWZGEPLQYVSIAFNSSSFIFLTYSLYVYMNEIDKLANAAHHWLLMAVVVLTVRHYRKNQDSILKFVLEMRTTFMYGDWFPEEEVNKIQMRRSDWNRTAVRSLWAVSIYIYINVAALGMLEKYIGDSEFFAFFPCWVPLNLDNPVSHIIFVIWESVCVQTAIFVLIGLLASQFFVYSFILSELMVLLAALKRSKELDNKFETKMAIKEIVIHHQTILKSFQEIHTFLCLTNIVIITCGVAVMTITGFALISDNTSVKMKFLGIMVIQLVFTYTSCRLSQDITAASFEVRHACYCTDWMSLDRSSVRSLQLMAERAIRPLSFIALTGHELSLEMYMSMIKAAYSYFNMMLQQK

>GbueOr147NC

YMNEIDKLANAAHHWLLMAVVVLIVLHYRKNQDSIHKFVLDMRTTFMYGDWFPEEEVNKIQMRRSDWNRIAVRSLWAVSIYTYINVAALGMLEKYIGDSEFYAFFPCWVPMNLDNPVSHIIFVIWESVCVQTTIFVIFGLVGSQFYVYSHIISELMVLLAALKRLKELDNEFETKMAIKQIVIHHQTILKSFQEIHTFLCLMNFVIITCGVAVMTITGFALISDNTSVKMKFLGIMIIQLIFTYTSCRLSQDITDAGFEVRQACYSTEWMSMDRSSVRNLQIMAERAIRPLSFIALTGHELSLEMYMG

>GbueOr148FIC

MIERRKLKIDDWLEKGVMFLKISCVYIDKTEWWGEPLQYVSIAFNSSSFIFLTYSLYVYMNEIDKLANAAHHWLLMAVVVMLVRHYRKNQDGIDKFVLEMRTTFRYGDWFPEEEVDKIQMRRSDWNRIAVRSLWAVCISTYINVATLGMLEKHMGDSEFFSFFPCWVPLNLDNLVSYITFVIWESVCLQTTIIVVFGVLGSQFYAYSFILSELMVLLAALQRLKKLDKEFKTKMAIKQIVTHHQTILKDNSSVKIKFLGIMILQLIFTYTSCRLSQDITDASFEVRQACYCAEWMSLDRSSVRNLQLMAERAFRPLTFIALTGHELSLEMYMS

>GbueOr149P

MNERRKLKIDDWLEKGIIFLKISCIYLDKTDWWVEPLQYVTIVFNSSSFICLTYSLYLYMNEIDKLANAAHHWLLMAVDVLIVLHYRKNRDVIHKLVLEMRTTFIYGDWFPEEEMDKIQMRRSDWNRIAVRSLWGACIYTYINVAAFGMLENYIGDSEFFAVFPCWVPINLDNFISHTIFVIWESVCMQTTIFVIFXSTQFYIYSYILSELMVLLAALQRLKELDNEFETKIAIKEIVVHHQTILQSFQELQSFLGLLNLVIITGGVAVMTISGFALISDNTSVKMKFLGIMVVQLVFTYISCLLSQDITDARHACYSTNWMSMDRSSVRNLQLMAERAIRPLSFIALTGHELSLEMYMSMIKAAYSYFNMMLRQK

>GbueOr150NC

MRTTFIYGDWFPEEEMNKIQMRRSDWNRLAVRSLWGACIYTYINVAAFGMLEKYIGDSEFFAVFPCWVPLNLDNFISHTIFVIWESVCMQTTIFVIFGLVGSQFYVYSYILSELMVLLAALQRLKELDNEFETKIAIKEIVVHHQTILQSFQELQSFLGLLNLVIITGGVAVMTISGFALISDNTSVKMKFLGIMVVQLVFTYISCLLSQDITDASLEVRHACYSTNWMSMDRSSVRNLQLMAERAIRPL

>GbueOr151NI

MRTTFMYGDWFPEEEVNKIQIRRSDWNRIAVRSLWATCICTQINIAALGILEKYMGDSKFFGFFPCWVPLNLDNIVSYITFVIWQSVCVQTTIFVILGALCSQLYVYSFTLSELMVLFAAIQHLKELDNKFETKMAIKEIVVHHQTILDNTSVKIKFLGIMVVQLVFAYTSCRLSQDITDASLEVRHASYCTDWMSMDRSSVQNLQLMAERAIRPLSFVALTGHELSLEMYMSMIKAAYSYFNMMLTQK

>GbueOr152I

MEGRRKLKTDDWLEQGIIYLKVACVYTDTSVRWVNPLHFIATIFNISSSILLIYSMYIYMYEIDKLANAAHHWILMFDVVILVYNYRKNRDIIENLIQEMRTTFPYGEWFPEEEVEKIQTIRTGWNRTAVRSLWGITVYTNINLAVLGMVEKYMGDNQYFILFPCWDNTSVKIKFLGIMVTELVYIYVSCRLSQDISDASSDVRETCYDTDWTSMTMDRSSIRSLQLMIERAFRPLSYSSLTGHELNLSMYMNMVKAAYSYFNMMLTQK

**135 GbueGr proteins**

>GbueGr1F

MGVKDLLLQIGKAVLPTPLKLVVSVISFSLPGSYYKDVKPILYTLRILGRLPVYITDNGLEKCSFLSWPWIYSIICLAVQIITTAMGMNYIVTSMNQSNDYDEYLFWLMMSLILSLNIVVPFTYWTDASSLTEYLKQWTRFQNEFFTVSKGPLGKIEFLEGDIQAGSKNYIFWMSVINIPMVVTITCVEVIFFVELPYIVIFPYVVIFGAIFSSSLYWCSACRQVSRISEILLDKTVQAIKNDKTEELVWCRTCWIRVSGLTSGLGSSMKNSALVNVIVMSVSFVICMYACLSVMSRQITSQYLVGILTEIPITITVLPITITVLILIFNHSNTATVGTQFGSTLLSMDLSHLSTASSAEVNFFLHSLTLSPPIVKLADYVDVNRRLITSFASNVITYLIVLLQFRDTGSKVINSYTHSLNTTNSPLSP

>GbueGr2JF

MKMKDTPQNAQERNITLLKFTQGEKKVGWVTNESYPISTYYEEMKPMYLTLFLLGRLPVKFTKHGMEKCDLLSVPVFFTVISTLFQFLLSTMTINYIVEVSQRGSTQSDVKPYFFALIIILSMNFSIPFSSWPDTKTNVKFAKDMASFQEEFMWSVSRLNSASPSAWIENFISATRGSRPSFIRIMIFIYFPIFSLIISIAALFLIEISLYLCVPFILIFMSIYATVLLWCMNCYCLIRFGDILSQQTIKAVKTGTIEELKWCRLTWCQLSHLVRSMGFSNQMTNILGNIIMGVSFVITCYAALHSYYSSRFSKHMMGILVGLPINSYIIYITFKSAHDVTKRIGPMFATGLLSQDLSHLSTRQCKEVEYLLQCLQLTPPIIKVADFITLDKSLITSFASSAITYLIVLIQFSGPGDKRFSPPNSNMTSSL

>GbueGr3N

GLEKSEILSLTVVYSVICTIFQILLSVLAIGHIIEIAMAEATNFDHKPFLFAMIVILTMNFAIPISHWLDTNSNAVFNNKMASFQEEFMWSVSILNSSSPSAWIEHFVAETRSSRRSFIRFMSYVNFPIVAIAIAIMTLFLVEMPMYVCAPFLFIFMSIFTATLTWCMNCYHLKKFAEILAEETVKAIRRGSLADLRWCRLSWCKLSYLTREIGFSCQFSAILGNVIMGVSFVITCYAGLAAYYTSRFSKHTMGILMGLPFNTYIIYITFKSAHDVTKKIGLTFAHRLLSEDLSHLNARQSKEIEYFLQCLQLVPPVIKVGDFVTLDKSLLTNFVSNVITYLIVLIQFSGYGKMKQTHDNYNGTALSS

>GbueGr4

MGILTHKETLVKHYTTDGFAEVPIKCCYYEVMKPVFWGLRLLFKFPYHISPQGEFRVHYQSFWMLYSIAMYIGLFYPAVIVIMEVYDQYKSSCTKDTLLMALSVSSFMAFHVQSPGGFLIESKKVACYINSWKEFEEEYWRMTGEELQPTLGGWVRTFVILLLPSIIIFFSVEQTVFSDLMDIKLIVLVATYLSAALYLYFFVFTCNYACYVAKSLKKHLKKTLITGISSVRSEALVNLRVLWVKLSDLTEEIGGSIGRTLMLKIGAYILSTIITSYNILENIVGWRDLWSPTPFYILGFLYFHVVAILNLYLLTASAHEAADEVGLCFVIDLFTMDTSTMDFETKFNIIEFINTVYLNKPRITIGGYFTINRQLFMSCLGNTVTYLIALSQFKTTEKKL

>GbueGr5

MVLRSTDGFGASKKCCYYEDLKPIFWGLRLLFKFPYHISPQGEFRVHYASYWMIYSIALYAVLSYPYTLILIEVYENYNTTCTLDSLLIVLIVYGFTSFHTISINACLFESKALANYLNSWKEFQEEYWRITGEELKPVLGGWVKTFTILIIPSTFIFVSIEESMIEDLMLFKLIVLITIYLGVFFYLYFFVFTCYYAIEVARSLKKQLIKTLLSTTSDTRAESLANQRVLWVKLSDLTQEIGGAISRTLMTKIIVFVLMIIISSYNTLMTVFSWDSDGNPTPFIASAFVYHFLIATINLYFISASAHAATDHVGISFALDLFKVDMSSMDLETKFNILEFIHTVYLNTPIIDVGGFFIINRQLFLSYFGSTITYLIALSQFKQAERRR

>GbueGr6JFC

MVLRSTDGFEPSKKCCYYEDMKPIFWGLRLLFKFPYHISPQGEFRVHYTSYWMIYSISLYAALSYAATLILIEVYENYNTTCTLDNLLMVLAVYGFTSFHVISLNACLFESKALANYLNSWKEFQEEYWRITGEELKPVLGGWVKTFTILIIPSTFIYVSIEGSMFDDLMLVKLTVLLTIYLGAFFYLYFFVFTCYYAIEVSRSLKKQLIKTLLSTDTRAESLANQRVLWVKLSDLTQEIGGAISRTLMTKIIVYVLIIVITSYNTLMNIFSWGSDGNPTPFTASAFVYYFLIATINLYFISASAHEATDHVGISCAIDLFKVDMSSMDLETKFNILEFIHAVYLNTPIIDVGGFFIINRQLFLS

>GbueGr7N

FKPFGWKAIYSYTVILLSAFMEVLSMIRITKRGIRYDTAGDIMFFGSNTIVMILFVKLSKDWREITECWLRVQKSTLPTKRRTKLKIQTVTCIITFLALFEHLSSDAYNFTQVQNKSKTLSGIFKLYFLKAFPLGFDTLPYSVGLGVLFSLVNLVDAIAWNYMDLIIIIIALALADSFHDVNALFYKIETKHVEKGDWKKLRETYNKLYVLTKLLDEKLSPLILMSFASNLYYICYQLLNSLKPIKSVSQGVYFFGSFLYLLGRTMSVCMSLASVPVVSKDILTSLFSTSSYSYNIEVQRLITQVSNEELSLTGCQFFAVNRNLIITIIGTIITYEIVLVQFNSLTTENSLSSTSSTNNTCICSS

>GbueGr8

MNSIGDKAADTYLGRTSIINNQNFIASRINLKPSTIGSTASKSEKLEDDSYSLQTSIAPALVIAAFCGFFPVKGITSKNTSDLRFSYWDWRSLYSYLIIFGSTLMGFMSIVRFLRSGIQLDTAGDIMFFMGNSIAMLLFVNLAANWKELMVAWHEVELILPPSNRRTNLKITFMIVVITFLSLFEHMSADANTLTTILKAESISWKVVGHYFLRSYKMMFDVLPFSIYLGVIFGIINLIDVITWNYMDLIIIILSIALTDLYRSANIMFDRTQDRVYDKDDWRQLRETYNKLHTLTSQLDDKLSLLVLLSIGSNLYFICLQLLNSLRPMKTLPQRVYFYSSFGYLLARTLLVCLSAASIPIEASYPLKILYATPSDVYNTEIERLITQITNEEVALSGCKFFSLNRNLVISILGTIITYEIVLVQFNNLNEEVGPPSISMNLTNNSVENLLDFK

>GbueGr9NIJ

GSNNLKNGKLKNENYSLQTVIAPALVIAAVCGFFPVKGITSKNTSDLRIDVITWNYMDLIIIILSIAMTDLYRSANIMFDKTQERVLDREGWRKLRETYSKLQSLTTKVDDKLSLLVLLSIGSNLYRPMKSLSQRVYFFSSFGYLLARTLIVCLSAASIPIEASYPLKILYATPSHSYNTEIKRLITQITREEVALSGCKFFTLNRNLIITIIGTIITYEIVLVQINNLNEDAVPASILMNSTNKTLE

>GbueGr10NI

VEEVNSIFGVGVLAAMFSVFIHVVITPYFFFLSFMYPTSYGIDKNNFLLVQVLLTAMYAAKLVLLVWPCSSAVQQITGAVTTYLVILIQFQNASS

>GbueGr11F

MKISVIESSRMEQDSYAEWSKCYKPLTTISRFMGLSSFEFGPNGVVPLKKSHLSYNIFLIIILTLDFFYQIVWLTLINTPISSITDFSQAFSLTSQSIIAISSTVTIATCAKHGLIKFKSMIQVDAELISIGIKPPYNFLNKCLKGIMGFIGVMYTILLSSDYIFRFVLETKPKVITFSTWITMNLYVVYSSMVLFQFIYIIIEIGLRFHLIEQEIKNMAQQSDNRTGLTSHQAVKKKTAHHQQKFTVKHKIEIINRLYNDLVEACLELSMFMSATNLLNIVTNLVAITAHIYTIVYLIMESKQNNKDYDSYDMIYNAYWTLFRTTALAIICTICSFTSSKANNLGVVLHKLIAAKVAVDAHEEIQNFSFQLMHRKLYFSVCGFFNIDLALFHGIIRTVATYLIILVQFAIAGFKESSKVGC

>GbueGr12

MGFKSELHSLCWFAKLSGMFHLNKNFKISIFWTLYCILIQLFSNALFLYSEIRRQMMKAEDINSVVFLFSAGAEAGLSLLNIACCISCYFNNKNWLEVIKGFEYIDWRLGSVKVTRWNRVTNGNIISTILLFLCAISATLSGRSIDIWIILFLWNTVTLHLTKMNTVLYLAELRLVKITQVVLHDPSKAVLCLDVYRQIDESISVLSSIFSYQIIIFFGITFATFTDTAFSLVMDVLNHTLQIDTIWGLIKIAADLLQLFYTMIFIPYQSTCTTKKAAKFNEAIYHLSVQDQLTGLFEKKKSRLHLLFQEDITMTAYGYFYMDYPLIQSMIAGAGTLIVILYQFNSSSGGKPPC

>GbueGr13

MNFLLKKLEGKNTYNGVYTDFSNLMFAAKLVGAYPYDVNINYSKSLIIYMSLSLLVSCSAGIRLCTLSSNEEQALLDGGNQLTEFTDKFFTFSCIFGEITNFILILLSKSEIRSCLYILNNFDSSILKYNLVPKNDKRFYLILFFSSILMLISITVDLEFYKNELEISILNAIFYYLCFYKNLVSRNIGNGILRLIRCRMALTIRCLTRITNNVNYPEICNICVLQEDLIQACIILNSSCLSTQYFFMTATSYVQALIYSFLLIVTIKTPLQRSVIPWKEVKTLILYSVSSFAWIFLLILDIWDLVSNFSQTVHMAEKFKEKLYSLVLRDNLSKLSLCDSSDYKLIHIHASSLPKLNFTAWGFYNFDFQLIHSMIATAATHLLVLLGFNPGTRTS

>GbueGr14C

MSNRIVFFALKGLGVFPYTLKHNKSQCSLELSYVGLAVTFISIITIIPKLLLARQAFTIFEGDLYILMAYNVINMLYCLIPLWKTIFNINYNKQFFETFFSLTGESAFKRFRRIDALLLFNTFCISVQAFLYVNTDWLISLNGITEMFFFNITFADTLVVIFILSVIEDCFQKLGEEVITRFNEGTITALEVSKFRNRLTSLFDFLMWVDYMFGLRNASSIFMTVLALMLSTYSILLIIVTTPFVLDYELIISLIFSFESIATILVLSYQCTQISEKNAKIADKIINFDLNLLHYNSLQEVYLFLEDLKDQKIIITACGFFEIGMPLVQS

>GbueGr15a

MQKVISIRQYKTSNARNNLILFKMSSSAMYNPYRDIERVYNFFRILGVIPLNSKHELTFNSFLYSTSIHFITLSTFYLTVYNKISWDKRGLFDTVWAKVFIILTSLAVVTYFVVMTKSSKYLIDLSQKMKDIDSKIGIPKKNVNNLYYIVIVGQSFYLILVVIIDFNLLPNPGRRSLAVFLRLYLFYHVISKLLALQPLIWRMDKLINLTKKNPITITKLLTLSDCYITIVDCAKAIDQYQNFPILVFMATFFFQMVATWYNTYTNVYSCLYGSCHPVTPILCLHCIMYFLNLLFIVNETSTLSIKAQEFNNKIYKRIINDKTNTLCQNKKLKLHITMKKDIVFTAWGFFNLDYSLIHSMFAAGFTFIVILIQLVPSELEKSMSMSMSNSTTEN

>GbueGr15bN

VISKLLALQPLIWRMDKLINLTKKNPITITKLLTLSDCYITIVDCAKAIDQYQNFPILVFMATFFFQMVATWHNTYTNVYSCLYGSCHPVTSILCLHCIMHFLNLLFILNETSTLSIKAQEFNNKIYKRIINDKTNTLCQNKKLKLHITMKKDIVFTAWGFFNLDYSLIHSMFAAGFTFIVILIQLVPSELEKSMSMSMSNSTTEN

>GbueGr15c

MCLSTEGNPYGDIRIIYLILRIIGVIPLNKNMTLSLPNLLYSVLVHLVVLLVTYWGFIDEIHDVINLTSFEHNWVVLFFVMAAISTLTYFIAMTKSSKLLIKLYKKIRIIDSIIGLPERNVSNKYYFVIIANICYLITVIVIELILFSKPEQSSIGFFLWTYLYYHVAAKMLALQPLIRRLDKLINLTKRQPITETRLSILSDCYIIIVDCSKTINKYQGFPILIFMMTFYLHMVATWYSFYKLAYTCVMAKICHPITLLIYLHCIMFVFCLWFIVDQTSALTDKAQEFNNKIYKRIINDKTNTLCQNKKLKLHITMKKDIVFTAWGFFNLDYSLIHSMFAAGFTFIVILIQLVPSELEKSMSMSMSNSTTEN

>GbueGr16a

MNLLNQQNFKAVKYQYTFFNLIGIFLMNSNFDLNFKHLIYCTFINAFVLIMYTSVVIYYYSRKIKTNDSSIVISYVFLSILIILSYVAYYLSSLNYRDKLKGIYLNFYYNFDRLINVSIKYSYDVKFILAIFFELLFLIWVCCNEQYPGNSEDKFGYMIFYHFTWVYLNLHVRCSFLPTYPIIQRLELLNKLINNIDKPEDLIKLLNCYNVLYDCSKELSSVIGFPVLLYLLSLLIQIVWTIYYVIFSFYYSFNYHIRVHKNKETIMLLLCIVTSFSLIMIADRVQCIKDKAQEFNHKMYKLMIKDNTNELCRNKKLKLHISTKKDIVFSAWGFFNLDYTLIHSMIAAASTYIIILIQLVPSKKEMFMRLNKNFEIGLESNMTNITHHQLDSAN

>GbueGr16b

METSNFDNARKSFGFLRFLGVFPLTNDFSISYKIFIYSLILNAGVAILYTNMTIDSVVRYYKQSIMKWFAVQSGLILISICVNFMCILKNRKSFKRIFEIINLVDQKIELKKDYNTCIRYLYTGTTIDICLWSILTIIKFGSSNFNKDFYINLLIYFITWIIFNSYAKSILLPVDVIKSRMTHIVKSIKKWKLTIEIENLSDCYYLLVDSSMICNNIIGFPIALYILIIFILLVNNTYSLYGFLMYDTLYTFFKYSLITIILLIIYLINLLQICWRTSSLIEAQEFNHKMYKLMIKDNTNELCRNKKLKLHISTKKDIVFSAWGFFNLDYTLIHSMIAAASTYIIILIQLVPSKKEMFMRLNKNFEIGLESNMTNITHHQLDSAN

>GbueGr16c

METSNFDNARKSYGFLRFLGVFPLTNDFSLSYKILIYSLILNAGVIILYTNMIIDSIVPHIKLSILKWIEVQRVLTVISICLNFTCFLKNRKSFKKMFEIINLVDSKIKIKTDYNTCINYLYTSTTIDLCLWLIMAIIKFDSPDFKVLDLSLSINFMTWIVFNSYVKSILLPVEVIKSRMIHIVKSIKKSKMNIEITDLSDCYYLLVDSSKICNNIVGFPMALYMLIIFLLLVRNTNGLYTILTYDTPYTYFEYSLALFIFLIICLISLIQTVWRTSSVIEKAQEFNHKMYKLMIKDNTNELCRNKKLKLHISTKKDIVFSAWGFFNLDYTLIHSMIAAASTYIIILIQLVPSKKEMFMRLNKNFEIGLESNMTNITHHQLDSAN

>GbueGr16d

MESSNFDNARKSYGFLRFLGVFPLTNDFSISYKIFIYSLIVNAVIIILYINTVIDCIETYKESIIKWFGVQGALTAISFCVNFTCILKNRKSFKKIFEIIKLVDSKIKVENDFYECMRYLYTSTTIDLCMWSIIAIIKLLSFDFKVINFSLSIYFMTCTLFNSYAKSMLLPVEVIKSRMSHIIKSIKISNIYFEKTNLSDCYYLLVDSSKICNDIIGFPIALYMLIIFFQLIINTYSLYGLLIHDTIKSFFTYSSLTFILLIIYLINLLQICWRTSSLIEKAQEFNHKMYKLMIKDNTNELCRNKKLKLHISTKKDIVFSAWGFFNLDYTLIHSMIAAASTYIIILIQLVPSKKEMFMRLNKNFEIGLESNMTNITHHQLDSAN

>GbueGr16e

MESSNFDNARKIYTFFRFLGVFPFKNDFTISYKILIYSLIVNAGIIMLYINTVIDHIKSYEESIMKWFAVQGALILISFCVNFMCILKNRKSFKTIFEIIKLVDSKIELKKDYNEYIRYLYANTTIDLCIWSTIAIIKFRSVDFKVIDFSLSTYFIVWLIFNSYIRSMLLPVEVIKSRMIHIVKSIKISKINFEITNLSDCYYLLVDCSKICNYIIGFPMTLFILIIFLKLVINTYGLYGLLIHDTNKTVYSYSLIIFFLIIITSSSLLQIIWRTSSLIEKAQEFNHKMYKLMIKDNTNELCRNKKLKLHISTKKDIVFSAWGFFNLDYTLIHSMIAAASTYIIILIQLVPSKKEMFMRLNKNFEIGLESNMTNITHHQLDSAN

>GbueGr17aJI

MLHLWNFEHLKGIYVFLRIIGVFPLSNSLSINYFLITYCLSLNILSLTVYIDTLVFTIFNIKDEVMKWVAIQGFLTVLTQIIYFIWCVKSRRKFCELFDYLVKVDYLIGRPLYRFPWRFVRSLTIELILLILLLTFDFKINHDVRISTYGEASHFFTWLYLNLHVKSILLPIDGIRSRMDYLVDLIKKKSINHPKISIISDCYYHLVDSSKVANSINGFPVAIFTLTQFIQIVIAWYLFYRYIYYYYDIEYLYNAIMTFIAITFFSYNLLYIVRVTSLLSDKKKLKLHISTKKDIVFSAWGFFNLDYTLIQSMMGAASTYIIILMQLVPSKHNLFSMMKEENSTAIV

>GbueGr17bJNI

VLIINFLAIEDEIMKWIAIQGFLTVICQIIYFIWCVKSRRRFREIFDYLVKVDDLIGRPLYRFPWRFVLYITIELILLILVMIFDLIYQKKRLFSNDKVLSYFFAWMYLNLHVKSLLLPIDAIKSRMDYLVDLIKKKSINYPKISIISDCYYNLVDSSKIVNNINGFPIAIFILTVFIQLVLTWYRLYRYIYNYKSDNNNLFRALKCLISITFFCFNILYIVRVTSLFSDKKKLKLHISTKKDIVFSAWGFFNLDYTLIQSMMGAASTYIIILMQLVPSKHNLFSMMKEENSTAIV

>GbueGr17cJI

MLNLLNFQNLNIIYGFLRIFGVFPYGNSLSTNYYLIIYYLSLNITLMILYMEALTFTMFNINDEVMKWIGLQGFLTVICQIIYIIWSVKSRRKFYEIFNYLVQVDDLIGVPLYRFPWRFVRYITIELILLILLLTIDFKLNFKLQLTTFGRASAYFFTWIYLNFHVKSLLLPIDDIRSRMEYLVDLIKRKPINIPKISIISDCYYHLVDSSKIANSINGFPIAIFTLTLFVQTVVTWYLFCTYIYYYYEIEYLYNAVMTFIAITFFSHNLLYIVQVTSLLSDKKKLKLHISTKKDIVFSAWGFFNLDYTLIQSMMGAASTYIIILMQLVPSKHNLFSMMKEENSTAIV

>GbueGr18aC

MKTFTSNYYKDVYLFYNIFRYFFIFPLRRDLSLSVKWLITSVVVITGSVILLFYLSSIDNEFHFISISRVWMVIYCLLMFVCIILNIIMIATFRNRLKEIYNIIEEFDDKIGKTDKNVGKTFYIFICLEILHCFYLIVFELYVYIQGVFSGIIFLSCFLFVLLNSQVKLLLLPLQPLYYRFIKLINLTKKYTKEQSPNTLSILSDCYFLLVDCSKLVNTIFGIPLCFYMLFLFKIIVITCFVTFKAIYFKLIGNEEFTIISILMILQIAFVFSNLIYIALQTSQVSDKAQEFNNRIYKLIIKDETNQLCQNKKLKLHISTKKDIVFTAWGFFDLDFTLIHS

>GbueGr18bC

MDDTEIHDYFNKKPSLLKIQKALYLFQIFGINIFKKDLNFGVKSFIYCLLMNLLIITFNAYMIFIHANTINATMMWTFCVGVSLSIIMYFIFLSRSSNKLKCILNKIYDFDNLLDEVQAFSYYRLMISVVFIDIFYCLFIFIFELSTSSDNYTGINITTCFTWMYLNFHSKHLFILIHPIIDRMIYLKKLIKKGPNSEDKLNKLSDAYYLLVDCTKILNKLNGPPILLFMLFVFTQFVTAWYSIYKILYWFYKSGYEFTASTATLTCHGFYLFFNLHSIVSVTSKLTQKAQEFNNRIYKLIIKDETNQLCQNKKLKLHISTKKDIVFTAWGFFDLDFTLIHS

>GbueGr19F

MIVRWKSGCEMTKVQFQGEVTSGSSGMFRWYKCSIFTWFKMLGCFPLNVVRSMIDISLLVYSACVQICLLVLYVWFHYQVQNRFTSEQSQVENFVQNTITFQMAFGLVFYFLNTIYNSEQILGILRELDSNDNWLSSNDFKWNCSMSYLLALCVDFCLSIIIVILSYLNDNTQRAVDSLSWIYLSLYIKQFTALLQPYRTKLDKLNDEVKLTACNLERLSERYNAVRRSCLAVNSSYSLTLVMYCLCLFCQLVVTAYNCSKHVIDYVVADVHDDLLHILSASLLPLILFCVNMLHIAHESSAVSEKSVSFNNGLYAKLMERNVNDIANIEKLRLDVSTNRNIQFTACGFFPLNYTLAHSMIAATTTYVVILMQLGPTTTDSSVSFVTISV

>GbueGr20J

MKAQSPLKTYKDFDRLTLFFKAVGLCPFDGCYSSFSAAWFVYSLVYHVVYIGSVVIFHVLYYYKHFDNNLMKVVSFAVTASNCASVVLTFCLTLFQRETLESVLKEVSCIGRRYDKTSCVGRPYLTFLFLDVVLFCVAFSFERSLSSDQVKMAFNYWMSIYLNLSTKQFTAVLQPVKLLLGCLKPLVRRSSSVKEIVQLARIYDRLADCCLKVNRTYSLALFVYFATCFCHLINTLFAFKILTDDCMHGKNCYVAAWSLYLTVSMFLNVIALVYECTTVADKGKKFIEQILEISLNKCKNPDVNFRIKTFLFMGRKIEFSANGLFILNKASLHSMMATIIAYLVIILQFENEYDEKT

>GbueGr21I

MNIHSKIFPVLLLMRITGLFPYDSKFQLSFLMAIISVILLCTPLGIFTYYIMTAEAVTSAQDELMVIVNKFLVFLYFVVIVTCFAYQKRFRITMSGFDSFDSWLSQSNFKANNRLLVFWIFYVLNCLLVTGILFVSYTDYELPFSSFEVSYWVLWLVVDLHMKKLQIILSLVEIRFSLLKKCLTDQPDRFVEFLNLYGILERVSASANQMFSVLLVIHTLVSWMSFLTESYAVVSLLISMLSKNHERRQYNIYMVLMTSTFFFMQLFTLAYQRERTAEKVSILMPLYLGQSDSSDWPTYNYVLMHLAKKKQIYLSASGFFNLDFRFVQSIVLTSTTYLVVLIQFYVESENSKSPTLTCVPTNISSLENNTMKNSVISSLVNIASFINKKVIN

>GbueGr22I

MSPKQYNIKNHVDTSVVRIFKLLNKIELFPYNDSLKTTKLDRFKYAVCLISIELIIICLRFLLKYVDKTFVKGSIKYKFDSFWINSQMILYHLFVVSIIIANILQLDNIYKVLLDLKMQLSTKEFNDCTFKSSLHLFLHKYGFIIGLAMFVIYMYTVLAYDMITSRESKWSYYITNILLSYTLTRIYIYQELICKLLKSQEIYLIQIDKVIKHNYKNNWTFTLGSVNNDIVDLLIFLEKQKEISVLLTYSLGLQIIIMYSLLLTFLFCHSYTFYLRLSAFPLGFKNLSILYMIMTLMFMVQKISFASANVKNQFKIEMTINRDADFGLIAYDCLELDVSIITKIISTVVTYLIILIEFREE

>GbueGr23a

MPTFNCNSFSSLSQTKLYVSNLFKFSTEVTALKLVFNIYSAFHLFPFDKGLNIVPFRFRASVAFTFLWLTVVIPVYLKRISKTLDSFDNHFFKYAVLDQAIIYLLSCAAVILRNILMINQYISIVKKLSFLRTRIKKEIYLKIIFDLKFVYLRFFCITWYLTYCLVYSHFYRSDADIILGFVRVYVFYNLYIVSELHFSFNTIKETHLKHLFYEFSYFRHNFTLNLTKEGNKFDSLVRHFDELIFLCYRSNNLYNFHQLIFIFSTSLGILIQLYFAISNFENFSSLKQPELIIAGVSYNVILMYSTVSAGVDIKTKAEKIIDLLQRLTRKREIISQKSTNFKIEMLISTRDKLEITVYDMFEVDLPLIITISSAVITNLIILIQFKD

>GbueGr23b

MNNHNKTNRTLFSKYNFLDERSALKCLFSSMSKFACFPLDMSLNKYSNGFRKVRSFLFLCIFSIIIVFVYMTYSFKQQSFLYDVGVITRFQKAAFFIQLAACFGLTYMLSFINSFKSGSLIQIINALKRIRQWLNRINPDLCLVYHIRSPSLRSFSFISSYSFFCIIISTLIYDLKNSCYIFFELYTAILLYMNVEIYLSFIHLIYIQIEDLCQRIKTSRGNGTKLNHLFLSVLKITSLCQNLNQTFNIQLLIEYFLLPLCIFIIIYMTSIVNYLALKDGWFHLAYLYGTGLWFTGLLLVLIFTSYGIKFRAEKIIDLLQRLTRKREIISQKSTNFKIEMLISTRDKLEITVYDMFEVDLPLIITISSAVITNLIILIQFKD

>GbueGr23c

MKLIFIKLTDIFYKVFTFIDELKIVKSLIWYWSRISLIPLLSGFKYNKLSLFLSILFGVLLCLVFNSVILSCQIGSINFLSNGFVKTIFTLQSLFIVNLSFAINFKNFIEHEKVLNIAKCLKTISLQLQTGLTMEYTITYCFNKYFWAILHISTNYGLFVYYNTEEGCQLIINWIYVYSSLRTILDIAMLVIPLHLIKNKSVYFCHDICRLYKYSDVQVLESHHFNCLTDLYSLQKRLLNLFSFQILIYICLLELIIFSHLYYVYFQFFEHNGHRLELILLVKNVLFNIYLLFDVILALSEARNEAEKIIDLLQRLTRKREIISQKSTNFKIEMLISTRDKLEITVYDMFEVDLPLIITISSAVITNLIILIQFKD

>GbueGr23d

MKIVLMKLTNKFYKFITFRNELKIVKSLIWYWSRISLIPLHSDIKYNKHGMLISILFQVLLHLFFNSLFLNCRSRRINLVTNRFVRSILELQSVFVLNLSFAINFKNIIEYEKVLNIARCLKTTSLQLQNGSKMEYTITYYFNKYFWAVLHVSTNYGLFFYFYGQKGCQLKINLIYIYICVRTILDIAMLLIPLDLVKTQSVFFCNHICRLYKYRNVQVLESHNLNCLTDLYSLQKRLLNLFSFQIFVYIGLLELIIFSNLYYFYNQFFEYNGYNFELILVAKNVLFYIYLLFDVILALSDARNEAEKIIDLLQRLTRKREIISQKSTNFKIEMLISTRDKLEITVYDMFEVDLPLIITISSAVITNLIILIQFKD

>GbueGr24aI

MNKSLNCLYFLSKLFLFPLNKSLKFNNFKKIRYLFIIIFMLSAGVILTIYSDNPNVFIKLPYDKLVLLLYVLTKFIVLSVICLINCFNIENICLIISDFNNQCNTLIFYGIKIIFELNCRYIRIIIFFLLTTHMTYYSMVFHFYKSFLIFNIKSIHLLHMTHIYFHVNFYFNIEMYLSLTAIQCTLLNELQKRIRNIANNDNLKFNQHFLEIAYSLLQTSYLINATFDHQFLTIVTSCGLYVLMHMYFSYILIDQTWEDQPILKTAILIVIFSFIAFYWTTASSHGITYKFKTEMFLSANDDFKITAYDVFEIRLPLIISMSSTIITYLIILIEFRKY

>GbueGr24bIP

MYKSLNFLYFLSKLFLFPLNKSLKFNTFKKIRYLLIIIFMLSAGVILSIFSNDLDEXFTKITYDKIVLLFYVLTKFVVLSVLCIKKSFKVQNICQIVSDFNNECNTLMLYGIKIVFEVNCQYGRTVIFSLATAYIIYYWTVPYFYNGFNILSIKSIDILLVTHIYFNLNFYFNIQIYSLTAIQFTLLNELQKRIRNIADNVNLKFDQHFXLEMAYRLLQTSYLINATFDHQLLIIVTSCGLCVSMHTYFSYILLDHTWEVQPILKTAILVVIFYFLAFYWTITSSNSITYKFKTEMFLSANDDFKITAYDVFEIRLPLIISMSSTIITYLIILIEFRKY

>GbueGr24cI

MNKPLNFLYFLSKLFLFPLNKSLKFNTFKKILYLLIIIFMLSAGVILTNLTERLANISIKLSFDKIVLSFYVLTKYFILSLVSMINCLKIDNICLIINDFNNQCHTLIFYGIKKNFKVNCQYVRLVIFLLITAHMTYYSMAFYFYTSFLILNIKSIYCLLITHVYFNVNFYFNVEIYLSLTAIQCSLLNEFQNRIRNIANNVNLKFCQHFLEMAYRLLHISYLINATFDHQLLFTIGICGLNVLLHIYFSYILLDQNLDSYPILRTTIFIVIFYLFAFYWITTCSNSVTFKFKTEMFLSANDDFKITAYDVFEIRLPLIISMSSTIITYLIILIEFRKY

>GbueGr24dI

MNKSLDLLYFLSKLFLFPLNKSLKFTKFKKIRYLFIVIFMLTSGVLSTILSDRFPNISIKLSYDKIVLSFYVLTKFFILSVVCMINFFKINNICLIINDFNNQCNILISYGIKIVFEVNYQYVRIVIFSVVIANMTYYSLLFYYYYSYLILTVKSIYCLLIAHIYFNVNFYFNVEIYLSLTAVQCTLLNELQKRIRNIANNYNLKFNQHFLDMAYRLLHISYHINATFDHQLLFTIGICGLYVLLHMYLSYILLDYTLDSKPILKTAMLIVIFYLLSFYWITTCSNSVTFKFKTEMFLSANDDFKITAYDVFEIRLPLIISMSSTIITYLIILIEFRKY

>GbueGr24eI

MNKSLNSLYFLSKLFLFPLNKSLKFTKFKKIRYIFIVIFMLTSGVLLTLLSDRFPNISIKISFDKLVLSFYVLTKFFVLSAVSIINCFNIDNICLVISNFNNQRNALIFYGIKIVFELNCQYVRMVIFSLVTAHMTYYSMVFYIYNSFLILTVKSIHCLLMTHIYFNVNFYFNVEIYLSLTAVQCTLLNEVKNRIRNIENNVNLKFNQHYLEMAYRLLHISYYINTTFDHQLLFTIEICGLYVLLNMYFSYILLDYNLDSHPIFKTTMLIVMFYLFAFYWITTCSNSVTSKFKTEMFLSANDDFKITAYDVFEIRLPLIISMSSTIITYLIILIEFRKY

>GbueGr24fNI

MKIHVYLKSFCLFIVYCKNSYALDNINEIINRLRKIHLLLAKEGLNKLYFDVKIENIRILFLILITLIISFLQFIYYILFYFNNKLKYFALFIIIFIIFIEVYLNAIVFLNSETYIIVGYLLKTYIDFFYRQLRELDKKCIKDEEKRRIYLFLHCLNQINLIVRLYSRTFGKQLLISIGHSGIYLCSLTFFVYTFIYQGKSKDQIVRFSIFLFNIILFGGNIILISKYTDDLNLEFKTEMFLSANDDFKITAYDVFEIRLPLIISMSSTIITYLIILIEFRKY

>GbueGr25IF

MNFLLKLLSCIYISYQFVINKNKNKQTFISILKYSMTLPLSKSQDEFKISKNIKTTIIFNLIYTLIKFSYFLFKYVANRNNLFELDFLTVRSLVFMWIMCFIISLWYYILKSADLIDIFNNINSLSDDIEGVQNKREIKCYPFIGNIIEYIIFTTLIVAVRNVNSNSFVVTFLMNILTAYDLLRYSLSIHLFTYVVFNLTQILTSINKVIKLFFYQNVMVKETMDLIFKNQLNLTENIYTINEYFYPLITLVLLSNFFFILHDLFYKYLFIREFVLSDLSYHIYVILWFVNIVKLLFGLIRSVFQLLNKIFATIITYLVILVQFRNEEEKKIVIASH

>GbueGr26a

MDLKKCFHLSQDTQFWLLSQFMFVLPIRKTKKHFVLTSHKRFIIFNTIYTIFVYILSFNFIYRTYNREDWLDFLIALLFTTTWVSAVLLTQWLYLLKTPDLIKILSNLTSVMNDINKLNHSINIINHSASFVYYFYICSCLILMFIRVLKLQMIYKTVLIFMCLSYKVIKCVITYEIYTYILHLLNCRIRAAIGVTHKILYSQTLYFKNIHNLLQIYFKLFRIADKINNCFVYEITLSLMSIMFSLMLDLYYEYLYFKYHVSIKIAFYHFSLLYWIIFYSFLTTRCYLTSSISTEQAAKLNDSLCRFISKQHDSPLAQSTIFKMEMILAVDNKIEFSAYGFKIDKPTFHSMLATIATYLVILIQFREDSSNS

>GbueGr26b

MNLKKRFNRSQETQFLLLSKFIFILPIRKKKCHFVLNSHKSFIIINTIYTISEYISSFYLMYRTYEDKRLDFQITLVFITTWISAVLLTQWLYLLKTPDLIQILSNLASVMNDINKLNRGINIINHSDLFVYYFYLCSSLILVFIRVLKSQMIYEAVIFFIYLSYKMFKCFITYEIYTYILHLLNFRINATLEVTHKILYSQTLYFKNIHNLLQIYFKLFRIADKINNCFVYEITLSLMSMMFSLMHDLYYEYLYFKYHISMKIAFYHFSLLYWIVLFSMLTTRCYLTSSISTEQAAKLNDSLCRFISKQHDSPLAQSTIFKMEMILAVDNKIEFSAYGFKIDKPTFHSMLATIATYLVILIQFREDSSNS

>GbueGr26c

MFKKIIFMNKLIIYSLALPVTNDFKLLDNSLKCLIFLSNIMITVLLYLTLPVRIGNDRDNLLDFKTSKITAIIMLTSISISSWFQLVKYKYITKIYNLTINLSNDSLNSYQSKIECFKCIFFLFIIIIGNVIRIINPIYTNERLLVFSVFTILHWKYFLSGEIYANISAFFTQLNQDLRRDFESLSSYNFRTYNQCINKFITDNYFKNYQNIIYFCSEFNNIFVVQYLAVYMSFVSMNLNNLYYQYLVFKGVLNNNNNNSINAQLSNGISSFNNLCIGSTIFLYTSQISLKAAKLNDSLCRFISKQHDSPLAQSTIFKMEMILAVDNKIEFSAYGFKIDKPTFHSMLATIATYLVILIQFREDSSNS

>GbueGr26d

MNLTSRLNKSQENKFWLLLQFTFILPINKTNSKFEITSRNYITIFYIYIILEYLKLYHFIYQVYGKSRFDFQISYIFLFTWISAITLTQWLYLIKTADLIKILNQLSSVMSNIKKIHRAIIIFNGSILSVYCIYVIYVFILFIVKLWQIPVFYEALIHFPLFYKVYKCIIIIEIYMYIIHIFNLNIKAIHKINKILNSKILNFKYIHILLIIHSNLLRTADKINSCFVYEVSLIVTSAMLSLVHDLLYEYLYLKYGITMDNIHYNFYLVYWIILYCFLITRFYLTTSTSTETAAKLNDSLCRFISKQHDSPLAQSTIFKMEMILAVDNKIEFSAYGFKIDKPTFHSMLATIATYLVILIQFREDSSNS

>GbueGr26e

MFKKTMFMNKLIIYSLALPVTDDFKLLNKPNKFIIFLSNIMITTLLYLTLPVKIGNSRKNLMDFTTSKITALIMLTSISILFWFQLIKYKYITQIYNLTINLSNDSLNSYQSIMECFKCIFFLFLIIIGNILRILNPLYKNERLLVFSVYTMLHWKYFLSGEIYANISMFFTRLNKDLRKDFESLSSYNFKRNIQNTNKIKTDYYFNNYLYIIYFCSEFKNVFVVQYLAVYMSFISITLNNLYYQYLVFKDLINNNSLVAQLSTGISSLNTLCIGTTIFLYTSQITIEAAKLNDSLCRFISKQHDSPLAQSTIFKMEMILAVDNKIEFSAYGFKIDKPTFHSMLATIATYLVILIQFREDSSNS

>GbueGr26fF

MIKKFLIYTLSLPLNKSFKFSSRNQKIFILLDLFCLFINATIISKYVRNQGMNFFDSTVFESVVYIWLLVIFPAFIYHELKAENIIDIYNNTFNIIGDFNYKINHIHRYILLKCFIYCFFLLPRILLDAFDSGGLYFPILVSVVYIYSMFRFYSIIETFLILIYLQSDFLNFCNRMCDSNLISKIDKIIVNLIRIDKLCLRLNQCCIPTICALSVAGCMSVFNHTSMAIAKFELGWLTCRKFSLQECFILWLQSYSISLIWLYLAVWIQWFTFIDTPSVADKAAKLNDSLCRFISKQHDSPLAQSTIFKMEMILAVDNKIEFSAYGFKIDKPTFHSMLATIATYLVILIQFREDSSNS

>GbueGr26g

MNMMDNDSKSLCYTHKYLKVTGSLPLNTKFEFYRVNSIIHTCVLIIFTVIPFIIMPLLVIKDKMLRFDQSVSLFMVFSWLILMFVAFWYQIYQYKAIIMIFNHLYTILNENKSFLNVDISYLYMKFISHIINVCTLLKFLYTYRGLRYRIFLISPMYIYIFTKMYLTIDTISILIRIWLKCISKIADKILNKYRNDFESNNIIHLISQFDDILLDIYDNFAVQIFCLTFAGFLSIFNNLYFQYMLFDGSVEALHSRKDLHCYGSLSECVLGHFLLYSLWLVLILRSQITMFFNPPQAATKAAKLNDSLCRFISKQHDSPLAQSTIFKMEMILAVDNKIEFSAYGFKIDKPTFHSMLATIATYLVILIQFREDSSNS

>GbueGr26h

MKNECPILFKTHRFLKLTRSLPLEKNFKFDRDKSLSSVYLLIIFTIISFFVMPLYIIEDEILAFDKIVSLFMVILWLILMFIAFWYQHFKNQAILNIYQNLYTILNDNKSFLKVDLSYLFVRFVAYAIYVCAVQAFSYTFGKISYKLLIIHPMYMYILIRYYLIIEVNSILINIWCSSIINIIEIVNNEQKCLKIDTILHLIIKLNDVLLKIYNNFDVQIFCLTLAGFLSIFNNLFFHYMLLDQAFGGLSGGKWLNCRISLAQCAVKLYFIYSWWLIMILNAQITIFFNPPQACSKAAKLNDSLCRFISKQHDSPLAQSTIFKMEMILAVDNKIEFSAYGFKIDKPTFHSMLATIATYLVILIQFREDSSNS

>GbueGr27a

MKYNIKKMEDNILVLKKTHKYLRLTHSLPLNSNFEIDCIRYLFSIYITIVFTIISFALMPTFVVTDKKLMFDRNYSFLMVSTWLILMFTGFWFQIFNYQSIISIFKEFNSIINGNKPFLNVELPYLFIKFVVSLFYISSLQIYVSGFREPTFKSFFIFMMYMFILVKYYLIVNINSILIDIWCKSITSINNFILNEQNHLFSVIIDAHLILIRKFNDIWCTMYKHSCVQIFCLILAGFLSIFNNLYFQYMVFDDSMLLLSSVTYKSCRGRLLRCTVERYISILWWLLSILSAQTSIFFRVPQASSKAAKLNDSLCRFISKQHNSPLAQSTIFKMEMMLAVDNKIEFTAYGFNIDKPTFHSMLATIVTYLIILIQFREESAT

>GbueGr27b

MLKILKIMMQKDFKILFKTNKFLRLTHSLPLNSQFEFDFVYSIISICLMVIFSVISFLMMPLHVLEENDILKFDRSVCLLMVCLWLILMFTGFSYQIVKFKLIISIFKDLKSIFTENKLFINVDLSYLFLKFVIYLIYICMLQNFIIGFQFLSAKSLLIYSMYSFILIKYYLTINTNNILIDIWLKSISKIIEIINITQINSYIVTFDEILNLIYEFNAILSNIYKHFSVQMFCLTLAGFMTFLNTFYLFYMIFNGAAITLSSGTYEDCRRQRLFECLSNPYSTYFWWLMMVLYAQLTIFFNLPKAIPMAAKLNDSLCRFISKQHNSPLAQSTIFKMEMMLAVDNKIEFTAYGFNIDKPTFHSMLATIVTYLIILIQFREESAT

>GbueGr27c

MIVNINYILFRIFDKMNNSGEILSKVQKYLKFTNCLPMQSLFEFDRVKSRKSTCLMILFIVVAFFVMPEHVLKDKLLNFDKDICLFMVALWSILMFTSFSYQLIQSKSIISIFKELHSILNENVSFINVDLSYLFVKLIVYFIYIFNLQISIIGYYFSSLNNFLIHLVYSFMLLKYYLNIDTYSILIDIWSKCLFNVIKVIKKERIYLQTAKIEGILNLLNRFNDILYEVYKHYSVQTFLLTTAGFISTCNILYLFYMTYDDLIVSLSSGRYKNCKSSLLLRFVESYLSYSWWFGMVCHAQVTIFFKPSQANSKAAKLNDSLCRFISKQHNSPLAQSTIFKMEMMLAVDNKIEFTAYGFNIDKPTFHSMLATIVTYLIILIQFREESAT

>GbueGr28a

MKYNIKKMEDNILVLKKTHKYLRFSHSLPLNTNFEIDRIRYLFSIYITIIFTIISFVLMPIYIITDKKSGFDRNSSFFMVFTWLILMFTAFGFQIFNYPSIISIFKESNSIINGNKKSFLNVEQPYLFIKFILSVFSICSLQIFIRFSSYRDLSFKSIFIFMMYVFILFKYYLIININSTLIDIWSKSITNINNFILNEKNHLFPVRIDANLILISKFNDIWCTMYKHSCVQIFCLILAGFLSIFNNLYFQYMVFDGSMITFSSVTYGACRGRLLRCTVERYIANLWWLLSILSAQTTIFFRVPQASSKAAKLNDSLCRFISKQHNSPLAQSTIFKMEIMLAVDNKIEFTAYGFNIDKPTFHSMLATIVTYLIILIQFREESAT

>GbueGr28b

MNSLFEFDRVKSRKSTCLMILFIVVAFFVIPEHVLKDKLLIFDKDICLFMVALWFILMFTSFSYQLIQSKSIISIFKKLHSILNENVSFMNVDLSYLFVKLIVYFIYICNLLISDVGYYFSNFNNFLMHLIYSFMLLKYYLNIDTCSILIDIWSKSLFNIINVINNTAEIEEILNLLYRFNDILYEIYKTYAVQTFLLTTAGFISTCNILYLYYMTYDDLIVSTSSVRYKNCESRLMLCFVESYLSYSWWFGIVSHAQIVIFFKPSQASSKAAKLNDSLCRFISKQHNSPLAQSTIFKMEIMLAVDNKIEFTAYGFNIDKPTFHSMLATIVTYLIILIQFREESAT

>GbueGr29

MENNVLVLKKTHTFLRLSHSLPLNTNFEIDHIRYLFSICITIMFTIISFAIMPTYVITDKKLNFDRNSSLLMVTTWLILMFTGFWFQIFNYKSIISIFKEFNSIINGNKSFLNVDLSYLFIKFIVSVLYIHFLQILMRGYRNLLFKNIFIYMMYIFILLKYYLIVNINSMLIDIWSKSINNINNFILNEQNHLCPVKMDENLSLIRKFNDIWCTMYKHSCVQIFCLILAGFLSIFNNLYFQYMIFDGSIILSSEPFKACRRGLLECTVERYVANVWWLLAILSAQTTIFFKVPQASSKATKLNDSLCRIISKQHNSALAQSTIFKMEMILAVDNKIEFTAYGFKVDKPTFHSMLATIVTYLVILIQFREESAT

>GbueGr30aF

MNPHKDIKYLSKILFWTMSLSLCTDHIQFCKYHRNKFKLIYFNILYSILTVLVILSHYNHTLKDKFDTFTSNIFIILWVIQSIIIFWLLIVKTESISNNVINIFSIMGYLKQKHLYKTIYSYQYINIKIRIILFTIYLSTYFILKFIFNYNYTWKIVIVYVLQFYALLRRMLAVEVYTFPIRLYSQIINSLNWNIIQMSKSPNFQIKINNKFWIYFNKLIYTNDKMNATHVLSLLVIISSQLIVIIEFLFYQYLMFISKNLNIKYAKYINAFSLSKLCYLQVSTFYYPSEATNKAAKLNDSLCKFIARQHDVPLAESSIFKVEMLLAVDNRIEFTAYGFKIDKPTFHSMLATIATYLVILIQFREDSSGA

>GbueGr30bF

MNFINSYKDKNDLSKILFWTMSLSLHSEQSNSRKIEQNKLKIIHFNIFLYMLTFLILLLQYNEKNINDSFDSMFSFIFIIIWVFENVLFFWLLIIKTDSISNILIEIININTDLNENNYYKNNHFLKYLNIKIRIILYAIYLSILMLMHIILRLNNKSFITYILHFYCSFRRAISVELYTYLIHEISQIIDCVSSNIDQMSKSKNQIKNKFWIYFHKLFSINEKINDTYDFSCLIIILSQLIVIIEFLFYHYLIVFINKRRYSNSKFNIKYEELINAMWLSKLCLLQVLIFYYPSEAIIEAAKLNDSLCKFIARQHDVPLAESSIFKVEMLLAVDNRIEFTAYGFKIDKPTFHSMLATIATYLVILIQFREDSSGA

>GbueGr31a

MDPKLLYKIKRFFILTQIYPLDSDFQISKIKKNLTFCLIFLTLGISVYINASPDYMLYTEKPLFDIAASRMISSSWLILTFFVYIYDVIRADIIIIHYNLLFKIKTFPRISESLTRLIYSCYQSMLIKFLCFTSLCLISNACNVLNNYFITEAIILFFTFVFTILKFYLIIEAYIIIVNIWSAFLEFFSNEINCIHIYNNVFDLKRIDDIIKNIRKAKTIVEKMNELFLFPITCLGLSGFFGVFYDLYLFYLQLISSMGIDLVSLNFKQKIICISLNLWILMNMLVLWCLCVSPSNADLKAAKLNDSLCKFISRQHDEPLAESSIFKVEMLLAVDNRIEFTAYGFKIDKPTFHSMLATIATYLVILIQFREDSSGA

>GbueGr31b

MNIINPYKDLKDLSKILFLTMSLSLHAEQSNSRKNQQNKLKIIHFNIFFTMLTFLMQLLNYNKTTIIESFETKTTFIFAIIWIFENLFIIWLLIIKTDSISSILIEIVNNTAHFNDNNYYRNDYLINYINIKLRIILYGIYLSFLILLLFIYDRGNKVLIIYLLHFYTLFRRTLSVELYTCSIHAYSQIIDFLISNIHQMSKSRRSLTKIKNKFWIYFHKLFSINEKINNTYDLSSLIIISSQLIVIIEHLFYHYLFVYAFKTRHTNENLNIIYVYINALWLSKVCLLQVWTFYYPSKAINKAAKLNDSLCKFISRQHDEPLAESSIFKVEMLLAVDNRIEFTAYGFKIDKPTFHSMLATIATYLVILIQFREDSSGA

>GbueGr31c

MNNINLCKNIKDLSKILFWTMSLSLHAEPSNSRKNQQNKLKIIHFNIFFTVLTFFILLLHSNEININGSFDSMFSFIFIIIWAFENVLFFWLLIIKTDSISNILIEIININTDFNENNFYKNRHLLKYLNIRIRIILYAIYLSNILLIHIILHLTYKSSIMYLLYFYCSIRRTLSVELYTYLIHEISQIIDCISSNVDLMSESQRFLTKIKNKFWIYFHKLFSINENINDTYDFSSLIIIISQLIVIVEFLFYHYLMVFINKRRYSNSNFNIKYEEYVNSMWLLKICLLHVLIFYYPSEAMIEAAKLNDSLCKFISRQHDEPLAESSIFKVEMLLAVDNRIEFTAYGFKIDKPTFHSMLATIATYLVILIQFREDSSGA

>GbueGr31dN

SENFDAIYNYIITFVWVGEHLIRNIFQVINADSFIKVLKRTVNLSKYTLYPNSRDGKHLKFSNIEMRIYILLTYTLLINIIKLIYFKIISSNDIIQCTIYMYILLRSLLSVELYICPIKSLLHVIKAITFTKDINLYVISGNFNVILKIYSFVEKINISYCTTLISIILSNYFLFLEYLFGRYIIFICHCPTEYYKFGNFFDGLYLASWVCIFFYLHTSIFYYPSEVVKCAAKLNDSLCKFISRQHDEPLAESSIFKVEMLLAVDNRIEFTAYGFKIDKPTFHSMLATIATYLVILIQFREDSSGA

>GbueGr31eI

MNPYKEVKYLSKSLFWTMSLALCTDQIQRCKNHRNNLKTIYFNIFFNILTVLVIISHYNHTLKDKFNTLTSYIFIILWVIQSIIIFWLLIVKTESISNNFINIFSIMGDLKQSHLYKTIYSYQYINIKISLVVAKSLSRFQWTTLNFQIKIKNKLWIHINKLIYTNDKMNETYVLSLLLIILSQLIVIIEFLFYQYLMLISKNRLKNTTNNQKYVKYINAFFLSKLCYLQVSTFYYPSKAINKAAKLNDSLCKFISRQHDEPLAESSIFKVEMLLAVDNRIEFTAYGFKIDKPTFHSMLATIATYLVILIQFREDSSGA

>GbueGr32aNJ

IKSYDFSSENFDAIYNYIATFVWVGEHILRYIFLVINADSLIKNLKTTVNLSKFILHPNSRDSIYPKFSNVEKRIYALLTYTILIIIIKLIYFKIISTNEIIHYAVYIYILLRSMLSVELYICPIKSILQVIKAIKFTKDINFYVISGNFNVVIKMYSIVEKINITYSKTLISIILSNFILFVEYLFGRYIIFICQCPTKYYKFGNFVDELYIASWLIITFYLHTSMFYFPSEVVKSAAKLNDSLCKFIARQHDVPLAESPIFKVEMLLAVDSRIEFTAYGFKIDKPTFHSMLATIATYLVILIQFRQDSSGA

>GbueGr32bJ

MDHKLLYKIKRFFILTQIYPLDNDFQISKIKRNVTFYFILLTLVIIVYYNASSDYKLYTKKPIFDIAVSRGISYSWIILTFFIYIYDLITADIIIRQYNLFFKIVFPPIISESLTRLIFSCYRRTFIKFLCFTSLCLISNACNVLNDFILKAIVIFLTYVYIFLKFYLSIESYIIIVNIWSALLEFFSNEINRICMHKCLLLKKFNYIIKNSWKAKTIVEKINELFMFHITFLGLSGFFGVFSNLYLVYLQLITFMRIDLVYLPITQKVFFIALNLWILMTMSVFWYLSASPSYADLQAAKLNDSLCKFIARQHDVPLAESPIFKVEMLLAVDSRIEFTAYGFKIDKPTFHSMLATIATYLVILIQFRQDSSGA

>GbueGr32cJ

MKNNSFFSYNPLDTPFSKFRNFFILTQTYPLNSNFQFSKLRRNLSLCLMIIAFILSVDFMPTFKVISNKSFFDSNAISLIIYLWCSLIYFVFFYDLFLKQTMLKRFTLLLKILEMWWFSNLNCKHLTDSLLCTYKSLIVKFSIFFFNIFILNIRAFVQRETVKESIYVSFVYMYCLLKYFLVLESYLIVINLWSLFLNMFVIDLNSYIFYNSLNLNKLNNFVETLTAVKWKCLVEDINELFLIQLTMTILSGFLGVYHSMYFHYLQNNIIHGRAFLKLTIWEQIYSFAAKLNDSLCKFIARQHDVPLAESPIFKVEMLLAVDSRIEFTAYGFKIDKPTFHSMLATIATYLVILIQFRQDSSGA

>GbueGr32dNJ

VNLSNNILNPYLGDDKYATFSNIELRIYILFTYTILLNIIKLVYFKKVTTEEIIYYLVYFYILIRSLVSVELYICPIRSILQVINAIKFTKVINLFVISGNFNLILKICSIVDKINITYSTTLILTILSNIILFVEYLFSRYIIIICQCATEYYDFGNFVDELYIASWICIVVYLQISIFFFPSEVMKSAAKLNDSLCKFIARQHDVPLAESPIFKVEMLLAVDSRIEFTAYGFKIDKPTFHSMLATIATYLVILIQFRQDSSGA

>GbueGr32eNIJ

ASRSDHMFYSKKPLFDIVASQLISFSWALLTFFIFIYDLIKADITVRHYNLFFKITFPRIISENLSRLIFSCYRSMLIKFLCFISLCLISNACNVLTIYSILKAIIHFFTYLYITLKFYLSIESYIIIVNIWSAFLEFFSNEINCIRIYNDLFDVKKINYIIKNLWKAKTIVAKINDLIWVTLYIALKAAKLNDSLCKFIARQHDVPLAESPIFKVEMLLAVDSRIEFTAYGFKIDKPTFHSMLATIATYLVILIQFRQDSSGA

>GbueGr32fJP

AVKFRKNKLKIIYFYLIFITTTILVVKLHYNDKIRDSFDSMSSYIFAMIWICENFLIFWVLVIKTDSISDILTDIVNIPIDFSKNNYLRNCYSNRSTNIKIKINLYSITCHFZCZCIFFGIVTLECYISMRYSFTPHLGVRYQLNVELYTYTIHVYLSIIDGLNSNINLMSKSSRFLIKVKSQFWIYFPKLFATNEKINNTYDLSLLVLILSQLIVKWNIYFIIILRFLLKGGLVVLCFIFISIAAKLNDSLCKFIARQHDVPLAESPIFKVEMLLAVDSRIEFTAYGFKIDKPTFHSMLATIATYLVILIQFRQDSSGA

>GbueGr32gJ

MDPKLLNKIKRFIIINQIYPLNNDFQISKIKRNVIFCLIILTFGITVSIDEAPDNLMFTTAKKSFDIAASRLISYFWLLLTYSVYFYDAMTADITIRLYNLFFNIKSYTDINCDNLTRLVNSCYRSILLRYLWVTSLCFISYGINSLYYYSILKAIIAFIFYVYFNLKFYFTIESYVIIVNLWSAFLEFFSNEMNCENNNSFDLQKVNDIIKNISKAKTIVENMNDLFFFHLTCLGLSGFSGVFSSLYLIYLHTSFFVGLDFTYLTIIQKVTFISLNLWIYMTLSVLWFMNTSPSNANLQAAKLNDSLCKFIARQHDVPLAESPIFKVEMLLAVDSRIEFTAYGFKIDKPTFHSMLATIATYLVILIQFRQDSSGA

>GbueGr32hNJ

CKNNRNKIKIIYFNTIFSILTVLILVIHYSDKINDKFEAVTSHIFIIIWVFESLMIFWLLIVKNESISNNLINICCIINDLKQNNTYKTNNSYKYVNIKIRIILFTIYLSFSMIINCLWNHTEEISIIHLLQFYALLRRMLAVEVYTFPIRLYSQIIDCLNSNINQMSKSLNSFIKIKNKFWIYFFKIIYRNEKMNETYDFSLLVIILSQLIVIIEYLFYHYLMFTITNRPTNPVIVIKYEEYINAIWLSKLCYLQVSTFYYPSEAINNAAKLNDSLCKFIARQHDVPLAESPIFKVEMLLAVDSRIEFTAYGFKIDKPTFHSMLATIATYLVILIQFRQDSSGA

>GbueGr32iJ

MQFQHNKKYCSITPFHKIRKYFFITQIYPINNHFQLSKISWILSLFIILFSFIISILFIPTFAINSHKRYFDINASQLFIYIWFILIYSVYFYNLLKAEMILKKFTLFLKIIEMWWFSNSHCKYLTKLISINYRTMIVKFIIFSSNTIFMNIIVTTNHVLFKEVLIVALMYLYSLIKFFLAFESYIVVVKLWSIFLNKFSFDIDSKLINRFVNEMYKFDLTELNRFMTTSIILRCSVEEINELFLFQLTFLMLSGFLGIFNSVYFHILKINIFHGREFFKLSILEQITSLSVNMCTVVYIMVMWVSFASPSKANWQAAKLNDSLCKFIARQHDVPLAESPIFKVEMLLAVDSRIEFTAYGFKIDKPTFHSMLATIATYLVILIQFRQDSSGA

>GbueGr32jIJ

MRTDSKFLRKIKRFFILTQIFPLNNDFQFSKIKRNVTFCLMILTVGITFTAHAIPIYLLSTKKPLFDFVASRLFLYFWILLSYSVYFYDTLAAKITIRQYNLFFQIKLFSNINCENLIYKYYQSMKIKFIYFIFNIFISNVCNGLDRGGIIEGIIFFMTYFFISLKFYLTIESHIIIINLWAVLSEYISNEINRRNRSSSDHTNIDYFVKYIWKTKRIVEKINDLAAKLNDSLCKFIARQHDVPLAESPIFKVEMLLAVDSRIEFTAYGFKIDKPTFHSMLATIATYLVILIQFRQDSSGA

>GbueGr32k

MDPKLLYKIKRFFILTQIYPLDNDFQISKIKRNVTFCLIILTLVITVYINASPNHMLYRKKTLFDIAASRLIASSWILFTFFIYIYDVITADITIRHYNLFFKITFPRIISENLSCSIYSCYRAMIIKFLCFTSLCLISNVCNFLNNNYSFLKALILFFTYVYTTLKFYLSIESYVIIVNIWSAFLDFFSNEVTCISINNNVFYLKKMNYIINNVWKAKIIVEKINELFMFHIICLGLSGLWGLFSSLYILYLQLVSFKRIDFVYLTITQKVILISLILWICMATTVLWFMNVSPSNADLQAAKLNDSLCKFIARQHDVPLAESPIFKVEMLLAVDSRIEFTAYGFKIDKPTFHSMLATIATYLVILIQFRQDSSGA

>GbueGr32lFP

MNILNSCEDIKDLSKILFWTMSLSLHTEQSNCRKYQLNKLKIILFNVFFTMLTVLKLLLALSKNNQNISFDSKTISVFGVIWVFENLLYFWLLIIKTDSISNILTDIVNIIAYFKQNNCYRNDFSNKYININIRMILYAIYLYFLLFMHFLYERGNKDLIIYLLHYYGFFRRRLSVQLYTCSIQVYSQIIDCLTSHIDNMSSTRFLSKIKNKFWIYFDKIFSINEKNNYTYDFTLLIIIISQLIVIIEYLFYHYLYVYVPKTRHTNAILNFKYVYINGLZILMFVLLQVLTFYYPSKAIDKAAKLNDSLCKFIARQHDVPLAESPIFKVEMLLAVDSRIEFTAYGFKIDKPTFHSMLATIATYLVILIQFRQDSSGA

>GbueGr32m

MNPYNEIKDLYKILFWTLSLSVHSEQSSSRENQQKKLKIIIFNIFLNIQLFLILLLHYIENNVNASFESKSTYIFAIMWAFESILYFWLLIIKTDSISIILIDILNVNSDLKHNNYYKNKNRHFLQNLHIKIRIILYAIYLSILQLMHFIFDLRKKSFMVYLLLFYCSLRRTLSVELYTFTIRIYSQNIDWISANIDRMPKTSKVLIKIKYTFWIHFHKLLSLNEKINNTYDFSLLTIILSQLFMIIEYLFYHYLIVFVNKREHRNTIFNFKHAEYINALWLLIICFLQVLTFYYPSEAINKAAKLNDSLCKFIARQHDVPLAESPIFKVEMLLAVDSRIEFTAYGFKIDKPTFHSMLATIATYLVILIQFRQDSSGA

>GbueGr33

MLTTIIFRNKHLETFNEKVIHLKYCLFIAKTVPVDSSFKYCSFYYKCSLLELIFFTLINVIYMPTFCMKYRNNKFDAPIIGVMIVIWLILISWTFFDNLIKSKDIIKLFDKSFELMNRMFGDDIDRTQVNIRLFLGKYLILLLLVIFMDTCLIIHLKGYLLSIIVFPMYTYTLGRYFVIIETHSMLIDFSIKCIDYLSESIECKLDSLFKRTNETIDILISNAMKIDILLRKVDKTLTFQVFLIASSGFLSLLTNFYLHYITVDNIFDRMKVMNGLSFGFRFDPGYIAANTIWLFFIGAMQLKIFITPSNATTVGAKFYDSLCKLISKNSDYTLLSDSSIFKIEMLLALDQKIDFTSFGFKIDKPTFHSMMATIATYLVILIQFRDSADI

>GbueGr34NI

IINFKIMTTQRIIYFFINLFITFLRPVVSVELYICLIKSILQLIKAIKFTKNINLHVISGNFNVILKIYSIVDKINVTYSTSLISFFLFNFIFFVEYLFNRYIISICQCPTEYIYFESFIDELYDASWVCIAFYLNTSIFYFPSEIVKCFKVEMLLAVDNRIEFTAYGFKIDKPTFHSMLATIATYLVILIQFRKDSYGA

>GbueGr35a

MNLWTNFKRYFQFHSEKDCLKPLLYLLQFEMFPIDTDLEYSKRKVIYSFFYLNLTGLVLISYSYVTMDKLLDIIIGNMFPFQRKAIFFQLNALVVIGWFITLVNCISSQSIFRVFQHLCQIRVNLNRINLFIILNMNTFFIRICYWIILFTVSLSMTVFHFSSINFILLFFVQFYSVTRLKLCYELFNSVINIIKVNLKCCNKFMKNTNLNVNNRSRFQIILNSIDNLFKLNPDISLESFGIQIFLIIYLYSLFVFIHMYIAYLICNLPFNMELSSVLPGLLYAATIVLSVITSISRLYDKNENIRDTLFRLLRKPEMQLESSLYFKTEMFLRARDEYVITAYDVFEIRLPLIISVSSAIITHLIILIEFRK

>GbueGr35b

MNLKSYFKSYSQFHSEIDCLKPLLYLLKFEMFPINTDLEYSKTKLVYSLFYLTINALVLFYISYFSMDNYLHTFLRDSFYVRIVSFQLSALTSLSWFNSLLNCLSSQSIFRIYRHLRQIRIDLYRINIFIIYSMNMFIIRIVYGCIFCLVTAISTFKYIFYVYYILITFVRFHSIARLQFSYELFDSVINIIKIDLDFFNKLLKNPNFNVTKRYRFQIILNSIDNLFKMNSDISVVSFGIQIVLLIFIYCLFVFTNSYMLYLTNIGKFEVDNFSLIMSILYSMTIVLSVILSISNENIRDTLFRLLRKPEMQLESSLYFKTEMFLRARDEYVITAYDVFEIRLPLIISVSSAIITHLIILIEFRKGLTYK

>GbueGr35cN

DSFQISAVSFQLSALRYLSWFNSLLNCLLSRSIFRIYRHLRQIRIYLNRIQIFIIFNMNMFIIRIVYVFIFCIIKFCIALIYIFNFYDILLFFVRVHSIVRLQLSYELFDSVINIIKMYLDFFNKLLKTPNFNVTKRYRFQIILNSIDDLFKLNSDIIVSFGFQIFLIIFLYSLFVFTSLYIIYLTNNLKLKAEKFSLITSILYSMAIVISVVISISHLTYKNENIRDTLFRLLRKPEMQLESSLYFKTEMFLRARDEYVITAYDVFEIRLPLIISVSSAIITHLIILIEFRK

>GbueGr35dI

MNLKSYFKTYFQFHSEIDSLKPLLYLLKFEMFPINLISLSWFNSLLNCLSSQSIFRIYRHLRQIRMHLHRINMFIIFNMNMFFIRIVIGFVFCIVSFIIALTYTFKVYDILITLVQFHSIVRLQLSYELFDSVVNIIKIDLDFFNKLLKNPNFYVSKRNNFQIILNSIDNLFKINSDISVVSFGIQIFLIIFIYCLFVFINLYMIYLTNIVKLKIDNLSFIMSILYSMSIVLSVIISISRLTYKNENIRDTLFRLLRKPEMQLESSLYFKTEMFLRARDEYVITAYDVFEIRLPLIISVSSAIITHLIILIEFRK

>GbueGr35eI

MHIKSYFKTYFQFHSEKDCLKPLLYLLKLEMFPINTDLEYSKRKIVYSLFYLNLTGLVFMSISYFTLDKFLHTFLSGSFQISAVSFQLLALIILSWFNSLMNCISSQSIFKIYQELGQIRMNLNRINLFIIFNMNMFITRIVYGFIIGIVGFFMALSYSFNIYNILISFLQHLYSLMVFSNLYMIYLANNQKLEVDHISLIMDLLYSITIVLSVIISVSLLTYKNENIRDTLFRLLRKPEMQLESSLYFKTEMFLRARDEYVITAYDVFEIRLPLIISVSSAIITHLIILIEFRK

>GbueGr35f

MNLWTNLRYFQFPSEKDCLKPLLYLLKFDMFPINTDLEYSKSKRVYSLFYLNLFGLVLISYSYVIMDKLLDIIIGNIFLFQRKAIFFQLNALVAIAWFISLLNCILSQSIFRVFRQLYQIRMHLNRINLFIILNMNMFFIRIGFWCILFIVSFLLMSSTYLSNINFFLLCTLQFYSVTRLQLSYELFNSVINIIKTNLYLINKYIKKPNFNVTNRSRFQIILNSIDNLFKLNPDISLESFGIQIFLIIYLYSLFVFLHLYMTYLIRNLPFNMEILSVLMGLLYAVTIVLSVITSISRLYDKNENIRDTLFRLLRKPEMQLESSLYFKTEMFLRARDEYVITAYDVFEIRLPLIISVSSAIITHLIILIEFRK

>GbueGr35g

MSNNMILWTNFKSYFQFHSEKDCLKPLLYLLKFDMFPINTDLEYSKSKMIYSLFYLNLTGLVFISYSYVTMDKWLDIIIGNTFPFQRKAVFFQLYALVAISWFISLVNCILSQSIFRVCRHLCQIRANLNRINLFIILNMNMFFIRIGSWFILILISLPMSAIHLSNVNFILLSCVQFYSVTRLQLSYELFNSVINIIKINLDSLNKFMKTSNFNINNRSRFQIILNSIDNLFKLNSDISLESFGIQIFLIIYLYALCVFIHLYAFYLMCNISTKIDSSNVLTGLLYAVTIVLSVITSISRLSNKNENIRDTLFRLLRKPEMQLESSLYFKTEMFLRARDEYVITAYDVFEIRLPLIISVSSAIITHLIILIEFRK

>GbueGr35h

MNLWTNIKSYFQFHSEKDCLKPLLYLLKLEMFPINTDLEYSKRKMVYSLFYLTINALVLLSISYFTMDNYLHTFLIDSFQISAVSFQLYAMLILSWFNSLLNCLLFRSIFRIFQHLRQIRVHLNRINIFIILNMNMFFLRIVYGFLFCIVSSYLSFSYYLNIYDILINFPQYHSITRLQFSYELFDSVINIIKIYLDYFNKLLKYPHFSFTKRSEFQIILNSIDNLFKLNSDISFKSFGIQIFLIIYLYCIFVFVNLYMIFITNNRKVEVDFLSLIMGLIYSMSIILSVIISISRLTYKNENIRDTLFRLLRKPEMQLESSLYFKTEMFLRARDEYVITAYDVFEIRLPLIISVSSAIITHLIILIEFRK

>GbueGr35iN

IILNIKMFFIRIIYGFIFCIVSFFMSLTYFSNIYYILITFPQFHSLASLQFSYELFDSVINIIKIYLDYFDKLLKTPNFNFNKRNRFRIILNSIDKLFKLNSDISFESFGIQIFLILFLYSLLVFINLYMIYLANNLKLKLDFLSLTMGLIYSIIIVLSVIISISLLTYKNENIRDTLFRLLRKPEMQLESSLYFKTEMFLRARDEYVITAYDVFEIRLPLIISVSSAIITHLIILIEFRK

>GbueGr35j

MDLYTKAIKTMNKLLNKLFSNYLKLKSERESLKILLHLYKCELFPINTNLEYCRIKMIKSLIFVNFTVVIFFIISLTTMNEYLTFINDEFKRHSLSFQINSVIFLAWFTSLLNCIMSEMISVVFKQFSLIRLQLLDIHIRVIFNINIFLIRLCFMIFLYVCSLCISTMFFLELNDTLISFTQLLSITRLQLSFELFDSVLNLSKTYLDFIQNHLYDIEKSYRVNHSKYKVILNSLGKLFDLYDRINDIFGLQVLIMIFLYSILVFMHLYMLYLIKDIQTPEDHLTISLGVIYSVLTVSSIITFTSSLTDKNENIRDTLFRLLRKPEMQLESSLYFKTEMFLRARDEYVITAYDVFEIRLPLIISVSSAIITHLIILIEFRK

>GbueGr35k

MTSLNEKENDLYKLLKLIYSISDLYLYPLNYNKLPKFKIYGSIALIILVNLLIIFLNIEYKWIFNPYTSVEYDKTVFVIHTVIKHYFLTMIHFKSCFQSSRIKFLIDNLEFISRSLSSYGFKVDFRLNDIRIRLIIFACLTFLAIYNGCTYYFTQFDTIAMIFGILLLLVFHCYLFNTLYFNAELFLTLGYIQNVFLNFLKSRITYTEDFHRLNYFLNISDLLVKVNRSYNVIFGQQLLMILMCNEFYLLLHFFFLYSLIDGDLSVGNVLMTNSLFQGFIFYFVIVHSAKLAHKNENIRDTLFRLLRKPEMQLESSLYFKTEMFLRARDEYVITAYDVFEIRLPLIISVSSAIITHLIILIEFRK

>GbueGr36

MTSLKSKENDLHTLLKIIYSISDWYLYPLNNQVSANKFKIYCSIALIILVNLLIIFVNIEYKWLFNPYTSVEYDKTVFVIHTVIKHYFLTMIHFKSCFQSSRIKCLIDNMEFISRLLSRYGFKVDFRLNDIRIRLITFACLSFLAIYNGFAHYFIQFDTIAMVFGVLFVLLIQCYLFNTLYFNVELFLTLGYIQNVFLNFLKSRITYTEDFNRLDYFLNISDLLVKVNRSYNVIFGQQLLMTLICNEFYLLLHFFFLYSLIGGDFSLGGVLMTKSLFQGFIFYFVTVHSAKLAQKNENIRDTLFRLLRKPEMQLESSLYFKTEMFLCARDEYVITAYDVFEIRLPLIISVSSAIITHLIILIEFRK

>GbueGr37aIC

MYVKKLLRKCFPKSDTNDFYVFKFILKCFSIILIFPTTKFIFYIIIIFHSVVTFSLLLTYYLLKGSFDVYKSDLNSYDLIVMMTNHGLLIIVFEAVLFLNIIYSKRILNIFNQYEKLNEDLQSSGCNVNYSYFWFYFRFVIFLGGGAYEFILLYKSILRGPSHPYFIGIKEIFIYFYVSSVVHAEEELYLVTARTQIIFLNYLADNYEELTLYRFKHYKDSTLSSLHYNNFKRIYSIINQLWDINTEIKVLYGPLFATIILFYSYTILNKCYFKYTAYYNFNNQHLKHIFAINNELSDMYYIVFITLLITSSAQIKQEFQGEMILHVKDPLILTAMDIYEVDNSLIIK

>GbueGr37bIC

MKTNYLKKYFPKTVFSNLYVFNFLFHFLSKTLCLPATASLQFSKLRLYVMIILHSTILVYLLIKYYKIITSDKTEMLSSKYDLHVIHANYLASFIVYELSIFINCFYSKRILILFNYYKNLNKYLINLGCEVNFNFSSFYIRFIIFFVFGLFYSCITIFNTLTSQSSHPIFVDPMLTAINCYVVAVIYLTSEIYLIATKTTKFYLRFLVDLYQKITVENINNLKDKDMNKIHSLLINIWVVSNEWNRVFGPYLATIIPFLSYALINKLYFDYFVYTYNLYLPKFIKKSGILILAKVYYLVHILATVHSSSKIKQEFQGEMILHVKDPLILTAMDIYEVDNSLIIK

>GbueGr38aC

MHSNDNELKGLQFPLFLLSIIIYYPRNNTASHFRKWFSFLFNNIIVGIFVVINYLDFSGFVSIIPRYEQIICNALNISIIAFAVLSSTNIVKSKNIYDLFNNIQGINELINNSKPKSNKADIILLVKVVSHIFYLIVYTTLIILTETSLDLKKYIYIIFGLYFSILAYAHLNLIFAFEDIVDMLLSIIHDKLSIYLTPVENSRDLNKINMDICIYCFDRLCDVNNQLNACFGLPYLMIITMYFFFILFECYYLYWQSNFTIINDSSASLALISTVSSIVCIAALLNFTSKIRFKGFEVIHVLRKLSRQKIRENQISN

>GbueGr38bFC

MHFRQRNILLSNELKGLQFPLFLLSIIIYYPTNNTASHFRKWFSFLFNNIILGLFVLINYLDFSGFVSATPRYEQIISNALNFSFIAFAVLSSTNILKSKNIYDLLNNIQRINELLNDSIFKNTKPKYNYTTIIILVKLVSCIFYIIAFTTLIILVKSSLDLKTYIYIIFTIYFSILAYAHLDLIFTFEDIVDMHLHMVHEKLSIFLTPPLEINRHDLNKVNTDVFIYCFDRLCDVNNQLNACFGLPYLMIITMYFFFILFECYYLYWQSNFTNINGSSASLILMPTVSSIVCIAALLNFTSKIRFKGFEVIHVLRKLSRQKIRENQISN

>GbueGr39a

MYAQNPVNDYIRPLFTFLKFSLAIPTDKYFQYSRKSLYKMIFFNSLWGILILKLAPYIVYEYEVEFDNVVTDIQYICLIIIVVVLPLVKTRSKINQINKLLNYLHFSIRSLKKPLNLDTHKKYLSYKIALVIFLFVITAPLFVHSVYTFPLILLIYYFPSFRYYCILEQFILFIDILNEFYQTVNSTAKIKQFKSVKFTSTDIKLLVIHEYEISIMANYLNDIFGIQNMLVIVYAGYICIHILFILPTIWICYFGNPTYRNLSFLIGIIIYPLTFVMAIFEIIFSTSHMFTKANEFKDLLRKIVRMDNDLLEDPSVSLLLILDPKIEFTAHGFFEIDRPLLTSIVGIITTYLFILIQFKEDSSRAVVG

>GbueGr39b

MIVQNPVNDYIRPLFTFLKLSAAIPIDDTFQYSLKSLCKMIVLNSVWTVLILILAPNVEHKYEIKFENIVHSTQYVGIIFIVAVLPLYRVHSKVRKINRLLKYINMSLSRLNKPLNLNNCKKYFKYRMTLVIIIFVITSPSFPHDIYELPNLLIIYYIPSFRYHCILEQFIIFTDILYEFYQTVNSSNKLKQFKSAKFTFIDIKLLVIHEYEISIMTDYLNGIFGMQNMLIIMYATYICTEIFFFIPSFWSNYVGTPSYKNLSLLIGFFIWPLTFIIAICEIIFSSSRIVTKANEFKDLLRKIVRMDNDLLEDPSVSLLLILDPKIEFTAHGFFEIDRPLLTSIVGIITTYLFILIQFKEDSSRAVVG

>GbueGr39cF

MLEENRLRDFISPLYTILKITSAIPVDSNWDYSRHSLIKMVVVNIIWIFLIYLLIPKIIMLDAKYVIEYVVYISQYIGIIFMIIVLPIVKVRKKERKNSEFLLHLFLSYKLYINNNVFIQFRAIYFYSRICMFIFLLAFVIIHQLLARGWIVCFYLAIVYYIPTLRYYLMLEQFVIFTDILCEFYRSINEKIKHLNIPDITHFDVKSLLMYHYQIAKSVGYLNDIFGVQIWLIMVYAIYSCTETWFFVPSYADSFMKNPSYVNFFYTIDFLSWPLLFVFTIFVVILSSSRVYQKANEFKDLLRKIVRMDNDLLEDPSVSLLLILDPKIEFTAHGFFEIDRPLLTSIVGIITTYLFILIQFKEDSSRAVVG

>GbueGr39dF

MLEENRLHDFISPLYTILKITSAIPVDSNWNYSRRSLIKMVVVNIIWIILIYFLIPEVISRDGNYVVDFVINISQIVGIILMIIVLPFVNVGKKYRKNTEFIRYLFLSYKLFIKKSIFIKFRAIYFYSRFIVVVVLLVLVILCQFLANGWIICLGGVIVYYIPTLRYYLMLEQFVIFTDILCEFYRSINEKMKHLNIPDITILDVKSLLMYHYQIAKSVGYLNDIFGIQIWLIIVYAIYSCTETWFFVPIYLFRFIRNPTYVNLFYLIDFITWLLLFFFTLFVVILSSSRVYQKANEFKDLLRKIVRMDNDLLEDPSVSLLLILDPKIEFTAHGFFEIDRPLLTSIVGIITTYLFILIQFKEDSSRAVVG

>GbueGr39e

MNVRNPVNDYIRPLFTFLKFSAAIPIDDTFQYSQKSLCKMIVLNSVWSVLIILLVPYIDPEYHIKFEDIVHSSQYVGVIIIVAVLPFYRAQSKVNKINRLLKYINLSLRRLNKPLNLNNCKKNLKFRMILVIIIFVITSPFFSNNIYELPCLLIIYYFPSFRYHCILEQFVIFNDILNEFYQTFNSSNKLKQFKSAKFTFIDIKLLVIHEYEISIMTDYLNGIFGIQNMFIMMYATYICTEIFFFIPSFWSKYVGTPTYKNLSLLIGFFIWPLTFIIAICEIIFSSSRIVTKANEFKDLLRKIVRMDNDLLEDPSVSLLLILDPKIEFTAHGFFEIDRPLLTSIVGIITTYLFILIQFKEDSSRAVVG

>GbueGr40

MDIEKTARQTGLRFVFNTCLFFGLHPFDKFFEIKFQFLFPTIIWLFFYAISILLNLIMNIQKDSLPDHFLEYLLFPVIVYGCFICLFLPLYYKSKYLDIFINLKTIDEIMRYHKNINQYEKYITVKLFVLVFVYIICSLYIFTYSCSWILCVNTVTYYSGYFSLIVIQIQYLVLLRNISDRMNYINIELKRNFQSNSIFSLLIYVYNLLIDSCYLVNSLHSIQILIIIIGLFLESLYIVYTVTQTKIEQNVTELNYFVLITYLALRGYQFWLILNASERLQHQAKEFNNNVYQMFMDNESNLAKNSKIKLHLLMKREVQFFAYGFFPLNYTLIRSVLAALSTYFVVLIQLHNK

>GbueGr41a

MNMLRSKSKVRDANEIKHVIELQHFNKLYIISKIVGNFPFQSVDSVSLFLVVYVMSSHCIVFAMSLSLLSFSHSVSSFVVVLKIVLVNVCLLGNLLSVLNSRVKWKNLVESIESFDSLAQRMGLQTKFDVNKHWIEYLYYLIFLLPIITMERVMRRELKFKLPDESSFFFAVILIKIICYCKLVVNECKYVKARFDALNFSLFQASNLHKQQHDLFFKERIQKKWENLTAQQERRTETNPVILRLEHLASLHDTLIDISRSAVKLRLTSTFFLTSSSFLLIIVSQYIALRFFMRGLILCSIHSGLWAVFMFYVIWDLVASNVNLSDEAAKFNKELQRLIIEDTTGLLSKNKVLRLHLAADRRVSFTACGYYTLDFPLIHSMVAAATTYLVILIQFTGTPSYLEDVTPSPSTNTTV

>GbueGr41b

MDAHANSLNDKKYNFISHDNIYNWINRFLFLPRLLGMFPYNSNFSVNYFLVFYVILVNVISLSLFTISTELLTKRRIQDIVLSIEYVSMFIVSLLIQLTVYKVHSSFKSNLSIINEYDRSLKFNGIISKYSKDKSYFIHILNSFLILICMFIEYSLASSSLWRVFDTNLYLCYLKFIGVRSIIVGYFKAIKIRLISTTDNLKILVKLVNFTTIERLENVCRLNSGLLRTIDYIRDDGLGIIALLVISLSMCFFVVDLYTDIFDVLWLLSRSVETYYIKPFQVFLIVAYMMILLSFQITNIWDLVYAVDTTNQMAAKFNKELQRLIIEDTTGLLSKNKVLRLHLAADRRVSFTACGYYTLDFPLIHSMVAAATTYLVILIQFTGTPSYLEDVTPSPSTNTTV

>GbueGr42I

MMVSKKAEDVVIGVFILSRLVGCAPIKKNFDKVYAAIVYSVMFASVFCGLDIMYTFRYISVISLQENNQAKEIKLTFLLSNLKTCLFAVLFVLSSFKVSRVKKVLVVCTEIDKILIDFNSFQPYEVRYFRRDVFYKFFFIMLTLCARLYIIKFKEGFIQAFIHIPLILTQHASICQYEILTSILHSRLKLINQALKNCFSNNKHLTLNEKEILALHLTNAHFKAVLTGGALNDIYSFQLLIYFLNIFFVLILTTFDLVECLISAWYGKHTGKQWTESLPLFIQFVIMLRTLIRSSEKLTDKELFVTHIMSKRTIIMTAWDLFNIDRDLALAIAGAITTYYVIVKDVLEI

>GbueGr43C

MLKQQYIGSCKLAMNYKYPQEIRIILFLSRFLGVFPYTEELVRSRFGSLTTTLYIIILVAVQFTLKIRSIEVINDKLNELLYFMVIVWMLTLVITVSIPLFSKENIKNLLDSYKKIDNLLHSTEENSLKKTNTILLCTVFRIAFFCLGLTLNISLRYRDNTSLPLIKKCQSLVLITYWFSVPFLFLQLQFFIFIKVLTRRAGLLANTVQKCSTNKTDLGRFIKTASEIFKASRLINSIYNLLLLALGVNCFLTNLVGVYLVINFHSTYSAYGEKENDKWINSSMTIFYIVQRNFEMFDILINIYELGQK

>GbueGr44PC

TTNPREIRIILLLSRFLGVFPYTEELVRSRFGSLTTTLYIIIVIAVQFTLKIRAKYDKLHELLYFMVIIWMLTLVITVSIPLFSKENVKNLLDSYKKIDNLLHSKEENSLKKNNTILLCTVFRIAFFCLGLTLKISLSYRDNTSLPLIKKFQRLVLITYWFSVPFLFLQLQFYIFIKVLTRRAGLLGNTVQKCSTNKTDIGRFIKTASEIFKASRLINSIYNLLLLAIGVNCFLTNLVGIYLVINFHSTYSAYGEKENDKWINSSMIIFYIVQRNFEMFDVLINIYELGQK

>GbueGr45aI

MTYEHKNTNVHINIKPLIKWMKRFGCCPLDENLKIDRLNLKFSYLLYSLVMIFIVSTHLNRFSIAKYVTMHDYKISFVSFLYFTSGILYIILVSVLIFNLTNLSGGICQILKVLSKTDEKLNGIKRVTYEDKLIINKKYIFTFISLIFTTSIKYIFFDNNPVLIWIIIKTVIALIPLIWFYLTNELIRTLGRNIAKHIKLCKDCLLLCTSKSSDILQQFASLMKSCYILFEMNSWYIFMLCFTTFFLLVVSIYSTFYISVIKEKLFTYLEIFYTMWIIFLAHSVWTVIETSEEIVKQRALILHLMASNRYKTPTVCGFFKLNRTLAHSIAAATATYLVVLIQFDPTTIDKSTSTPNNCTPT

>GbueGr45bI

MKISCCCLLDENFKIDQPHLKFSYLLYSLVITIIVLTHFSWSWIVKCETTYVYNINIMVFLGLISGITYIGLISSMIFNLTNASGGIRQILKILNETDEKLNGIKHFKYEDKLLLKNKGYIFTFVLLLFSISIKFIFFDNNHERPMSIIKTCYILFEVSRCFICKLFFTTFFLLVVSMYSTFYVNVIKEKIFGATEIFFTMWFIFLLQSVWIVVETSEEIVRQRALILHLMASNRYKTPTVCGFFKLNRTLAHSIAAATATYLVVLIQFDPTTIDKSTSTPNNCTPT

>GbueGr46a

MFTRGRIGFVKTRRNLLNVLKPLTTVASIFNVYVLDEALNFSYLKASFIIVFEVFKCLSEIAYTYRHLRRIHDFSGEESTRHSAIMSASQMYNFSVVLAGVTCVVNHFFTYKTLRKTFQTLNNVDLLLDRIDLSFTYYNDNNYFVYILFCSIYVCFCFILELATNLYKVTREPFWTATHILVRYHSIAISGTQMFQFSLIVYLINRHLDHIRTNLRRARAASRSNVEYVVSCHDVLCGLADDSARLHGPDLLIAIASCFQSIVVLLYRTIIYPDSLNHFYLISPKVVGFFKSALFVTELWLAIETSASASNKAKQFTQTVYEIMISENNHLSKEEEELFKMHLSRKREISLMVCGLFPLDRTLAHSIFATATTYLVILIQFGDKDDGENKDQMSTSEYSNATTMISTTFD

>GbueGr46b

MMKGEFKIEEKESVVSLLCPLLTVSSVVGCYPVTSKLELSWPLIVYSFGLTILLPFLVLNNLCSVHEYWKVYWMSNESFGLAMTVVTILSFYVCVITVPLNVDNLRRCLFRLENTEYTFVALGAPIKWTDDFSKSTVYSLGGIAVLFTCDSLMRYITYNNFLADNILYLLLGNYLCTVCLTCSTLLFRNLFIFIKKYLEHCTLVFREGRSKGTAIGTKNSLRLRELLAIFESLCQAKQFTQTVYEIMISENNHLSKEEEELFKMHLSRKREISLMVCGLFPLDRTLAHSIFATATTYLVILIQFGDKDDGENKDQMSTSEYSNATTMISTTFD

>GbueGr46c

MFWFVKSDNELISSLKAIFFVSRLFGTFPLNKKLQLSIPNVAYSICLAFFCLASMYFDKDESLRRRGWRDIVFIAYLSISTAICDSVEFITSILFCWNRSKIQSVFKEFMSILETLKTVGEPVEFHGVLHVRLLLLWMGYFLLAFACDINTRVETEENYFVDLIDYSIVGKFLMLISPCTVLTLYIALFTVTRLIFKHLRTRTRRMFQGKPMKDNYLKVKTFLTAFNRLCDLCVTTCSVFGLILFVTILNTFYSATIMTYYMITKLNNFEKHTPLHWFVLWITHFSAKVWLIIESVTLATKEAKQFTQTVYEIMISENNHLSKEEEELFKMHLSRKREISLMVCGLFPLDRTLAHSIFATATTYLVILIQFGDKDDGENKDQMSTSEYSNATTMISTTFD

>GbueGr47aP

MITFSYFVLLTKISNVIKLIFNISKITGINTLNEKWKFSFPLFFYGFVLRLLWLYKAQRHIFLNRGTZLDITAGSIISIAHMSTITLSLVLPLRNASNYRILLYEIKYIKSVLKKRFIFVKSNLINIKNVVKILIPYIMYTICIITQVLNGDDYECPDKNCVINTLIFHSYPMIMNLIVLTQILTILQFLSMLFTSLNKSLKKHLSLNNLETRSVRSLIRFHNILCNNCDRVCDIYEWQILFSLIEIYCLCIINFHWLVAVGLFQNLVTAITFLSVIAVFCLKVWFIIEEASNIKSLANEFTLILHKRILITPGITSKDCPTAEKHILMRRRVIFSVCGFFPLDRTLIHMMVGAATTHLVILKQMGDYSMFSHNTT

>GbueGr47b

MFSYWRDPLKKIYILLNVSKIFGSTFISDRRNESKMLVIYSIILRIFWYFTTFHQLIFTRDIKTFYRRVFKKTISIAYLTSMVISFYQPLKHFDNLKQIILRIHWLKDCKLNVSENAYDFNRSDLLKILFLFGSSITLVIAEFVGETRQDILFHILFHEYPTFNNIVLLTLVRVILQSCKGLFTAIHKSLKEYLSKKDSILETELIRNLIRSHNILTNTCEQINNVFEWQILASLLTSYLMIVNNLYTILDANERNSKNLLRMTSFVLTAALYVVSTFWIVEESVAVSKEANEFTLILHKRILITPGITSKDCPTAEKHILMRRRVIFSVCGFFPLDRTLIHMMVGAATTHLVILKQMGDYSMFSHNTT

>GbueGr47c

MIQILKIILKKKLNIAKEALQVNCFINMSKRKSNLNMSGQLQIVFHFSRFVGSNCFDNNFNISIFLTFYSVSLRVFWFLFCEPRTAKITLTQFYMLFKILVYYCYLLAGLTTFVEPLLKLNDIKHFISNLQKVKGILLKIEKRSQRIRISNIIKFILIVPNMICSSCYLQFYLRRNFINAFYHNFPMWANIVSLIQLRTILHSVSKLFTRVKELLSEYMKTDGAILESKSIRDLVRLHNSLTKISSEINSTYEWQTLSSLFTAYVIIINNLFNIMQINTSSFTMVIFLLQTVIAFIVAIFWVVDEAETASTEANEFTLILHKRILITPGITSKDCPTAEKHILMRRRVIFSVCGFFPLDRTLIHMMVGAATTHLVILKQMGDYSMFSHNTT

>GbueGr47d

MTFKNILNKISDPFGHVNVVIMLSRIFGSFPINTDWNTSIPLIFYSALLRFFWFISINNYYFRIIFPHSQSFYRAVFKTVVNNTYIITVIVSLIEPLRNCKKIKSCVKNIYILKSFNFKHGDTNYKTDLWMFLKYIIVVGNVACSTYLQYRAAMHFKTLYVNYYHSFPMWTNIFFMIHLRSILYVIFILFSKIKFSLEGYLAEADSKLESKHVRDLVQLHNILGNVLENVNGTYEWQTLVSLLTAYITIVNNFFSVIHDQPQNNTFKNVLLFSHVCLFIMAIFWVVDDSMAVTKEANEFTLILHKRILITPGITSKDCPTAEKHILMRRRVIFSVCGFFPLDRTLIHMMVGAATTHLVILKQMGDYSMFSHNTT

>GbueGr47e

MFESRVFSELADITSQIKIVFQVSQVTGTFQINDKWQVSGFLVLYSVVLRVLWFVSSLYAKLKNTNTFLTRLFKNILYITFMITSATSLSESIRLVSVYKTFKRNLELARKFPFRDGKPVKNVNIRNVVKYAVVSITLLINATGTVIFEDHDFVGKIIEMMYTNYPLLTIVLPLIQLNTILNLIKMLFIRTKESLNIYLSERSSTLEANTVKDLIKGHNFLCDICDQVNRAYEWQILSSLLTAYVNIVDNIYNIIQFRFSVLEYVSSALEILSIILIIFWVIEESVAIKKEANEFTLILHKRILITPGITSKDCPTAEKHILMRRRVIFSVCGFFPLDRTLIHMMVGAATTHLVILKQMGDYSMFSHNTT

>GbueGr48aC

MNTKIPYHLNENVRILVQGKMSKQQRLSFENLNSPTLKLFYRIFRISGHFYLNDHLNYSKLLHLYGTASTIFVLSLLITDRFYAKIPPGFNKFDFAAYSLQFYLFIFTVFFINVNNYQINKNIRDILNEIQKIKDLTNTNISIHIYPNIMRMIMVLLIHTFKFISDTYVHYEFTSVRFFIPFRSIPLLFLHLVTEHFCILLRFVEKFLQTINDRLIDSICNKTFDSRNLESSINIYERMVDVCNKISKIFGLQNLYAQFVIFYTLTRKSSVIILLVLESWDRPNYYSHFIWAICCAIIFTIIYLATISTSNMVSEIKETLYQLGTGDVSPSTRFKISMVLAMNREIKFTALGFFALDKESLHS

>GbueGr48bC

MFRIREFSQTNNINDHFAIFLRIYRFMGCLTFGKDLKCFKFLVIYSILFNIFLVVNMFYFLYLLSLEMTFTQFGSTFKIFTIFSSIIFPSIIIINFSLYSYSKDIKIYISEMNDLNNKYIKYNEIMIFNKKIYLIRIALMLSLVSIVVVAYSAFSRNGFEVDIYHVLYNIIFSCTIGFLQIFHLQFCHVNIEVIGLYMKGYLNMIRRDILKLIQNYDTLNDYFIIYESVIDISDRFNNIAGIINLYNNVSFVASAINICIILIYMSDFSRVLPKSYSSLFTFIAQLLYLGLIQFFTFIPIFNVSEIKETLYQLGTGDVSPSTRFKISMVLAMNREIKFTALGFFALDKESLHS

>GbueGr48cC

MKKNVINSFKTNNVEEYFKLLFTILKLTGSFTLNKNLKFSLFSCFYSFLLNLCLIFIAHITIAESKVLFMIRINMLKTSGIIYLISIIADHIIILINIISSYSQINLITKLYKLKVDEKSEFKKYYSKTRIIFMVRFTLTTIIFIVFMNILAPISLIFQLYLHSYILKTGRCILLYFIYIHTEKLYMINKFIDLTLDFIHKNINNYYFVNDKSIVDTILRLEFLLDSVQNIIDDIRFAITFICIQNFIFNLGLITWASFLIENPSKFIYSEIIYLESIMFFISLFIIYNPPLSIISKVSEIKETLYQLGTGDVSPSTRFKISMVLAMNREIKFTALGFFALDKESLHS

>GbueGr48dC

MRIFKRIVKLFEINNTQEYFNRLLNILRCTGSCCVFNNLKISPLLCLYSFFWNSCFMYVAYITVFMSTPILLIRLNAFKASESEYLVSAVIDPAVVLINIISLNDQLKLIIKLYRIAEMKIVTDGDFHRYYSISIFFFTLRFIFISVSFVRIVYVWAPFKLFFILSFYFFLPQLGKWIAQYFIYIHTEKYFLFLKFIELTLRCISRTISDYCDLKHLNDGKIVEVLLNFESMIDSAQIVNREFRILIGYIFSRFFIFNLGLAKWYYMFKGNSMSKLFTPLINFDILIFLMTLYLFCSPPLSITTKVSEIKETLYQLGTGDVSPSTRFKISMVLAMNREIKFTALGFFALDKESLHS

>GbueGr48eC

MWNYHKVLLYFKIDNTEEYFNLLFNLLRICGSHPFGKNLKFSYDLCFYSLAFNLLFIIYELFFITSFIRSLNLYNGGKMFFMLLPIIDPLLILLNLKSSQDKLNSIMDLIKSIELKIERRHFNKLYTEAQILFTVRLVLIDILYVTFMYYTANFKLKFLFIFHNYIFKVGRSVLECLVYIYTEKCFIFVKFIELSLVDIYKTLNSENFNKYNNHRIVDVLEHLELSIELIRTLNREFRFLITFTCFQTFIYFFGNIQWAIFLSYPDRVFYFRNSWFYIVLNLLKLYLFWTPFFKIISKVSEIKETLYQLGTGDVSPSTRFKISMVLAMNREIKFTALGFFALDKESLHS

>GbueGr48fC

MNLIKNKISSNKSNNSAYKYFSFLLFLLRLTGSFVHDNNGKFSSELLTYSIVFNLSILWFINDIFLPFIWLDFDWTLVIVADVIKWELQPVLIFFKCHLVYIASKQVLNVLEKSSDLLNDCESLGSVISNCNVAFVIRLIIVLFLCLLITILECHLCGNHYFVYSNILFIMYILIEDFGNYFKFFEIIGKCLWTKICEILSYNVVNSEKLTDQFLLAFERLIILSDEVNATFNYLIFCTYFFCVISLISLSFNYTNSVIVVEVYFSEYKYQVLFDLMLVIYGFYYYLSLTRLSNKVSEIKETLYQLGTGDVSPSTRFKISMVLAMNREIKFTALGFFALDKESLHS

>GbueGr48gPC

MNLIKIEISSNKSNNSAYNYFSLLIFILRLTGSFVYDNNGKNFSXYSIVFNLLISWYSVGLYAPVIFVYIDWHLVALADIIRWELQPAFIFLKCHLVYIASKKALNVFQKIGDLINDCQLLGCVILTNNITIRIRLIIALFSCLLVTFLECHLSRIKHYVHSTILFIIYILTEYFGNYFKFLETVAKFVSNKIYDFLSFNVVNSEELIDQFLLAFERLIILSDEINATFNYLIFCTYFYCVVSLISSSFIFTSSSVEIQFYIYELKFNILFNWLLVIYGFYFYLSSTRLSSKVSEIKETLYQLGTGDVSPSTRFKISMVLAMNREIKFTALGFFALDKESLHS

>GbueGr48hPC

MNLIKNKISSNKGDKSAYNYFSFLLFLLRLTGSFVHDNNGKFSSELFTYSIVFNLSILWFSAGLYVPILFFLLXHYVVVAYVIRLESQAIFIFFKFHLVYIASKKVLNVLEEISGLINDCKLLGCVILASNFAIRIRLIIALFICLLITFLECYVCGIEDFVHSTIMFIIYILIENLGNYCKFLEIIGKIFSNKINDFFSFNVVNSEILIDQFLIAFERLIILSDEVNSTFNYLIFCACFFCTISLISLSYIFTSFMAIKYYTYKLKFQLLFDFLMVIYGLYFYLSLTHLNSKVSEIKETLYQLGTGDVSPSTRFKISMVLAMNREIKFTALGFFALDKESLHS

>GbueGr48iFC

MLKMFHKLREFNAVEYLHLICNFLRIIGLFPYDKNYDFSPALYVYSVVFDLFALICILYLYLPAFRVFITGNILFLIENLKEIITPLIIVLKIHSTCSVSKKIRNVIPEIKNVIHLIEELKLSISLELFSPIVRFTCISIIITAGILGNGPYYTLQEGLSTAITWFLSYFLLESYAYFFTFLDSLLLSMLHNFIIFDNSRFIKDKYIRIIQTHKCKIRNETVIKYLIIYDNLISLSYKMYPIFFSFIIYNNFYLFYNSLYWFKLIWKYFDHIYSDYFFSYFKYDMSFSFALISFILYYNYIAVARLNEKVSEIKETLYQLGTGDVSPSTRFKISMVLAMNREIKFTALGFFALDKESLHS

>GbueGr48jC

MFNKLRRLNSVEYLHLICNFLRFIGSFPYDKNYVFSPGLYVYSVVFNLFALICILYLYLPVFQVFIKGTGNILLILENFREIITPLVIVLKIHSTCSVSKKIRNVIPEIKNVIHHIEELRSSTSLELFSPIVRFTCIFIIITAGILGDGPYYTLQEGFPIVTILFLSYFVLESYAYFFTFLDSSLLSMLHNFNIFNNSRFIKDKYIRIIYTYKCKIRNERVINYLIMYDNLISLSYKIHSIFSSFIIYNEFYFFYNTLYWFVIIWKFIKDSYFNYVIWYFKYDFLFSLALISFILYYNYIAVARLNEKVSEIKETLYQLGTGDVSPSTRFKISMVLAMNREIKFTALGFFALDKESLHS

>GbueGr48kC

MNFFCKMFKKEFSCCAVVYLTVVIYFLRITGSFPYNNNAIFSPSLLVYTLSVNLFMLVSIVYGYYDVFNESMAWAVLIVLETLKSYIEPLVIIWKIYFACCYSKEIRNIIPDLKTLIRRVDGFGWSISLDIFIPGVRLIMYFLIVMAVIFLSAPIYDVFEGLAFGSTIALCHVLLETYANFFTLLNSVLITLSHNLKSLVLRSKQNYVKFTRFNQTIIKFLLVYDDLISLSYKIISIFGSLVIFNHFFTFCNILYWINAIFQMINQSYFKYYVNYFKYDFIQSLAHFSCVLYFNFICSSRINEKVSEIKETLYQLGTGDVSPSTRFKISMVLAMNREIKFTALGFFALDKESLHS

>GbueGr49

MDTFPFRSKVRDLSVKRINKDFNYLFIIAKLLGDIPFNSEFNYKWYLILYILATYTGLISAAVLCLTSPTEFKGFNKLFVIINYVQFNLIGLCGIINIFCSLRLKKSLIKITQQLEYFDANLKRIGDVVHYDYSSCMYGAAFDLIFIVIDLFLDMIYSDGIAEMPWLMSSVFYYGMFAKMFMYKRLITNMMYVFKVRFKKLKGLLNSESFTEKLGYINVEILCYQHHRLVEAANLLNIAFGPQIMAFISIFFMITVVLLYTLTSTVMALQVQNPRANVTPFTIVVMSWSIMAFYNVWHIIHAASVTSNEAEDFNKELHKAMVKDSTGNLSTNKLLLLHIGARRKVVFTAWSFYPLEYLLIHSMFAAAATYLVILVQFSPPAPSTDISV

>GbueGr50F

MTRWVNPVKENDIRSFFKLSAFVGLYPFNDNFKISIKLSLYGIFAIITILVAYGFSIFGFSGKRKKWFGQDFFYWLLFSIMLNFSVVCIYKTFYYGNFVRQLIQNLEKADALLSYKQKNIKIWYKSLIFKFTWIGMTVVTFSLSNNMLRPFQEYIPEIICVWPTIIISVFVESQFLIIITNIYQRIDLCKIFLNKTLYSFRKLDNLILINNNLLEDAYKTNKMFSPQLLLLVSWMFAEIIHIVYKTISGTFKFEGSYKFYLSQISYLLWIFFRCSQLYSIIRQCNQLTQKAKEFNNTLYQLIMDDPSGVITKNGKIRAHLLAKREVKFTVFGFFPLDYTLIHSLSAAIATYLVILIQLGDTDIIDACSSPTPITYLQQNLAGNLSSSD

>GbueGr51

MPRRVDLKQENDIKSFFKLSTIVGLYPFDDKFKISMKHSLRGMFVITAVLAGYGFEIWKFGGQKIAWFGLRFFYWLIYFIMFNLCVVCIYKSFSYGNFVSRLLQNLNEADVYFSYDRRSIRVWYKSLVCKFTLLGVIFVTFRVSLTSNPFVDYVPGIIFQLLTLVISVVVETQFLIIIASIYRRIDLCQRFINKTLDLFRKLDNLMRVYNNLLEDADKTSKMFSPQLLLLVCWIFAEIIHVVYTTISSTSNYEDMYRFYLSQTIYTTWIFLRCFQLYLIIRRCNLLAQKVKEFHNTLYQLILDHPSDLSKNGRIRAHLLAKREVKFTACGFFPLDYSLIHSMSGAVATNIVILMQLGEADESVLKIDECISPSPITFLAGNLSSCN

>GbueGr52I

MLIISRFLACCPISRTYVRSILWSIYSILIMLSISMLLITYVVIDVIKDGIVGSKLDFYYAICICILSVVILNVYIIKSLRFNYLKKIIKCCSLIDSLLRAFGYSAEYRMKWFWVSTFIRVSVVILSFVIDYIYERVTILYSSFTISLLIINTVECQFEILLHIVTSRFAALNQSLRNIKKSDYEDTNKLQMIVALTKANHRLFYSSQDLIEFYSLQIFLFVLFTFFILVFGWYDVIESMMLFVRNQKSELDTLWISDAPYVLLAMIMLFSIVLRCESLTRTHEYFGGKLFRMMIDPSLKILLKSTVGVITTYFVIMKQFSLFQN

>GbueGr53F

MSQKNDQRKLIFQKYSFVFRTLSTFGQSTLNNELKSSNYLLVLGLCQVLFYLYGAAISLYKLETLVGYASSIHLTFDKIRLVLMILTVFIGLFETWLNRSKLDLVYSEFENLKDLLVEKDLVRTKWFIVTILILLLCCVYTFFLDAAVILNRLTFEMFIVAFTYDYLVFSMNLIAIKIALNLYIISSYLKSIRDCVEKLNDSDKNDVDNLYINTDIKNRDKEDKMIKLIIYQKRLMNCLELINESFKFQLSLLVLTSFLQVTGLVFARIKKVVDPSDEEKVEHHHASPFHHVKQLIKTTIDICVTGFNVIVIVRAADNIDDKSFDIFKLLKQISNAFLFMFPDDRSCCNVNPRVELTACNLFKLDYALVMNMIAAAATYVVVLLQFSMSNKCSQ

>GbueGr54

MLQKILQNIQMKMNLQFSNRLSVKRSKAQIIIDFSFFLSKLTGLIPFSYSYTDSAKRLIYCVTLKLIILAYTTFVLIHFILRDFNHLWTRDVYQASMSIYLIIIIQSYNEIKNSKKFTNIYNTLITIDKKLRRTVKMKYKYKTYCFAILLNILLPIFAAFWLSLKVYPYPRSILIIIHVSLLFLIAFCLQITAIFWLIGDQIDSLTDCLKSNEISHLNLIVTTKWRLGCEYRQANHYFSIVSAVITFIILINTITSIGVAVFRLDWSNSSTILLLFYPFVTGMPFYTMLKAVDDASNQSINFSNKLTGLLVNKRIDLPANAKELIGLYIRTDKVLKFSAEDLFFIDRPNFSSMITVVVTYLVLILQFGSSLFETPVST

>GbueGr55a

MGSPVDSPVSQFYRDFRKMCLILIPVGSVYIGNLKSGSSSSLGFSKLLCAYAVLYMLLVNFGVEYYQGSLGIDYVPFHILVYQFVATFITNTLGQTEIIRLFRSLEGYDVSFPTFGRDLVSQTPTLKKFLPLIVSSCIYGIKLTVVTLYLSKFRTFTYLYTFSSTPMTGILCMYLFFAREIEERFVKLNRRFDEEVASGKCISSRLETVRKQHLVLTRSVKTLREAFGPQLLAMFSCLAMNVLCAIYIISKDAGSIAYLSPIGLFFMGFTGVISFSTHSMINQSRQLLRNTFHVPLTNLDPASQFQMSYLCQQLMNTEEEVTCLDLFTIDMSFLGSFSTLLATYTIAILQIPWTSQSQ

>GbueGr55b

MRYRCSDQVGLYRDTRRLCLIITPVAGVFLRGLGRTSCACSLGFSPSLCLYAFFYFVSVVLGVLYYQKTTHDYIPGFIVISNAIFTFVTKFISQTKIVELFVALDGYDKSFMYYNDGPSIEIPLCKRFSPLTVSSVFYLVKLSLVTYNVSRVGFFAYFYTLYSLPTAGTCILYLFFTREIKERFVRLNGHLEEDLVEDRISSSGLEAARMLHLRLCRCIGALREAFGAQLLVMFCSFVLICLSAIYAVAQDLAMMSHLAPLALFYMVVIGYISSTTHSMIRQSRQLLRNTFHVPLTNLDPASQFQMSYLCQQLMNTEEEVTCLDLFTIDMSFLGSFSTLLATYTIAILQIPWTSQSQ

>GbueGr56a

MVQSINLKRNEATPRIVLVFKKRSIYEKFRPIAQLSQLITMLALSNVWVKNKRYTFRWLSFPTIFVLVSNIGETISNTFKVYTENIGTAFDKVSLPILDCISLIILLLFVHKSPLLLKLLEILNNFDERLFKTNLNISTKNDNSLKYYVIIIILYLTLCFGGIVESIIGLTNSKFSFEFTKIDCQFMLSVIYVFFCNEIKLRVKEIKRVFDFNSPTENIENIRSLHLCLYEGITILESCFSSLCSGMITFISLSTLVQTTLIIKEEGSASFTSFIYVFSLIAIYASVSLVTQRVINETQRMFAEIYQLPVNNLKPEVRFQTCYTMVYEQLFIAHTIIINDKNLEIVQVILKNSFSTVMITYIIALMQFAEPIKKFFRYLESNQMVYENTTRENFY

>GbueGr56b

MSQLFLIFQDRNQRLRKRETAYESFKPIARFAQIFGIFALNNLWDKSNKPSFKYLSLPVFYVLFVNVMIVMYDFYNIFFSIVTIPIVTLFVQILVIGLDLITLIVIIFSVCKTNNFVILVKKLNDFDHQYLDFGIYELFNFRKSNKTYLFMIYFYLFSWLMTTYYGIVHLALSTSLFLPEFSFYFHKMSIQYFFSLIYNYFCNEIRFRFKLLNKAIPSFVGKKYGRNIDDVRFLHLSLYECVRSLESCFSGVIIGVLMCMTTFIIVQSVQVINDGALLSDTTLVYLIWLIVIFISLAISTQLLFNETQRMFAEIYQLPVNNLKPEVRFQTCYTMVYEQLFIAHTIIINDKNLEIVQVILKNSFSTVMITYIIALMQFAEPIKKFFRYLESNQMVYENTTRENFY

>GbueGr56c

MPVSIISEFRCKSKYGRRIKKRKRIYETVYPQLKFGQLVGVVVLNNVKYHEAPTFSVLSLPVFYGIGFSLLSLGLNICFTTTLLKHNTVRLLSSFGLVLVDIGSICLVLMGVITTDKLVKLLHKLDDFDKICEKWILIKQSKPQDRFNLIIMIIYLTNWCIGLPIYFYITDESLIFKLAYFIFSFTKMNLQFISSLLFCLFCGEVHTRLNCLRKSSQFIINTNTAPNVALLRYLHSHLYKSLLLVGYIYQHLFIGIVFFISAFMLGKTLQLVLLEETSLHLFDSFSAIWLTGVVTSVTTSIQRLTSETQRMFAEIYQLPVNNLKPEVRFQTCYTMVYEQLFIAHTIIINDKNLEIVQVILKNSFSTVMITYIIALMQFAEPIKKFFRYLESNQMVYENTTRENFY

>GbueGr56d

MPVSIPHSGNKYVEKSNDRKNIYQTLYTTLKVGQLASVLILSEVKYNEAPKFSVLSISVFYGLSFCILSLGLNLYCIIMFWERASLVQMFTSFVMIFVEGGTIALILYGVVVFPLLINILNNIDDFDRICENFSLIKMYKSPERQFQIVMILYLINWCFGFPIYFYSSKKELIWRLTYIIFTFHKMNLQFISSFLFCFLCSEIRTRFLWLRKASQDIIRSDESANVAFLRSLHNNVYNCMLKIQNAYQHIFIGVVFYLSNFVLGKTIRLLLVGNSVFNVYDVFTLLWLAGVLTALAISTQRLTYETQRMFAEIYQLPVNNLKPEVRFQTCYTMVYEQLFIAHTIIINDKNLEIVQVILKNSFSTVMITYIIALMQFAEPIKKFFRYLESNQMVYENTTRENFY

>GbueGr57a

MYLNLPNSFPNVFDKFIKIARITKYLGGFPIKNLSQLGVDDQDDVNYSYLVIFLTIVIHLSCICVEYVITNERLETSFVVLKFCIYISNWSVVVATNYHAKYYVKLARILQGIDSFTYSLNLKWNQFGYLQRLYKFRIAVLVISYAFCFGVGGFMRYPFLNKWESILVAGTQISKNIAVLAQALLYIFFCNEFTQRLINIKKCLDHILTNTDVQFLENVRCVHGQIITAGHVLEKCFAVNIFMLYLRLVSFVIICVFFLIIMDTMSDDITFRYTSMFWVFIDFCTVIQQCHSLTKTSESILDDMYSYPVDHLNPEIKFKLTFFHYQVKRFPVKVSCGFFDVNMSFFGSYTTVLVTYIIALVQFKPVIMRYLGDPSCLQGKQCDNTTTLLDFTTQTSTVI

>GbueGr57b

MEGIYGRFRPIAVMCRAIGTLPLKNLNRTNEELSFTWLCPDTFIVIIVHLTCCCLEFYATFDYLESGMLILKFFMYLLSWVALAATFYQTKSIISLIRSLEQVEKQMMDMELNLSRKCGISFSLFLPYLICSFGVFISGFIKYPNQALENNLLAGVASASRSIVIISHSFLYVFFVLEIRRKFQILKLNLSRTLTNWDSVFLEKMRMLHVDLCTTVECLQKCFGFTIFVICMRVGLYIGICVVFLMRYETGSDVTFSRYASIFWASLDFTTIVLHTQWLENTSESILDDMYSYPVDHLNPEIKFKLTFFHYQVKRFPVKVSCGFFDVNMSFFGSYTTVLVTYIIALVQFKPVIMRYLGDPSCLQGKQCDNTTTLLDFTTQTSTVI

>GbueGr57cF

MLFKFQSSLRKRFYPEALFCKILAASNLDDSKYNNQSPFVSILFILAVTYQISFVIVDLHFTSVQYQIRYVHVGLTKMILSLISLLWGFHQSNNIKKLIETLERIENSMLLNKFSTVKYSTNYYRLFGYFSILISSLFSLQSYLLFKKPNLIVTIISYFLVCIKMSDNYVYNMLYFFFSNEIQIKLTMMKYNVDRMIRDEKSIDEMRSIHSDFLLSMDLFRSCFGVHVMVVLFKISFFTHLYVILFTVNKFGTMDVARLTVVVWTILSFLMVFYQSYKISSISESILDDMYSYPVDHLNPEIKFKLTFFHYQVKRFPVKVSCGFFDVNMSFFGSYTTVLVTYIIALVQFKPVIMRYLGDPSCLQGKQCDNTTTLLDFTTQTSTVI

>GbueGr58

MKFKFKIKEVKNENIINVLKPIIQACGFLGVIPLTFDQHNKKFRHDLVSLITLYSVVWYLSLASWAAFDSYDMLMKFATVKHIMFFWYNALTFMLQINLLLSVLYSCDKIINLAESIRSFDKFYSSVKFLQYRLSNSRLLWIIVLGIYFAIWMIMLIQGFPESTPILQLIFFEYPLRFKFFTTVSYFAFCKELHLRYVHIRFQICNCTTNAWRIVTLRLLLSRLYDCTRELENAYGVIWLLYVMHATCVIGWQLIVIIDARNMEEILIVHTIAIMQYAYLVPLVIVTQRLTDQNELLLREIHRVSNNVTDRDSKFQLIYLLKLLRQQPIRITCCGIFNINIPFLVSCWNGVATVGIAMAQFSPQIRRVFDNRINSTENSLT

>GbueGr59

MKAIAYHFTNTFFKKDFTGSIIEVLIPILQISNIVGVIPITFNTSTGYLSYDLVSLKTLYSALWYCLIPSTAVIILHDVISQNSEYTSIIYLFHELSFLIFQTAVLLCVLYACNKIIYLTHSLNSYDYHYKNCKYLRNMNSNTRMYWILICIYISIWISLIVYKIVNDKLSIINSIVKTILFEYTLRSKFYIFISYLIFSREIINRYSYLCLQISDFSSETSKLVSLRLLFTRLHKCTQLLENIYGPIWFVCIMHTSYSLIGELVLHLALSPYPHINLVSENLAIAAQAWYLLPLTITSHQLINQRDLALKEIHKALNNHNDIDLIFQEMFLLKQLRIQPIRISCFGLFIINIPFLVSFVNGMATMIIAVYQFEHQFTKFFKKSEK

>GbueGr60

MLKMSEFYLSFRKVCFSFAIFGSAFVSNLHCKTGKELQFHFISWPFLWCLVVLALPHFIYHHFDIPGDKTSLTMQILFFTCNLLTVNRLITLVKLIEQYDKAFMKMFKEQPIKVKISKKLINIIIFVIYVIIHIILYLLDDDEGEVHKLYVCANLFMIIPPTSMFFFFLFFLNELEGRFKRLNSTWYHVVSSSKIDPKSVESIRVLFALLTNIISHLGNTFEWQLVLLLLFLITQIVTASITLSAFYYLDTVLLRSILIFMTLFILVAFQTDKMIQESRSLLKNTWTIKINNQTPNVYIQIELLMDLVVNNDQDVLCCGFFKISLSSLCSLMTVVMGYVIAAVQFSSSSSNSPQNED

**45 GbueIR proteins**

>GbueIr25aI

MRKLCRTSMSVMVVNEEDNILAAKSLQVALNFIKRNPKMGINVDKLASITVSGDDAKAILESLCQNYHDMLDESKPPHILIDMTITGVPSESVKSFTAALALPTVSTSFGQPGDLRVDHKYKSLLLNVPTRHMMIEVQDAKSAAHYLTRFRDLDLVNFFILGRLSTIRTVLDVANSNKLFEPDPSTREKLNLLKTSYSLTAEPEITAVFYYDVIIRSLLAVKGMMDKSEWPQNFTYTTCKDYSAESEPRRKSFNLRRNLKLVTEAYSYAPIAIETNGKSHQVFQMRIEKVTIHQGQAVSAETVGTWRAGLETQLSVKDSSALSNLSAVTIYRVVTVAQKPFVFESEENGIKKFKGYCIDLLEEIRKIMKFEYEIYVAPDNKFGNLDDNGQWNGMIKELIEKRAEIALGSLSVMAERENVVDFTVPYYDLVGITILMKKQRNPTSLFKFLTVLESDVWLCILAAYFFTSFLMWVFDRWSPYSYQNNREKYKDDEEKREFTLKECLWFCMTSLTPQGGGEAPKNLSGRLVAATWWLFGFIIIASYTANLAAFLTVSRLDSAIESLDDLAKQYKIQYAPVNGSSAMTYFERMANIESKFYEIWKDMTLNDSLSEVERSKLAVWDYPVSDKYTKMWQAMKEAKLPNNLDEAVARVLDSKSSSEGFAFLGDATDIKYLELTNCDLVAVGEEFSRKPYAVAVQQGSPLKDQFNNAILQLLNKRILEKLKEVWWNENQDKKQCEKVEDQTDGISIHNIGGVFIVIFVGIAMAMITLGFEYWWYRYKRPPLTQTLQERARTGGISGQPAPPPGMDKLSVAALNKQLHSVRPRNTIRSP

>GbueIr8a1

MMKSLQGYQTILVLYFAWSSFILIICQAQKNRQLILLKDIGDDRDAIDLWKKDWDVTVVTMDRNDKQESKTKMCEALAKGGNLVLDYTYHEGWTEAKDAARSAGLVYVRIKTIFSVLHQAADEFLVSIRKARDSALIFQYPYQMEESLYYLVHESPLRVILFDGLQGKDIQRLQDMRPTPSYYLLFANTRPMNSIFKTAAENKLAKLNDRWNLVFTDFSYQTFDRNLISMRTLAFMVNATFCCGLHTSANPCSCNPSDDLEELLTETAMHCINEAVSKTDSQPISAQCKDNYKSDPSSPAFYRQLQQCLKKTSSIRLEEEDEISADLQVDVQVWRATDPEVHIASWTSNGGVTTITDIPPVKRFFRIGTGFSIPFGYPLIESGKEVVDEEGNQVWVGYCIDLINKLSELMEFDYELITSYDFGEKNKNGTWTGLVGMLAYAKIDLVVAPLTMTSEREEVIDFIAPYFEQTGLSIVIRKPVREISLFKFMTVLKVEVWLSILAALVSTAIMIWFLDKYSPYSARNNKQLYPYPCRDFTLKESFWFAVTSFTPQGGGEAPKPLSARTLVAAYWLFVVLMLATFTANLAAFLTVERMQSPVQSLEQLAKQSRINYTVVKDSIAHMYFNNMKHAEDTLYRVWKEITLNASSDQTQYRVWDYPIKEQYSRILLAILKTGTVSSPEEGYAKVLESEQGEFALIHDALEIKYQVDRNCNITEIGEPFAEQPYAIAVQQGSQLNEEISRKILDLQRDRYLEELTAKYWNASLKGECNDIDENEGITLESLGGVFIATLVGLGLAMITLGVEVFYQKKKDKKTSDIKPVTKEGTSPTKTVPNKDFIQNKEFMLRDELFTAREFGLSFSKKEKKSAKILQPPSNVAFISTYPRGQLY

>GbueIr8a2N

ACILYSYMMSVTEDEIKSTLRIGTFFTVPYAYPSKKNSSIWEGFCLDTIGQIAKEMNCNYELVTDMEIGRKLVNGTWTGLIGLLATARLDMIVGPLVPTPQMEEVVDFVPPFYTSVGFSIVVLRPMKDSSLFKFLTALNLDVWVSLLSCILVTALLIWIIEYFSPSSSTNIKSDNESRHRKFSFKESLWFAATSLTQQGGGETPRPLAAKTLVTAYWFFAVLMLAAFMANLAALQTVERLQYPIQSIDELMDQTRIKYTVIKDSNAHKHMVAMQKAEARLYKLWKDVILTKKEGEDEFVEWTYPVKERYKRTLYHILSTGLQPDAETAYAKVLKGEIALVHDDFDIRFRAARDCRLKEIGETFSPRPFYVALQHASPIANNITKILVDLLRHKYFEDLTKKYTEPRVIDSECVRDYVPPGITTSSLRGVFVCTLIGVGIGIIVLIFEITWKKTASSNTGNDSKLHNQNLLRKGEHPEEFPRELNVTGRSLKDAYSNFDKKLAIPLQPWTN

>GbueIr93a

MPPKYRGEFSKVKEIITESLSATLKDAAISIVYLNSPDISNTRDVVAVFSVASCLDTWKIYSSAREGGIIFISLTESDCPRLPVSEGITIPLVELGGETSQLLLDLRTFDYIDWKSALFIYEESIDTGTVKNMIDSLTKSVSPEAKSCDLILYKVPNSDVEWERNKIVSRIMSQVPDRKKQRNNIFMGSSTMITPIWQNAKTRKLTSPLSRWLFIVTDLPGEGLNISSLAPLIDEGENIAFIHNATDTQHKCMSGVVCHVEETMRLLGEALATNLKEEQLLASQLSGEEWDAIRPTKDQRREALLTYFVNQLRTRGKCGNCTSWVMETSQSWGRSFTHLTKQLLLQIGYWTPTTGPVLNDHLFPHVAHGFRAVQLPIATFHSPPWQTITVNESGHETYRGVVFEIISELAVRLNFTYKIINFSKSVNSTNSTKLMKNKKPANASLWVFLGGMGFTITDEREAEANFTSYIAIEPYVFLVSRPKYLTRALLFLQPFTSDTWLCIIIAVVIISPLLHVIHNVSPFYEHYESRSACGFHKMINCFWYVYGALLQQGGGSMPTADSGRLLIGTWWLVVLVLVTTYCGNLVAFLTFPRRDSLISNVDELLERRDFLTWGMPAMSLIHSILSKTNNEKLEDLLDGAKLHEELTEEVINEVREGRHVYIDRKSKLLYLMKSEFLKTGKCHFAIGDEQFLEEKLAMIVDQDNPYVELINREIVRMHKVGLINKWMHDNLPERDKCWGSLLSKEADNHKVNLDDMQGSFFLLFFGVLIAVFLILFEFLYKKWRTVQDRTYVRPFAS

>GbueIr76bI

MVTGGLVGTLLVGLCSNAIHQITESNLPFNCSLKLGGHVKKNSLVGRKLKVLTYDDAPLSYAEIDDDGILRGKGVSFELLETLQEIYGFEYEVLKSDSKFIGDAASGILGEIASGEVDMAAGFLPVLPGTNKYIKWGTDLTQYKYYVMMKRPAASATGNGLLASFSMEVWFLILANLISVGPIFYMVMWARKRLCEDHSVKMYPISSCVWFVYGALMKQGSTLNPETDSSRIIFATWWLFILILTAFYTANLTAFLTLSIFTLPINSLDDVAKPANKWFAASGGAIEFSIKNELGDLDALKPSVEKNHGKFVEVDDQKVLNFVTEAKESFLLRSVAFAYPNGSDLPTYFNPILQDFVESGIIKHLTSMNLPVSPICPLNLGSKERTLMNSDLWATYTVVIIGFVFAFVIFVCEVALRCIDKNIKRVKKVEDKPEIVSTKFNNNKMVSFSESITKNINGRDYLMVTLKNGQSQLIPVRNNSAVLFHYR

>GbueIr68aNI

FEWLVSDVSLKFTHLTCLTLFTDSFYHSTFSSKFFLRIGFVPVFKVLVSDSDRLSSPRFSILSVLQESRRKGCNAYIILLANPLQTTQLLVFGDRYSLSIWKYRSRKGVDLYRDKTSDLRDQTLRVVTFPYILSVVNRSLQDSTNLTTDPFTRYDGLEIMMLKALGEAMRFRPVLVEAVGGAKEGWGHLQVDGSWSGLMGAMTKGTADLALASLHQTPHYLAVMDLSLPYSLHCLTFLTPESTTDSSWKTLFHPFPLSMWLGVIGSLLIITLVLRSLAVADDKWSEDYRNHNGNKANVTDHTRIGYPVPVVWLRETSTRISPVSGRRTSRRRHVVWTKPPGRRQRVIGNVFLILGFLAGVYPQTRLGLPFQVSLARLPEGISLRVLIGFSWMYFVLISNSYKAAMTAILANPPPRVTIDSLEELASNSLDCGGRGEQNKELLLTSLDSASRIVGKRFRVFHDITESGIDLVAMGKFAFYENIYLLKHALATRKRNTTVNKTGAILGNGSGEEMHIMSRCVINMPVSVGLSKNSPLKPRVDVLITRLVEAGLVNKWLADVVQIHRSEDDGSQEALVDLKKLYGAVVLLGTGYLLGFTALAAELLYWRFSVQKSPLFDKYALHLLYANKAKLVNKM

>GbueIr21a

MHKATVNYCNIILTFFQLGEELMSIDQPCSKNGVPNLVRLIEYIINTHMKSDTPVILFDEFYSENANFHVSLKYLQMRSSSPLRYGKIMTTLAIPQINNQLFKPDWNEHLSFFIIIKNLDLASDIIELYVKSCTKVVIMSQATKFHLKTFLSSSTSRLYPNLLVVEDPTMQLDLFDNLTRSEVSLFPNKFKNGLRGQHFVVAAAEAPPFVLRKRGQNSGEGYMVTKWEGIEIRLIESLAGILGFTYEFVEPQDSLEDGADRVLNEVLAGKAHIGIGGIYSTEDRAKLFSLSVPHTLDCAAFISLASTALPKYRAIMGPFFWDVWLALTATYILAMFPIAFSEWHSLRPLIDNPSECENTFWYVFGTFTNCFTFVGKNSWGHTKNVATRIFIGLYGSYWAFTIMITAAYTGSLITFITIPEMPEVIDTPRQIVEKDFTINTIGGWVNLLRWAEDATGIELWKRHHTVPDVLTGLNNVTQSIKLNEPTAFLGSKEHLDFVVTTNFTTNLGSKKTVFHTSRVCFTPLLVSLVLPNKALYIEEINIALIKAFQAGFIIKITNQVHWLMYRSSTGKLLQSVISKSVLFRSGDRQLTLDDTSGMFLLLGTGFSIAALALLIEIITWIRRDIKQRKFGHPTIKKRISDYCIDNCRKLSALFLSPGKEYADEQDAYIWHYIYNQAKRRLSAVGWSLFGSPVRQASHPPSPHRLPPPKISITRNSF

>GbueIr40aNF

TYFKLFVLLKEALYLGPICKLESLSAQVHPDSKNQKARFYPINGRHLSSALRDMISAMEGTLVSLVADLSSERSLFKRVAGKLFKSEMTVSLVDLNLHPGLANENLARRSIVFMFVWRRGDVSKRFQDSVQEAMRVCLLRQPRRATLNVLCNQAKSDESQELLLVNWWSLKDGLFYLPLLPPAQTVYKDFHGRIFNIPILHKPPWNFVNYNTNNTNGIQVVGGRDDILVALLADKLNFRYDYFDPPDRSQGSSLVNGTMPGVLGLIWKKEVEMFIGDLTVTYERSLGVQFSFLTLADNEAFITHAPGKLNEAFALIRPFHWKVWPLLIITMILSGPIIFFLFRLSDWVNKKDNKESLSDCIWLTTTIFLRQSTEISIDSIKIRLVITILYMATTYVIGDMYSANLTSMLAKPAREKPITTVEQLYEGMISQDLKLLVERNSASHAILVNGTGIYKKIWEIMESQNTNLINSTEEGMRKVRSRKNYALIGGIKTFDYDSRRFGAQNFHISEKLYTRYSAIALQIGCPFIDNFNKVLMKLFESGIYSKITQEEYHKLRERQLNEDNISQDNAAGSLNKQEDDTRLTAMNMRLLQGAFYLLIAGHLISGIVLLTEMLSQDNIEIKLKMIKSFEQHTKKKSKNSFVFLP

>GbueIr41a

MKLHIYILYIYSISCSFSELTPERVEIDNLEFILNDFISQMLERYFTNENCLAVIGNRYLTTRFNLTVNFIRFGIEDGDSLDHCVKDIRSFLLFALDHNCSSYILISKEVTCFWAGYEWARNNSIVRQQPKFLFLPQHFSHNLTDDLFSNKNTKLWPHIIGADFSNQSDDNITRLLLYTNDFHSRDNYDKILLDEWSRDEGFIDNNELYFDKIADLKGKVIRVSTFSYPPYTMFDPLDGFEGGLIVEFCKIHNCTTKTVSDEGHLWGAVYSNLTGNGILGQIVTDRSDVGAAALPISPWENSVLDFSSAYFSSVVTCLVPRPKQLPYWMVPFMPFDLKMWLALLASLMIAAISLRIVTRIAFLYTRFGPDLKAKGQFFSMGDSIMRSIGLVLAQSPSSRMIGDSPNKHLFTSFEILFLFLVACYSAGLEAYLTVPVYYPPIDSLDELISSNLKWLAEHEAFLYCLQYDMDPKIVDLVNRAVIRPSDELLKLGLTGEYAIALEILPDGSSPDRDHLSEKVISLSHLMRGRLYDSPVGTAFQKGSPYLPGFNSMVHSFMDTGILFFMISDAARNFLSHGKQSALKHSRVTHDHPEPKVLKLEHIQGAFSLFLGGLLLSIIIFIIERLAFKKVKKEKQTPFAIEMS

>GbueIr41bC

MNNWNILIIQITFYYFIIFRCSSAQTQDSMTDNADGANNIYSQAFIEEPNGDLKVIMSRIVLKALETEMVGYRCVLWIQEPDMAWSPELDMATKRLAVMTMDLNMESIYYDPRLDNSSTVMPLAVYTESNLQEINLYPDKIKDLQGKQLVFAILEYPPYGMLEPLDGMEVRLIQYFCKIHNCTLVPLVDDYAWGDIYPNKTGDGILGNVYTDKADFGTCAIYLWPNHYPYLDYSSAYSITKVTCMVPKPLLYPEWQIPLHPFSRMMWATQIIAIVVAALILFYINKVAKKYEEEKGEAKATEFDSLSGNLLRSLGMSVLQPPSGGPIKPDSPMRSFFTMFE

>GbueIr41cN

FAAELKEKNQFFSVGDSLIRALGLVVLQAPSIRMIGDSPNRYLFTSFEILFLFITSCYGAGLASNLTVPMYSTPVDSLADLLASGMNWLVEHEAFLYSFKDVHDQDIVTLSSRALVRQPEELIQLGLSGKYAVGLEVLASGSPYINELNMLIYKILDAGIMEYWEGDIARHFLNSKIQSGLKRGRTLQHDSEIVESLKVKHIQGTLFILIVGLLLASVAFIVEIFTYKKQQRQKLKDLAIIIN

>GbueIr41dNIC

MFLVQFITFCLLCSISYQQIIKFENEFHSQLSANLMNFTKTILDRYFYSDRCIVIVSNQPLSIQIDLSVEFFEIHDDLSLEECWQDIGALLLPGFKFNCSSYVVMTSTPLCFWKGYFWARNNSLVRQYPRFLFLPPVDLSSNNSRELSLPRYDDLFKINQTEISPDIVGVYIDNATNQDSEFKMKLSIYTNNFYVHTKDSDSREPSILLDEWDSERGFYVGTNLFYKKLNDLQGKVIRWTSWSYPPFSVAESNGIIDGIEGKFARLFCELHNCTLKRITDEYMWGTVNENMTGNGVIGQVFTEEADMGGCGVYLWPRELWFVDWSTAYTTSVITCLVPKPHLLSGWMVPFLPFDWKMWAVVFGSLLAAAASLRIVTQIAFKYTLQNDVFLLATILKIINLKFSKPIQLSQMLRCTCELNFVQIGYAPEQPYITEELVDRSHIMRQHLYGAPLGIVIQKNAMKSGRNKCENSRSLVHTALICLYFYLDLVFYVTFLIKIILKGLFALNGSNTKKQIGKNKRKTRGVSSN

>GbueIr75a

MVSSAFNMINIIIILYFYPVILVQGNKSFQETVIRYFSDSSVKSVDLFYCGEEEVDVQALYKAGPSNKSIKLHVINNKNITKFDYIKSLQEEPTYNYPLGLFIEVTCKEGISLLHEFSLKSLLSMQFYFLLYGDYDTSLQILNETNLSLQSFVTLITPDGKLLSLYKLYFDTLLIEENIDSNIATDDIIRKRLTKKNFFGYTMNVTYVFPDLNYVKDHSIEFLMDHSLENNKLPLFRLYLRDFDNLKEMLNLTYSYAPVDSWGWPDGNGGFTGMMGRLKDGSSELGMTQVAIQLARFPAMDYSNQNNVDKIIFTFLQPRSFGSYKALAVPFRLSTWISYFAINFIFFMSFRFFALYELSPDIKEWAGNFELVVRTLSQQGIEDTLKLSSSKLVTSSLLLLSFIAYSYYNAAILNGFLSPSASTINSKDDLLESGMPVILHDAPYCKDRSNGSVFGTDSPFRRRLYAAMSKDTGQPYYSTKDGLTKVATGKSAMVIHEMSMYDDLRALFTEAQVCSSDNIVLSLIQVGFVTSKKSAFKEPLRQAFSIMHERGLLKRSRTVWYTPKPECIAAQEAISIGLSSLIIAYTMLLLGIIIAPFIFVTEVITRRFETKQFY

>GbueIr75b

MKNKVLVLTLLSLKLCVCNGYPNNLADLIAERISEENLNAINLFTCAEQDWIFRLLEPNVLPNTAVSVDPWQRDDFRSRCFDKEIGKIGMFLDLTCNNTEKLIDDLSECNKFSGSFKWLVWTDNLLDGLSRIEQAKISVDSDLTLIEPEFILHDIYKVHYQAPVRSTRLGTWTEEKGFIPEKPKDPRTDFGGFPFNVSIAVTEEETNVSIPVEMLADRLYKEDTSPETRFTFNMFLHIKEKFNMRYNYKEASEVGGDLPGSWNGMVNDLAVGEADFGLHPARLRQDTMDYLQPVFTARIVRSFFSLIQPWTFSTYRALIVPFTGEVWAYFGGIIALISVTYRVISKYDKSPDIDSWGATLIQTVGAISQQGFEDRTFLTSSRLVSFFLLLLSAIAFVYYNAAVLNSLLSPSPNTILSVEDLLYSGVELGAWDVPITKDHQSDSSIFGTESSLRQEVLDKLNFTEPVYYKLEDAVKKLRGRKFAFMSDDSLIFRPADRMLTDAENCAFYQIDTVTPFHSTLLLIKGSPYTETLRRTFIWMKEVGLQQRALKLWFDEKTPCRWRQEAVSIGLEALFLSYFIYFGGALLSFLIFIYEYVNKPKSLVSG

>GbueIr75c

MFGEKQNFFSLVSPLTGVIAFKVEIELLFFINMYILCLHPKDTTDVISNFKFGSNNSINSVKYFEILSGIEDTRMLSSVSYHNRLGVFLDLSCQYGIDFLRKCSKEYLFTGRYIWLFWNKNYEDAIRFLDEINMSVDTDAALVDSTFVIYDLYKIHYSMATISTKIASWSPGKGYVHEKLPPLRDNLQGFTMNISAVLPNLYYDENTTLEDYMDPLYIPQVALVSRETFSLAVHLKEMLNITFRNRFHDSWGYEQTPGNFSGCIGAVIRSESEMCVSGINIREDRLQYVDFIYTVRMTKSGFSFLQPSSFGSFKALTDPLSNDVWAYLGLLTLLFIFTFRLLARFDPSPDINTWGNIIIYATGAIAQQGFEDTIKLDSGRILNMSLLLMSFIVFTYYNASVLNALLSPSPNTIFTIKQLMDSGMKLGLLDVPYNKERTSNESLFGVKNDERDRLYKIFNETSPLYYQHDEVIDRVREGGFAVYSLVESFYPRVKATYSDAEICSFYHISRIPPFPLALAIRKHSPYIKLLRQAMFRMSQGGLIDRADKEWYVPKPRCQWRHEATSIGPESLAVAYTVLLVGIVASLCILAIEIFYSKINSSTIHEVGSTRTKKKRSKVKKMTIAPSPIKYSDKF

>GbueIr75d

MLIFCLFIIGFTKVVCSFETLPYDIVSKYFVYKKNLDWIDYFYCQNENISNSSLIDNIFKSYKNENNRTPMKAYIGYDEYLLLDTFVNVRGGIFLDLSCPKANLILKQSSLYRLFSINYDWLIVTDSIRNIYNLSDLTLSMNSQVTLMILVEMPLSRNISIFNTYKFYDVYKYHYSKSLILNQEDLFNSKEEIALKNNMAIRKNFGGIELNVSFAFIGVKQDESNDLEIWANQFYRPNTPPHTRSLITLFQHFRDFYNVTFHYKPTDSYGIETEMESGNWTGMIGQLSRHETEFCIQESRMRYDRMLVADVIITTRVYSFLQPQSFGSYKALIEPLAWQLWLGLFAVSVLLTIKFKVISLFDSSSDINEWSAAALHTIGSITQQGAEQRGELVSYHLATLVLLIFSFLATVYYSGAVLNGLLSPSKSSINTLQDLLDSNYRLGLWDIVFVKDFEGPYNPYGPHTSVTQRLFDKLKIADTLKQYYSIEDGVERLSQGRFAFFSDDANLYTIINSKLTPTQICSLTHIKTISPFHLGIYTVKKSPYKEHLLILFQWLREVGLTSRAQNIWYEKATPCLGTFEAVAIGIKAISLGFFILVAGFLLSVLIFMLEINVSRRSKTLK

>GbueIr75e

MRCSCVGIILTAIVLSVQCASLFDILHYFSAKNVSAVHIYLCEFSDFLEQELIVSLRSTPLLASLNLMVQNTSIDRLIVDRAMPNRIGISIDIKCPSTKFFLNRCSEKEMFSGSHAWLVWSSLEEGLKVLDQLKLAVNTDLTLASWDHPKKQAFSIYKILYNSSIIYTPIDMKAPRKEIVRDFRGNTIDVAIATDITLREGESFADLSNQMFRRAESSPGHRATLLQYDAIKETLNMRYRYWETKSWGTDTGNGTWNGFMGLLQSRKAEFSLNFCKLRKDRMDIAEVFLSIQHSRSLLLFVQKPQFGSYKALVVPLTYNVWISLGVTCIFFIMMYRFISLFDDSQDFNNWGAIIILTIGTLAQQGFNDTTRMASSRIVCFSLLIFSFMAFLYYGAAVLNGLLSPSLSSIKTVKQILDTDMPITILDENYMHDFNTDEGVFGPRDAETLKLFSRFAGSGSKFLKMDEGLKSVMKGGVIFFADNHALYTALSQTVSSSEMCSFVIIPKNFPFQAGIAARKGSPYKEMITVIFARLMETGMNERIKSLWYDKKPACAFKREAISIGMEGATVAFLVLLAGFILSALVLAQEFYSTRVKS

>GbueIr75fI

MLPFVAFLFLIPFGVFSSTSSTLEINWKNRFTAIEAFIKTYFIKPNVREIEFYFCNKSLKTDQLSLYFRGFHIDKPIGVYSLSERSESNYLIGPNKSKRNIGIFLDLSCQEGLDFLRKSSDEKLFGESYSWLMWMDVYRPRNTLNDLALAYDSDVLIMEPQHNRILLSDVYKVHYTTDLILSKLAIIDFNDTLEKFADRSYYPDTKPTPRFTISWFLDIAQMYNFSYNYTSVTLWGSHDPETGQWTGMLGIIERGEVELSMTVVHIRKDRMHFGIFITKFIEFRMFLVFLQPAIFGTPLAIIRPLSTASWLCLAASLPLAAIVARIFSKYDSSQDIDSWSSIIEFTIGNLSQQGPNDNMQLISSRIMALTLFMMSFFVSIYYSAAILSGLLSPAPKSINSLDKLMDSPLSAAISDIAYLKDFSTEGVYGQMNPNKQKLFKKVEAEKRMFLPLEEGVERVKNDKFSFLVDDTVIFPVIRRAFDASQICSMDLIAYISPKVNLGMMMRKNTPYREIFSQKLLWQEEVGLKYRNKKLWVDSRPACIANQQAVSISLEALCLAFYMLAAGSTAALAIFLIEFLLYKHLERKTN

>GbueIr75gNI

QVFIKNGSTLSNVQTYVKLIQLTNGTESEDLYDVQRKFLNIGVFLDLTCNEGVEFYKKASMKIGFMGRTYRWLLYSESDDAPLWLEKLNFGWDSDVTLLSPSGVYDLYKVHKTTPLLMNLRAPDPKAVTEIYPKPDRRNLMGYQMNAAHALTNSRDLDMFEDRFYMPDSHPVPRFTLSWYFDIAKKNNFTYNYTLVTSWGNEDDEGNWSGMMGVLQRYEVELSFSQVYVRRDRMSACDFIFNLKEDKVMFVFLQPAAFGTSAALTMPFSFEIWMYVGAFLILIICVARIFQSFDDSPEFDSWGSIIEFSMGNVTQQSVHGVYGPMNEKRIRLFKILQSQERMFYNIQDGIERVKVGRFALLTDGILIFRMVKLRFNDVQICNTFIIIHEIPKMNCVTVINKITPFRELFSRQLMMLEERGLKDYNYLAWIDKMPACNFQQEALSIGMEALSLAFYILLIATCLSLIIFSLEIIKTRIENLRSNKVDEKNSKKGKINVKFVIKLPKSYKF

>GbueIr75hJ

MDKILLFIVVLKTLLSVKSSNDFDSVRNLFKLKKVYISELYVFACQYNENSPTLREEPVTKSYILDGSEEVLSILRTTRHRSRYGVILDLSCAFSRQFLIQSSEYHLFNTSFTWIILQGQEDDLNSFSDLNISFDTDMTVITRDGRLVDVYKPHYDGNLLTQVLGHLGNENLAKKNVRNGFGSRLNIALMTTNRWLEDDQVEILADPLYRPQDHQGYRTGVPFALAFRDALNFTFNIIRTDEFGARSDSGNWTGFTGLYQRGEIDLSFHQLKFRKDRMDVADIAFPLQTGSTVYFFLQPPMFGSARALIVPLSREVWAILGFITFLFNIGFVIISRYEKSPDVENWSAVMIHTLGTIAQQGFNDTTQLVSIRVSSFSLLMFSFLLYLYYGAAVLNGLLSPSVNSIKTEEQLLDSGMKIGILNRTYMTEFNSDESLLGPHTRQTLRMLETAKRQDRMYYGLNEGVEMVKKGRFVFISDTMALVPLLGKQLSDVQLCSAFLINKITPFSLGLNLRRHSPYRVVWMNKMVWMKEVGLVKRTLNYWKPSKPPCVWHQEALAIGLEATSVALAVVFLGLVVATLTLLVEIFLSKKQTL

>GbueIr75iI

MLKDKVCELVLVCIVILTAVRISEGLDSFKNIKKIFDLKNISISELQVFLCRYNEDVSLAGLREEPATKLYILDGSEDALSILATTRYRTKYAIILDLACIFNQQFLIKSSEGFLFNVSYSWIVVNDKEDLGFLRDLNLSLDTDLTVVQPDGSILDVYKPHYNNQLFLQFLGRLPTNSLTATNKRNGYGSMLNIGLYRIVWSDSWGINPEKKGNWTGFVGLYQRGEIDLSFHPLKYRKDRMDQTDIGFALGQGRFVSISFITSRFDQSPDVENWGAVIIHTFGTISQQFSLLMFSFFLYLYYGAAVLNGLLSPSPNSITTEKELLDSGMKIGILDKPYMTDLSSDESLIGPHTEQTVRMLEKAKKQDRMFYDYDEGMSLVLKSRFTFILDTMGLIPGLSKKLTGVQLCSAYLINKIPPFTVGLNIRRQSPYKQEWMRKLVWMKEVGLVKRTGNIWTPSKPPCVWRQEALSIGLQATSIAAFLALAGILLSAIILPLEIFHFKLNKTV

>GbueIr75jNI

YLFINSPNEIGLNTTTRISVLNESNVKRGVDENLLKEIRFIGIYVDITCADGEKFVEQSALERKLFSDNYKWLLKSDSYEASASLLNNLKISIDSDVSLAVHYNDSFLVYEIFKIHESLGIESYPMAHFNPNMERSSTGIKPNREDLKGFVMNVTFVILQNHLTADQPVEILLDPFFNPELDRYSRFSYLLFIHVSEVLNFTAIFLFFQPRTFGTYKALVVPFSSTVWICFGLISVILPFVYGFIAKYDESSRSNDLSSWSSIFIYLAGAIAQQGFPESTQGLISTKVASSFLLMFTFILCLYYNAAVLNGLLSPSLPTITSLESLIDSNFKLGVADLPYMRDFDSEESVVGYHDDLTTKMIKKLFHYDRMFYGLEEGLEEVKKGKFAFFAVDDGILPLVTNTFSEAEICSTHILIKPPKMNVGLTFKKNSPYKEMIRNKFSWMREVGLFKRNLDIWYPGLPKCTWRQQAIPIGSEAVSIAFLVLIIGYVLSLLIFIAEFYIKPETVMKFNIF

>GbueIr75kN

FLLNNSPNESGLNITTRISVFNGTKMKRGIDANLLKENRLIGIYVDITCTEGEHFFEQSSMARKLFNNNHKWLLRSDSYEASISLLNNLKISLDSDVSLAVHFSDSFVIYQIFKIHESLGIESYPMAHFNPNMEQSLTGIQPNRENLKGFIMNVMETDLKANQSVEMFLDPFFNPELDRFSRFSFPLFIHISEALNFTYNYLVVHDFGVEDALGNWSGLLGYLQRGECEFSLQFSKARKDRLNVVDIDDTYVKPKAIFLYLQPRTFGTYKALVVPFSSTVWIYFGFICVILPFVYRVIAKFDDSSRSDDLSSWSAIFIYLAGAIAQQGFPESTHGLISTKVASSFLLMFTFILCLYYNASVLNGLLSPSPPSITSLENLVDSSFKLGVLDLPYLRDFDSEESLIGYHDDLTKQLIKKFFQYDRTFYGLVEGLDTIKKGKFALFADDGVTLPLITKTFSEAEICSLYILMKPPRISAGLTLKKNLPYKELIRSKFSWMREVGLFKRNLDIWYPGLPKCTWRQQAIPIGSEAVSIAFLVLILGYASSILIFIAEINIKPETARKLNIF

>GbueIr75lNP

FLLANSSNENGLNTTTSIFVFNESKMKKADANLLKEISLIGIYVDITCKDGEQFVEQSALERKLFNNNYKWLLRSDSYDASASLLNNMKISLDSDISLAVSFNDSFLLYEIFKIHESLGIESYPMAHFNPNMEHSSTGIKPNRKNLKGFIMNVIFVVMETDLKADQPVEMLLDPFFKPESDRFSRFSFPLFIHVSEALNFTYNYLVLNEWGVEVTPGNWSGLLGYLQRGEHEFSLQFSSARKDRLNIVDIGDTYLKPKAIFLFFQPRTFGTYKALVVPFSSTVWLCFGFICVILPFVYWVIAKFDESSRSDDLSSWSAIFIYLAGAIAQQGFPESQGLISTKVASSFLLMFTFILCLYYNAAVLNGLLSPSPPSITSLDSLLKSNFKLGLLDLPYMRDFDSEESVMGTHDDLTKKLIKKLFRYDRIFYGLEEGMEKVKKGKFAFFAVDGATLPLVTKTFTESEICSTYVLMKPPRLSAGLTLKKNSPYKEQISSKNLDIWYPGLPKCTWRQQAIPIGSEAVSIAFLVLILGYVASILIFIAELKIKPKTV

>GbueIr75m

MLRNTKRRIVCVFTSWIWYRMGTVQGLEAGSQYLEAAVNDPTTSAVEMYLCTDKDDALLRLNMMKEVKKVVKIWKTPEITLRDDYLPVLHYLQTSCLENTTLLLNQISGMKLLDKKHKWLMEGRLEERELNALTGLRLDSNFVWLHTDDTIYDLYKVHPSQPLIQTQLHSPDYIRPTRNDMMGFPVPACLINYGDRLRPNESFEMIADKFLHPEGSPKLVALLPLLRHLLDSFNFTYNFIYPAGWGVRSPNNTFTHALGMLQNGTCELCLTIIQMRIDRADAGDMLPQLVHSEMMIIFQEAKSFGTWNALLDPFTSQSWLFTFLILALFFFTFYYSLKSEQRSDNLSAVIEYLSASLSQQGVEYHGRKTSNRLLLLCLVLFTYFIYTYYNCQVLNSLLSSSPSKINSLEQLLDTDIRFGLTDAPFMRGNLTSGNVSIIGPVDEETRVLVKRVYENEKRYYSMEEAHPLIRARRLALLANEGLLFHFVQTRFTESDVCSTVALLKYKSHNTIFFSKGSYLKEMASQKIFWMREIGLYSRNTMFEKMPTCVLKAEAVSIGMKGCTIALILLLVGIITTILIFPLELYFKSKKIGSY

>GbueIr75nI

MLRNTKGRIVCVFASWIWLGTVQGLEGGSQYLDAAVNDPTTSAVEMYLCTNKEEALLKLNLMKEVKKVVKIWKTPEITLRDDHLPVLHYLQTSCLENTTLLLNQGLRLDSNFVWLHTDDTIYDLYKVHPSQPLIQTQLHSPDYIRPTRNDMMGFPVPACFIKFGERLGNNESFEVMADKFLHPEGSDKMVALLPLLRHLLDSFNFTYDLIYHKGWGLRLPNNTFTEAFGSLHQGKCELSLTIFHVKIDRADAGEMLPLPVYSDMMIIFQEAKSFGTWDALLVPFTSQSWLFTFLILAVFFFTFYYSLKSEQSSDNLSETIEYLSASLSQQGVEYHGRKTSNRLLLLCLVLFTYFIYTYYNCQVLNSLLSTNPPKINSLGQLLDTDIRFGFVDFPYMRANLTSGSVSIIGPVDEETRVLVQRVYENEKRFYSMEEAHPLIRARRLALLANEGLLFHFVQTRFTESDVCSTVALLKYRSHNTIFVTKGSFLKEMASQKVTWMRETGLYNRRISMFEKMPTCLVKADTISVGIGMKGCTVALILLLAGFVSTILIFPLELYSKISKK

>GbueIr75o

MELNGFYLMKILLVTSISNSISGTIVNDYFDFLGVKEFHSFYCNEQDAVNELTADMLYNEPQITSHRYLNDGFNISFQGMNRGSAFIDLSCSQGRNLFLISSKLNYLRSFQPWLLWSSSLDEANLLLRKSSLGVDSRVLLVIGSNLNLDSEHRFFSIFKYHPRRDELVVRQIDSFRDPTLKNPEKIDFNGHNLSAIIVFTELNIEASTLPGLTVDDTYLPESDVSNRISISLYLLFQELYNFTSVYSVAKGWGYLQPNGSFSDMIGMIETGDYDIGMSITYYVSNRLSVAQYLPVVTHWDIHFLFRQNPSFGGPKALLRPFHSTIWIAMTLVLFATACTFAIGSRLVVRSEEQINHKDSYYTKEERYTWSSALLLLFGIMGQQGIDEESQKTSLRVLSLSCIVFSFLVYSHYSSEILNGLLSKSPSTIKNFQDLVDSNLRLALWDVPYVKTDFNKTDPISRSLVMKGQKQPGGLQLANLVRGVELIQTGHYAFLTSPPVVYSQIFNKFSNNEICTLYSVTKPDLLKIAAPMLTRISSFREISFIGYTKLKETGLEKRELIRWLPPRPPCLPEQEFNSVAPESLMIAYLILILGLLSSTIILLTEILNNGNNRSGYIKCFRAVKNNLDRKTH

>GbueIr75pNIC

ADNFVALSPQYDVMVKVHTTEYLNITEFNLPFVTRAGVFIDITCQQGANLLVNQNHIPQSYEERILNATITWLLWSTSVEDALTLARKSNLGPDSDVTLATPEPTLYRLYKFHPSLTDVFMVSNGFWTYDDLYLEYFNYHEINFQGMRYTAVTRGWGYLMPNGSFTGMFGHLQRGEVELAVVVSEQNAERQIIASFLPPLRKWIEDDSQNGSLRLLSIAAIEFFFLVVAYYNAGILHGLLSPSPNAIQNLDQLIESSFSLGYWDSPYVKHRISGNQTELARKLFAKSRNVQHGGYYELDLVAGVEHIKEGGFAYLAGEGTLFVEILKQFSNREICSLSVLTNPDWEYYASSMTKRFSPLTDFFRNG

>GbueIr75q

MVWQIKLLIFWYLEVLIKFSSSVPLNFPYQFFIAKHTKHLTAVLCNDFDSDNFVALSPKYDVMVKVHTTEYSNITEFNLPVETHAGVFLDITCQQGANLLANQNHIPQSYEERILNATITWLLWSASVEDALTLARKSNLGPDSDVTLATPEPTLYRLYKLHPSSANVFMVPNGFWTYDDLHLEHFNYHEINFQGTHINCTAVLTRLNATEEEIPGRLLDVFYQPEVDDSSRVSIALIQQAAVRYNITMRYTAITRGWGYLMPNGSFTGMFGHLQRGEVELVVVGSEPSADRQRIASILPPYIKWRIVYLFLQPDRLGGSLALALPFHVNVWACLAFSILLFSILFVLSSHFLIPQETKYNYDPNSYYTHEETYTSSSALLLTVGLLAQQGIEDDSQNGSLRLLSIAAIELFFLVIAYYNAGILHGLLSPSPNAIQNLDQLIESSFSLGYWDSPYSGNQTELARKLFAKSKNLQHGGYYELDLDAGVEHIKEGGFAYLAGEGSLYAEILKQFSNREICSLSVLTNPDWEYYASPMTKRFSPLTDFFRNGLAVARERGLQDREDRRWWARKPKCLPQTFLVPVSMESLSLAYFVIFIGLTFSSVILLFEIFHYKKYGG

>GbueIr75rC

MDWRINLLIFWSLDVLITFSSSVPLNFPYQFFIAKHTKHFTAILCSDSDADSFVAVSPQYDVMVKVQTSEYLNVTGFHLPVATRAGVLLDTTCQQGAHLLTSKNHIPQRYEERILNATITWLLWSASVEDALTLARKSNLGPDSDVTLATPESTLYRLYKLHPSSANVFMVPNGFWTYDDLHLEYFSNHDINFKGSEINCTAVLPRLNENKDSMPGRLLDQFYHPGVDEASRASMAQVHSAAERYNISLRYTSITRTWGRLFPNGSYAGMLGHLQGDDVEIAVVGSENNGERLSIASYLPPIIKWKISYSFLQPDRLGGSFALLWPVNVNVWVALALTLPFFSCLFVISSYMLTPLDVRGRYDPKSYYTHEETYSPSSALLLMVGLLAQQGIEEDSQRISLRVLSFAAIEFFFLMISYYSAGILNGLLSPSPDAIQNLDELIDSSFALGFWRSPDVKQRITGNRTELAVKLLKKSRKNREEGYNELELVEGVRRVKKGNFAFLAGEGTLYAEVIKQFSNKEVCSLSVLTYRDWEYFASPMAKRFSPFTDFLRNG

>GbueIr75sF

MLYKRIYSLYLIVLTQIIPVYVYSLSSSIPFIELYFKDKQINGLHKFVCDSEILELMENESEPYSSSSYLIPSGNNFIGALRNDSFHRIGIFLDLKCETAREVLKKCSDEYLFAMNQAWLLLTDSYNEAVDWLQDINLSLNTDLSIMLTNGSSSYIMYDVYKIHYSTPPIVTPVTVINKNGNIPLKPTPVRTDLMGFEFSVVLTLLDLELKPGEQIDVLWDLSYQYNADPTVRYSLPLFMALKEALNMSYSYKIVDDWGEDNGTGKWTGMIGELQHGGCEFSLNVAKIRKDRLEVMDNIVTLNKYRSRFIFHQPKTFGSYKALVIPMNPEVWYSIGGFVLLYILVFNLISSYDESEDFNTFGDVILLTLGTLAQQDFNDTKMFSSSRMTCFTLLLLSFTACLYYNAAVLKGLLSPTPNSIRTLDELLESNMKLGVVDAPYTKDFESDESVYGYHDESTIRLINKIKSEDKMFYTFNDGLDLVKNGYFAFFVDEGPSMRHIINKFTDSDFCLAYVIEKNIYQNAVSLRKYSPYKELLARKLTWMLENGLRKRAVDNWLLKMPECQWRQEAVSIGIEAVIVAFITLFLGMILSLIVFFFEVFTFNKKKIIKSF

>GbueIr75tI

MLLYAGLLVLCFINSTNTMSYSSKMIKDFFKTKTITIAHLLTCSDEDALKLSNELITQLGNDETKMAVSSPSLYPHEWLYPMPYRMGVFVDLDCNETIDTIFQLSNNYTIFNGSYVWLCWSEHKHSVEFFSELEIYLDSDITLLIPKRDENTTPVPVTLFNVYKAFPERPLNVTEIGSWSMNSGIFINLYINRKPNFEGIRLSVSMVLTALKVDMKKLGEQISDKSYHPELDIVNRYFYVLNEILRIMNNYTWVIVMTNSWGYPQEDGHFNGMIGAVENGTAHFAMGPAEPRADRQTVADFLIPLHGFRTCFVFRQPKVLGTYKALASPLDTKVWTYLFLIMLIAAVTYKIIYSFRTRYDNENEDSLFWGLLLTIGNRENPLAAAAYEKMMKQKPKGIVDSLSTGVASVRGSRYAFYAEDTILYSEIDQTFRDVEKCDLSELEMLRPYPVASSIKKGSPFKESLNKNILLMMERGILFREDKVWYAQRPNCLGSQDILYVSLEAVSIAFWVLSTGFALSSALFTLELVSGWWYTSNNRNKGKNKTTATKRVNFY

>GbueIr75u

MFKNLCALVIVQQIYKLYSCEAFITSNGQTRPIFKDVINNYFERGQHSGIMAYLCEEYDFIAMDLIKSIKYEIMETSRYKSNEKEFFETLEYHNTGVILDLTCIEGTNMLKKLTTKNVFTDLQRWLFWSNSFQKTLGALSNLDMSIDTDAILLISDIKLRKPSVLIYDVYKVHRTNPIIYHLIGYWNHEENKIVQMERPTRKDFQGFKINVVHVPLDLAVNNTVDALDKHTDMFYEPTSNPIPRYSLSQLLDLRGIYNFSYNYTFVESWGREDKNGNWNGMMGVLQRKEAEFGFAHVYMRRDRKRVCDFRYSFIDIKILFVFIQPPMFGTATALTAPFRQDVWIILGLSMIGMSFVIWLILKQEEWSETNLAFSTTYVFGCMTQQGIDNYFVKFSGRQTTLTLLIYSFFISLYYNAAVLNGLLLQIPSPITNLRKLLDSKLDLGAFDEPYFRDLSEDGLWGPRNLKRLTLEKRMTSGKHGMYYNGEEGIEHLKKENFALLIEEFSYHRNILPTFSETQMCSSQEVKHELPRVICGVAFARQSPYRNLLTTKLIWQDEVGLRKLNADKWNYKFKPECGNHQQSISIGIPGLTVAFQVLLLGLITSVFIFIIERFINTNQILKLRRNQKFSKSI

>GbueIr75vJIC

MVSSISYGTVIIIITICIANNFAINFNEEYFSRIINAYLNDGHYEGIVAYQCSEGHSLNGYMEKHTKIEELNHNASINQLILSQNIRENRLSVAVFLNLSCNGSHEILKKSSERKLFSLQYRWLMWSRSYTESIRRLSKLNISIDSNITLAVLEEEGCSNCLTTKFYDVYKVHYKVKEVISNLAGLWTWHSGFRIYERQLRNDFRGCQINAGHVYNYTFVDSWGFNHDDKGHWNGLIGLLQRGEVEVALIQFNFRTDRMDAVRYYGYYLNTKLVFMFIQPAVFGRPTALITPFSNTLWFCLGISVVLISLTMWIISKWDEKQREKTYVDNLSIVVGHIAQQGPDNLIGKLSSYIVFTILYFFAFLVFNYYSAAVLNGILSPAQSPINNLEKLLESDMRLGVADYATEKGMWGRKNKLRLELEERLKADNIGKHSIYYGEREGAEYIKKGKFAFATTLLTFYKDASSPHLYTDSEICNLKWFPHNAPKLTLGGALAKNSPYTEFFTI

>GbueIr75wF

MVSSIHCGTGIIIVTICIANIFAINLNEKYFSRIINAYLNDGHYEGIVAYQCSEGHSLNGYMEKHTKVEELNHNASINQLILTQNIIENRLNVAVFLNLSCNGSHEILKKSSERKLFNLEYRWLMWSRSYTESIRRLSELNISIDSNITLAVLEEEGCSNCLTTKFYDVYKVHYKVKEVISNLAGLWTLQSGFRIYERQLRNDFRGCQINAGHVLMDLLVSDDVPLEKLTDIFYYPDKIGVPRYNLVLFLELRRIYNFTYNYTFVDSWGYNHDDKGYWNGLVGLLQRGEVEVAFSHFNFRTDRMDIMRYYGYYFNTKIVFLFIQPAVFGRSTALITPFSNTLWFCLGISVILISLTMWIISKWDERQREKSYVDNLSIVVGHIAQQGPENLIGKLSSYIVFTILYFFAFLVFNYYSAAVLNGILSPAQSPINNLEKLLESDMRLGVADYAYLKDRTEKGLWGRKNKLRIDLEERLEAGNSGKHSIYFAEREGAEYIKKGKFAFMTTLMTFYKAASSPHLYTDSEICNLKWFPHKIPKLNIGGALAKNSPYTEFFTINLIKENEVGLIKRQLYLFFYRYTLKCNGLTELVSISVPAMRAAFLILLFGFILSVIILLIEVLKEKI

>GbueIr75x

MQWVHFFKMSYREVRKRCGGSKRLCLIYSVYISLRFTQRRDGVRIQLTLNHLNVSVWVKSFDHKLMTSINTIPTSLSTSNIIFTQMDCQQSIAFFEQCSKANMMRKGYQWILQSDSDEVPLEALNQTDIDIDSSVIFLSGIKVYQIKRESRGVTKTPVLNWEAKSEKLNLNSRTLTAAIIFLKFLKTTQRKLTLNKLISVLNVCKLSFCSCVKIFITHYSIKDYDFKTTLKVMSASRNKNKKFLIKKICNKTLKTKTRITYIIKMEYTKTIGYYDEVEPFKKSQVALLKDRKVDLGFSAIELLPSSVAYIDWLPPVKTVEQAFLLVHPHIFTQSRALFKTLEPIIWWSLLTIINIYATGLTIISLFKSKDYGGSDDLKWSGEFLQILGSIAQQGLSNPSMRFSKRVAHFFLLILSMLGYAFYNSALVIFLLSPVPQTIHTVNDLTDSGIELIAEDLPINKFHLNNPIYPEAQAAYEDIQKPTFITGESKFYKLDEGVEHMKNSTSSLYSIKSNIYEKLDDILNDDEKCRLFKVKVHSTFQIAVPLVKNSPFKEIFTQGVLKMKEAGLIYREEKYWFSSKPICMGSIGTVHIGLSTIAVACKIYLFGLLTSITILALELFWAKYVRLNMSIKFKCLAQ

>GbueIr101

MILFLRRIHIRIFVVAIVLFKVKKCDENISILYDWNIYNESSTNESSFDNILVSQSRNESLLDFQWLLFEENSTFFDICYFALNISYHSSTAFILHSHSDFVQNSVLFYYQKYIGPVVLSDKVFFTDGVTVMFLSNDSYFFNKTDKVFLDLSVFYIFIVENVFSNMHEIFDHLWEEYRLYNYLMLCGSCNDVIFEFDVEWRKVIELKSENLPSRIYHHLGKLRHWHRTDIKTVVFARAPSLVFEREGLELKGYDGMVLNSMGKYLNITLLLFHTMDEERFGYSLGNGTFVGTMKEMVDRTVDFTGNAHFIKWYNIPFLDFTHYVTSEKLCISVPKKGQVPNFFAIFKTLNPVSAVTFFATYTSIGFLYAGFKLLYVKMHIYRSCNNSTALQMFLMSIGGSIPFKGNLWSEKLLLGPLFIMSLFTVNTFQSTLMTLLTTPSFYSDINTLDELRGSNIEIWSSSKDLSFLDDVGLNIVVKSQKVMIEAQKNDRIGRIIRFNILSGNQDTYGGLKVLTPYHVVGECLGNFYVSYMLPKGSPLMYGLNWLVSSLIESGIIIKWIHETDKLTATKKPNTKQKLNVISNSFGLSDLSAAFLFFFIGTLLGFVAFLAELFVFYKVSKSNVVFVKTPKDKPKTIRNKKTILPYLD

>GbueIr102

MLIKLLIIYGLYNFSTINGKEIEIGKFELVKMNGSQIWVDVAKLLLETPSPQTGIVLLFEKIIGDKYVKPITDELGPVTLLTFAQMYTIAWTKVKLLIILLENVETVYNKVVESKIVLNNIDHLIILKSKADSYEDNFIEIWRTMFPRGIKVLRLYNGNMLVRFPYPIHYCTEIECDSFTVNGTNLNHFKDILNKKVINMNRRELKVSIFERKPSAILLPNGTFTGFDSEVLNILQQKMNFTVNLIQGTNPNCRYLYKLEDGTYCGAISDLIYDFADVCFNNMFLKNYGVKNITFSVSVGHDKLCVVVPPARPIPQWLVLFSTFNLDEWFCIIVMYFICTSVLYCMERIVNGPLNSYFDSMFYSFAYIVQSPVSRLPTKNSVRIAVITATFCGFLITIYFQASLLTMLTKDIYEREINTLEELDTSGLKIYSSSANLKDTFSQDSPISQSLSKKFVVNENSVTNYVEMVASGNRMAAIGRKYDLGGKFGFDLRRKIHLVKDCPRIYFLVYPLPYLSPYFESINSILGRLFDSGILRSYFVSYSFTPLSNLKKLPGSTNYYAPLNLTNLKILFLILILGCAGALMVLVIEYIHALKHRQFRM

>GbueIr103

MKLINLYLMVLFSDVYGFKQIPLWNEDSIYFSLSLFEEVLSDKKNILVFLPLALDNIPVEWFKNVNAPITIISGNVTEKTMKETEPYQGYALWTEENNFRWQVLQRMGKIKDCECFRSNAFMIIYVVRNNWLVEYSIRSLLSYLWKNYKMYNIVVTPLIRGNNNSSLTNPNIYSYNMFENRIMNLDEGNITKQLDDRFEDMKGYELKVSMFKNYLGYYYKNKYSGLEGTGVDGEFLNLISTRLNFTPIIIAPKGEDFESETFFETKYLQSLKQVSRQQVDFSLNGRYVKTVPGEVKIEYSNVLYEDKICFVVPTGFPYRGWEIFYKACDDIVWLYILVTAIVVSLTVMTIVKVETGRWTSGILLTVSSMFSVPISSTPTSFRLRIIVAVCLIFALVFMTLFQGYLFHMIHTSPKKHLYNTMQDIKNSKIILITGGYNETLDWLKNNVDYETLMHGKWRTSYGFPKNMQHTEATVLISKRHFGFLIDSRRFKAQERPHMLNQCLITHNLAYIFPRGSPFYKIINEKFVARAIEGDFDTFWYNRFISKVLDYKMRRHSLLRKADVRSEEESIPLDKLEISFFILGAGCSVSFIVFLKEVLSCDSPRFKKGKLVKIKHFSNQFTNKTQQKKQSKTQAAKFSTLFRNAKNPPVKEFIL

>GbueIr104

MIPLTWVPLLLSSSVIPVLNSGVKSNLMRDLIVKMISRGKSITNIRCVFSEEAESVPRLLEEFYGRNKPVELFDDRNVTRVFLKDVNKINPDLYVIEPRDDNKFFDRWNQTAKRRRHFSKATFYIIVGETFDDVTGLFDVLEKKFVWNSVVMKIVENDVHVYIGDFNCKKIQGIRHLVYKGGTLDKHIVARTYRYDNCTLRLATEHWPPYVIKENGRLDGAELKLVQEFARWRRYKLDYVDLEIGEEGIWPAVVDSVRNNFSDLGFCALIISDSLNGTDSTKSYLSFYMSFVVPDAYFVPKWLLSVTALSLDINVAILVTAVVFSWSALQIARRETDDPRYRSTGYVATAAFGTFFGQSLKQPSGRLLRCLFAAWYLYTILINVVFNTALTTSATVPIRVQQIDSLGQLVRTRLPLYVSRRMNFVAEILDDEALRAYLERFTVVGLKNKLDVVRKLSDHRNFSILINMDHIDFLTGKRRGNYHVLPEKLLTSSMAVIIQRGDKIISLLDDFIMKVTESGLVAKWLSDSYIYSRPLLELTKTLPDLQAPLTLRDTLLPFIFIVVALTASLLTLLIELIHHKLLNRNIKVFVK

>GbueIr105

MMYRRFRMYKVIVMYSLICCILAQDSFYLHYKHPKSKLILSNRLNNVILNEVKKGPIILEASQLHKFKPLVESNVSRIIVNWSAAYRSFPHSSTIVVLARPRDIKVLLHKSLLSRSKYILVSESFRPLQQCFQAFWKYKILNVIAVLQSKIFHKTVEIFTYRPFSSRCGDTKPKLVGVRYDKLERKSLFPDLRKIKFNHCNLSFTVMDNIPDISILRSGIKIKGGLMKIFVAVREHLEMGVRLWTGEEYWDPDLFAGSKWNGIPGYIWEVLKGRADFSLGRFSWMYHNHSGASFPMQVGMECITWAVPYGAGSFPSSWLLYTNQFNSLTWLLVFIAVLVAGAFMAFLEPRKSQPYLVMLRLLLANGSNKLPVSQSSRIFLVHWAVYSFVFAAAYQASTGSLVLVPPGSGNIETKEQLLESDLVLWTDVEMYKLCERLNFGSEFQEYLQLFPSDIFLDVIYRIYKKKDSAVLSSERSIKYAWNGLRRYSNDTHIISLLSQCLFTSPLSFFLVREGSYLDAPVHRSLRYLSEGGILQLWENVLEDEPTPRNFSQFKNNHPLSIHQTSGAFYILSAGYMLASLAFSIELFYSKKIFIYVSVSRYFKKRINFLN

>GbueIr106

MVYIKVSIILFLMKELQCYSFFSDALKKLERGVISPNFSELFNYTFVNFHCVQVGSDKSVFSTVIEDDLFKAWGPLLVNTLQDDRYCPGLVFISSSISSMAVLDDVYFPRSLIVLPGHITDHNVKALDNVLKGGEGLVVMVNDVRWSVYRTDVGSPRFTQTNLDAGPWSFYIIFGILGYENSIRLLECHRIRMLLKHLELATFNWSFYSMTGPLDKHRRPEWIDGVEMKFIDDLSQIWNFTYSIKMPENSWFWGFILPNGTWSGGILKLVNDREADLGFANVWVTADLLGSEMDISNSWETVCIRFLVPRPTRLGARWSGIFSPFEPPTWILTLVAALFFSLAVNILSTISKQHNYQIKRKFLLAFLSILLGSIFSSSLVSHLTRPKFTAKVESIEALAKEKYTLLSSRRLDVKNIFNMEDKYHRELSRRVMVIKENTDFSKYNVPKNKVVVFSSDLAGAVDVNSLFPDDFLDDFEITSKCLKKTRIGIVMHHNSPYKRLMDKYVMAFIESGLFSKSLSDISKAMAKKRPNIMAVFDLTEKRPQSAIPLAVANLQGAFYLLFLGLVLAFIAFLVEIVSYLLLKRKNETQTYNKMDFYYTSESYTIVEYINYSR

>GbueIr107

MSTRNEVVACLLVTFVLQVQSYEKISQVLERSGIRCMRLDAIWNPWSLGLLKNLSKSSLFMPAIRLDTVGLVSSCNTKLLISESPLEGLKNLRPQLLPVRFLTLFTFQQLPSNITQLNALLEDPLWRKRRSVLLPLNQDIVFVKSEFDHNFTTIFSIDNYTLPEPDKLTGQEISVSTFNCTIYSHLIVDPLTHRGYNTDGVEMRLFEVMTSHLGLKIKIVPASSGHGILEPNSTYTWGSVKYLVTGGQADVGFCELWVTPDLYQRVDMLRPWHSSGIRYLVPRPQHKRYLYAGLESIPSIFWLYLVVTMAACTPLLRLLTTRPPFPTEHLGGGVRSGWWYWQTQLVSIVVTGMPAGGGSRMRTLVPLRLVVTGWSFFSLLVVTVYSCKLVSHLNKPLWEQPLQNEPDMLKAGWIWSRHNIIMTTLKYIYPEERSWLAAWISRHKFDADMAERVRRMKTKKYAVYGQAIGNIYYLPEDSKPPADVLSTYQAADHSVINYSITIMMSKWSGFTPAIRRLFSHLSDAGIVHKIFSDVLLSPTMNRYIVREAFDRCQSCFKEEESEGLGLGVMVGPFILVGLGYTLSTLVFILEILCNRLLRPTLQYTE

>GbueIr108

MFKQLGITVSLIILSYSCFVCTSGIFSNYYYIQYYSSKPIVKELVKINKVMEATFTDNIKCIRIEADSSVFTLGVIKSLIKYRVIPSMRFEFLGLQSFCNTHMLITSYPSSVTKLLKKLTIPVRYLTFVIFRDFGNYNKAAFLKNGVFKNRRAIIVPIEGSSVYSKSELSYTFTKTTADNYTLPVESNYTGQEITVSTFNCTIYSHSVFNPQTGQVSELDGVEMRFFQIMAEHLNLRVKYKFAAGHGSLTPNGSYSWGSAKHAVTAGIADVGFCEFWVTPDLYHKVDMLRPWHKSCVRYLVPKPSHVRYLYAGLEAIPPMFWIILIVLMAVTTPLMRLLTTLEFSNPKLVRRAVPWWYWPVEMVSIVLTGFPTSKMISTSSPCRILVVSWSFLSLLFLAIYSSKLISHMNEPTLRKKIQTTEEMLDAGWIWSRYDIKMNTTDYIYPNETDIIKRWISKHQYDKNMEDRVNKMKTKKYAIYGESIKNIYYLPEESKPPDDILETYEAADTCVISYSITIMMRKWSGFTPAFRKLFSRLSDSGIPEMLFSSVLLSPRMKKYVVSQAFNKAGTGGSKKNVDSLGLDVMIGIFLILMAGSVLATIVFVIELISYRGDKKIKNKKMIPYYN

>GbueIr109N

LLFMKFSESSVSVTRTTGHLFLQVCFFLVSTAYSCSILGYLNSPPLPSTIDSVKDMVEHGLFWVRSTKPTYYPPLFFNMKDPYQAELNSRLQVLPLMRDMDEALSNRRAGVVAQRLQDRFITLDQTVSPQVARTLLPIKQNLATYSVAIGLNKDSPITPHLNRMIGHMKNGGLLDKISRDVRLVWSNNASLTFKSSTDTGLSSSSTKILDLAGLSGAFYLLIAGLLMSTALFLFENIYFIRVRKKINTPTC
