# Supplementary material for: The genome of the water strider Gerris buenoi reveals expansions of gene repertoires associated with adaptations to life on the water
Source: BMC Genomics. 2018 Nov 21;19:832. doi: 10.1186/s12864-018-5163-2 (PMC6249893; doi:10.1186/s12864-018-5163-2)
Supplement: Supplementary file 10 — Gerris buenoi serosins nucleotide and protein sequences. (DOCX 12 kb) [file 12864_2018_5163_MOESM10_ESM.docx]

**Serosins nucleotide sequences**

>Serosin_1 (Scaffold3130)

ATGGCTCGCTACACTCTTCTGTGTGTTATTGCTTCATGCCTGGTTGCCCTTGCTGTTTCGGTGCCTTTTGAGCAGAAAACAGCTTTCGAGTTAAAGGAACGTCACGACTTCTACAACCCTAGGAGCGACAACCCGTTCAGCACGTCTGGATCGGATGCACACATGAAGACACAAAGCGCTAGAGTAGAGCATGACTTCATCGGAGGCAAGAACTGGGCTGCTGGTGGTTACGCTCAACATGAAAGACAGAGCATGTTCGGACAGACCCGTCGTAACAACGAAGGTGGATTCCAATTTAAAGCGAGATTTTAG

>Serosin_2 (Scaffold2193)

ATGGCCCGCTACACTCTCCTCTGTGTTATCGCTTCATGCCTGGTTGCTCTTGCTGTTTCGGTGCCGTTCGAACAGAAAACAGCTTTCGAGTTAAAGGAACGTCACGACTTCTACAACCCTAGGAGCGACAACCCGTTCAGCACGTCTGGATCGGATGCACACATGAAGACACAAAGCGCTAGAGTAGAGCACGACTTTATCGGAGGCAAAAACTGGGCTGCTGGTGGTTACGCTCAACATGAAAGACAGAGCATGTTCGGACAGACCCGTCGTAACAACGAAGGTGGATTCCAATTTAAAGCAAGATTTTAG

>Serosin_3 (Scaffold3130)

ATGGCCCGTTACACTCTCCTCTGTGTTATTGCTTCCTGCCTGGTGGCTCTTGCTGTTTCGGTGCCGTTCGAGCAGAAAACAGCTTTCGAGTTGAAGGAACGTCACGACTTCTACAACCCTAGGAGCGACAACCCGTTCAGCACGTCTGGATCGGATGCACACATGAAGACACAAAGCGCTAGAGTAGAGCATGACTTCATCGGAGGCAAGAACTGGGCTGCTGGTGGTTACGCTCAACATGAAAGACAGAGCATGTTCGGACAGACCCGTCGTAACAACGAAGGTGGATTCCAATTTAAAGCGAGATTTTAG

>Serosin_4 (Scaffold2193)

ATGGCTCGCTACACTCTCCTTTGTGTTATCGCTTCCTGCCTGGTTGCTCTTGCTGTTTCGGTGCCGTTCGAACAGAAAACAGCTTTCGAGTTGAAGGAACGTCACGACTTCTACAACCCTAGGAGCGACAACCCGTTCAGCACGTCTGGATCGGATGCACACATGAAGACTCAAAGCGCTAGAATAGAGCATGACTTTATCGGAGGCAAGAACTGGGCTGCTGGTGGTTACGCTCAACATGAAAGACAGAGCATGTTCGGACAGACCCGACGAAACAACGAAGGTGGATTTCAATTTAAAGCAAGATTTTAG

>Serosin_5 (Scaffold3130)

ATGGCTCGCTACACTCTCCTCTGTGTTATCGCTTCCTGCCTGGTGGCTCTTGCTGTTTCGGTGCCGTTCGAACAGAAAACATCTTTCGACTATAAGGAACGTCACGACTTCCAGGACAACCCGTCTGGATCGGATGCCCACATGAAGACCCAAAGAGCTAGAGTAGAACATGACTTTGTTGGAGGCAAGAACTGGGCTGCTGGTGGTTACGTTCAACACGAAAGACAGACTATGTACGGAGAGACACGTAAGCAAAACGAAGGAGGAGTCCAAGTTAAAGTAACATTTTAG

>Serosin_6 (Scaffold3130)

ATGGTCCGCCACGCTTTGTTTTGTGTTATCGCTTTCTGCCTGGTTACTCTCGCTGTTTCGGTGCCATTTGAGCAGAAAACAGCTTTTGACTACAAGGAACGTCACGACTTCTATAATCCTAAGAACGACAACCCGTTCAGTACGTCTGGATCGGATGCACACATGAAGACACAAAGCGCTAGAGTAGAGCATGACTTTGCCGGAGGCAAGAATTGGGCTGCTGGTTTTTACGCTCAACATGAAAGACAGAATATGAACGGACAGTCCCGTCGTAACAACGAAGCTGGATTCCAATTTAAAGGAACATTTTAG

**Serosins protein sequences**

>Serosin_1 (Scaffold3130)

MARYTLLCVIASCLVALAVSVPFEQKTAFELKERHDFYNPRSDNPFSTSGSDAHMKTQSARVEHDFIGGKNWAAGGYAQHERQSMFGQTRRNNEGGFQFKARF

>Serosin_2 (Scaffold2193)

MARYTLLCVIASCLVALAVSVPFEQKTAFELKERHDFYNPRSDNPFSTSGSDAHMKTQSARVEHDFIGGKNWAAGGYAQHERQSMFGQTRRNNEGGFQFKARF

>Serosin_3 (Scaffold3130)

MARYTLLCVIASCLVALAVSVPFEQKTAFELKERHDFYNPRSDNPFSTSGSDAHMKTQSARVEHDFIGGKNWAAGGYAQHERQSMFGQTRRNNEGGFQFKARF

>Serosin_4 (Scaffold2193)

MARYTLLCVIASCLVALAVSVPFEQKTAFELKERHDFYNPRSDNPFSTSGSDAHMKTQSARIEHDFIGGKNWAAGGYAQHERQSMFGQTRRNNEGGFQFKARF

>Serosin_5 (Scaffold3130)

MARYTLLCVIASCLVALAVSVPFEQKTSFDYKERHDFQDNPSGSDAHMKTQRARVEHDFVGGKNWAAGGYVQHERQTMYGETRKQNEGGVQVKVTF

>Serosin_6 (Scaffold3130)

MVRHALFCVIAFCLVTLAVSVPFEQKTAFDYKERHDFYNPKNDNPFSTSGSDAHMKTQSARVEHDFAGGKNWAAGFYAQHERQNMNGQSRRNNEAGFQFKGTF
